# Clicking acylgermanes: modular access to photoinitiators for visible-light photopolymerization

**DOI:** 10.1039/d6qo00741d

**Published:** 2026-07-15

**Authors:** André Culum, Julia Fuchs, Carolyn Vargas, Fabian Rath, Dagmar Brislinger, Thomas Lainer, Julian Maier, Roland C. Fischer, Ana Torvisco, Anne-Marie Kelterer, Dmytro Neshchadin, Thomas Griesser, Sandro Keller, Tanja M. Wrodnigg, Michael Haas

**Affiliations:** a Institute of Inorganic Chemistry, Graz University of Technology Stremayrgasse 9/IV 8010 Graz Austria michael.haas@tugraz.at; b Institute of Chemistry and Technology of Biobased Systems IBioSys Stremayrgasse 9/II 8010 Graz Austria; c Division of Cell Biology, Histology and Embryology, Gottfried Schatz Research Center, Medical University of Graz; BioTechMed-Graz Neue Stiftingtalstraße 6 8010 Graz Austria; d Institute of Biomechanics, Graz University of Technology Stremayrgasse 16/II 8010 Graz Austria; e Biophysics, Institute of Molecular Biosciences (IMB), University of Graz, NAWI Graz; Field of Excellence BioHealth; BioTechMed-Graz Humboldtstr. 50/III 8010 Graz Austria; f Christian Doppler Laboratory for New Semiconductor Materials based on Functionalized Hydrosilanes Stremayrgasse 9/IV 8010 Graz Austria; g Chemistry of Polymeric Materials, Technical University of Leoben Otto-Glöckel-Strasse 2 8700 Leoben Austria; h Institute of Physical and Theoretical Chemistry, Graz University of Technology Stremayrgasse 9/II 8010 Graz Austria

## Abstract

Herein, we report a modular and biocompatible platform of Cu-catalyzed azide–alkyne cycloaddition (CuAAC)-compatible acylgermanes. Broad functional-group tolerance is demonstrated by a library of novel derivatives that were isolated and characterized by NMR, UV/Vis spectroscopy, and mass spectrometry, with selected structures confirmed by single-crystal X-ray diffraction. We also disclose the first acylgermanes bearing unprotected carbohydrate motifs, whose marked polarity enabled initial biological assessment. Representative examples showed low cytotoxicity and measurable affinity for lipid bilayers in assays with 1-palmitoyl-2-oleoyl-*sn-glycero*-3-phosphocholine (POPC) large unilamellar vesicles. Photo-DSC and photo-CIDNP experiments confirmed efficient photoinitiation and radical pathways characteristic of this class. Coupling acylgermane chemistry with robust CuAAC click methodology expands the scope of acylgermane photoinitiators, enabling rapid late-stage functionalization with diverse motifs and opening routes to complex materials and entry into biological applications.

## Abstract 2

Modular CuAAC click chemistry enables rapid late-stage functionalization of acylgermanes, yielding diverse triazole-linked photoinitiators retaining visible-light photoreactivity for materials design and photochemical applications.

## Graphical abstract

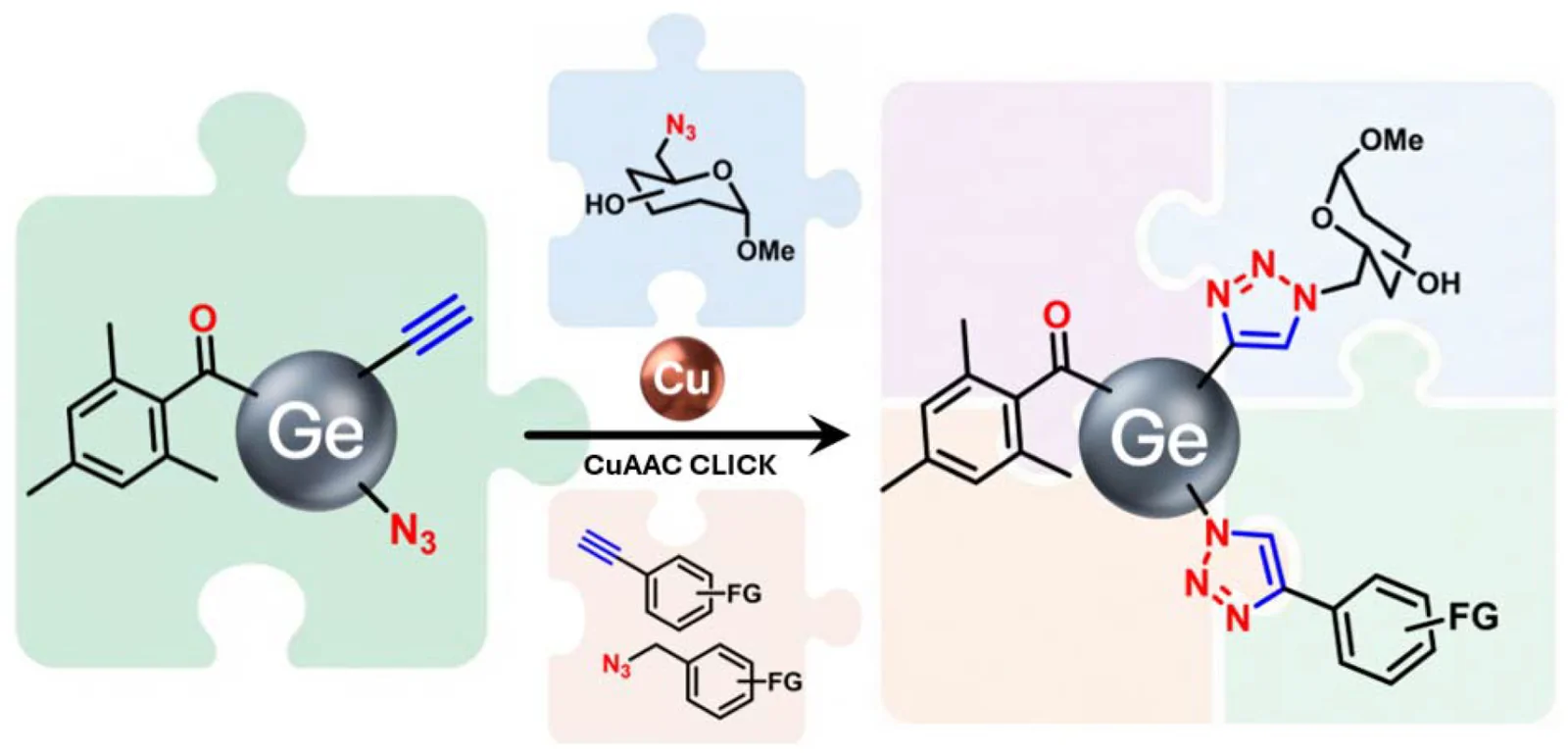

## Introduction

**Fig. 1:**
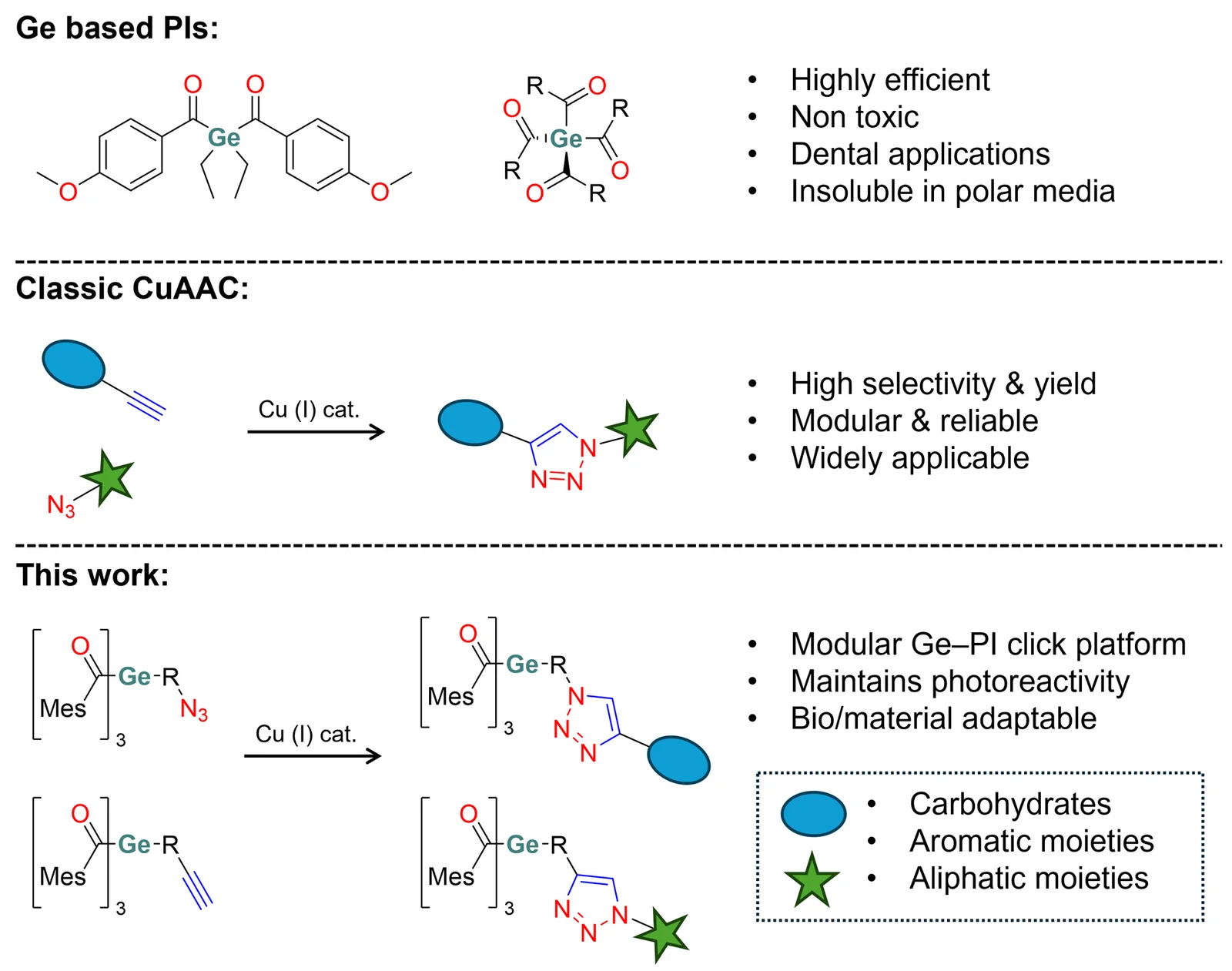
State-of-the-art and this work.

Phosphorus-based photoinitiators (PIs), such as diphenyl-(2,4,6-trimethylbenzoyl)phosphine oxide (TPO), have long been widely used due to their high initiation efficiency and their ability to initiate polymerization upon irradiation with near-UV to visible light. However, increasing regulatory restrictions – most notably the ban of TPO in the European Union due to toxicity concerns – have intensified the search for safer and more sustainable alternatives.^1^ Consequently, the development of non-toxic and biocompatible PIs has become a critical objective.^2–4^ In this context, acylgermanes, a class of germanium-based PIs, have recently attracted significant attention due to their enhanced photochemical efficiency and intrinsically low toxicity.^5–15^ These features make acylgermanes particularly promising candidates for biological and biomaterial applications. Acting as Norrish Type I photoinitiators, they undergo homolytic Ge–C bond cleavage upon irradiation with UV or visible light, generating highly reactive radicals that initiate polymerization.^16^ Their versatility has been demonstrated across a broad range of applications, including dental materials, wavelength-orthogonal polymerizations, and surface modification (Fig. 1, Ge-based PIs).^17–22^

Despite these advantages, two major drawbacks limit their wider application: their poor solubility in polar media and the difficulty of synthetic post-functionalization. To address these challenges, we turned to the well-established copper-catalyzed azide–alkyne cycloaddition (see Fig. 1, classic CuAAC).^23–26^ Recent advances in click chemistry have enabled the development of a wide variety of functional materials, including surfaces, polymers, biomaterials, and even biological systems such as cell membranes, glycans, and enzymes.^27–32^ For example, clickable monosaccharides have become indispensable tools for glycan labeling, while modified polysaccharides provide biocompatible hydrogels suitable for 3D bioprinting^33,34^ as well as bacterial antiadhesives.^35^ The bioorthogonality and low toxicity of azide/alkyne functionalities make them particularly attractive synthetic handles.

Motivated by these advances, we sought to extend click chemistry to acylgermanes, enabling both straightforward functionalization and their integration into complex systems such as surfaces, polymers, and biological environments (see Fig. 1, this work). While organogermanium compounds have been widely used as components in transition-metal-catalyzed cross-coupling,^36–48^ click chemistry with these derivatives remains largely unexplored; only two studies have employed alkyne-substituted germyl groups as precursors.^49,50^

Although numerous organogermanium compounds exhibit well-documented biological activity,^51–55^ the behavior of acylgermanes in biological environments remains poorly understood, with existing studies largely limited to cytotoxicity assessments. Conjugation with biomolecules such as carbohydrates or amino acids would not only improve solubility but also enable more comprehensive and biologically relevant investigations. Such approaches could significantly advance the understanding of acylgermanes in biological systems and expand their potential application in biomedical and biomaterial applications.^56^

With this in mind, we defined the following objectives: (I) establishing a scalable synthesis of click-functionalized acylgermanes; (II) retaining photoreactivity in the resulting conjugates; (III) ensuring stability in polar and aqueous media; and (IV) maintaining low toxicity.

Herein, we present an efficient and concise synthetic route to azide- and alkyne-functionalized acylgermanes, along with a comprehensive structural, photochemical, and initial biological investigation of their triazole conjugates.

## Results and discussion

### Synthesis alkyne-functionalized acylgermanes

**Scheme 1:**
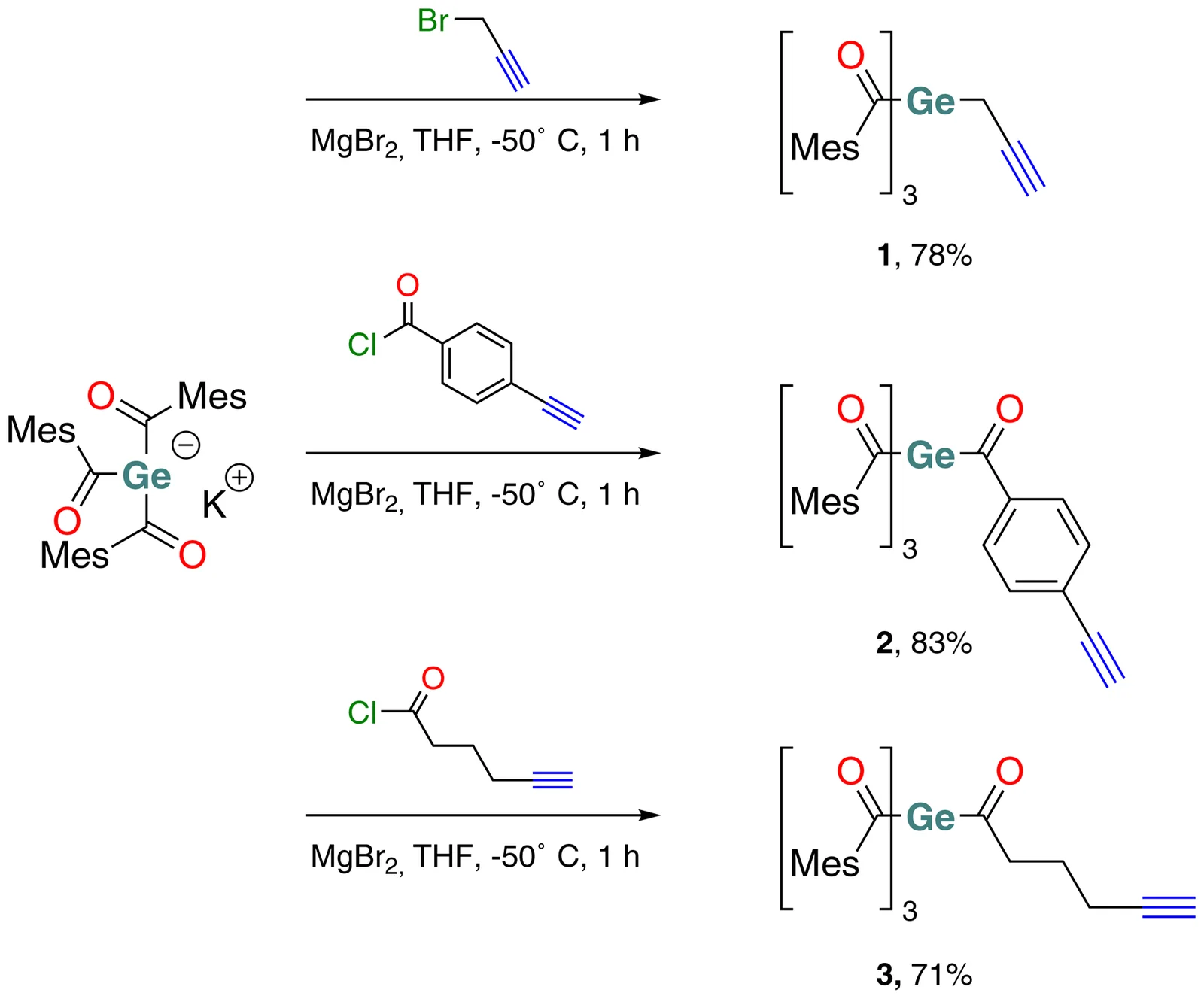
Preparation of alkyne acylgermanes 1–3.

For their synthesis, we employed the well-established potassium trimesitoylgermenolate, KGe[(CO)Mes]_3_, as a robust and accessible starting material.^12^ However, our initial attempts to obtain 1–3 by direct reaction of the germenolate with the corresponding electrophiles were unsuccessful. No target products could be isolated, and the reactions led to inseparable decomposition mixtures. This failure resulted from an acid–base reaction between the basic germenolate and the mildly acidic terminal alkyne (p*K*_a_ ≈ 25), which outcompeted the desired nucleophilic substitution. We resolved this issue *via* simple *in situ* transmetalation of the germenolate with MgBr_2_, which decreased its basicity while maintaining nucleophilicity.^57^ Subsequent reactions with the appropriate electrophiles afforded the desired products 1–3 in good yields (Scheme 1). Notably, the procedure is readily scalable, providing up to 10 g of product without compromising yield or purity. As a result, three differently substituted alkynes were prepared, providing versatile platforms for further synthetic elaboration. The complete synthetic procedures and full characterization data for all compounds are provided in the SI.

### Synthesis of an azide-functionalized acylgermane

**Scheme 2:**
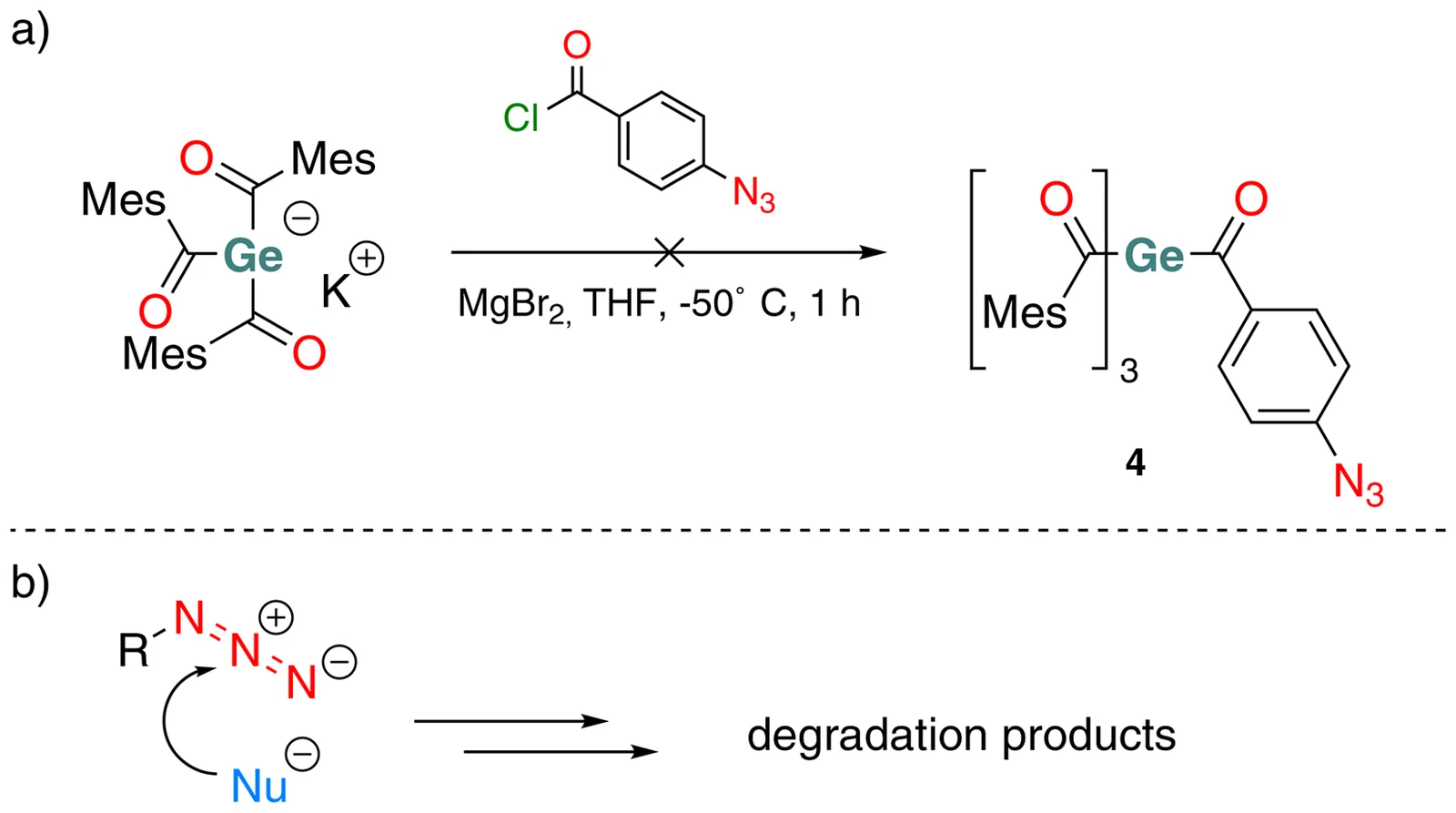
(a) Inaccessible route for the preparation of azide 4. (b) Insertion of a nucleophile into the azide moiety (one mesomeric structure depicted).

The preparation of an azide-functionalized acylgermane proved challenging. Direct reaction of 4-azidobenzoyl chloride with the potassium trimesitoylgermenolate failed to afford the desired product 4, instead yielding an inseparable mixture of decomposition products. During the reaction, the evolution of gas – presumably N_2_ – was observed, suggesting an insertion of the germenolate into the azide functionality (Scheme 2).

**Scheme 3:**
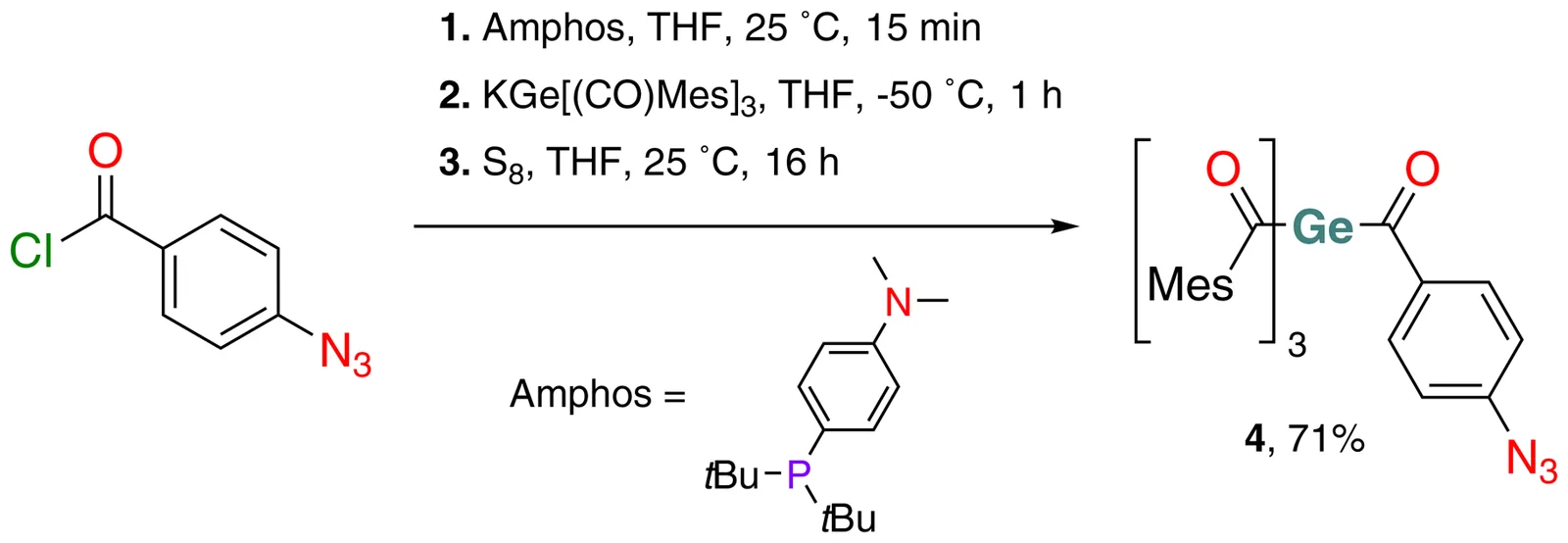
Protecting group mediated synthesis of azide 4.

This behavior is consistent with previous reports by Wang *et al.*, who found that tris(trimethylsilyl)-silyllithium reagents react with azides to form lithium amide compounds.^58^ To prevent this undesired side reaction, the azide moiety was protected *in situ* as a phosphazide. This was achieved by pre-reacting the 4-azidobenzoyl chloride with the Amphos reagent ((4-(*N*,*N*-dimethylamino)phenyl)di-*tert*-butylphosphine) prior to addition of the germenolate. The resulting phosphazide acylgermane 4p was isolated as a dark red oil and exhibited good air and moisture stability (for the structure of 4p please see SI). Subsequent oxidative deprotection with elemental sulfur (S_8_) afforded the desired azide-functionalized acylgermane 4 in an overall yield of 71% (Scheme 3). To the best of our knowledge, this is the first azide-functionalized organogermanium compound reported in the literature.

### General reaction conditions

**Scheme 4:**
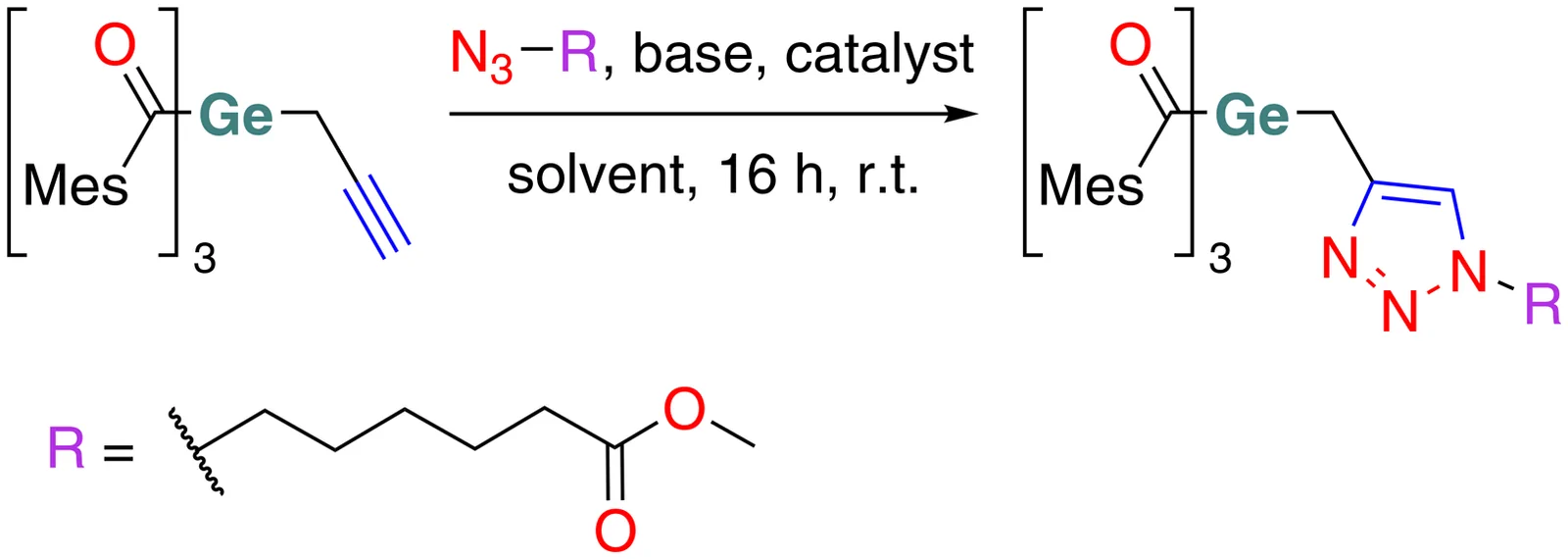
General reaction conditions for the optimization of CuAAC.

Although the CuAAC has been extensively reported under a wide range of conditions, we evaluated the compatibility of our acylgermane substrates with typical click reaction parameters. In particular, we were concerned about potential decomposition or ligand exchange under basic conditions. Therefore, a small-scale screening with compound 1 and methyl 6-azidohexanoate was conducted varying the base, solvent, and copper(i) source (Scheme 4 and Table 1). As expected, the reaction proved highly tolerant: neither parameter had a significant influence on the isolated yields, confirming the robustness and universality of the CuAAC. Importantly, no degradation of the acylgermanes were observed under any of the tested conditions.

**Table 1:** Varied reaction conditions for the optimization of CuAAC

Entry	Base	Solvent	Catalyst	Yield [%]
1	DIPEA	DCM	CuCl	84
2	Et_3_N	DCM	CuI	86
3	Et_3_N	THF	CuCl	80
4	DIPEA	THF	CuI	85
5	Et_3_N	THF	CuI	87

Based on these findings, subsequent reactions were performed in THF using Et_3_N as base and CuI as catalyst, the latter chosen for its slightly higher solubility in apolar media. All reactions proceeded cleanly at room temperature over 16 h. The optimized protocol thus provides a reliable and substrate-tolerant method suitable for future applications of these germanium-based systems. Further experimental details are provided in the SI.

### Synthesis of click-conjugates

**Scheme 5:**
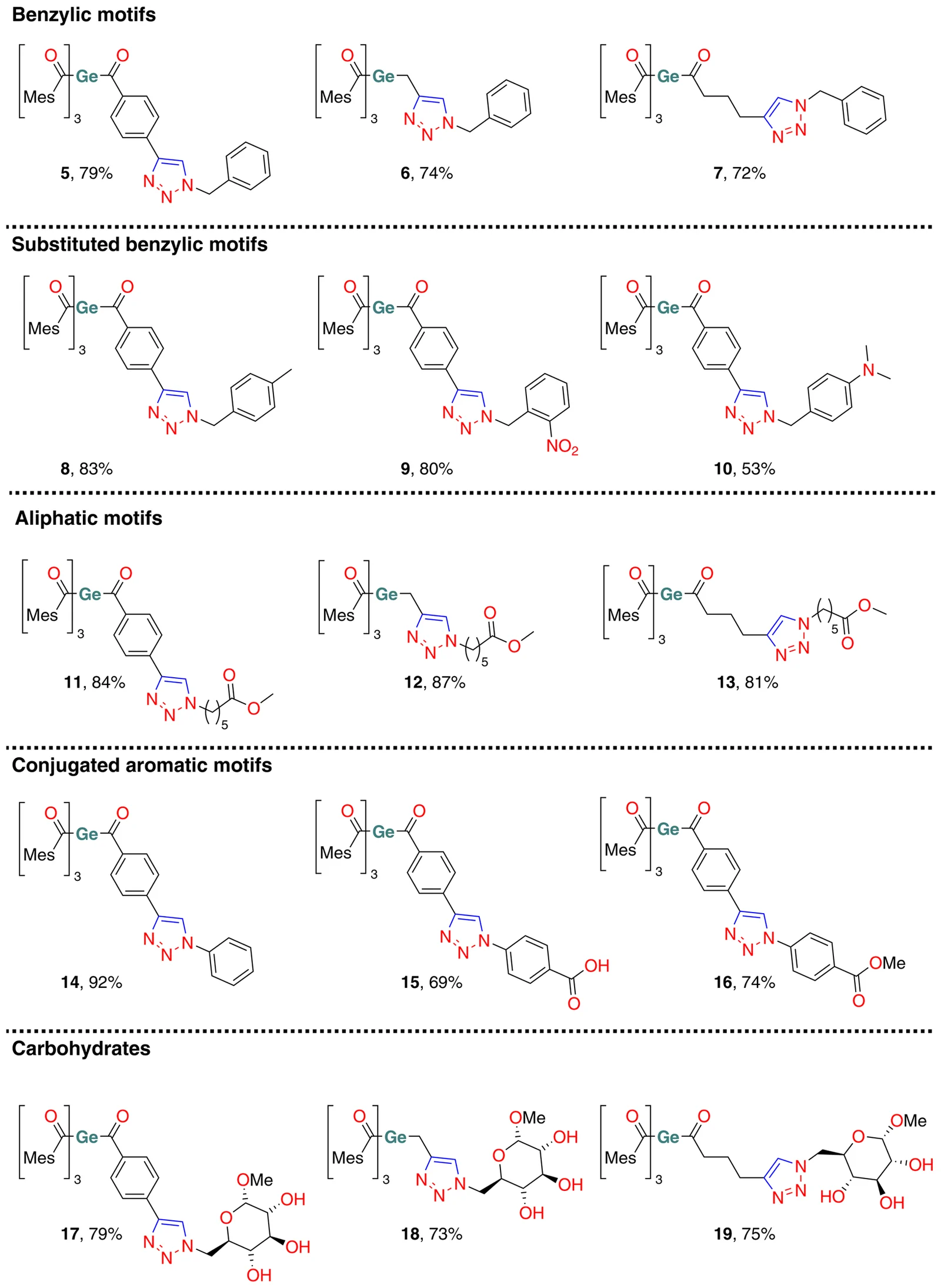
Overview of prepared triazole compounds derived from alkyne substituted acylgermanes 1–3.

**Scheme 6:**
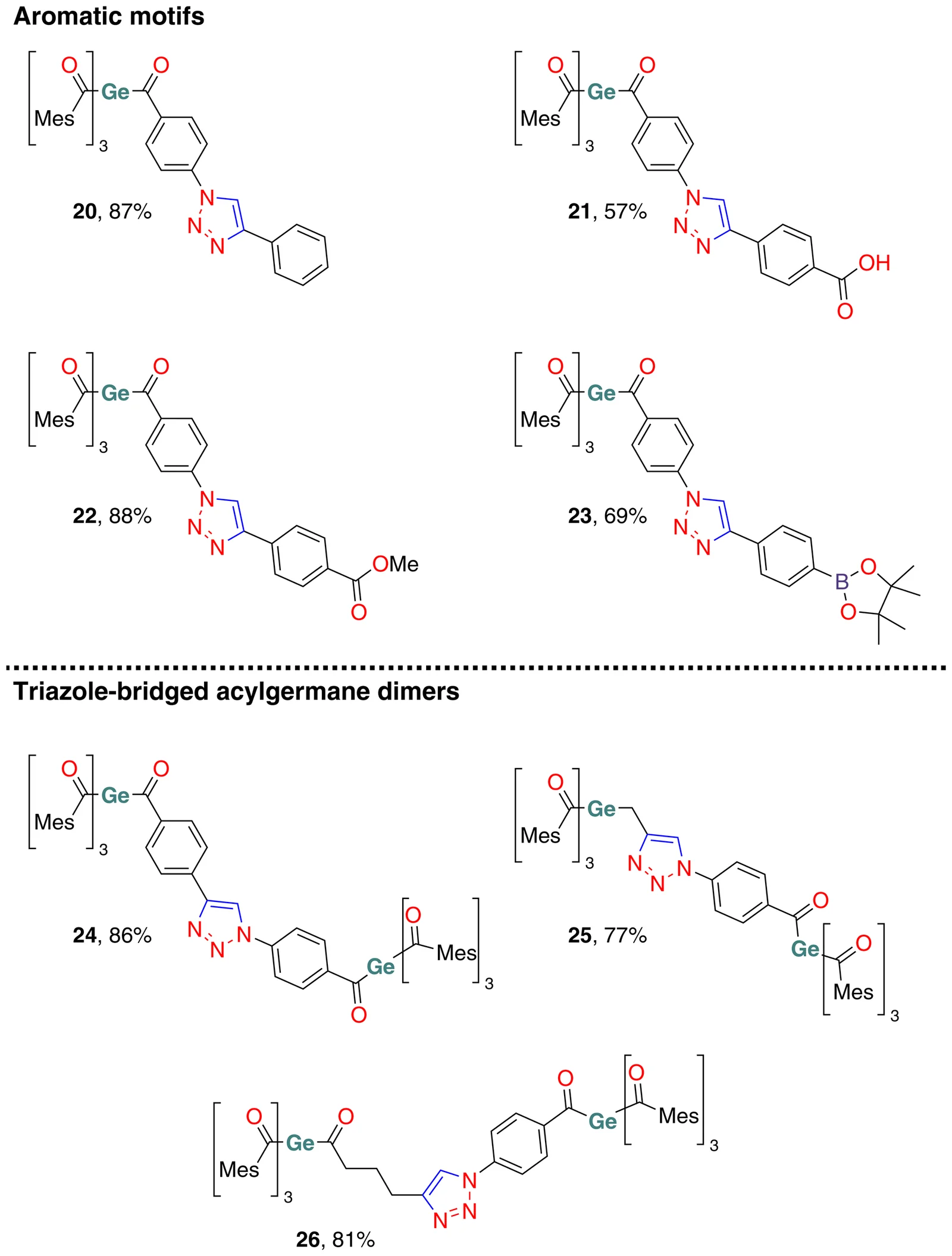
Overview of prepared triazole compounds derived from azide acylgermane 4.

With the successful synthesis of the target alkyne- and azide-functionalized acylgermanes and the establishment of suitable reaction conditions, we next explored the substrate scope of the CuAAC reaction. To facilitate straightforward analysis of the resulting triazole adducts, we focused on small, well-defined molecules representative of potential future applications of these click-capable germanium compounds. The selected reagents cover a broad range of structural classes, including aliphatic, aromatic, and biomolecular motifs. For clarity, the substrate scope is presented in two parts: (I) derivatives of the alkyne substituted acylgermanes 1–3 (Scheme 5), and (II) derivatives of the azide substituted acylgermane 4 (Scheme 6).

Aromatic examples of triazoles 5–7 were synthesized using benzyl azide, while reactions of alkyne 2 with substituted benzyl azides furnished compounds 8–10, enabling the investigation of electronic and spectroscopic substituent effects. Notably, compound 10 was obtained in slightly lower yield, likely due to partial degradation of the starting material in the presence of the amine functionality. Compounds containing aliphatic moieties 11–13 were prepared from a simple hexanoic acid ester as a model substrate, affording the desired products in excellent yields.

To evaluate the influence of extended conjugation, we next prepared compounds 14–16, lacking the benzylic methylene spacer. These derivatives, characterized by their long conjugated π-systems, displayed markedly reduced solubility in common organic solvents, presumably due to increased crystal packing interactions. Compound 15 was particularly prone to degradation (see SI Fig. S43), an effect attributed to the presence of a carboxylic acid group, likely due to proton-induced decomposition of the acylgermane core – a trend later confirmed for compound 21 (Scheme 6).

To enhance the polarity and facilitating biological integration, we also synthesized carbohydrate-based triazoles 17–19 from methyl 6-azido-6-deoxy-α,d-glucopyranoside, an easily accessible glucose derivative. These compounds were isolated in excellent yields (73–79%) and exhibited high solubility in methanol and acetonitrile. Owing to their stability in polar media and good solubility in DMSO/water mixtures, they serve as ideal model systems for future cytotoxicity assays and membrane-interaction studies.

Triazoles 20–22 (Scheme 6), obtained from azide 4, were synthesized under the established reaction conditions. The carboxylic acid 21 exhibited reduced yield and pronounced instability, mirroring the behavior observed for 15 and indicating a general sensitivity of this motif toward acidic functionalities. The boronic acid pinacol ester 23 was obtained in good yield (69%), underscoring the potential of these compounds as versatile intermediates for subsequent functionalization, such as Suzuki–Miyaura cross-coupling, and further expanding the synthetic utility of acylgermane derivatives.

To further explore the modularity of our system, we also performed the CuAAC reaction using both precursor types, which yields triazole-bridged acylgermane dimers 24–26 in excellent yields. These dimers combine the spectroscopic features of their respective precursors. For instance, compound 26 contains aromatic, and aliphatic acyl substituents at the germanium centers, exhibiting all three characteristic UV/Vis absorption bands. Full synthetic procedures and characterization data are provided in the SI. To assess whether these structural modifications affect the photophysical properties, the compounds were further investigated by UV/Vis spectroscopy.

The successful synthesis of a diverse library of triazole conjugates underscores the modularity of this approach, enabling rapid late-stage functionalization of acylgermanes without perturbing the photochemically active core. Notably, electronic and structural variations introduced *via* the click reaction exert only minor influence on reaction efficiency, highlighting the robustness and broad applicability of the CuAAC protocol. However, the reduced stability observed for carboxylic acid-containing derivatives reveals an inherent limitation of the acylgermane scaffold under acidic conditions, establishing an important guideline for future molecular design. The incorporation of carbohydrate motifs significantly enhances solubility in polar media and provides a promising entry point for biological applications, including membrane interaction studies and potential targeting strategies. Together, these findings demonstrate that click conjugation represents a powerful and versatile strategy to tailor acylgermane photoinitiators for advanced materials and biological environments.

### UV/Vis spectroscopy

**Fig. 2:**
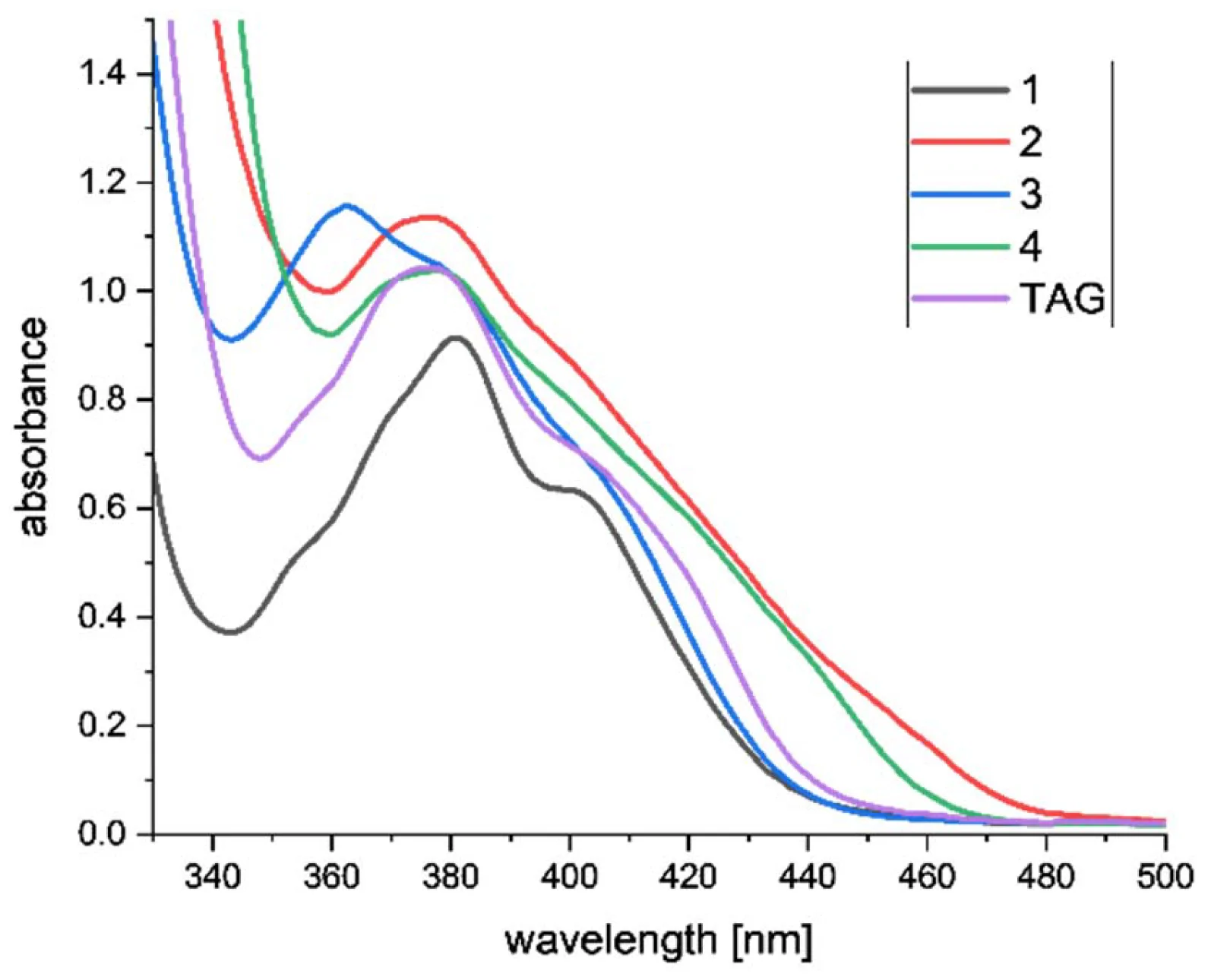
UV/Vis spectra of 1–4 compared to tetramesitoylgermane (TAG) in THF (5 × 10^−4^ mol L^−1^).

A fundamental step in assessing photochemical properties of the synthesized acylgermanes is the characterization of their absorption behavior by UV/Vis spectroscopy. Measurements were conducted in THF, chosen for its good solubilizing properties across all derivatives and its moderate polarity, which reasonably reflects environments relevant for photopolymerization applications. The alkyne precursors 1–3 and azide derivative 4 exhibit the characteristic absorption features of acylgermanes (Fig. 2). The triacylgermane 1 shows overall lower absorption intensity and more pronounced fine structure, consistent with the lower number of chromophores substituted at the germanium center. In contrast, the tetraacylgermanes 2–4 display nearly identical spectral shapes, with the aliphatic derivative 3 showing a slight hypsochromic shift relative to the aromatic analogues. Notably, compound 2 exhibits the most pronounced bathochromic shift of the absorption edge within the series. The broad intense band below 350 nm corresponds to π → π* transitions of the aromatic chromophores, while weaker n/σ → π* transitions with visible fine structure extend from 350 nm to 480 nm. These transitions were computationally assigned using the CAM-B3LYP/def2-TZVP/CPCM(THF) method (see SI).

**Fig. 3:**
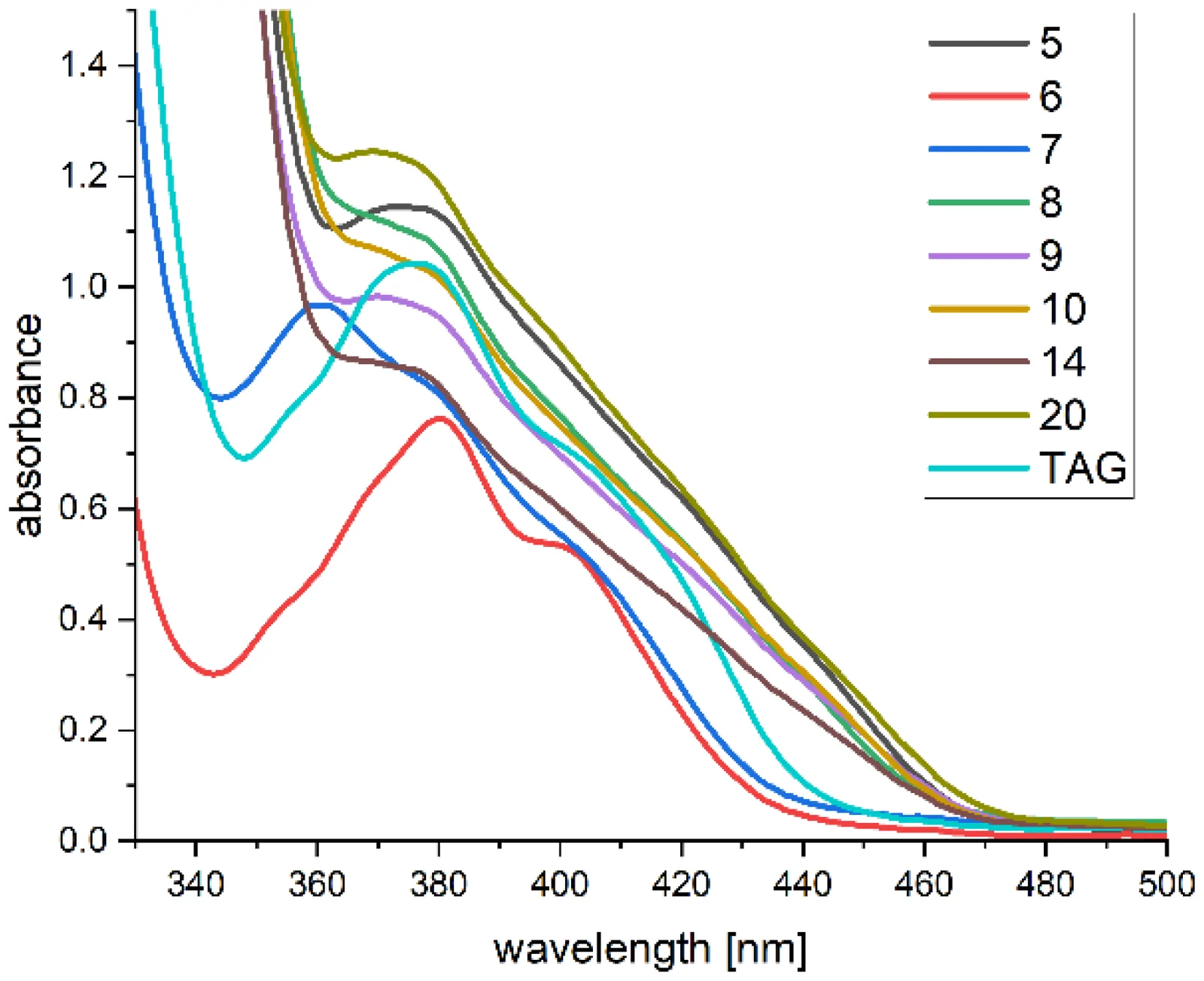
UV/Vis spectra of 5–10, 14 and 20 compared to tetramesitoylgermane (TAG) in THF (5 × 10^−4^ mol L^−1^).

A comparable absorption behavior is observed for all triazole conjugates. The overall spectral shapes and absorption intensities remain essentially unchanged upon triazole formation, demonstrating that the acylgermane chromophore is electronically unaffected by click conjugation. This indicates effective electronic decoupling of the triazole substituents from the photoactive core. Accordingly, all derivatives retain the characteristic combination of π → π* and n/σ → π* transitions extending into the visible region. Representative spectra of compounds 5–10, 14, and 20 are shown in Fig. 3.

**Fig. 4:**
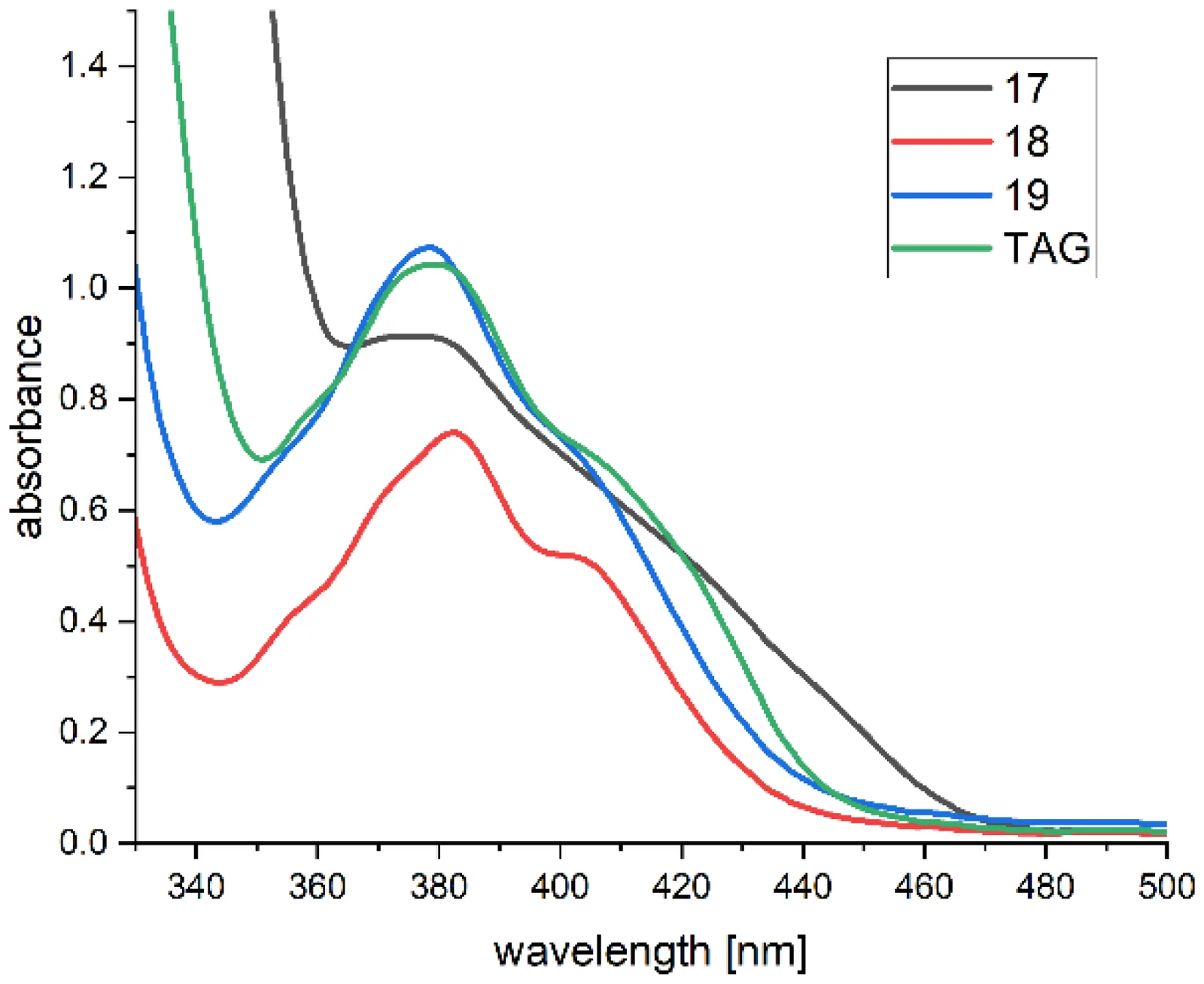
UV/Vis spectra of 17–19 compared to tetramesitoylgermane (TAG) in THF (5 × 10^−4^ mol L^−1^).

**Fig. 5:**
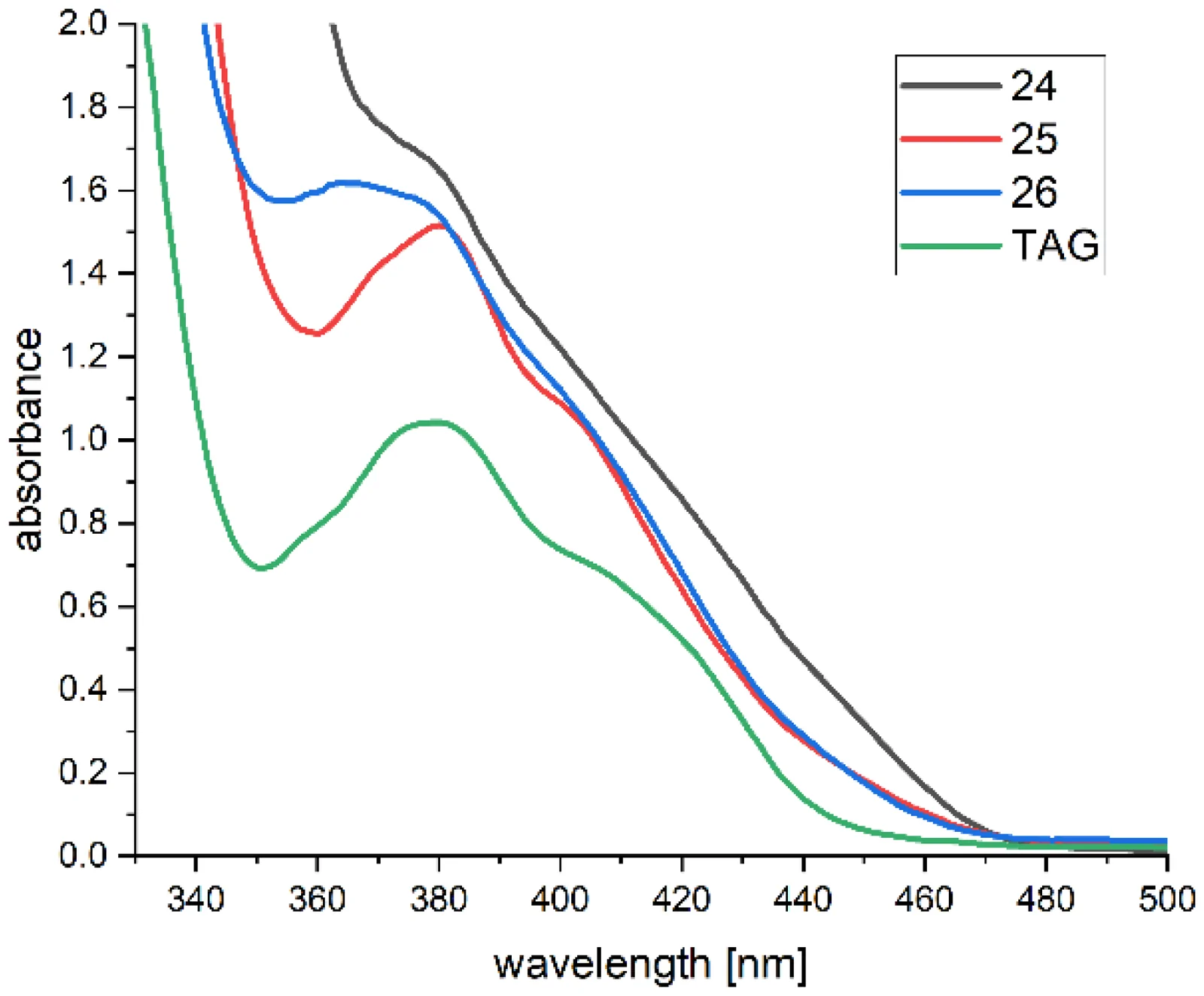
UV/Vis spectra of 24–26 compared to tetramesitoylgermane (TAG) in THF (5 × 10^−4^ mol L^−1^).

The same holds true for the carbohydrate-substituted triazole derivatives 17–19 (see Fig. 4), which are of particular interest due to their enhanced solubility in polar media (see Solubility section) and their potential applicability in aqueous or biologically relevant photopolymerization systems Moreover, the triazole-bridged acylgermane dimers, which allow for the incorporation of multiple acyl substituents, also retain the characteristic absorption profile. However, due to the increased number of chromophores, these systems display higher extinction coefficients and a more pronounced tailing toward longer wavelengths compared to simpler triazole-linked systems (see Fig. 5).

Taken together, these results demonstrate that both, the precursor compounds and their click-derived conjugates, retain the photophysical properties of simple acylgermanes. The electronic robustness of the chromophore, combined with its tolerance toward extensive structural modification, highlights the suitability of this platform for the design of functional photoinitiators. Importantly, the ability to introduce diverse substituents without perturbing the absorption characteristics provides a key advantage for tailoring acylgermanes toward specific applications in advanced materials and emerging biological contexts.

### DFT calculations

**Fig. 6:**
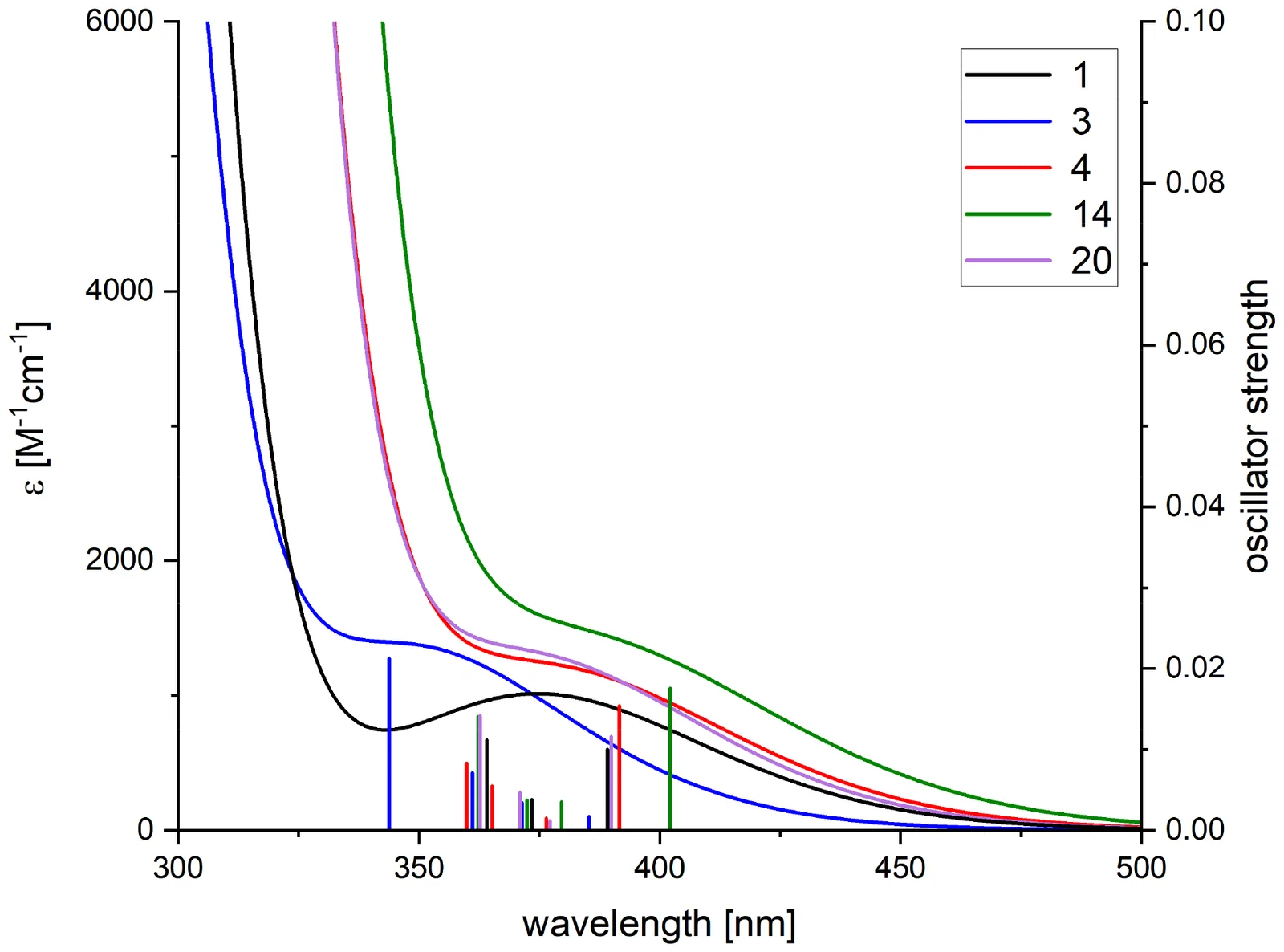
Simulated absorption spectra highlighting the long-wavelength band of compounds 1, 3, 4, 14, and 20. CAM-B3LYP/def2-TZVP/CPCM(THF) method with a Gaussian broadening of 2500 cm^−1^.

To gain detailed insight into the absorption characteristics, we have computed the vertical excitations of representative compounds, namely 1, 3, 4, 14, and 20. In these compounds, the low-intensity band between 340 nm and 480 nm consists of four n → π* transitions (Fig. 6), which are red-shifted relative to 3 when the aromatic system is introduced by a phenyl group in 4 and 20, and further red-shifted in the conjugated aromatic molecule 14. Compound 1 has a lower intensity in the UV spectrum compared to the other compounds because only the three mesitoyl groups contribute to the long-wavelength band.

The high-intensity band around 270–290 nm consists of π → π* excitations (see SI Fig. S117). The relevant natural transition orbitals (NTOs) for excitations S_1_–S_6_ are depicted in Tables S4–S7 with their linear combination coefficients for *c* > 0.1. These NTOs show that the triple bond in 1 and 3 is not involved in the long-wavelength band, and also the phenyl rings at the end of the aromatic systems in 14 and 20 do not contribute to the long-wavelength band. This result aligns with the experimental UV/Vis measurements and indicates that the triazole substituents are electronically decoupled from the transitions responsible for the long-wavelength absorption.

### Solubility

One of the primary objectives of this study was to enhance the polarity of acylgermanes to enable investigations and photopolymerizations in water or under biologically relevant conditions. This aspect is highly relevant for the future application of acylgermanes as photoinitiators in more sustainable and environmentally friendly systems.

Accordingly, the solubility of carbohydrate derivatives 17–19 was examined in aqueous DMSO mixtures commonly used for biological assays (Table 2). Because cytotoxicity and membrane-interaction studies require minimal organic cosolvent, a target concentration of ≥50 µM in 0.5% DMSO/water was defined. All compounds fulfilled this requirement, demonstrating that carbohydrate conjugation substantially improves aqueous compatibility.

**Table 2:** Solubility of carbohydrate derived compounds. Solvent mixtures refer to % of given solvent in water

Solvent mixture	Compound solubility [mg mL^−1^]		
	17	18	19
50% DMSO	1.270	1.142	1.428
5% DMSO	0.059	0.053	0.062
1% DMSO	0.045	0.041	0.047
0.5% DMSO	0.043	0.039	0.045
100% MeOH	181.1	201.0	227.7
20% MeOH	0.702	1.203	1.326

Solubility was additionally determined in methanol and methanol/water mixtures, as methanol is routinely used as a stock and measurement solvent in photochemical studies, including the ^1^H photo-CIDNP experiments described below. The derivatives displayed excellent solubility in MeOH (>180 mg mL^−1^) while remaining soluble in aqueous mixtures, highlighting their balanced amphiphilic character.

### Cytotoxicity assessment

**Fig. 7:**
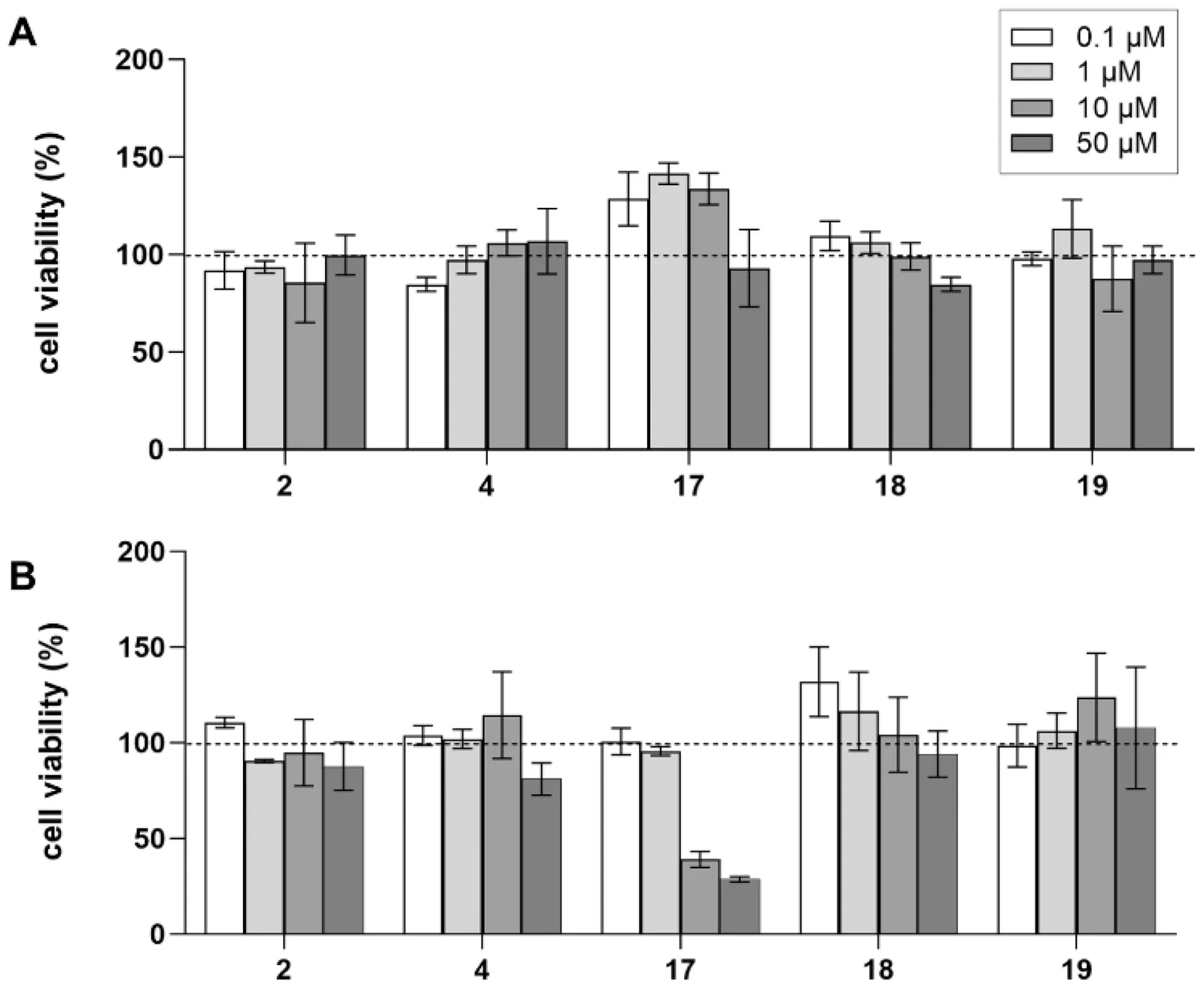
Cytotoxicity of the compounds 2, 4, 17, 18 and 19 in HT29 cells. HT29 epithelial colorectal cancer cells were treated with increasing concentrations (0.1, 1, 10, and 50 µM) of compounds for (A) 24 h and (B) 48 h. Cell viability was assessed using the cytotoxicity assay and normalized to untreated control cells. Compounds did not substantially impair cell viability at concentrations up to 10 µM, whereas a significant decrease in viability was detected at 50 µM, especially after 48 h of treatment. Data are presented as mean ± SD from 3 independent measurements.

To evaluate the biocompatibility of the synthesized PIs, we decided to perform cytotoxicity screenings on a select set of representative compounds. The cytotoxic profiles of compounds 2, 4, 17, 18, and 19 were evaluated in HT29 epithelial colorectal cancer cells using the cytotoxicity assay at concentrations of 0.1, 1, 10, and 50 µM following 24 h (Fig. 7A) and 48 h (Fig. 7B) of incubation. Across all compounds, cell viability remained close to control levels at concentrations up to 10 µM after both 24 h and 48 h of treatment, indicating limited cytotoxicity within this dose range.

A reduction in viability was observed at 50 µM, although the extent of this effect varied among the compounds. After 24 h, compound 17 showed the clearest viability decrease at 50 µM, whereas compounds 2, 4, 18, and 19 exhibited only moderate reductions. Following 48 h of exposure, cytotoxicity at 50 µM became more pronounced for compounds 4 and 17, while compounds 2, 18, and 19 continued to show only modest decreases relative to the control. These findings indicate a mild dose-dependent response for selected compounds, with the most notable decline in metabolic activity occurring at the highest concentration and extended incubation time.

Overall, compounds 2, 4, 17, 18, and 19 demonstrated low cytotoxicity toward HT29 cells at concentrations ≤10 µM, while moderate viability reductions emerged at 50 µM in a compound- and time-dependent manner. Experimental details are provided in the SI.

### Membrane interactions

Since one potential application of water-soluble acylgermanes involves their use in biological environments, the interactions of carbohydrate derivatives 17–19 with phospholipid membranes were evaluated using 1-palmitoyl-2-oleoyl-*sn-glycero*-3-phosphocholine (POPC) large unilamellar vesicles (LUVs) as a model system. Membrane partitioning thermodynamics were quantified by isothermal titration calorimetry (ITC) experiments (Fig. S12). All three compounds exhibited exothermic binding to POPC LUVs, with integrated heats that smoothly decreased in magnitude with increasing lipid concentration, consistent with a simple partition equilibrium. Global fitting yielded intrinsic partition coefficients and the thermodynamic quantities summarized in Table 3. Among the two tetraacylgermanes, compound 19 displayed roughly three-fold higher membrane affinity (*K*_0_ = 2.1 × 10^5^) than 17 (*K*_0_ = 7.9 × 10^4^), despite a less exothermic transfer enthalpy (19: −7.4 kJ mol^−1^, 17: −9.4 kJ mol^−1^). The enhanced partitioning of 19 is thus driven predominantly by a more favorable entropic contribution (19: −*T*Δ*S*° = −22.9 kJ mol^−1^, 17: −*T*Δ*S*° = −18.5 kJ mol^−1^), consistent with a stronger hydrophobic effect associated with its aliphatic butanoyl linker, which presents greater nonpolar surface area than the aromatic spacer in 17.^59^ Triacylgermane 18, which carries one fewer acyl group than 17 and 19, exhibited the lowest partition coefficient (*K*_0_ = 5.7 × 10^4^) but the most exothermic transfer enthalpy (−13.5 kJ mol^−1^). Its weaker overall partitioning reflects a smaller yet still favorable entropic contribution (−*T*Δ*S*° = −13.6 kJ mol^−1^), consistent with its reduced nonpolar surface area that is desolvated upon membrane insertion.

**Table 3:** Thermodynamic parameters characterizing the partitioning of organogermanium compounds into POPC membranes at 25 °C

Compound	*K*_0_ (×10^4^)	Δ*G*° (kJ mol^−1^)	Δ*H*° (kJ mol^−1^)	−*T*Δ*S*° (kJ mol^−1^)
18	5.7 [4.6–6.8]	−27.1 [−27.6 to −26.6]	−13.5 [−16.1 to −11.5]	−13.6 [−16.1 to −10.5]
17	7.9 [6.3–9.8]	−27.9 [−28.5 to −27.4]	−9.4 [−11.1 to −8.0]	−18.5 [−20.5 to −16.3]
19	21 [13–38]	−30.3 [−31.9 to −29.2]	−7.4 [−9.1 to −6.3]	−22.9 [−25.6 to −20.1]

The thermodynamic signature observed across the compound series—increasingly favorable entropy compensating for decreasingly favorable enthalpy—is characteristic of the classical hydrophobic effect.^59^ The aliphatic spacer in 19 presents greater hydrophobic surface area than the direct linkage in 18 or the aromatic spacer in 17, leading to more extensive ordering of water molecules in the unbound state and, consequently, greater entropic gain upon membrane partitioning.

### Photo-DSC analysis

The photoreactivity of the synthesized germanium photoinitiators was investigated using photo-differential scanning calorimetry (Photo-DSC). Measurements were evaluated in terms of total heat evolution and the time required to reach 95% conversion (*t*_95%_). Representative Photo-DSC curves are provided in the SI (Fig. S111).

Carbohydrate derivatives 17–19 were once again selected due to their unique structural motifs. All three initiators exhibited comparable polymerization kinetics, reaching 95% conversion within a similar time range (Table 4). Only minor variations in total heat release were observed, with initiator 18 showing the highest heat evolution (464 mJ mg^−1^) and initiator 19 the lowest value (403 mJ mg^−1^). These results confirm that the click-derivatives are fully competent photoinitiators, displaying efficiency comparable to that of previously reported acylgermanes.

**Table 4:** Results of the Photo-DSC measurements of three germanium-based photoinitiators

Compound	Heat of polymerization [mJ mg^−1^]	*t*_95%_ [s]
17	439	30.0
18	464	36.6
19	403	34.2

### ^1^H photo-CIDNP experiments

**Fig. 8:**
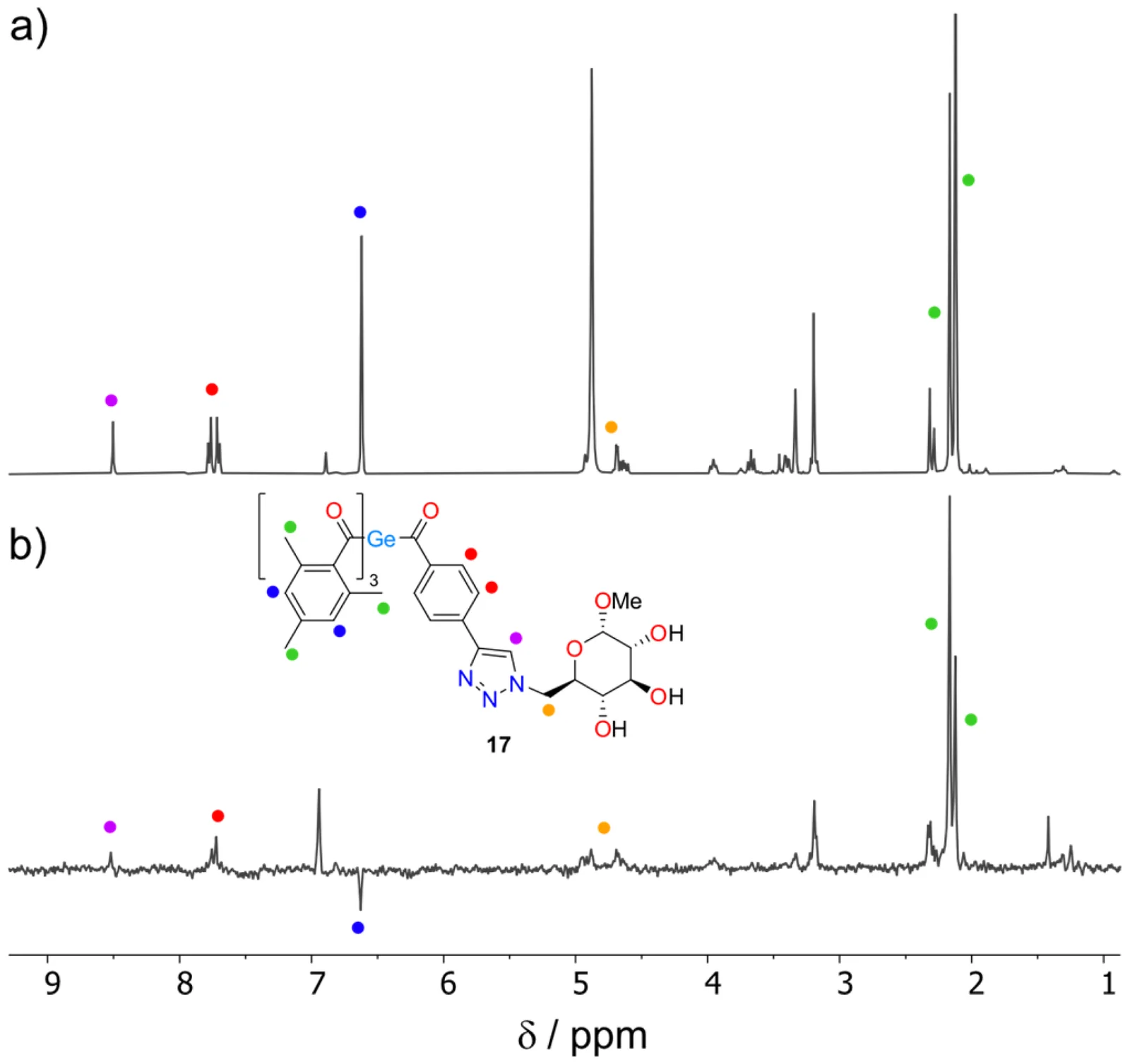
^1^H NMR (a) and photo-CIDNP (b) spectra of 17 recorded methanol-d_4_ 2 µs after the laser flash (355 nm, 8 ns).

To rationalize the photo-DSC results and gain mechanistic insight into the photopolymerization of our compounds, we turned to photo-CIDNP. Compound 17 was selected as a representative initiator candidate. Time-resolved photochemically induced dynamic nuclear polarization photo-CIDNP^60^ experiments were performed on the microsecond timescale; the resulting ^1^H NMR and photo-CIDNP spectra of 17 in methanol-d_4_ are shown in Fig. 8. The CIDNP spectrum is dominated by polarizations arising from 17, and no significant product-related signals were detected within the accessible timescale and sensitivity limits of the experiment. Geminate recombination is dominant on this timescale. This behavior is consistent with reversible photo-induced α-cleavage of 17, yielding a germanium- and a carbon-centered radical. Two competing pathways can be envisaged: α-cleavage generating the mesitoyl radical (pathway I) or the *p*-triazolylbenzoyl radical (pathway II). Recombination of the geminate radical pair produces the observed CIDNP signals. Since the magnitude and sign of CIDNP effects are governed by the hyperfine coupling constants (HFCs) of the radical pair partners, the CIDNP effect provides a sensitive probe to distinguish between the two α-cleavage pathways. QM calculations (see SI) indicate that in both the mesitoyl and *p*-triazolylbenzoyl radicals, unpaired electron density is partly delocalized onto the aromatic ring system. The resulting non-zero proton HFCs account for the observed polarization of aromatic protons in both fragments (Fig. 8), confirming that both α-cleavage pathways are operative.

This analysis is admittedly simplistic, as it does not account for contributions arising from the germanium-centred radicals. Due to their size and structural complexity, reliable calculation and unambiguous assignment of the corresponding HFCs are currently not feasible, rendering a quantitative analysis of their CIDNP contributions impractical at this stage.

**Fig. 9:**
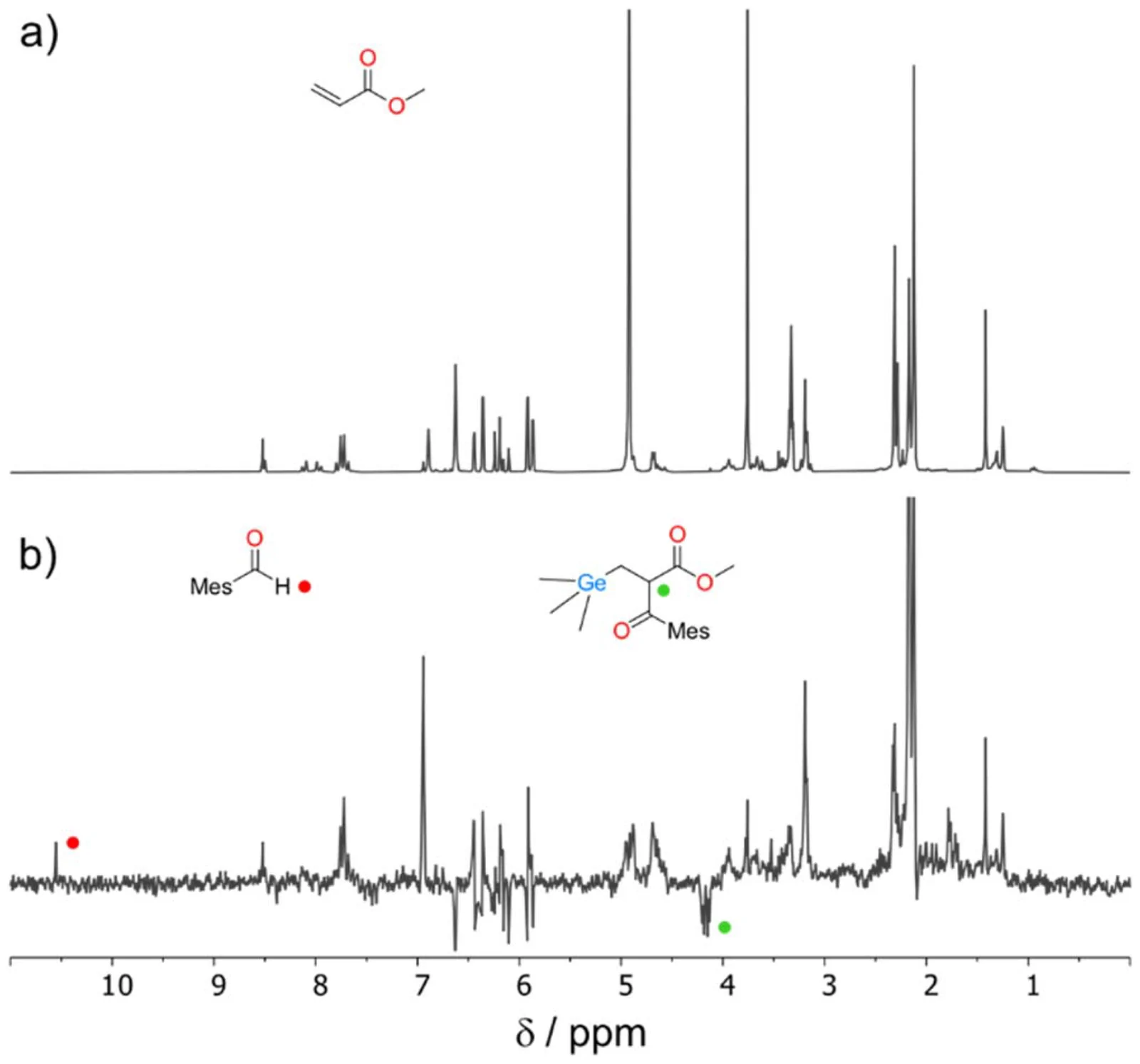
^1^H NMR (a) and photo-CIDNP (b) spectra of 17 in the presence of methyl acrylate recorded methanol-d_4_, 2 µs after the laser flash (355 nm, 8 ns).

Photo-CIDNP experiments performed in the presence of methyl acrylate as a radical trap allow us to assess whether the primary radicals generated *via* α-cleavage of 17 are capable of adding to the monomer double bond and thereby initiating polymerization. The ^1^H CIDNP spectrum shown in Fig. 9 confirms that both the germanium- and carbon-centred radicals add to the C=C bond of methyl acrylate, as evidenced by polarization of the addition products and their subsequent involvement in radical termination processes. CIDNP effects observed for the alkene protons of the monomer provide independent confirmation of radical addition to the double bond. Notably, the unusually large polarizations of both diastereotopic protons of 17 (CH_2_, 4.5–5 ppm) are consistent with substantial spin density delocalization from the phenyl ring into the triazole system of the *p*-triazolylbenzoyl radical, as supported by the QM calculations (SI). Extended spin delocalization stabilizes this radical and is expected to reduce its addition rate to the monomer double bond. Consequently, the *p*-triazolylbenzoyl radical contributes more to cage recombination relative to the mesitoyl radical, which accounts for the comparatively larger polarization intensity of the *p*-triazolylbenzoyl fragment signals. The lower reactivity of *p*-triazolylbenzoyl radicals is also in agreement with the absence of the triazolylbenzoyl aldehyde, as the row of polarized products, which could have been formed as the result of the hydrogen transfer after the first addition of Ge-centred radical to the double bond.^61^ The reactivity pattern of 4 in the presence of monomer closely resembles that of 17 (see SI*).* Alkyne- and azide-functionalized acylgermanes 2 and 3 also undergo α-cleavage upon irradiation, however their follow-up reactivity is complicated by the inter/intra-molecular addition of the primary radicals to the triple bonds (see CIDNP spectra in the SI).

### X-Ray crystallography

**Fig. 10:**
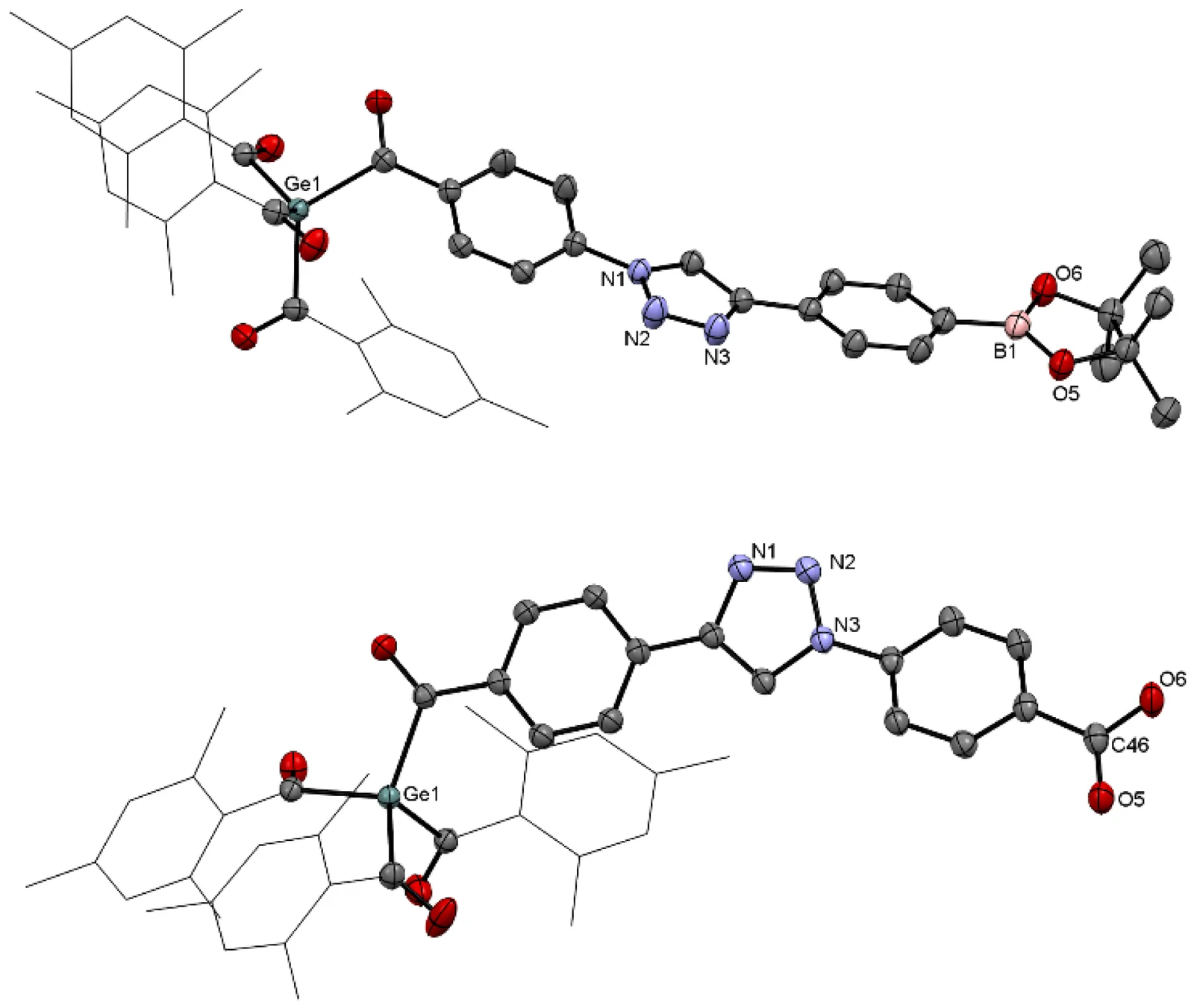
ORTEP representation of compounds 15 (above) and 23 (below). Thermal ellipsoids are drawn at the 50% probability level. Hydrogen-atoms are omitted for clarity and the mesitoyl groups are presented in wireframe style.

Single-crystal X-ray diffraction analyses were performed for alkynes 1–3, azide 4, protected azide 4p, and six triazole compounds (14, 15, 16, 20, 21, 23). This further confirmed the proposed connectivity and unambiguously established the 1,4-triazole connectivity (see Fig. 10 as representative examples are the ORTEP representations of 15 and 23). All compounds possess comparable Ge–C and C=O bond metrics, and no unusual structural features were observed.

These data unequivocally support the proposed molecular structures and further demonstrate the crystallinity and stability of this novel class of click-derived acylgermanes. Complete crystallographic data and additional structures are provided in the SI.

## Conclusion

In this study, we reported the synthesis of CuAAC-compatible acylgermanes, comprising three alkyne-functionalized derivatives and one azide. Their versatility and functional group tolerance were demonstrated through the preparation of a library of 22 novel compounds, all of which were isolated and fully characterized by NMR and UV/Vis spectroscopy as well as mass spectrometry (HRMS). In addition, the molecular structures of 11 compounds were confirmed by single-crystal X-ray diffraction. Notably, compounds 17–19 represent the first examples of acylgermanes bearing unprotected carbohydrate motifs. The pronounced polarity of these derivatives enabled initial biological investigations. Cytotoxicity assays conducted on a representative subset further support the low toxicity profile of acylgermanes. Complementary studies on their interaction with lipid membranes using POPC large unilamellar vesicles provided valuable insight into their membrane affinity. In addition, photo-DSC and photo-CIDNP experiments confirmed their efficiency as photoinitiators, displaying reactivity and radical pathways characteristic of this compound class.

Taken together, these results broaden the application scope of acylgermane photoinitiators, allowing the introduction of diverse functionalities *via* well-established CuAAC click chemistry. This allows acylgermane chemistry to lean on the extensive click chemistry toolbox and facilitating the development of increasingly complex materials.

## Author contributions

A. C., T. M. W. and M. H. conceptualized the project. A. C. performed the experiments and analyzed the results. J. F., C. V., F. R., D. B., T. L., J. M., A. T., A.-M. K., R. C. F., and D. N. assisted in performing the experiments and contributed to data analysis. T. G., T. M. W., S. K., and M. H. supervised and directed the project. A. C. and M. H. wrote the manuscript with input from all coauthors. Funding was acquired by M. H.

## Conflicts of interest

There are no conflicts to declare.

## Supplementary Material

QO-013-D6QO00741D-s001

QO-013-D6QO00741D-s002

## Data Availability

The data that support the findings of this study are available in the supplementary information (SI). Supplementary information is available. See DOI: https://doi.org/10.1039/d6qo00741d. The authors have cited additional references within the SI.^62–79^ CCDC 2541768–2541778 contain the supplementary crystallographic data for this paper.^80*a*–*k*^ In addition, all underlying NMR and UV/Vis data supporting this work are openly available *via* the TU Graz repository at: https://doi.org/10.3217/wqzvf-n9j76.
