# Supplementary material for: Clicking acylgermanes: modular access to photoinitiators for visible-light photopolymerization
Source: Org Chem Front. 2026 Jul 15;13(18):6126–38. doi: 10.1039/d6qo00741d (PMC13439046; doi:10.1039/d6qo00741d)
Supplement: Supplementary file 1 [file QO-013-D6QO00741D-s001.pdf]

Supplementary Information (SI) for Organic Chemistry Frontiers.
This journal is © the Partner Organisations 2026

Supporting Information

Clicking Acylgermanes: Modular Access to Photoinitiators for Visible-Light Photopolymerization

André Culum,[a,b] Julia Fuchs,[c,d] Carolyn Vargas,[e] Fabian Rath,[a] Dagmar Brislinger,[c] Thomas Lainer,[a,f] Julian Maier,[g] Roland C. Fischer,[a] Ana Torvisco,[a] Anne-Marie Kelterer,[h] Dmytro Neshchadin,[h] Thomas Griesser,[g] Sandro Keller,[e] Tanja M. Wrodnigg[b] and Michael Haas*[a,f]

[a] Andre Culum, MSc, Fabian Rath, BSc, Dr. Thomas Lainer, Dr. Ana Torvisco, Prof. Roland C. Fischer, Prof. Michael Haas
Institute of Inorganic Chemistry, Graz University of Technology
Stremayrgasse 9/IV, 8010 Graz (Austria)
E-mail: michael.haas@tugraz.at

[b] Andre Culum, MSc, Prof. Tanja M. Wrodnigg
Institute of Chemistry and Technology of Biobased Systems
Stremayrgasse 9/II, 8010 Graz (Austria)

[c] Dr. Dagmar Brislinger MSc, Dr. Julia Fuchs, MSc MSc
Medical University of Graz, Division of Cell Biology, Histology and Embryology
Neue Stiftingtalstraße 6, 8010 Graz (Austria)

[d] Dr. Julia Fuchs, MSc MSc
Institute of Biomechanics, Graz University of Technology
Stremayrgasse 16/II; 8010 Graz (Austria)

[e] Dr. Carolyn Vargas, Prof. Sandro Keller
Biophysics, Institute of Molecular Biosciences (IMB), University of Graz, NAWI Graz, Field of Excellence BioHealth; BioTechMed-Graz
Humboldtstr. 50/III, 8010 Graz (Austria)

[f] Dr. Thomas Lainer, Prof. Michael Haas
Christian Doppler Laboratory for New Semiconductor Materials based on Functionalized Hydrosilanes
Stremayrgasse 9/IV, 8010 Graz (Austria).

[g] Julian Maier, MSc, Prof. Thomas Griesser
Chemistry of Polymeric Materials, Technical University of Leoben
Otto-Glöckel-Strasse 2, 8700 Leoben (Austria)

[h] Dr. Dmytro Neschchadin, Prof. Anne-Marie Kelterer
Institute of Physical and Theoretical Chemistry, Graz University of Technology
Stremayrgasse 9/II, 8010 Graz (Austria)

Table of contents

Experimental section **5**

General considerations 5

General procedure for copper catalyzed azide-alkyne Huisgen cycloadditions (Click reactions) 5

Prop-2-yn-1-yltris(2,4,6-trimethylbenzoyl)germane (1) 6

Tris(2,4,6-trimethylbenzoyl)(4-ethynylbenzoyl)germane (2) 6

Tris(2,4,6-trimethylbenzoyl)(hex-5-ynoyl)germane (3) 7

Tris(2,4,6-trimethylbenzoyl)(4-azidobenzoyl)germane (4) 7

Tris(2,4,6-trimethylbenzoyl)(4-(1-phenyl-1,2,3-triazol-4-yl)benzoyl)germane (5) 8

Tris(2,4,6-trimethylbenzoyl)(1-(1-benzyl-1,2,3-triazol-4-yl)methyl)germane (6) 8

Tris(2,4,6-trimethylbenzoyl)(4-(1-benzyl-1,2,3-triazol-4-yl)butanoyl)germane (7) 8

Tris(2,4,6-trimethylbenzoyl)(4-(1-(4-methylbenzyl)-1,2,3-triazol-4-yl)benzoyl)germane (8) 9

Tris(2,4,6-trimethylbenzoyl)(4-(1-(2-nitrobenzyl)-1,2,3-triazol-4-yl)benzoyl)germane (9) 9

Tris(2,4,6-trimethylbenzoyl)(4-(1-(4-(dimethylamino)benzyl)-1,2,3-triazol-4-yl)benzoyl)germane (10) 10

Tris(2,4,6-trimethylbenzoyl)(4-(1-(6-(methoxycarbonyl)hexyl)-1,2,3-triazol-4-yl)benzoyl)germane (11) 10

Tris(2,4,6-trimethylbenzoyl)(1-(1-(6-(methoxycarbonyl)hexyl)-1,2,3-triazol-4-yl)methyl)germane (12) 11

Tris(2,4,6-trimethylbenzoyl)(4-(1-(6-(methoxycarbonyl)hexyl)-1,2,3-triazol-4-yl)butanoyl)germane (13) 11

Tris(2,4,6-trimethylbenzoyl)(4-(1-phenyl-1,2,3-triazol-4-yl)benzoyl)germane (14) 12

Tris(2,4,6-trimethylbenzoyl)(4-(1-(4-carboxyphenyl)-1,2,3-triazol-4-yl)benzoyl)germane (15) 12

Tris(2,4,6-trimethylbenzoyl)(4-(1-(4-(methoxycarbonyl)phenyl)-1,2,3-triazol-4-yl)benzoyl)germane (16) 13

Tris(2,4,6-trimethylbenzoyl)(4-(1-(6-deoxy-6-(methyl-α-D-glucopyranosyl)-1,2,3-triazol-4-yl)benzoyl)germane (17) 13

Tris(2,4,6-trimethylbenzoyl)(1-(6-deoxy-6-(methyl-α-D-glucopyranosyl)-1,2,3-triazol-4-yl)methyl)germane (18) 14

Tris(2,4,6-trimethylbenzoyl)(4-(1-(6-deoxy-6-(methyl-α-D-glucopyranosyl)-1,2,3-triazol-4-yl)butanoyl)germane (19) 14

Tris(2,4,6-trimethylbenzoyl)(4-(1-phenyl-1,2,3-triazol-4-yl)benzoyl)germane (20) 15

Tris(2,4,6-trimethylbenzoyl)(4-(4-(4-carboxyphenyl)-1,2,3-triazol-1-yl)benzoyl)germane (21) 15

Tris(2,4,6-trimethylbenzoyl)(4-(4-(4-(methoxycarbonyl)phenyl)-1,2,3-triazol-1-yl)benzoyl)germane (22) 16

Tris(2,4,6-trimethylbenzoyl)(4-(4-(4-(pinacolboronyl)phenyl)-1,2,3-triazol-1-yl)benzoyl)germane (23) 16

Tris(2,4,6-trimethylbenzoyl)(4-(4-(tris(2,4,6-trimethylbenzoyl)germanyl)-1,2,3-triazol-1-yl)benzoyl)germane (24) 17
Tris(2,4,6-trimethylbenzoyl)((1-(4-(tris(2,4,6-trimethylbenzoyl)germanyl)benzoyl)-1,2,3-triazol-4-yl)methyl)germane (25) 17
Tris(2,4,6-trimethylbenzoyl)((1-(4-(tris(2,4,6-trimethylbenzoyl)germanyl)benzoyl)-1,2,3-triazol-4-yl)butanoyl)germane (26) 18
Single Crystal X-ray Crystallography 19
Cytotoxicity 27
Cell culture 27
Cytotoxicity assay 27
Isothermal titration calorimetry (ITC) 28
NMR Spectroscopy 30
Prop-2-yn-1-yltris(2,4,6-trimethylbenzoyl)germane (1) 30
Tris(2,4,6-trimethylbenzoyl)(4-ethynylbenzoyl)germane (2) 31
Tris(2,4,6-trimethylbenzoyl)(hex-5-ynoyl)germane (3) 32
Tris(2,4,6-trimethylbenzoyl)(4-azidobenzoyl)germane (4) 33
Tris(2,4,6-trimethylbenzoyl)(4-(1-phenyl-1,2,3-triazol-4-yl)benzoyl)germane (5) 34
Tris(2,4,6-trimethylbenzoyl)(1-(1-benzyl-1,2,3-triazol-4-yl)methyl)germane (6) 35
Tris(2,4,6-trimethylbenzoyl)(4-(1-benzyl-1,2,3-triazol-4-yl)butanoyl)germane (7) 36
Tris(2,4,6-trimethylbenzoyl)(4-(1-(4-methylbenzyl)-1,2,3-triazol-4-yl)benzoyl)germane (8) 37
Tris(2,4,6-trimethylbenzoyl)(4-(1-(2-nitrobenzyl)-1,2,3-triazol-4-yl)benzoyl)germane (9) 38
Tris(2,4,6-trimethylbenzoyl)(4-(1-(4-(dimethylamino)benzyl)-1,2,3-triazol-4-yl)benzoyl)germane (10) 39
Tris(2,4,6-trimethylbenzoyl)(4-(1-(6-(methoxycarbonyl)hexyl)-1,2,3-triazol-4-yl)benzoyl)germane (11) 40
Tris(2,4,6-trimethylbenzoyl)(1-(1-(6-(methoxycarbonyl)hexyl)-1,2,3-triazol-4-yl)methyl)germane (12) 41
Tris(2,4,6-trimethylbenzoyl)(4-(1-(6-(methoxycarbonyl)hexyl)-1,2,3-triazol-4-yl)butanoyl)germane (13) 42
Tris(2,4,6-trimethylbenzoyl)(4-(1-phenyl-1,2,3-triazol-4-yl)benzoyl)germane (14) 43
Tris(2,4,6-trimethylbenzoyl)(4-(1-(4-carboxyphenyl)-1,2,3-triazol-4-yl)benzoyl)germane (15) 44
Tris(2,4,6-trimethylbenzoyl)(4-(1-(4-(methoxycarbonyl)phenyl)-1,2,3-triazol-4-yl)benzoyl)germane (16) 46
Tris(2,4,6-trimethylbenzoyl)(4-(1-(6-deoxy-6-(methyl-α-D-glucopyranosyl)-1,2,3-triazol-4-yl)benzoyl)germane (17) 47
Tris(2,4,6-trimethylbenzoyl)(1-(6-deoxy-6-(methyl-α-D-glucopyranosyl)-1,2,3-triazol-4-yl)methyl)germane (18) 48
Tris(2,4,6-trimethylbenzoyl)(4-(1-(6-deoxy-6-(methyl-α-D-glucopyranosyl)-1,2,3-triazol-4-yl)butanoyl)germane (19) 49

Tris(2,4,6-trimethylbenzoyl)(4-(1-phenyl-1,2,3-triazol-4-yl)benzoyl)germane (20) 50
Tris(2,4,6-trimethylbenzoyl)(4-(4-(4-carboxyphenyl)-1,2,3-triazol-1-yl)benzoyl)germane (21) 51
Tris(2,4,6-trimethylbenzoyl)(4-(4-(4-(methoxycarbonyl)phenyl)-1,2,3-triazol-1-yl)benzoyl)germane (22) 52
Tris(2,4,6-trimethylbenzoyl)(4-(4-(4-(pinacolboronyl)phenyl)-1,2,3-triazol-1-yl)benzoyl)germane (23) 53
Tris(2,4,6-trimethylbenzoyl)(4-(4-(tris(2,4,6-trimethylbenzoyl)germanyl)-1,2,3-triazol-1-yl)benzoyl)germane (24) 54
Tris(2,4,6-trimethylbenzoyl)((1-(4-(tris(2,4,6-trimethylbenzoyl)germanyl)benzoyl)-1,2,3-triazol-4-yl)methyl)germane (25) 55
Tris(2,4,6-trimethylbenzoyl)((1-(4-(tris(2,4,6-trimethylbenzoyl)germanyl)benzoyl)-1,2,3-triazol-4-yl)butanoyl)germane (26) 56

UV-Vis Spectroscopy 57

Photo-DSC 70

Photo-CIDNP 71

DFT Calculations 74

Computational Methods 74

Experimental section

General considerations

All experiments were performed under a nitrogen atmosphere using standard Schlenk techniques. Solvents were dried using a column solvent purification system. KO*t*Bu (>98%), C_6D_6 (99.5 atom%, D) and $CDCl_3$ (99.8 atom% D) were used without any further purification. For the measurement of air sensitive samples deuterated solvents were additionally dried (C_6D_6 was dried by 24-hour reflux above a sodium/potassium alloy). Tetrakis(trimethylsilyl)germane and FCO(Mes) was prepared according to published procedures.

^{1}H- and ^{13}C-NMR spectra were recorded on a Bruker Ultrashield 300 or a 400 MHz JEOL JNM ECZL spectrometer with a Royal HFX-Probes with an autosampler. Samples were prepared as in C_6D_6 or $CDCl_3$ solutions and referenced versus TMS using the internal ^{2}H-lock signal of the solvent.

Infrared spectra were obtained on a Bruker Alpha-P Diamond ATR Spectrometer from the solid sample. Melting points were determined using Stuart SMP50 apparatus and are uncorrected. Elemental analyses were carried out on a Hanau Vario Elementar EL apparatus. UV absorption spectra were recorded on a Perkin Elmer Lambda 5 spectrometer.

General procedure for copper catalyzed azide-alkyne Huisgen cycloadditions (Click reactions)

The germanium alkyne or azide was (1.0 eq.) was dissolved in THF along with the respective azide or alkyne reactant (1.2 eq). To this CuI (5 mol%) and Et_3N (0.2 eq) were added and the reaction mixture was stirred for 16 h. Starting material conversion was confirmed via TLC. The reaction mixture was diluted with DCM and extracted with a saturated NH_4Cl solution, the organic layer was dried with Na_2SO_4 and the solvent removed *in vacuo*. The crude reaction mixture was purified via column chromatography (eluents are indicated in the respective experimental procedure), yielding the desired product.

Prop-2-yn-1-yltris(2,4,6-trimethylbenzoyl)germane (1)

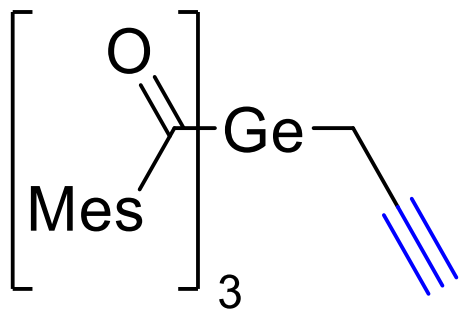

Potassium trimesitoylgermenolate KGe[(CO)Mes]$_3$ (1.0 g, 1.45 mmol, 1.0 eq.) was dissolved in 10 mL THF and stirred with $MgBr_2$ (0.15 g, 0.80 mmol, 0.55 eq.) for 5 min at ambient temperature. This solution was subsequently added dropwise to 10 mL of a cooled (-50 °C) 0.3 M solution of propargyl bromide in THF (1.90 mmol, 2.0 eq.). The reaction mixture was stirred for 1 h after which it was brought to room temperature. TLC analysis (pentane/toluene, 1/2, *v*/*v*) confirmed complete conversion of the starting material. The reaction mixture was diluted with 50 ml diethyl ether and washed with a saturated NH_4Cl solution, the organic layer was dried with Na_2SO_4 and the solvent removed in vacuum. The crude reaction mixture was purified via column chromatography (pentane/toluene, 2/1, v/v). Yield: 630 mg (1.13 mmol; 78%) of analytically pure **1** as a crystalline yellow powder. **M.p**.: 122-124 °C. **^{1}H-NMR Data** (400 MHz, $CDCl_3$, ppm): 6.68 (s, 6H, Mes-*H*), 2.26 (s, 9H, Mes-C*H*$_3$), 2.13 (d, 2H, *J* = 2.9 Hz, Ge-C*H*$_2$), 2.10 (s, 18H, Mes-C*H*$_3$), 1.77 (t, 1H, *J* = 2.9 Hz, propargyl-C*H*). **^{13}C-NMR Data** (101 MHz, $CDCl_3$, ppm): 233.65 (Mes-*C*=O), 141.24 (Mes-*C4*), 139.71 (Mes-*C1*), 132.90 (Mes-*C2*), 128.88 (Mes-*C3*), 80.02 (propargyl-*C*^{q}), 69.51 (propargyl-*C*H), 21.25, 19.17 (Mes-*C*H$_3$), 4.85 (Ge-*C*H$_2$). **UV-vis**: λ [nm] (ε [L mol^{-1} cm^{-1}]) = 356 (928), 381 (1561), 400 (1136). **MS (ESI-TOF)**: Calculated for [$C_{33}H_{36}GeO_3$]: m/z [M+H]$^+$ 555.1954; found [M+H]$^+$ 555.1985.

Tris(2,4,6-trimethylbenzoyl)(4-ethynylbenzoyl)germane (2)

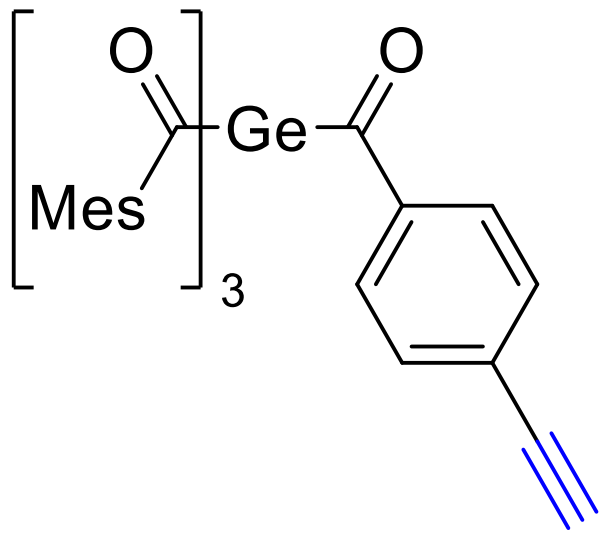

Potassium trimesitoylgermenolate KGe[(CO)Mes]$_3$ (1.0 g, 1.45 mmol, 1.0 eq.) was dissolved in 10 mL THF and stirred with $MgBr_2$ (0,15 g, 0.80 mmol, 0.55 eq.) for 5 min at ambient temperature. This solution was subsequently added dropwise to 10 mL of a cooled (-50 °C) 0.25 g solution of 4-ethynylbenzoyl chloride in THF (1.52 mmol, 1.05 eq.). The reaction mixture was stirred for 1 h after which it was brought to room temperature. TLC analysis (pentane/toluene, 1/2, *v*/*v*) confirmed complete conversion of the starting material. The reaction mixture was diluted with 50 ml diethyl ether and washed with a saturated NH_4Cl solution, the organic layer was dried with Na_2SO_4 and the solvent removed in vacuum. The crude reaction mixture was purified via column chromatography (pentane/toluene, 2/1, *v/v*). Yield: 774 mg (1.20 mmol; 83%) of analytically pure **2** as a crystalline yellow powder. **M.p**.: 182-184 °C. **^{1}H-NMR Data** (400 MHz, $CDCl_3$, ppm): 7.63 (d, 2H, *J* = 8.4 Hz, aromatic-C*H*), 7.35 (d, 2H, *J* = 8.4 Hz, aromatic-C*H*), 6.57 (s, 6H, Mes-*H*), 3.25 (s, 1H, alkyne-*H*), 2.20 (s, 9H, Mes-C*H*$_3$), 2.12 (s, 18H, Mes-C*H*$_3$). **^{13}C-NMR Data** (101 MHz, $CDCl_3$, ppm): 232.33 (Mes-*C*=O), 220.86 (Mes-*C*=O), 141.34 (Mes-*C4*), 140.05 (Mes-*C1*), 139.39 (aromatic-*C1*), 133.07 (Mes-*C2*), 132.08 (aromatic-*C2*), 128.80 (Mes-*C3*), 128.75 (aromatic-*C3*), 127.05 (aromatic-*C4*), 82.82 (alkyne-*C*^{q}), 80.90 (alkyne-*C*H), 21.22, 19.32 (Mes-*C*H$_3$). **UV-vis**: λ [nm] (ε [L mol^{-1} cm^{-1}]) = 378 (1930), 402 (1482), 453 (385). **MS (ESI-TOF)**: Calculated for [$C_{39}H_{38}GeO_4$]: m/z [M+H]$^+$ 645.2060; found [M+H]$^+$ 645.2074.

Tris(2,4,6-trimethylbenzoyl)(hex-5-ynoyl)germane (3)

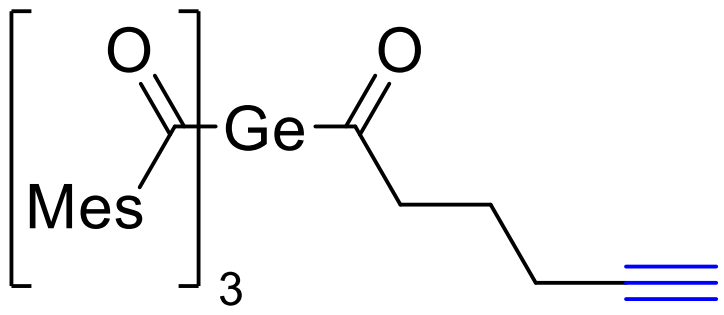

Potassium trimesitoylgermenolate KGe[(CO)Mes]$_3$ (1.0 g, 1.45 mmol, 1.0 eq.) was dissolved in 10 mL THF and stirred with $MgBr_2$ (0,15 g, 0.80 mmol, 0.55 eq.) for 5 min at ambient temperature. This solution was subsequently added dropwise to 10 mL of a cooled (-50 °C) 0.15 g solution of hex-5-ynoyl chloride in THF (1.52 mmol, 1.05 eq.). The reaction mixture was stirred for 1 h after which it was brought to room temperature. TLC analysis (pentane/toluene, 1/2, *v/v*) confirmed complete conversion of the starting material. The reaction mixture was diluted with 50 ml diethyl ether and washed with a saturated NH_4Cl solution, the organic layer was dried with Na_2SO_4 and the solvent removed in vacuum. The crude reaction mixture was purified via column chromatography (pentane/toluene, 2/1, *v/v*). Yield: 627 mg (1.03 mmol; 71%) of analytically pure **3** as a crystalline yellow powder. **M.p.**: 95-96 °C. **^{1}H-NMR Data** (400 MHz, $CDCl_3$, ppm): 6.96 (s, 6H, Mes-*H*), 2.60 (t, 2H, *J* = 7.0 Hz, C*H*$_2$-2), 2.24 (s, 9H, Mes-C*H*$_3$), 2.11 (s, 18H, Mes-C*H*$_3$), 1.97 (dt, 2H, *J* = 7.0, 2.6 Hz, C*H*$_2$-4), 1.97 (t, 1H, *J* = 2.6 Hz, alkyne-*H*), 1.56 (tt, 2H, *J* = 7.0, 7.0 Hz, C*H*$_2$-3). **^{13}C-NMR Data** (101 MHz, $CDCl_3$, ppm): 232.09 (Mes-*C*=O), 229.79 (Mes-*C*=O), 141.69 (Mes-*C4*), 139.88 (Mes-*C1*), 132.75 (Mes-*C2*), 128.89 (Mes-*C3*), 83.36 (alkyne-*C*^{q}), 68.96 (alkyne-*C*H), 49,94 (*C*H$_2$-2), 21.24, 19.12 (Mes-*C*H$_3$), 21.20 (*C*H$_2$-4), 17.61 (*C*H$_2$-3). **UV-vis**: λ [nm] (ε [L mol^{-1} cm^{-1}]) = 262 (2090), 380 (1876), 403 (1265). **MS (ESI-TOF)**: Calculated for [$C_{36}H_{40}GeO_4$]: m/z [M+H]$^+$ 611.2216; found [M+H]$^+$ 611.2279.

Tris(2,4,6-trimethylbenzoyl)(4-azidobenzoyl)germane (4)

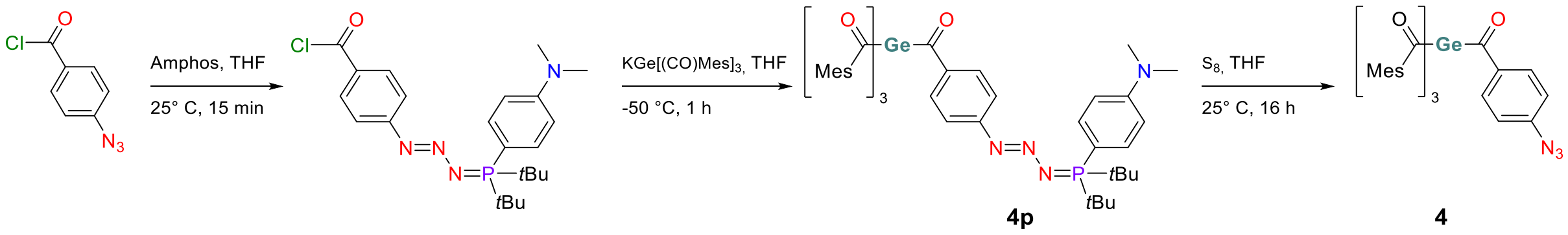

Scheme S1. A detailed overview of each reaction step required for the preparation of **4** via the protected azide **4p**.

4-azidobenzoyl chloride (276 mg, 1.52 mmol, 1.05 eq.) was dissolved in 10 mL THF and di-tert-butyl(4-dimethylaminophenyl)phosphine, also known as Amphos, (423 mg, 1.60 mmol, 1.1 eq.) was added in portions over the course of 5 min. It is important to add the phosphine reagent in small portions, otherwise the formation of an uncharacterized precipitate is formed and the reaction cannot proceed. After stirring the reaction mixture at r.t. for 10 min, potassium trimesitoylgermenolate KGe[(CO)Mes]$_3$ (1.0 g, 1.45 mmol, 1.0 eq.) dissolved in 20 mL THF was added dropwise at -50 °C. The reaction was allowed to warm to room temperature, diluted with 50 mL of diethyl ether, washed with a saturated NH_4Cl solution, the organic layer was dried with $MgSO_4$ and the solvent removed in vacuum. The product is air and water stable, however signs of degradation at elevated temperatures can be observed *via* TLC, thus all rotary evaporation steps should be conducted at 30 °C. The crude, protected compound **4p** (Scheme S1), a dark red oil, was dissolved in 30 mL of THF and a large stochiometric excess of S_8 (1.8 g, 7.25 mmol, 5.0 eq.) was added. The reaction was stirred at r.t. overnight, TLC (pentane/toluene, 1/2, *v/v*) indicated complete deprotection of the azide functionality. The excess S_8 was filtered of and the reaction mixture was concentrated under reduced pressure. Purification via column chromatography (pentane/toluene, 2/1, *v/v*) yielded the desired azide **4** as a fine yellow powder. Yield: 627 mg (1.03 mmol; 71%). **Anal. Calc.** (%) for $C_{37}H_{37}GeN_3O_4$: C, 67.30; H, 5.65; N, 6.36; Found: C, 67.12; H, 5.71; N, 6.29. **M.p.**:198-200 °C. **^{1}H-NMR Data** (400 MHz, $CDCl_3$, ppm): 7.71 (d, 2H, *J* = 8.7 Hz, aromatic-C*H*), 6.87 (d, 2H, *J* = 8.7 Hz, aromatic-C*H*), 6.57 (s, 6H, Mes-*H*), 2.20 (s, 9H, Mes-C*H*$_3$), 2.11 (s, 18H, Mes-C*H*$_3$). **^{13}C-NMR Data** (101 MHz, $CDCl_3$, ppm): 232.63 (Mes-*C*=O), 219.04 (aromatic-*C*=O), 145.35 (aromatic-*C1*), 141.39 (Mes-*C4*), 139.92 (Mes-*C1*), 136.82 (aromatic-*C4*), 133.14 (Mes-*C2*), 131.16 (aromatic-*C2*), 128.83 (Mes-*C3*), 118.87 (aromatic-*C3*), 21.23, 19.36 (Mes-*C*H$_3$). **UV-vis**: λ [nm] (ε [L mol^{-1} cm^{-1}]) = 369 (1631), 379 (1668), 420 (1036), 445 (454). **MS (ESI-TOF)**: Calculated for [$C_{37}H_{37}GeN_3O_4$]: m/z [M+H]$^+$ 662.2074; no [M+H]$^+$ found.

We were unsuccessful in obtaining analytically pure NMR Data of protected compound **4p**, however crystals suitable for XRD were obtained from crystallisation from diethyl ether at -30 °C for 3 months. The crystal structure (Figure S5) matches the proposed structure of **4p**. Due to the instability of the compound further characterisation was not performed.

Tris(2,4,6-trimethylbenzoyl)(4-(1-phenyl-1,2,3-triazol-4-yl)benzoyl)germane (5)

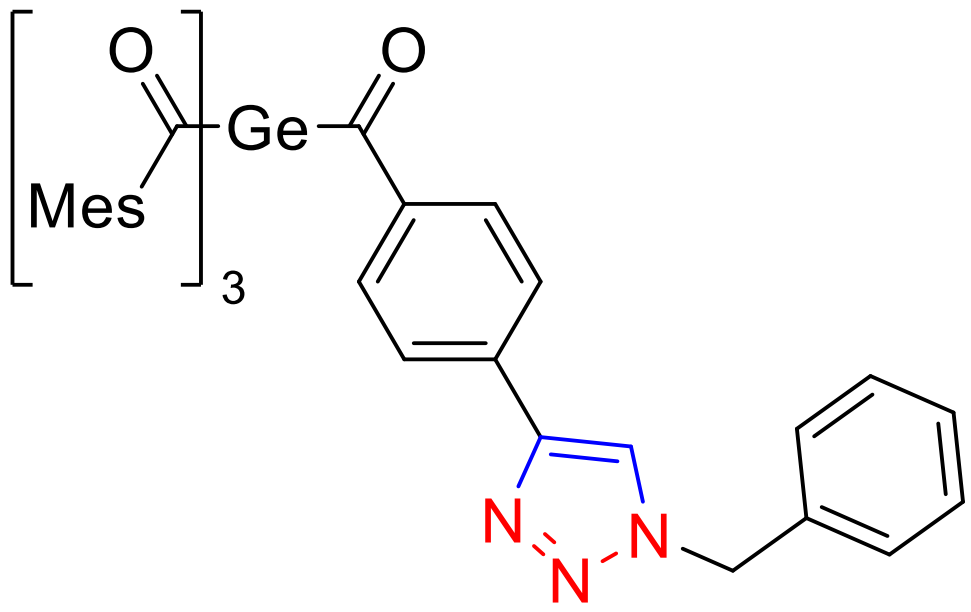

Following the general procedure for click reactions, 200 mg (0.31 mmol, 1.0 eq.) of compound **2** and 50 mg (0.37 mmol, 1.2 eq.) of benzyl azide were reacted in 10 mL THF in the presence of 3 mg CuI (5 mol%) and 10 µL (0.06 mmol, 0.2 eq) Et_3N. The reaction mixture was purified via column chromatography (cyclohexane/ethyl acetate, 10/1, *v/v*) yielding a yellow oil. Yield: 190 mg (0.24 mmol, 79%). **^{1}H-NMR Data** (400 MHz, $CDCl_3$, ppm): 7.70 (m, 5H, aromatic-C*H*), 7.40 (m, 3H, aromatic-C*H*, triazole-C*H*), 7.30 (m, 2H, aromatic-C*H*), 6.56 (s, 6H, Mes-*H*), 5.59 (s, 2H, benzylic-C*H*$_2$), 2.13 (s, 9H, Mes-C*H*$_3$), 2.12 (s, 18H, Mes-C*H*$_3$). **^{13}C-NMR Data** (101 MHz, $CDCl_3$, ppm): 232.64 (Mes-*C*=O), 220.32 (aromatic-*C*=O), 146.97 (triazole-*C*^{q}), 141.40 (Mes-*C4*), 139.86 (Mes-*C1*), 139.26, 135.18, 134.51 (aromatic-*C*), 133.06 (Mes-*C2*), 129.76, 129.33, 129.05 (aromatic-*C*), 128.77 (Mes-*C3*), 128.17, 125.41 (aromatic-*C*), 120.61 (triazole-*C*H), 54.45 (benzylic-*C*H$_2$), 21.16, 19.30 (Mes-*C*H$_3$). **UV-vis**: λ [nm] (ε [L mol^{-1} cm^{-1}]) = 374 (2288), 415 (1353), 446 (561). **MS (ESI-TOF)**: Calculated for [$C_{46}H_{45}GeN_3O_4$]: m/z [M+H]$^+$ 778.2700; found [M+H]$^+$ 778.2740.

Tris(2,4,6-trimethylbenzoyl)(1-(1-benzyl-1,2,3-triazol-4-yl)methyl)germane (6)

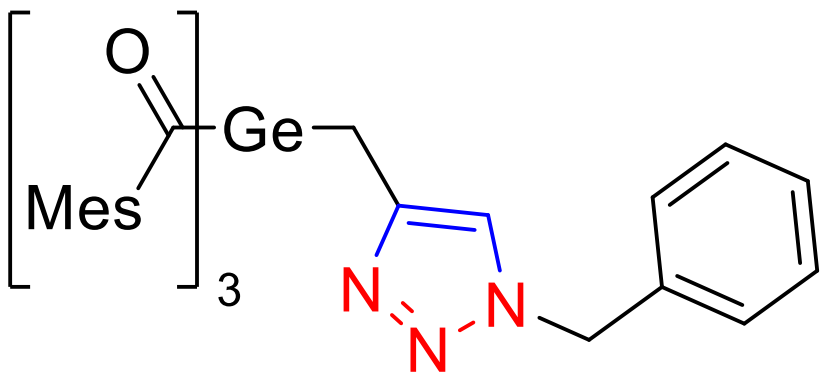

Following the general procedure for click reactions, 200 mg (0.36 mmol, 1.0 eq.) of compound **1** and 58 mg (0.43 mmol, 1.2 eq.) of benzyl azide were reacted in 10 mL THF in the presence of 4 mg CuI (5 mol%) and 11 µl (0.07 mmol, 0.2 eq) Et_3N. The reaction mixture was purified via column chromatography (cyclohexane/ethyl acetate, 10/1, *v/v*) yielding a yellow oil. Yield: 184 mg (0.27 mmol, 74 %). **^{1}H-NMR Data** (400 MHz, $CDCl_3$, ppm): 7.36 (m, 3H, aromatic-C*H*), 7.23 (m, 2H, aromatic-C*H*), 6.64 (s, 1H, triazole-C*H),* 6.61 (s, 6H, Mes-*H*), 5.32 (s, 2H, benzylic-C*H*$_2$), 2.82 (s, 2H, Ge-C*H*$_2$), 2.23 (s, 9H, Mes-C*H*$_3$), 1.99 (s, 18H, Mes-C*H*$_3$). **^{13}C-NMR Data** (101 MHz, $CDCl_3$, ppm): 234.96 (Mes-*C*=O), 143.20 (triazole-*C*^{q}), 141.59 (Mes-*C4*), 139.40 (Mes-*C1*), 134.98 (aromatic-*C*), 132.89 (Mes-*C2*), 129.11 (aromatic-*C*), 128.77 (Mes-*C3*), 128.67, 128.09 (aromatic-*C*), 121.16 (triazole-*C*H), 53.98 (benzylic-*C*H$_2$), 21.22, 19.13 (Mes-*C*H$_3$), 13.75 (Ge-*C*H$_2$). **UV-vis**: λ [nm] (ε [L mol^{-1} cm^{-1}]) = 355 (800), 370 (1261), 380 (1414), 401 (987). **MS (ESI-TOF)**: Calculated for [$C_{40}H_{43}GeN_3O_3$]: m/z [M+H]$^+$ 688.2594; found [M+H]$^+$ 688.2697.

Tris(2,4,6-trimethylbenzoyl)(4-(1-benzyl-1,2,3-triazol-4-yl)butanoyl)germane (7)

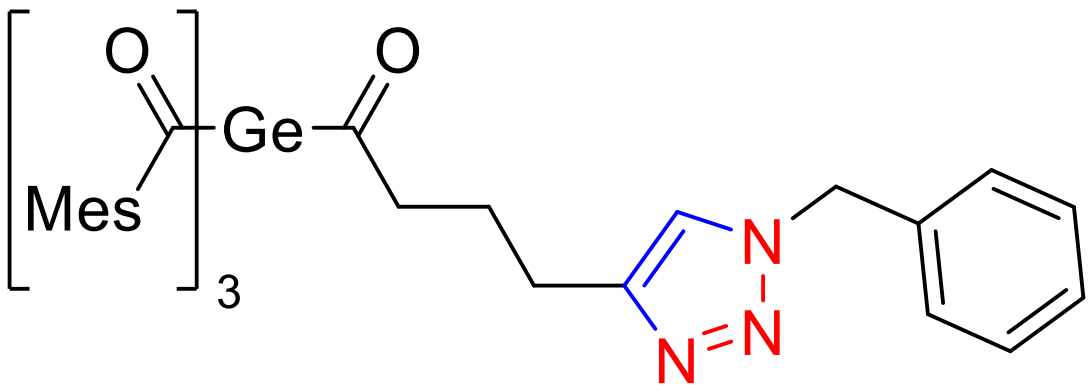

Following the general procedure for click reactions, 200 mg (0.33 mmol, 1.0 eq.) of compound **3** and 52 mg (0.39 mmol, 1.2 eq.) of benzyl azide were reacted in 10 mL THF in the presence of 4 mg CuI (5 mol%) and 11 µl (0.07 mmol, 0.2 eq) Et_3N. The reaction mixture was purified via column chromatography (cyclohexane/ethyl acetate, 10/1, *v/v*) yielding a yellow oil. Yield: 175 mg (0.24 mmol, 72 %). **Anal. Calc.** (%) for $C_{43}H_{47}GeN_3O_4$: C, 69.56; H, 6.38; N, 5.66.; Found: C, 69.44; H, 6.41; N, 5.63. **^{1}H-NMR Data** (400 MHz, $CDCl_3$, ppm): 7.38 (m, 3H, aromatic-C*H*), 7.27 (m, 2H, aromatic-C*H*), 7.18 (s, 1H, triazole-C*H),* 6.67 (s, 6H, Mes-*H*), 5.50 (s, 2H, benzylic-C*H*$_2$), 2.53 (t, 2H,

J = 6.9 Hz, aliphatic-CH_2-1), 2.45 (t, 2H, *J* = 7.8 Hz, aliphatic-CH_2-3), 2.24 (s, 9H, Mes-CH_3), 2.09 (s, 18H, Mes-CH_3), 2.45 (tt, 2H, *J* = 7.8 7.8 Hz, aliphatic-CH_2-2). **^{13}C-NMR Data** (101 MHz, $CDCl_3$, ppm): 232.19 (Mes-*C*=O), 229.97 (aliphatic-C=O), 147.89, (triazole-C^q), 141.72 (Mes-*C4*), 139.95 (Mes-*C1*), 134.92 (aromatic-*C*), 132.77 (Mes-*C2*), 129.21 (aromatic-*C*), 128.91 (Mes-*C3*), 128.80, 128.18 (aromatic-*C*), 120.73 (triazole-*C*H), 54.19 (benzylic-CH_2), 50.66 (aliphatic-*C*1), 24.88 (aliphatic-*C*2), 21.97 (aliphatic-*C*2), 21.27, 19.15 (Mes-*C*H_3). **UV-vis**: λ [nm] (ε [L mol^{-1} cm^{-1}]) = 359 (1693), 379 (1458), 403 (947).

Tris(2,4,6-trimethylbenzoyl)(4-(1-(4-methylbenzyl)-1,2,3-triazol-4-yl)benzoyl)germane (8)

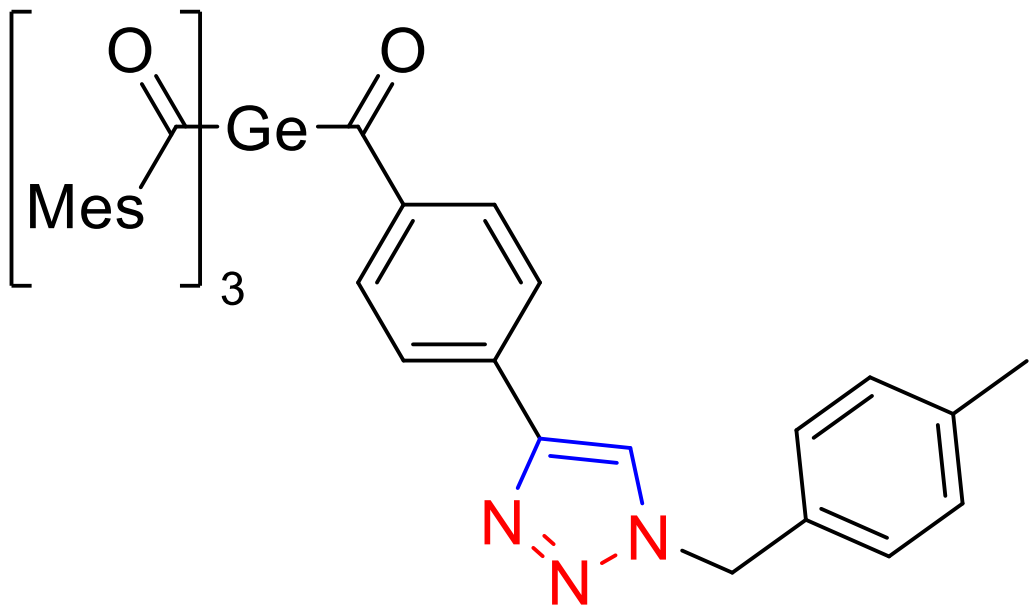

Following the general procedure for click reactions, 200 mg (0.31 mmol, 1.0 eq.) of compound **2** and 55 mg (0.37 mmol, 1.2 eq.) of 4-methylbenzyl azide were reacted in 10 mL THF in the presence of 3 mg CuI (5 mol%) and 10 µl (0.06 mmol, 0.2 eq) Et_3N. The reaction mixture was purified via column chromatography (cyclohexane/ethyl acetate, 10/1, *v/v*) yielding a yellow oil. Yield: 204 mg (0.26 mmol, 83 %). **^{1}H-NMR Data** (400 MHz, $CDCl_3$, ppm): 7.69 (m, 5H, aromatic-C*H*, triazole-C*H*), 7.21 (s, 4H, aromatic-C*H*), 6.56 (s, 6H, Mes-*H*), 5.54 (s, 2H, benzylic-CH_2), 2.36 (s, 3H, aromatic-CH_3), 2.13 (s, 9H, Mes-CH_3), 2.12 (s, 18H, Mes-CH_3). **^{13}C-NMR Data** (101 MHz, $CDCl_3$, ppm): 232.65 (Mes-*C*=O), 220.30 (aromatic-*C*=O), 146.87 (triazole-C^q), 141.40 (Mes-*C4*), 139.86 (Mes-*C1*), 139.23, 139.04, 135.24 (aromatic-*C*), 133.06 (Mes-*C2*), 131.45, 129.98, 129.75 (aromatic-*C*), 128.77 (Mes-*C3*), 128.25, 125.40 (aromatic-*C*), 120.53 (triazole-*C*H), 54.26 (benzylic-*C*H_2), 27.00 (aromatic-*C*H_3), 21.16, 19.30 (Mes-*C*H_3). **UV-vis**: λ [nm] (ε [L mol^{-1} cm^{-1}]) = 378 (1956), 401 (1417), 439 (561). **MS (ESI-TOF)**: Calculated for [$C_{47}H_{47}GeN_3O_4$]: m/z $[M+H]^+$ 792.2856; found $[M+H]^+$ 792.2886.

Tris(2,4,6-trimethylbenzoyl)(4-(1-(2-nitrobenzyl)-1,2,3-triazol-4-yl)benzoyl)germane (9)

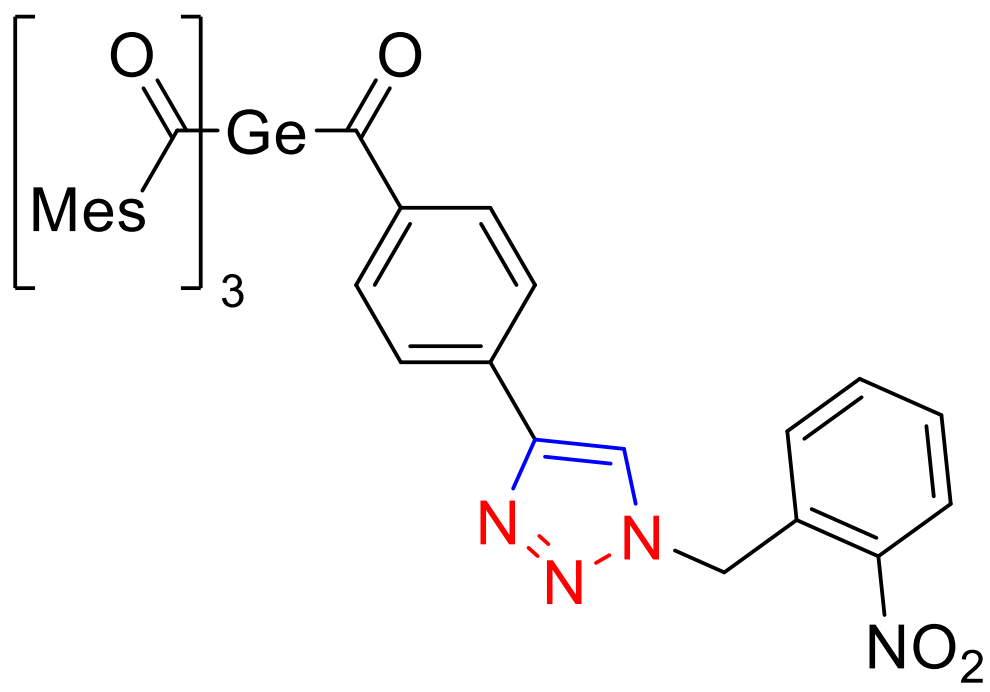

Following the general procedure for click reactions, 200 mg (0.31 mmol, 1.0 eq.) of compound **2** and 66 mg (0.37 mmol, 1.2 eq.) of 2-nitrobenzyl azide were reacted in 10 mL THF in the presence of 3 mg CuI (5 mol%) and 10 µl (0.06 mmol, 0.2 eq) Et_3N. The reaction mixture was purified via column chromatography (cyclohexane/ethyl acetate, 10/1, *v/v*) yielding a yellow oil. Yield: 204 mg (0.25 mmol, 80 %). **^{1}H-NMR Data** (400 MHz, $CDCl_3$, ppm): 8.16 (dd, 1H, *J* = 8.1, 1.4 Hz, aromatic-*H*), 8.01 (s, 1H, triazole-C*H*), 7.73 (s, 4H, aromatic-*H*), 7.64 (td, *J* = 7.6, 1.4 Hz, 1H, aromatic-*H*), 7.55 (td, *J* = 7.6, 1.4 Hz, 1H, aromatic-*H*), 7.21 (dd, *J* = 7.7, 1.5 Hz, 1H, aromatic-*H*), 6.56 (s, 6H, Mes-*H*), 6.00 (s, 2H, benzylic-CH_2), 2.14 (s, 9H, Mes-CH_3), 2.12 (s, 18H, Mes-CH_3). **^{13}C-NMR Data** (101 MHz, $CDCl_3$, ppm): 232.62 (Mes-*C*=O), 220.42 (aromatic-*C*=O), 147.65 (triazole-C^q), 147.06 (aromatic-*C*), 141.42 (Mes-*C4*), 139.91 (Mes-*C1*), 139.44, 134.92, 134.57 (aromatic-*C*), 133.10 (Mes-*C2*), 130.82, 130.39, 130.07, 129.84 (aromatic-*C*), 128.81 (Mes-*C3*), 125.61, 125.55 (aromatic-*C*), 121.75 (triazole-*C*H), 51.15 (benzylic-*C*H_2), 21.20, 19.34 (Mes-*C*H_3). **UV-vis**: λ [nm] (ε [L mol^{-1} cm^{-1}]) = 370 (1715), 420 (905), 444 (439). **MS (ESI-TOF)**: Calculated for [$C_{46}H_{44}GeN_4O_6$]: m/z $[M+H]^+$ 823.2551; found $[M+H]^+$ 823.2573.

Tris(2,4,6-trimethylbenzoyl)(4-(1-(4-(dimethylamino)benzyl)-1,2,3-triazol-4-yl)benzoyl)germane (10)

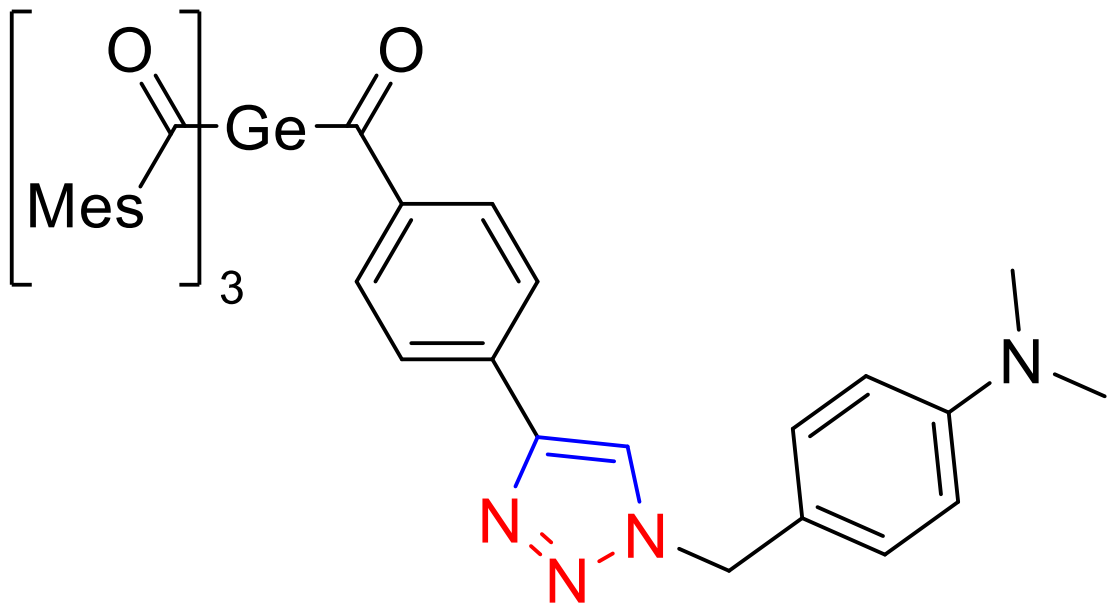

Following the general procedure for click reactions, 100 mg (0.16 mmol, 1.0 eq.) of compound **2** and 33 mg (0.19 mmol, 1.2 eq.) of 4-(azidomethyl)-*N*,*N*-dimethylbenzenamine were reacted in 5 mL THF in the presence of 2 mg CuI (5 mol%) and 5 µl (0.03 mmol, 0.2 eq) Et_3N. The reaction mixture was purified via column chromatography (cyclohexane/ethyl acetate, 10/1, *v/v*) yielding a yellow oil. Yield: 68 mg (0.08 mmol, 53 %). **Anal. Calc.** (%) for $C_{48}H_{50}GeN_4O_4$: C, 70.34; H, 6.15; N, 6.84. Found: C, 70.12; H, 6.21; N, 6.77. **^{1}H-NMR Data** (400 MHz, $CDCl_3$, ppm): 7.69 (m, 5H, triazole-C*H*, aromatic-C*H*), 7.22 (m, 2H, aromatic-C*H*), 6.75 (m, 2H, aromatic-C*H*), 6.56 (s, 6H, Mes-*H*), 5.48 (s, 2H, benzylic-CH_2), 2.98 (s, 6H, amine-CH_3), 2.13 (s, 9H, Mes-CH_3), 2.11 (s, 18H, Mes-CH_3). **^{13}C-NMR Data** (101 MHz, $CDCl_3$, ppm): 232.71(Mes-*C*=O), 220.30(aromatic-*C*=O), 146.68 (triazole-C^q), 141.45 (Mes-*C4*), 139.89 (Mes-*C1*), 139.18, 135.94, 135.41, 133.33 (aromatic-*C*), 133.10 (Mes-*C2*), 130.79, 130.36, 130.03, 129.81 (aromatic-*C*), 133.06 (Mes-*C3*), 129.77, 129.73 (aromatic-*C*), 128.81 (aromatic-C), 120.40 (triazole-*C*H), 54.31 (benzylic-*C*H$_2$), 40.59 (amine *C*H$_3$), 21.11, 19.34 (Mes-*C*H$_3$). **UV-vis**: λ [nm] (ε [L mol^{-1} cm^{-1}]) = 367 (1717), 379 (1685), 419 (1009), 441 (540).

Tris(2,4,6-trimethylbenzoyl)(4-(1-(6-(methoxycarbonyl)hexyl)-1,2,3-triazol-4-yl)benzoyl)germane (11)

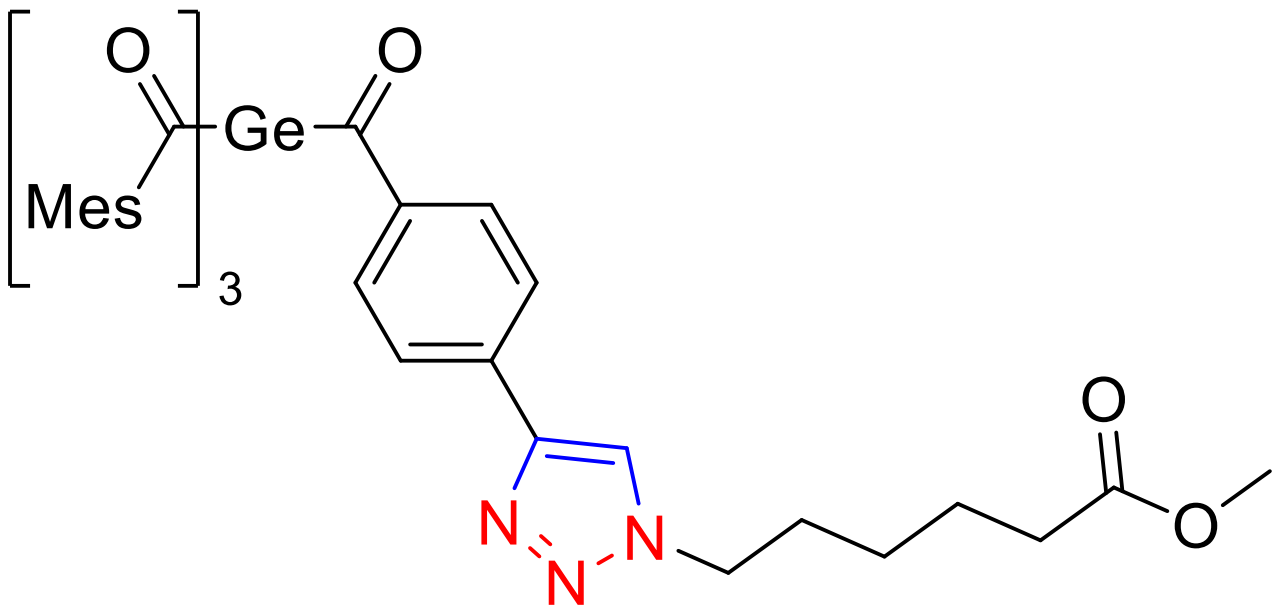

Following the general procedure for click reactions, 200 mg (0.31 mmol, 1.0 eq.) of compound **2** and 64 mg (0.37 mmol, 1.2 eq.) of methyl 6-azidohexanoate were reacted in 10 mL THF in the presence of 3 mg CuI (5 mol%) and 10 µl (0.06 mmol, 0.2 eq) Et_3N. The reaction mixture was purified via column chromatography (cyclohexane/ethyl acetate, 10/1, *v/v*) yielding a yellow oil. Yield: 212 mg (0.26 mmol, 84 %). **^{1}H-NMR Data** (400 MHz, $CDCl_3$, ppm): 7.83 (s, 1H, triazole-C*H*), 7.73 (s, 4H, aromatic-C*H*), 6.57 (s, 6H, Mes-*H*), 4.43 (t, 2H, *J* = 7.1 Hz, aliphatic-CH_2-5) 3.66 (s, 3H, ester-CH_3), 2.33 (t, 2H, *J* = 7.3 Hz, aliphatic-CH_2-1), 2.14 (s, 9H, Mes-CH_3), 2.12 (s, 18H, Mes-CH_3), 1.99 (tt, 2H, *J* = 7.2, 7.2 Hz, aliphatic-CH_2-4), 1.70 (tt, 2H, *J* = 7.4, 7.4 Hz, aliphatic-CH_2-2), 1.40 (m, 2H, aliphatic-CH_2-3). **^{13}C-NMR Data** (101 MHz, $CDCl_3$, ppm): 232.69 (Mes-*C*=O), 220.35 (aromatic-*C*=O), 173.92 (ester-*C*=O), 146.58 (triazole-C^q), 141.47 (Mes-*C4*), 139.92 (Mes-*C1*), 139.30, 135.36 (aromatic-*C*), 133.14 (Mes-*C2*), 129.87 (aromatic-*C*), 128.83 (Mes-*C3*), 125.46 (aromatic-*C*), 120.70 (triazole-*C*H), 51.75 (ester-*C*H$_3$), 50.34 (aliphatic-*C*-5), 33.74 (aliphatic-*C*-1), 30.13 (aliphatic-*C*-4), 26.03 (aliphatic-*C*-2), 24.26 (aliphatic-*C*-3), 21.23, 19.37 (Mes-*C*H$_3$). **UV-vis**: λ [nm] (ε [L mol^{-1} cm^{-1}]) = 371 (1047), 381 (1022), 419 (577), 440 (327). **MS (ESI-TOF)**: Calculated for [$C_{46}H_{51}GeN_3O_6$]: m/z $[M+H]^+$ 816.3068; found $[M+H]^+$ 816.3084.

Tris(2,4,6-trimethylbenzoyl)(1-(1-(6-(methoxycarbonyl)hexyl)-1,2,3-triazol-4-yl)methyl)germane (12)

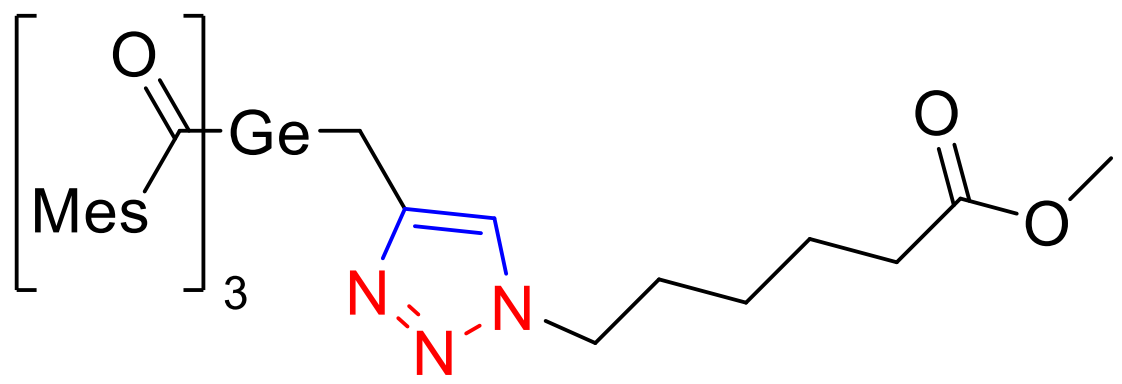

Following the general procedure for click reactions, 200 mg (0.36 mmol, 1.0 eq.) of compound **1** and 74 mg (0.43 mmol, 1.2 eq.) of methyl 6-azidohexanoate were reacted in 10 mL THF in the presence of 4 mg CuI (5 mol%) and 11 µl (0.07 mmol, 0.2 eq) Et_3N. The reaction mixture was purified via column chromatography (cyclohexane/ethyl acetate, 10/1, *v/v*) yielding a yellow oil. Yield: 227 mg (0.32 mmol, 87 %). **^{1}H-NMR Data** (400 MHz, $CDCl_3$, ppm): 6.79 (s, 1H, triazole-C*H*), 6.64 (s, 6H, Mes-*H*), 4.13 (t, 2H, *J* = 7.2 Hz, aliphatic-C*H*$_2$-5) 3.64 (s, 3H, ester-C*H*$_3$), 2.84 (s, 2H, Ge-C*H*$_2$); 2.27 (t, 2H, *J* = 7.4 Hz, aliphatic-C*H*$_2$-1), 2.23 (s, 9H, Mes-C*H*$_3$), 2.02 (s, 18H, Mes-C*H*$_3$), 1.79 (tt, 2H, *J* = 7.4, 7.4 Hz, aliphatic-C*H*$_2$-4), 1.63 (tt, 2H, *J* = 7.4, 7.4 Hz, aliphatic-C*H*$_2$-2), 1.29 (m, 2H, aliphatic-C*H*$_2$-3). **^{13}C-NMR Data** (101 MHz, $CDCl_3$, ppm): 235.09 (Mes-*C*=O), 173.86 (ester-*C*=O), 142.67 (triazole-*C*^{q}), 141.61 (Mes-*C4*), 139.44 (Mes-*C1*), 132.92 (Mes-*C2*), 128.80 (Mes-*C3*), 121.20 (triazole-*C*H), 51.63 (ester-*C*H$_3$), 49.80 (aliphatic-*C*-5), 33.75 (aliphatic-*C*-1), 30.05 (aliphatic-*C*-4), 26.02 (aliphatic-*C*-2), 24.34 (aliphatic-*C*-3), 21.22, 19.15 (Mes-*C*H$_3$), 13.69(Ge-*C*H$_2$). **UV-vis**: λ [nm] (ε [L mol^{-1} cm^{-1}]) = 357 (862), 371 (1302), 381 (1507), 402 (1051). **MS (ESI-TOF)**: Calculated for [$C_{40}H_{49}GeN_3O_5$]: m/z [M+H]$^+$ 726.2962; found [M+H]$^+$ 726.3017.

Tris(2,4,6-trimethylbenzoyl)(4-(1-(6-(methoxycarbonyl)hexyl)-1,2,3-triazol-4-yl)butanoyl)germane (13)

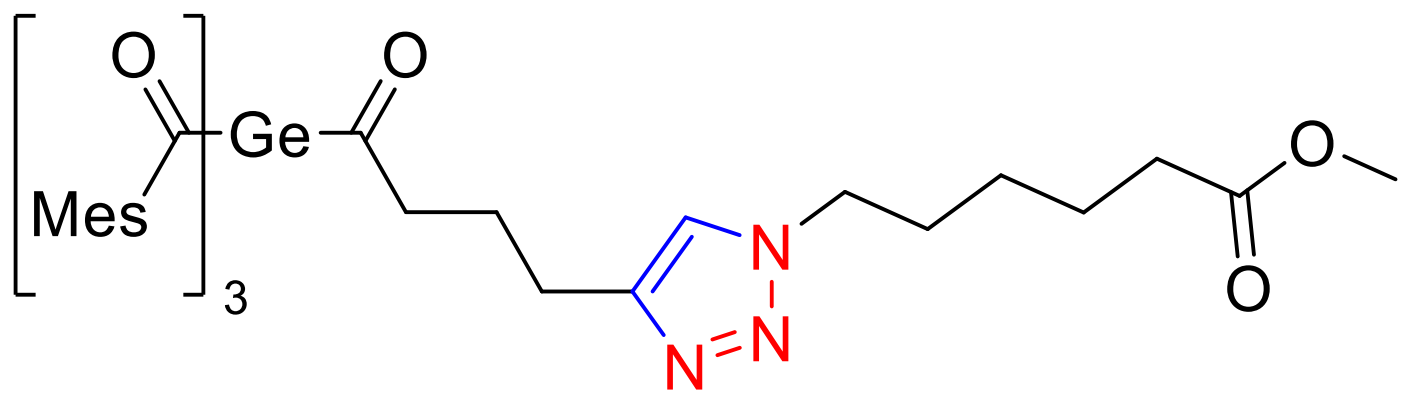

Following the general procedure for click reactions, 200 mg (0.33 mmol, 1.0 eq.) of compound **3** and 67 mg (0.39 mmol, 1.2 eq.) of compound methyl 6-azidohexanoate were reacted in 10 mL THF in the presence of 4 mg CuI (5 mol%) and 11 µl (0.07 mmol, 0.2 eq) Et_3N. The reaction mixture was purified via column chromatography (cyclohexane/ethyl acetate, 10/1, *v/v*) yielding a yellow oil. Yield: 209 mg (0.27 mmol, 81 %). **^{1}H-NMR Data** (400 MHz, $CDCl_3$, ppm): 6.66 (s, 1H, triazole-C*H*), 6.64 (s, 6H, Mes-*H*), 4.30 (t, 2H, *J* = 7.2 Hz, aliphatic-C*H*$_2$-5), 3.65 (s, 3H, ester-C*H*$_3$), 2.53 (t, 2H, *J* = 6.9 Hz, aliphatic-C*H*$_2$-1), 2.45 (t, 2H, *J* = 7.8 Hz, aliphatic-C*H*$_2$-3), 2.29 (t, 2H, *J* = 7.3 Hz, aliphatic-C*H*$_2$-1'), 2.23 (s, 9H, Mes-C*H*$_3$), 2.08 (s, 18H, Mes-C*H*$_3$), 1.99 (tt, 2H, *J* = 7.4, 7.4 Hz, aliphatic-C*H*$_2$-4'), 1.69 (m, 2H, aliphatic-C*H*$_2$-2), 1.64 (m, 2H, aliphatic-C*H*$_2$-2'), 1.33 (m, 2H, aliphatic-C*H*$_2$-3'). **^{13}C-NMR Data** (101 MHz, $CDCl_3$, ppm): 232.22 (Mes-*C*=O), 229.94 (aliphatic-*C*=O), 173.91 (ester-*C*=O), 147.44 (triazole-*C*^{q}), 141.72 (Mes-*C4*), 139.96 (Mes-*C1*), 132.77 (Mes-*C2*), 128.92 (Mes-*C3*), 120.68 (triazole-*C*H), 51.68 (ester-*C*H$_3$), 50.68 (aliphatic-*C*-1), 50.04 (aliphatic-*C*-5'), 33.77 (aliphatic-*C*-1'), 30.16 (aliphatic-*C*-4'), 26.10 (aliphatic-*C*-3'), 24.87 (aliphatic-*C*-3), 24.34 (aliphatic-*C*-2'), 22.08 (aliphatic-*C*-2), 21.28, 19.16 (Mes-*C*H$_3$). **UV-vis**: λ [nm] (ε [L mol^{-1} cm^{-1}]) = 364 (1058), 381 (938), 404 (635). **MS (ESI-TOF)**: Calculated for [$C_{43}H_{53}GeN_3O_6$]: m/z [M+H]$^+$ 782.3224; found [M+H]$^+$ 782.3279.

Tris(2,4,6-trimethylbenzoyl)(4-(1-phenyl-1,2,3-triazol-4-yl)benzoyl)germane (14)

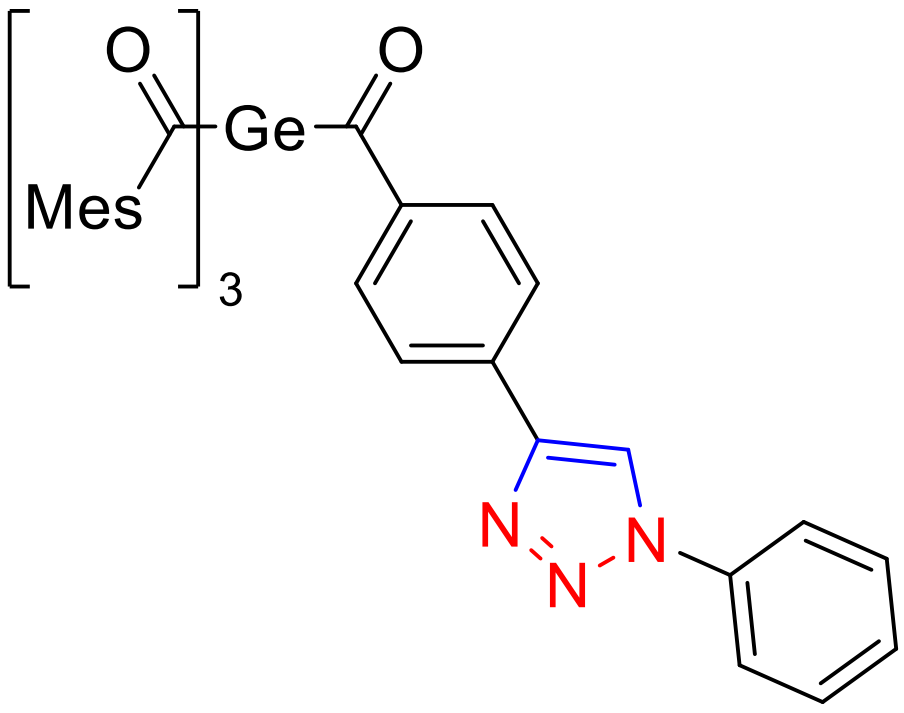

Following the general procedure for click reactions, 200 mg (0.31 mmol, 1.0 eq.) of compound **2** and 0.75 mL (0.37 mmol, 1.2 eq.) of a 0.5 M phenylazide solution (in tert-butyl methyl ether) were dissolved in 10 mL THF. The reaction was initiated by the addition of 3 mg CuI (5 mol%) and 10 µl (0.06 mmol, 0.2 eq) Et_3N. The reaction mixture was purified via column chromatography (cyclohexane/ethyl acetate, 10/1, *v/v*) yielding a yellow crystalline solid. Yield: 217 mg (0.29 mmol, 92 %). **M.p.**: 141-142 °C. **^{1}H-NMR Data** (400 MHz, $CDCl_3$, ppm): 8.28 (s, 1H, triazole-C*H*), 7.79 (m, 6H, aromatic-C*H*), 7.56 (t, 1H, *J* = 7.7 Hz, aromatic-C*H*), 7.48 (t, 1H, *J* = 7.4 Hz, aromatic-C*H*), 6.58 (s, 6H, Mes-*H*), 2.15 (s, 9H, Mes-CH_3), 2.14 (s, 18H, Mes-CH_3). **^{13}C-NMR Data** (101 MHz, $CDCl_3$, ppm): 232.62 (Mes-*C*=O), 220.39 (aromatic-*C*=O), 147.17 (triazole-C^q), 141.42 (Mes-*C4*), 139.94 (Mes-*C1*), 139.46, 136.91, 134.91 (aromatic-*C*), 133.10 (Mes-*C2*), 130.01, 129.87, 129.19 (aromatic-*C*), 128.82 (Mes-*C3*), 125.60, 120.63 (aromatic-*C*), 118.71 (triazole-*C*H), 21.22, 19.35 (Mes-*C*H3). **UV-vis**: λ [nm] (ε [L mol^{-1} cm^{-1}]) = 378 (1553), 394 (1231), 382 (1242), 420 (793). **MS (ESI-TOF)**: Calculated for [$C_{45}H_{43}GeN_3O_4$]: m/z $[M+H]^+$ 764.2543; found $[M+H]^+$ 764.2575.

Tris(2,4,6-trimethylbenzoyl)(4-(1-(4-carboxyphenyl)-1,2,3-triazol-4-yl)benzoyl)germane (15)

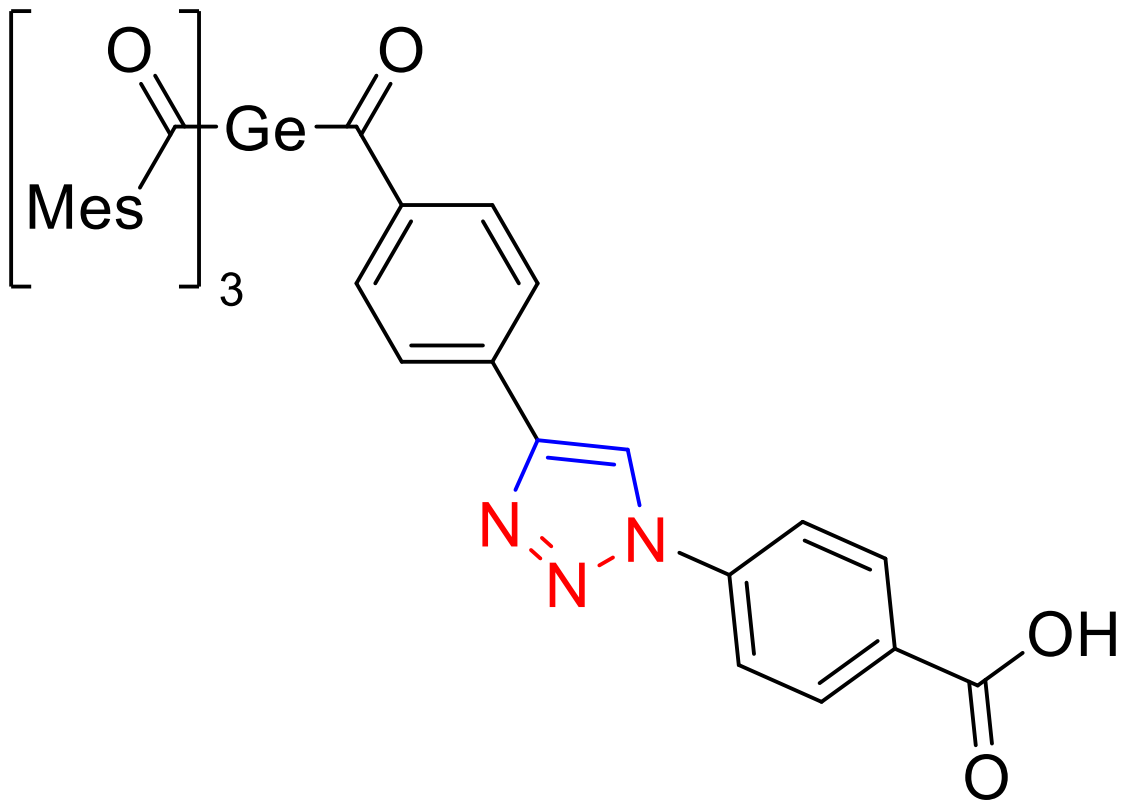

Following the general procedure for click reactions, 200 mg (0.31 mmol, 1.0 eq.) of compound **2** and 60 mg (0.37 mmol, 1.2 eq.) of 4-azidobenzoic acid were reacted in 10 mL THF in the presence of 3 mg CuI (5 mol%) and 110 µl (0.78 mmol, 2.5 eq) Et_3N. The reaction mixture was purified via column chromatography (cyclohexane/ethyl acetate, 1/2, *v/v*) yielding a yellow crystalline solid. Yield: 173 mg (0.21 mmol, 69 %). **Anal. Calc.** (%) for $C_{46}H_{43}GeN_3O_6$: C, 68.51; H, 5.37; N, 5.21. Found: C, 68.33; H, 5.44; N, 5.18. **M.p.**: 161-163 °C. **^{1}H-NMR Data** (400 MHz, $CDCl_3$, ppm): 8.35 (s, 1H, triazole-C*H*), 8.34 (m, 2H, aromatic-C*H*), 7.98 (m, 2H, aromatic-C*H*), 7.81 (m, 4H, aromatic-C*H*), 6.58 (s, 6H, Mes-*H*), 2.16 (s, 9H, Mes-CH_3), 2.14 (s, 18H, Mes-CH_3). **^{13}C-NMR Data** (101 MHz, $CDCl_3$, ppm): 232.53 (Mes-*C*=O), 220.58 (aromatic-*C*=O), 169.74 (ester-*C*=O), 147.74 (triazole-C^q), 141.47 (Mes-*C4*), 139.76 (Mes-*C1*), 136.20, 134.42 (aromatic-*C*), 133.17 (Mes-*C2*), 132.31, 129.95, 128.92 (aromatic-*C*), 128.87 (Mes-*C3*), 125.77, 120.15 (aromatic-*C*), 118.36 (triazole-*C*H), 21.28, 19.40 (Mes-*C*H3). **UV-vis**: λ [nm] (ε [L mol^{-1} cm^{-1}]) = 368 (1657), 379 (1592), 429 (717).

Tris(2,4,6-trimethylbenzoyl)(4-(1-(4-(methoxycarbonyl)phenyl)-1,2,3-triazol-4-yl)benzoyl)germane (16)

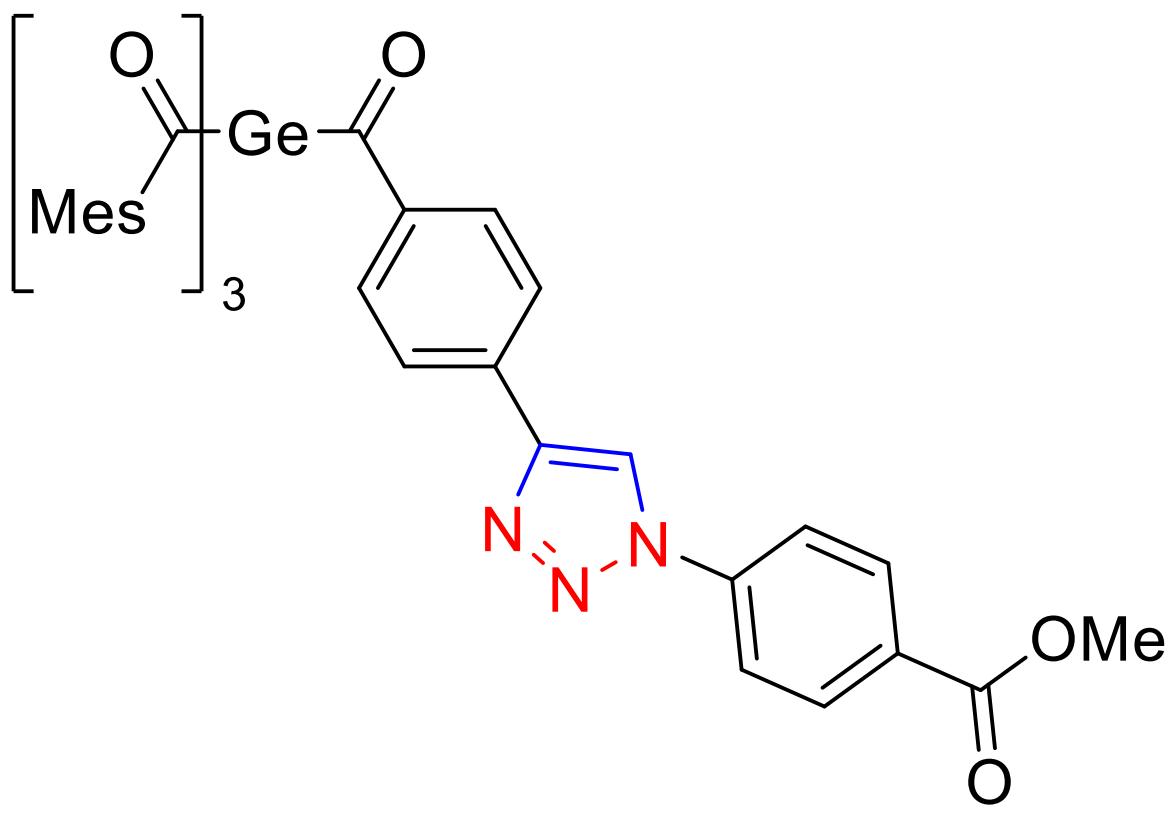

Following the general procedure for click reactions, 200 mg (0.31 mmol, 1.0 eq.) of compound **2** and 66 mg (0.37 mmol, 1.2 eq.) of 4-azidobenzoic acid methyl ester were reacted in 10 mL THF in the presence of 3 mg CuI (5 mol%) and 10 µl (0.06 mmol, 0.2 eq) Et_3N. The reaction mixture was purified via column chromatography (cyclohexane/ethyl acetate, 10/1, *v/v*) yielding a crystalline yellow solid. Yield: 188 mg (0.23 mmol, 74 %). **Anal. Calc.** (%) for $C_{47}H_{45}GeN_3O_6$: C, 68.80; H, 5.53; N, 5.12. Found: C, 68.61; H, 5.60; N, 5.08. **M.p.**: 127-129 °C. **^{1}H-NMR Data** (400 MHz, $CDCl_3$, ppm): 8.36 (s, 1H, triazole-C*H*), 8.22 (m, 2H, aromatic-C*H*), 7.90 (m, 2H, aromatic-C*H*), 7.78 (m, 4H, aromatic-C*H*), 6.56 (s, 6H, Mes-*H*), 3.96 (s, 3H, ester-C*H*$_3$), 2.14 (s, 9H, Mes-C*H*$_3$), 2.12 (s, 18H, Mes-C*H*$_3$). **^{13}C-NMR Data** (101 MHz, $CDCl_3$, ppm): 232.55 (Mes-*C*=O), 220.47 (aromatic-*C*=O), 165.94 (ester-*C*=O), 147.55 (triazole-*C*^{q}), 141.41 (Mes-*C4*), 139.91 (Mes-*C1*), 139.63, 134.50 (aromatic-*C*), 133.09 (Mes-*C2*), 131.56, 130.60, 129.88 (aromatic-*C*), 128.82 (Mes-*C3*), 128.78, 125.70, 120.00 (aromatic-*C*), 118.46 (triazole-*C*H), 52.63 (ester-*C*H$_3$), 21.22, 19.35 (Mes-*C*H$_3$). **UV-vis**: λ [nm] (ε [L mol^{-1} cm^{-1}]) = 376 (1619), 420 (564), 441 (478).

Tris(2,4,6-trimethylbenzoyl)(4-(1-(6-deoxy-6-(methyl-α-D-glucopyranosyl)-1,2,3-triazol-4-yl)benzoyl)germane (17)

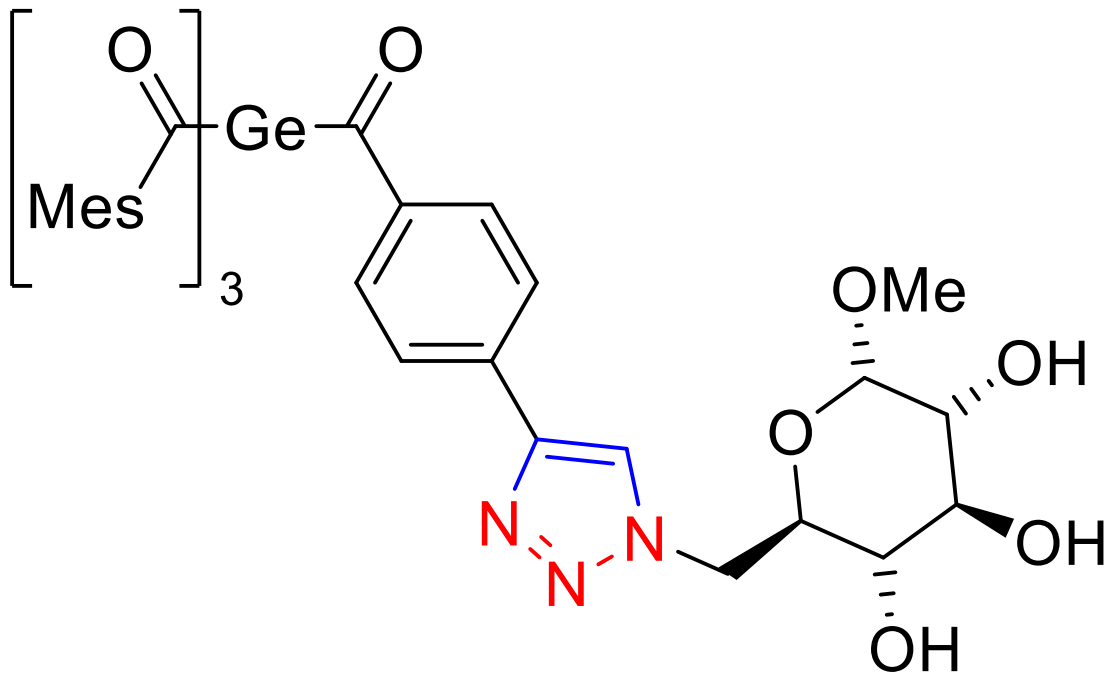

Following the general procedure for click reactions, 200 mg (0.31 mmol, 1.0 eq.) of compound **2** and 81 mg (0.37 mmol, 1.2 eq.) of methyl 6-azido-6-deoxy-α-D-glucopyranoside were reacted in 10 mL THF in the presence of 3 mg CuI (5 mol%) and 10 µl (0.06 mmol, 0.2 eq) Et_3N. The reaction mixture was purified via column chromatography (ethyl acetate/methanol, 10/1, *v/v*) yielding a yellow oil. Yield: 211 mg (0.24 mmol, 79 %). **^{1}H-NMR Data** (400 MHz, MeOD, ppm): 8.47 (s, 1H, triazole-C*H*), 7.74 (d, 2H, *J* = 8.5 Hz, aromatic-C*H*), 7.67 (d, 2H, *J* = 8.5 Hz, aromatic-C*H*), 6.58 (s, 6H, Mes-*H*), 4.89 (m, 1H, C*H*$_2$-6a), 4.65 (d, 1H, *J* = 3.7 Hz, C*H*-1), 4.59 (dd, 1H, *J* = 14.2, 7.9 Hz, C*H*-6b), 4.59 (ddd, 1H, *J* = 10.3, 7.9, 2.6 Hz, C*H*-5), 3.64 (dd, 1H, *J* = 9.0, 8.8 Hz, C*H*-3), 3.37 (dd, 1H, *J* = 9.7, 3.7 Hz, C*H*-2), 3.16 (s, 3H, OMe), 3.15 (m, 1H, C*H*-4), 2.13 (s, 9H, Mes-C*H*$_3$), 2.09 (s, 18H, Mes-C*H*$_3$). **^{13}C-NMR Data** (101 MHz, MeOD, ppm): 233.00 (Mes-*C*=O), 219.79 (aromatic-*C*=O), 145.74 (triazole-*C*^{q}), 141.36 (Mes-*C4*), 140.00 (Mes-*C1*), 139.04, 135.35 (aromatic-*C*), 132.64 (Mes-*C2*), 129.46 (aromatic-*C*), 128.52 (Mes-*C3*), 124.95 (aromatic-*C*), 123.76 (triazole-*C*H), 99.98 (*C*1), 73.60 (*C*3), 72.03 (*C*2), 71.67 (*C*4), 70.31 (*C*5), 54.23 (OMe), 51.31 (*C*6), 19.96, 18.26 (Mes-*C*H$_3$). **UV-vis**: λ [nm] (ε [L mol^{-1} cm^{-1}]) = 370 (1762), 386 (1754), 419 (1014), 440 (558). **MS (ESI-TOF)**: Calculated for [$C_{46}H_{51}GeN_3O_9$]: m/z [M+H]$^+$ 864.2915; found [M+H]$^+$ 864.2934.

Tris(2,4,6-trimethylbenzoyl)(1-(6-deoxy-6-(methyl-α-D-glucopyranosyl)-1,2,3-triazol-4-yl)methyl)germane (18)

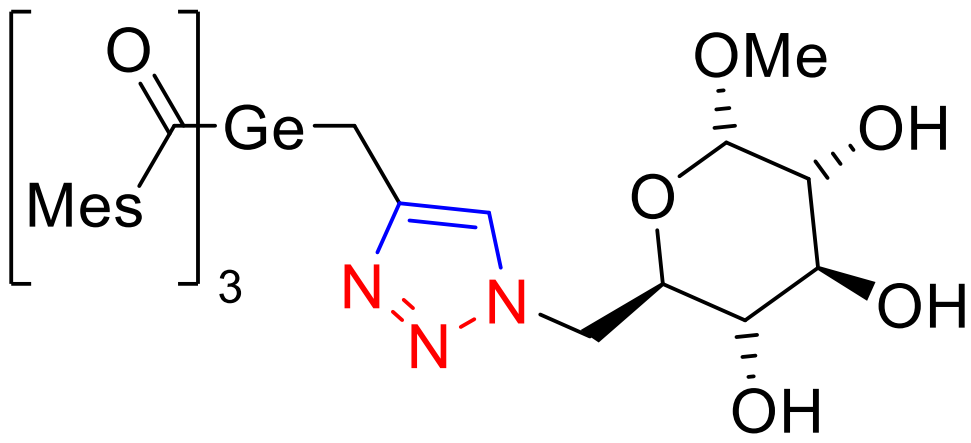

Following the general procedure for click reactions, 200 mg (0.36 mmol, 1.0 eq.) of compound **1** and 94 mg (0.43 mmol, 1.2 eq.) of methyl 6-azido-6-deoxy-α-D-glucopyranoside were reacted in 10 mL THF in the presence of 4 mg CuI (5 mol%) and 11 µl (0.07 mmol, 0.2 eq) Et_3N. The reaction mixture was purified via column chromatography (ethyl acetate/methanol, 10/1, *v/v*) yielding a yellow oil. Yield: 203 mg (0.26 mmol, 73 %). **^{1}H-NMR Data** (400 MHz, MeOD, ppm): 7.05 (s, 1H, triazole-C*H*), 6.69 (s, 6H, Mes-*H*), 4.61 (d, 1H, *J* = 3.8 Hz, C*H*-1), 4.58 (m, 1H, CH_2-6a), 4.32 (dd, 1H, *J* = 14.3, 7.7 Hz, C*H*-6b), 3.78 (ddd, 1H, *J* = 10.0, 7.6, 2.3 Hz, C*H*-5), 3.60 (dd, 1H, *J* = 9.2, 9.2 Hz, C*H*-3), 3.35 (dd, 1H, *J* = 9.8, 3.7 Hz, C*H*-2), 3.16 (s, 3H, OMe), 3.07 (dd, 1H, *J* = 9.4, 9.4 Hz, C*H*-4), 2.80 (s, 2H, Ge-CH_2), 2.25 (s, 9H, Mes-CH_3), 2.00 (s, 18H, Mes-CH_3). **^{13}C-NMR Data** (101 MHz, MeOD, ppm): 235.03 (Mes-*C*=O), 142.16 (triazole-C^q), 141.51 (Mes-*C4*), 139.67 (Mes-*C1*), 132.58 (Mes-*C2*), 128.55 (Mes-*C3*), 123.33 (triazole-*C*H), 99.99 (*C*1), 73.52 (*C*3), 71.94 (*C*2), 71.44 (*C*4), 70.28 (*C*5), 54.53 (OMe), 50.87 (*C*6), 19.97, 18.21 (Mes-CH_3), 13.10 (Ge-CH_2). **UV-vis**: λ [nm] (ε [L mol^{-1} cm^{-1}]) = 355 (784), 370 (1212), 381 (1423), 402 (981). **MS (ESI-TOF)**: Calculated for [$C_{40}H_{49}GeN_3O_8$]: m/z [M+H]$^+$ 774.2809; found [M+H]$^+$ 774.2849.

Tris(2,4,6-trimethylbenzoyl)(4-(1-(6-deoxy-6-(methyl-α-D-glucopyranosyl)-1,2,3-triazol-4-yl)butanoyl)germane (19)

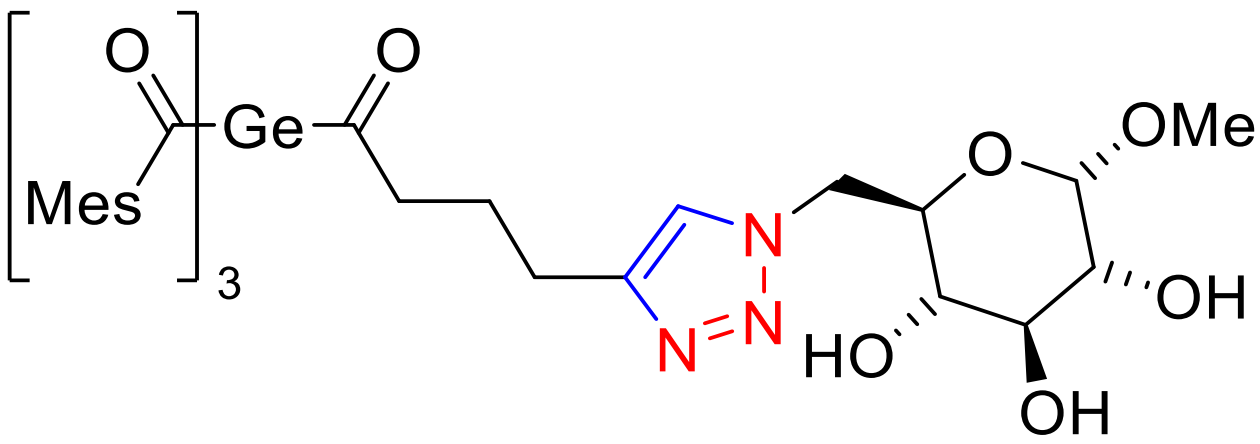

Following the general procedure for click reactions, 200 mg (0.33 mmol, 1.0 eq.) of compound **3** and 86 mg (0.39 mmol, 1.2 eq.) of methyl 6-azido-6-deoxy-α-D-glucopyranoside were reacted in 10 mL THF in the presence of 4 mg CuI (5 mol%) and 11 µl (0.07 mmol, 0.2 eq) Et_3N. The reaction mixture was purified via column chromatography (cyclohexane/ethyl acetate, 10/1, *v/v*) yielding a yellow oil. Yield: 205 mg (0.25 mmol, 75 %). **^{1}H-NMR Data** (400 MHz, MeOD, ppm): 7.68 (s, 1H, triazole-C*H*), 6.71 (s, 6H, Mes-*H*), 4.79 (dd, 1H, *J* = 14.2, 2.4 Hz, CH_2-6a), 4.62 (d, 1H, *J* = 3.7 Hz, C*H*-1), 4.47 (dd, 1H, *J* = 14.2, 8.1 Hz, C*H*-6b), 3.84 (ddd, 1H, *J* = 10.3, 8.1, 2.2 Hz, C*H*-5), 3.62 (dd, 1H, *J* = 9.0, 8.9 Hz, C*H*-3), 3.36 (dd, 1H, *J* = 9.7, 3.7 Hz, C*H*-2), 3.13 (s, 3H, OMe), 3.12 (m, 1H, C*H*-4), 2.53 (t, 2H, *J* = 6.9 Hz, aliphatic-CH_2-1), 2.45 (t, 2H, *J* = 7.7 Hz, aliphatic-CH_2-3), 2.24 (s, 9H, Mes-CH_3), 2.06 (s, 18H, Mes-CH_3), 1.65 (tt, 2H, *J* = 7.1, 7.0 Hz, aliphatic-CH_2-2). **^{13}C-NMR Data** (101 MHz, MeOD, ppm): 233.61 (Mes-*C*=O), 230.58 (aliphatic-*C*=O), 148.01 (triazole-C^q), 142.94 (Mes-*C4*), 141.32 (Mes-*C1*), 133.69 (Mes-*C2*), 129.92 (Mes-*C3*), 124.49 (triazole-*C*H), 101.19 (*C*1), 74.92 (*C*3), 73.32 (*C*2), 72.94 (*C*4), 71.74 (*C*5), 55.53 (OMe), 52.36 (*C*6), 51.52 (aliphatic-*C*1), 25.43 (aliphatic-*C*3), 23.18 (aliphatic-*C*2), 21.32, 19.45 (Mes-CH_3). **UV-vis**: λ [nm] (ε [L mol^{-1} cm^{-1}]) = 354 (1400), 377 (1632), 401 (1214). **MS (ESI-TOF)**: Calculated for [$C_{43}H_{53}GeN_3O_9$]: m/z [M+H]$^+$ 830.3072; found [M+H]$^+$ 830.3095.

Tris(2,4,6-trimethylbenzoyl)(4-(1-phenyl-1,2,3-triazol-4-yl)benzoyl)germane (20)

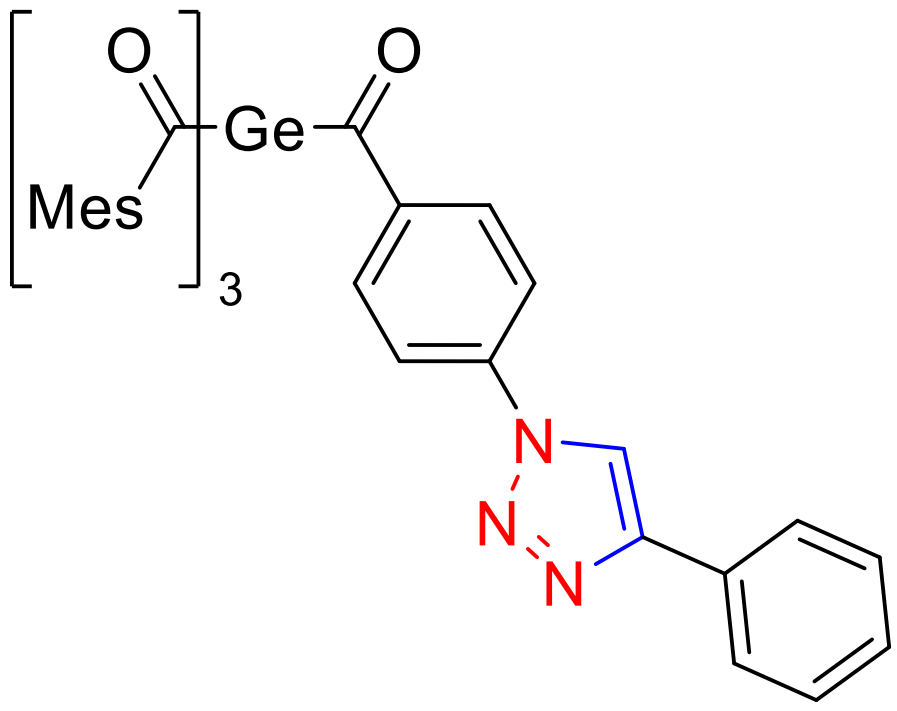

Following the general procedure for click reactions, 200 mg (0.30 mmol, 1.0 eq.) of compound **4** and 37 mg (0.36 mmol, 1.2 eq.) of phenylacetylene were reacted in 10 mL THF in the presence of 3 mg CuI (5 mol%) and 105 µl (0.06 mmol, 2.5 eq) Et_3N. The reaction mixture was purified via column chromatography (cyclohexane/ethyl acetate, 10/1, *v/v*) yielding a yellow crystalline solid. Yield: 201 mg (0.26 mmol, 87 %). **M.p.**: 117-119 °C. **^{1}H-NMR Data** (400 MHz, $CDCl_3$, ppm): 8.21 (s, 1H, triazole-C*H*), 7.90 (s, 4H, aromatic-C*H*), 7.73 (d, 2H, *J* = 8.6 Hz, aromatic-C*H*), 7.49 (t, 2H, *J* = 7.5 Hz, aromatic-C*H*), 7.40 (t, 2H, *J* = 7.5 Hz, aromatic-C*H*), 6.59 (s, 6H, Mes-*H*), 2.17 (s, 9H, Mes-C*H*$_3$), 2.14 (s, 18H, Mes-C*H*$_3$). **^{13}C-NMR Data** (101 MHz, $CDCl_3$, ppm): 232.08 (Mes-*C*=O), 220.46 (aromatic-*C*=O), 149.04 (triazole-C^q), 141.37 (Mes-*C4*), 140.14 (Mes-*C1*), 139.49 (aromatic-*C*), 133.17 (Mes-*C2*), 130.84, 129.91, 129.18 (aromatic-*C*), 128.89 (Mes-*C3*), 126.07, 119.71 (aromatic-*C*), 117.13 (triazole-*C*H), 21.24, 19.38 (Mes-*C*H$_3$). **UV-vis**: λ [nm] (ε [L mol^{-1} cm^{-1}]) = 371 (2231), 379 (2200), 413 (1470). **MS (ESI-TOF)**: Calculated for [$C_{45}H_{43}GeN_3O_4$]: m/z $[M+H]^+$ 764.2543; found $[M+H]^+$ 764.2559.

Tris(2,4,6-trimethylbenzoyl)(4-(4-(4-carboxyphenyl)-1,2,3-triazol-1-yl)benzoyl)germane (21)

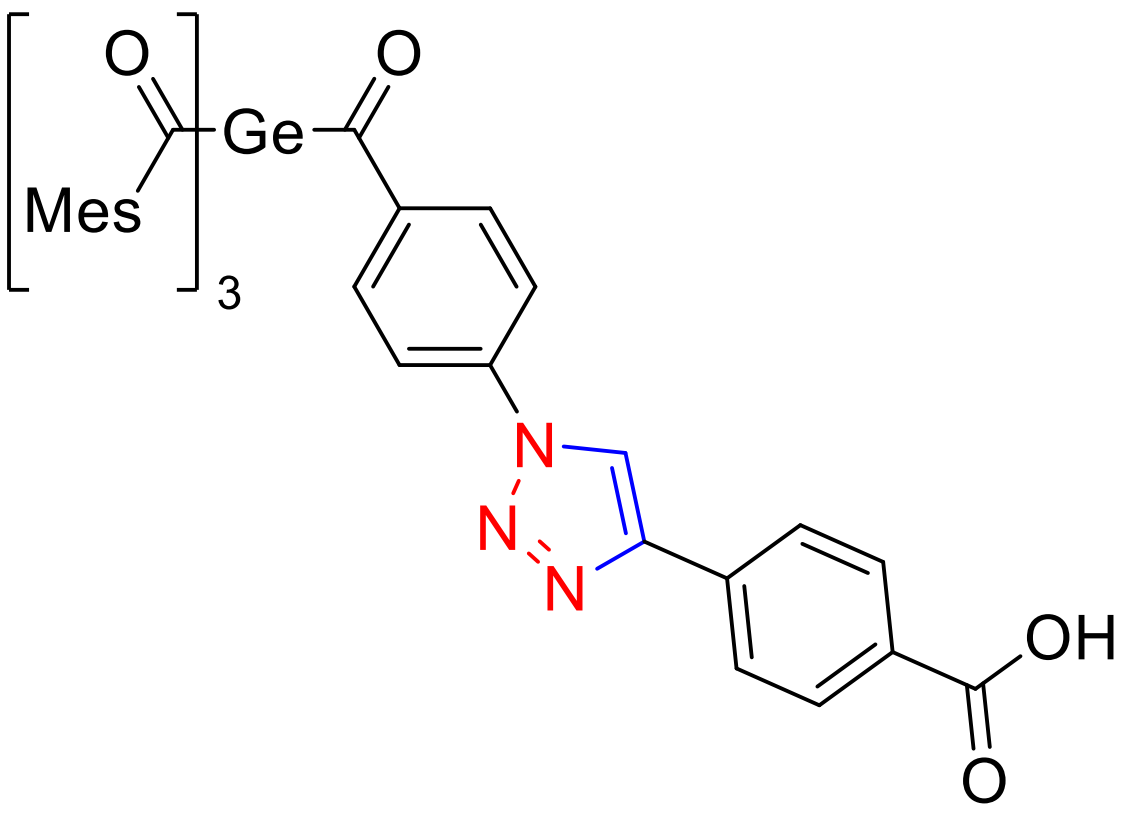

Following the general procedure for click reactions, 200 mg (0.30 mmol, 1.0 eq.) of compound **4** and 53 mg (0.36 mmol, 1.2 eq.) of 4-ethynylbenzoic acid were reacted in 10 mL THF in the presence of 3 mg CuI (5 mol%) and 105 µl (0.06 mmol, 2.5 eq) Et_3N. The reaction mixture was purified via column chromatography (cyclohexane/ethyl acetate, 1/2, *v/v*) yielding a yellow crystalline solid. Yield: 138 mg (0.17 mmol, 57 %). **M.p.**: 167-169 °C. **^{1}H-NMR Data** (400 MHz, DMSO-d6, ppm): 9.60 (s, 1H, triazole-C*H*), 8.09 (s, 4H, aromatic-C*H*), 7.97 (d, 2H, *J* = 8.6 Hz, aromatic-C*H*), 7.77 (d, 2H, *J* = 8.6 Hz, aromatic-C*H*), 6.64 (s, 6H, Mes-*H*), 2.10 (s, 9H, Mes-C*H*$_3$), 2.04 (s, 18H, Mes-C*H*$_3$). **^{13}C-NMR Data** (101 MHz, DMSO-d6, ppm): 231.89 (Mes-*C*=O), 219.49 (aromatic-*C*=O), 166.96 (acid-*C*=O), 146.83 (triazole-C^q), 140.69 (Mes-*C4*), 139.85 (Mes-*C1*), 139.66, 138.44, 133.94 (aromatic-*C*), 132.15 (Mes-*C2*), 130.51, 130.21, 130.16 (aromatic-*C*), 128.61 (Mes-*C3*), 125.38, 120.75 (aromatic-*C*), 119.43 (triazole-*C*H), 20.58, 18.63 (Mes-*C*H$_3$). **UV-vis**: λ [nm] (ε [L mol^{-1} cm^{-1}]) = 370 (1272), 379 (1257), 421 (681). **MS (ESI-TOF)**: Calculated for [$C_{46}H_{43}GeN_3O_6$]: m/z $[M+H]^+$ 808.2442; found $[M+H]^+$ 808.2443.

Tris(2,4,6-trimethylbenzoyl)(4-(4-(4-(methoxycarbonyl)phenyl)-1,2,3-triazol-1-yl)benzoyl)germane (22)

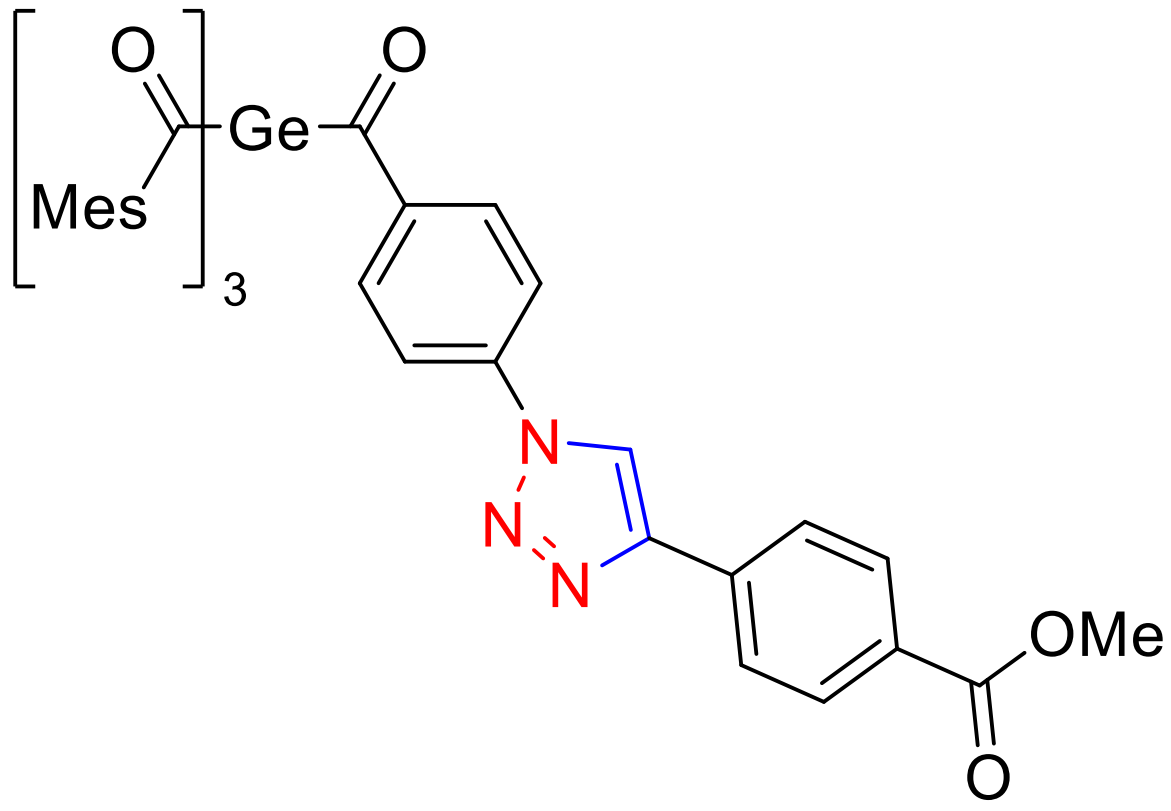

Following the general procedure for click reactions, 200 mg (0.30 mmol, 1.0 eq.) of compound **4** and 58 mg (0.36 mmol, 1.2 eq.) of 4-ethynylbenzoic acid methyl ester were reacted in 10 mL THF in the presence of 3 mg CuI (5 mol%) and 10 µl (0.06 mmol, 0.2 eq) Et_3N. The reaction mixture was purified via column chromatography (cyclohexane/ethyl acetate, 10/1, *v/v*) yielding a crystalline yellow solid. Yield: 217 mg (0.26 mmol, 88 %). **M.p.**: 154-157 °C **^{1}H-NMR Data** (400 MHz, $CDCl_3$, ppm): 8.31 (s, 1H, triazole-C*H*), 8.15 (d, 2H, *J* = 8.4 Hz, aromatic-C*H*), 8.01 (d, 2H, *J* = 8.6 Hz, aromatic-C*H*), 7.90 (d, 2H, *J* = 8.7 Hz, aromatic-C*H*), 7.73 (d, 2H, *J* = 8.7 Hz, aromatic-C*H*), 6.58 (s, 6H, Mes-*H*), 3.95 (s, 3H, ester-CH_3),2.16 (s, 9H, Mes-CH_3), 2.13 (s, 18H, Mes-CH_3). **^{13}C-NMR Data** (101 MHz, $CDCl_3$, ppm): 232.01 (Mes-*C*=O), 220.52 (aromatic-*C*=O), 166.81 (ester-*C*=O), 147.98 (triazole-C^q), 141.33 (Mes-*C4*), 140.16 (Mes-*C1*), 139.79, 139.64, 134.18 (aromatic-*C*), 133.16 (Mes-*C2*), 130.87, 130.52, 130.31 (aromatic-*C*), 128.89 (Mes-*C3*), 125.85, 119.81 (aromatic-*C*), 118.09 (triazole-CH), 52.40 (ester-CH_3), 21.25, 19.38 (Mes-CH_3). **UV-vis**: λ [nm] (ε [L mol^{-1} cm^{-1}]) = 371 (1742), 378 (1731), 401 (1300), 420 (513), 446 (481). **MS (ESI-TOF)**: Calculated for [$C_{47}H_{45}GeN_3O_6$]: m/z $[M+H]^+$ 882.2598; found $[M+H]^+$ 822.2584.

Tris(2,4,6-trimethylbenzoyl)(4-(4-(4-(pinacolboronyl)phenyl)-1,2,3-triazol-1-yl)benzoyl)germane (23)

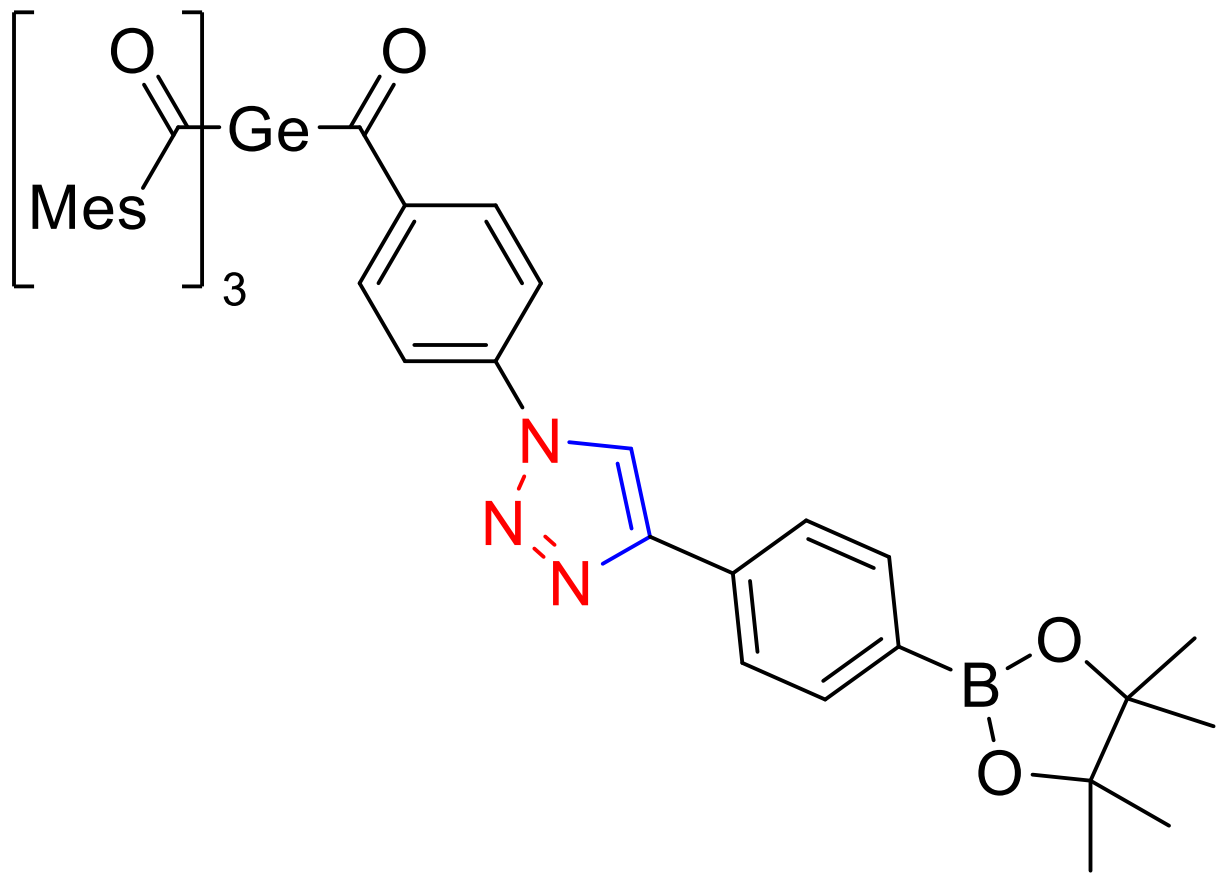

Following the general procedure for click reactions, 200 mg (0.30 mmol, 1.0 eq.) of compound **4** and 82 mg (0.36 mmol, 1.2 eq.) of 4-ethynylbenzeneboronic acid pinacol ester were reacted in 10 mL THF in the presence of 3 mg CuI (5 mol%) and 10 µl (0.06 mmol, 0.2 eq) Et_3N. The reaction mixture was purified via column chromatography (cyclohexane/ethyl acetate, 10/1, *v/v*) yielding a yellow wax. Yield: 184 mg (0.26 mmol, 69 %). **M.p.**: 106-109 °C. **^{1}H-NMR Data** (400 MHz, $CDCl_3$, ppm): 8.25 (s, 1H, triazole-C*H*), 7.93 (s, 4H, aromatic-C*H*), 7.88 (d, 2H, *J* = 8.6 Hz, aromatic-C*H*), 7.72 (d, 2H, *J* = 8.6 Hz, aromatic-C*H*), 6.58 (s, 6H, Mes-*H*), 2.16 (s, 9H, Mes-CH_3), 2.13 (s, 18H, Mes-CH_3), 1.37 (s, 12H, pinacol-CH_3). **^{13}C-NMR Data** (101 MHz, $CDCl_3$, ppm): 232.09 (Mes-*C*=O), 220.50 (aromatic-*C*=O), 148.91 (triazole-C^q), 141.36 (Mes-*C4*), 140.16 (Mes-*C1*), 139.95, 139.50, 135.61 (aromatic-*C*), 133.17 (Mes-*C2*), 132.36, 130.84 (aromatic-*C*), 128.89 (Mes-*C3*), 128.84, 125.21, 119.74 (aromatic-*C*), 117.58 (triazole-CH), 84.14 (pinacol-C^q), 25.05, 21.25 (Mes-CH_3), 19.39 (pinacol-CH_3). **UV-vis**: λ [nm] (ε [L mol^{-1} cm^{-1}]) = 378 (2028), 402 (1418), 425 (937). **MS (ESI-TOF)**: Calculated for [$C_{51}H_{54}BGeN_3O_6$]: m/z $[M+H]^+$ 890.3395; found $[M+H]^+$ 890.3432.

Tris(2,4,6-trimethylbenzoyl)(4-(4-(tris(2,4,6-trimethylbenzoyl)germanyl)-1,2,3-triazol-1-yl)benzoyl)germane (24)

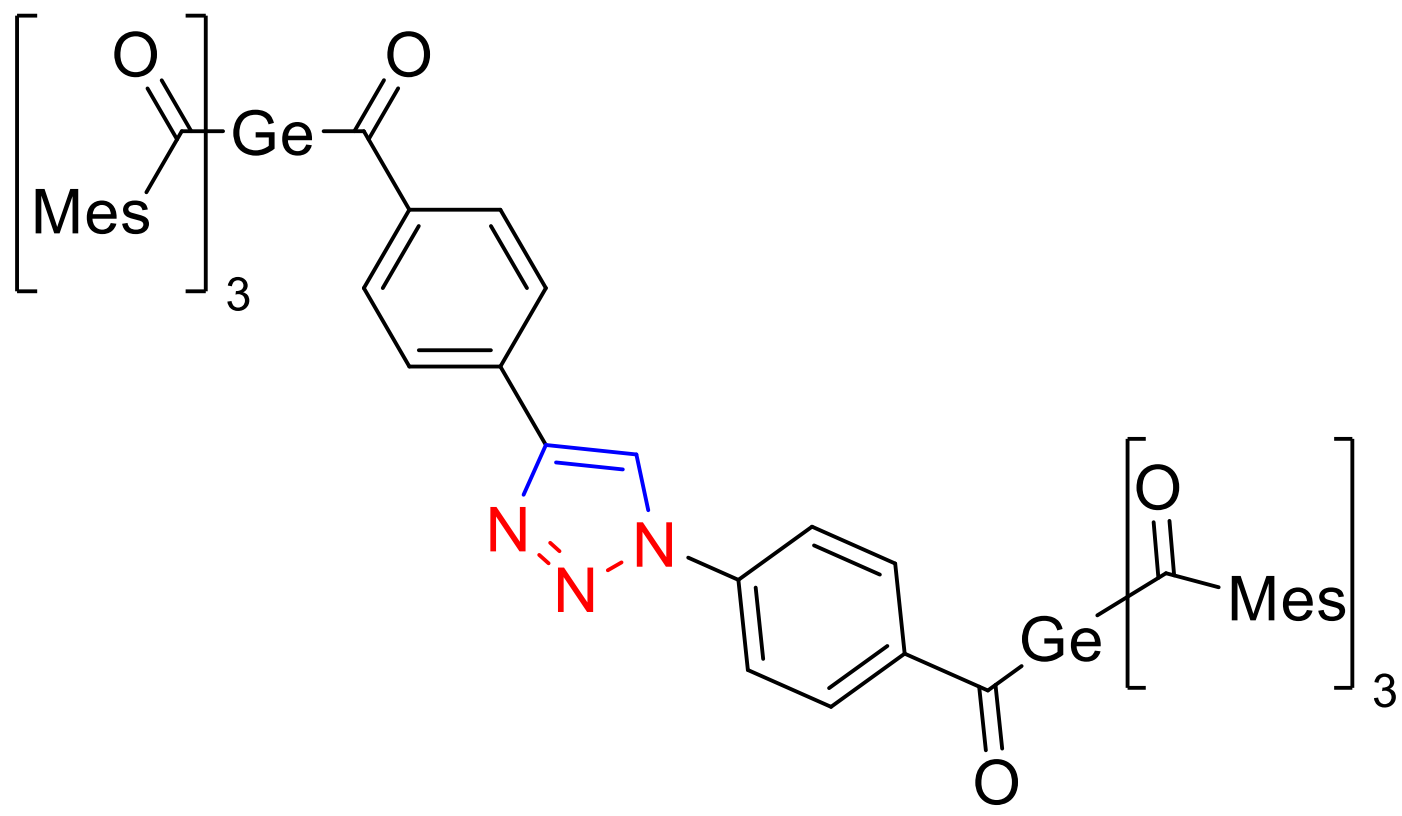

Following the general procedure for click reactions, 100 mg (0.15 mmol, 1.0 eq.) of compound **4** and 103 mg (0.16 mmol, 1.05 eq.) of compound **2** were reacted in 10 mL THF in the presence of 3 mg CuI (5 mol%) and 10 µl (0.06 mmol, 0.2 eq) Et_3N. The reaction mixture was purified via column chromatography (cyclohexane/ethyl acetate, 10/1, *v/v*) yielding a yellow oil. Yield: 170 mg (0.13 mmol, 86 %). **Anal. Calc.** (%) for $C_{76}H_{75}Ge_2N_3O_8$: C, 70.02; H, 5.80; N, 3.22. Found: C, 69.83; H, 5.92; N, 3.18. **^{1}H-NMR Data** (400 MHz, $CDCl_3$, ppm): 8.31(s, 1H, triazole-C*H*), 7.88 (d, 2H, *J* = 8.7 Hz, aromatic-C*H*), 7.82 (m, 4H, aromatic-C*H*), 7.74 (d, 2H, *J* = 8.7 Hz, aromatic-C*H*), 6.58 (s, 6H, Mes-*H*), 2.16 (s, 54H, Mes-C*H*$_3$). **^{13}C-NMR Data** (101 MHz, $CDCl_3$, ppm): 232.52, 231.98 (Mes-*C*=O), 220.53, 220.50 (aromatic-*C*=O), 147.65 (triazole-*C*^{q}), 141.43, 141.30 (Mes-*C4*), 140.14, 139.96 (Mes-*C1*), 139.72, 139.67, 139.65, 134.35 (aromatic-*C*), 133.13 (Mes-*C2*), 130.87, 129.92 (aromatic-*C*), 128.87, 128.84 (Mes-*C3*), 125.72, 119.75 (aromatic-*C*), 118.15 (triazole-*C*H), 21.24, 21.22, 19.37 (Mes-*C*H$_3$). **UV-vis**: λ [nm] (ε [L mol^{-1} cm^{-1}]) = 376 (2248), 397 (1961), 430 (1124).

Tris(2,4,6-trimethylbenzoyl)((1-(4-(tris(2,4,6-trimethylbenzoyl)germanyl)benzoyl)-1,2,3-triazol-4-yl)methyl)germane (25)

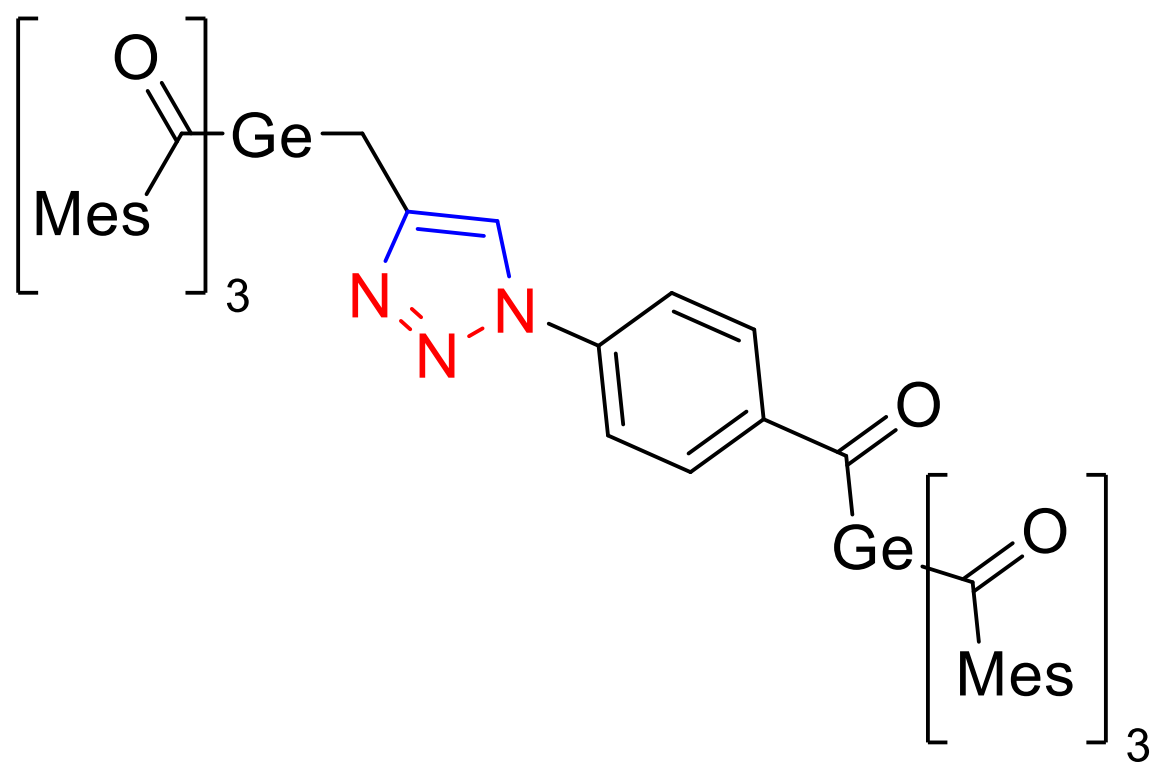

Following the general procedure for click reactions, 100 mg (0.15 mmol, 1.0 eq.) of compound **4** and 89 mg (0.16 mmol, 1.05 eq.) of compound **1** were reacted in 10 mL THF in the presence of 3 mg CuI (5 mol%) and 10 µl (0.06 mmol, 0.2 eq) Et_3N. The reaction mixture was purified via column chromatography (cyclohexane/ethyl acetate, 10/1, *v/v*) yielding a yellow oil. Yield: 140 mg (0.12 mmol, 77 %). **^{1}H-NMR Data** (400 MHz, $CDCl_3$, ppm): 7.84 (d, 2H, *J* = 8.6 Hz, aromatic-C*H*), 7.76 (s, 1H, triazole-C*H*), 7.66 (d, 2H, *J* = 8.6 Hz, aromatic-C*H*), 6.67 (s, 6H, Mes-*H*), 6.57 (s, 6H, Mes-*H*), 2.95 (s, 2H, Ge-C*H*$_2$), 2.20 (s, 9H, Mes-C*H*$_3$), 2.15 (s, 9H, Mes-C*H*$_3$), 2.13 (s, 18H, Mes-C*H*$_3$), 2.06 (s, 18H, Mes-C*H*$_3$). **^{13}C-NMR Data** (101 MHz, $CDCl_3$, ppm): 234.70, 232.11 (Mes-*C*=O), 220.25 (aromatic-*C*=O), 144.25 (triazole-*C*^{q}), 141.65, 141.36 (Mes-*C4*), 140.13, 139.69 (Mes-*C1*), 139.06 (aromatic-*C*), 133.14, 133.00 (Mes-*C2*), 130.77, 129.08 (aromatic-*C*), 128.92, 128.89 (Mes-*C3*), 119.23 (aromatic-*C*), 118.88 (triazole-*C*H), 21.24, 19.37, 19.24 (Mes-*C*H$_3$), 13.80 (Ge-*C*H$_2$). **UV-vis**: λ [nm] (ε [L mol^{-1} cm^{-1}]) = 368 (2254), 380 (2327), 399 (1965). **MS (ESI-TOF)**: Calculated for [$C_{70}H_{73}Ge_2N_3O_7$]: m/z [M+H]$^+$ 1216.3950; found [M+H]$^+$ 1214.3974 (major isotope cluster, Ge isotopes).

Tris(2,4,6-trimethylbenzoyl)((1-(4-(tris(2,4,6-trimethylbenzoyl)germanyl)benzoyl)-1,2,3-triazol-4-yl)butanoyl)germane (26)

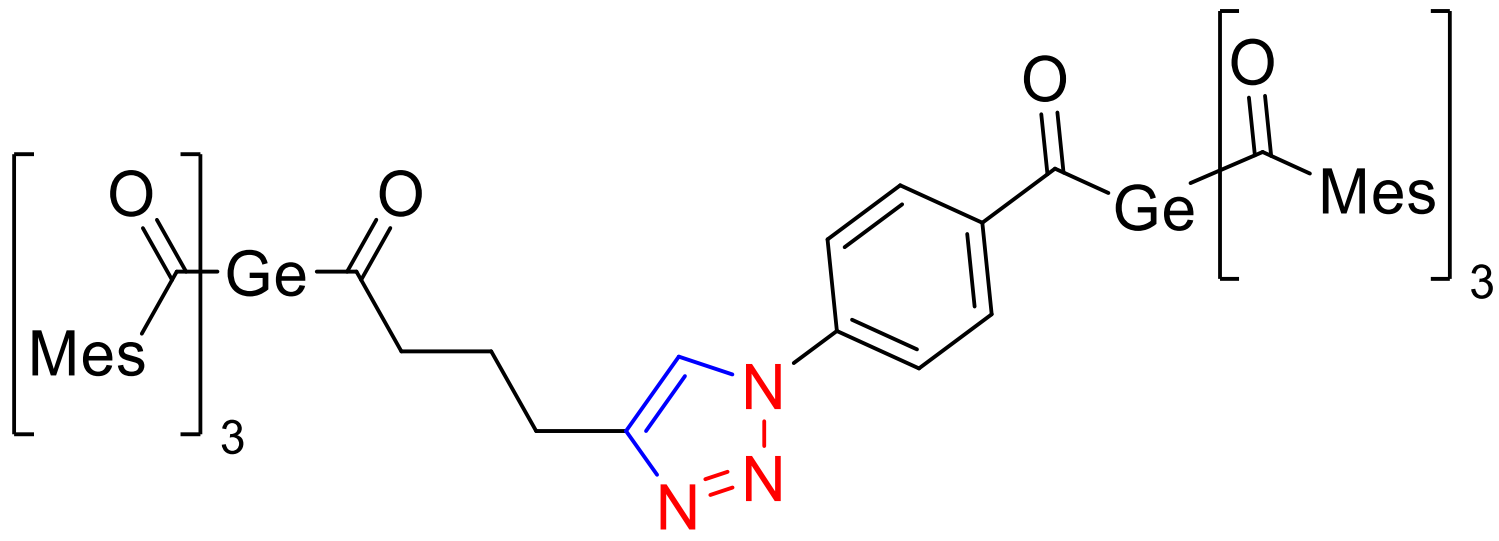

Following the general procedure for click reactions, 100 mg (0.15 mmol, 1.0 eq.) of compound **4** and 98 mg (0.16 mmol, 1.05 eq.) of compound **3** were reacted in 10 mL THF in the presence of 3 mg CuI (5 mol%) and 10 µl (0.06 mmol, 0.2 eq) Et_3N. The reaction mixture was purified via column chromatography (cyclohexane/ethyl acetate, 10/1, *v/v*) yielding a yellow oil. Yield: 154 mg (0.12 mmol, 81 %). **Anal. Calc.** (%) for $C_{73}H_{77}Ge_2N_3O_8$: C, 69.06; H, 6.11; N, 3.31. Found: C, 68.90; H, 6.18; N, 3.28. **^{1}H-NMR Data** (400 MHz, $CDCl_3$, ppm): 7.84 (d, 2H, J = 8.6 Hz, aromatic-C*H*), 7.76 (s, 1H, triazole-C*H*), 7.66 (d, 2H, J = 8.6 Hz, aromatic-C*H*), 6.67 (s, 6H, Mes-*H*), 6.57 (s, 6H, Mes-*H*), 2.58 (m, 4H, aliphatic-CH_2-1, aliphatic-CH_2-3), 2.23 (s, 9H, Mes-CH_3), 2.15 (s, 9H, Mes-CH_3), 2.12 (s, 18H, Mes-CH_3), 2.09 (s, 18H, Mes-CH_3), 1.80 (tt, 2H, J = 8.7, 7.0 Hz, aliphatic-CH_2-2). **^{13}C-NMR Data** (101 MHz, $CDCl_3$, ppm): 232.18, 232.11 (Mes-*C*=O), 230.15 (aromatic-*C*=O), 220.39 (aliphatic-*C*=O), 148.73 (triazole-C^q), 141.70, 141.36 (Mes-*C4*), 140.11, 140.05 (Mes-*C1*), 139.25 (aromatic-*C*), 133.15, 132.82 (Mes-*C2*), 130.80 (aromatic-*C*), 128.96, 128.87 (Mes-*C3*), 119.52 (aromatic-*C*), 118.70 (triazole-*C*H), 50.47 (aliphatic-*C*1), 24.80 (aliphatic-*C*3), 21.92 (aliphatic-*C*2), 21.30, 21.22, 19.36, 19.21 (Mes-*C*H_3). **UV-vis**: λ [nm] (ε [L mol^{-1} cm^{-1}]) = 364 (3236), 377 (3148), 399 (2272).

Single Crystal X-ray Crystallography

All crystals suitable for single crystal X-ray diffractometry were removed from a vial and immediately covered with a layer of silicone oil. A single crystal was selected, mounted on a copper pin, and placed in the cold N_2 stream provided by an Oxford Cryosystems cryostream. Data for presented crystal structures were collected using a Rigaku XtaLAB Synergy, Dualflex, HyPix-Arc 100 diffractometer. Data were measured using either Mo Kα (λ= 0.71073 Å) or Cu Kα (λ= 1.54056 Å) radiation. The diffraction pattern was indexed and the total number of runs and images was based on the strategy calculation from the program CrysAlisPro.[62] The unit cell was refined and data reduction, scaling and absorption corrections were performed using CrysAlisPro. Using Olex2,[63] the structure was solved with the SHELXT[64] structure solution program and refined with the SHELXL[65] refinement package using full matrix least squares minimization on F^2. The absolute configuration was established by anomalous dispersion effects in the diffraction measurements on the crystal. All non-hydrogen atoms were refined anisotropically. Hydrogen atoms were placed in calculated positions corresponding to standard bond lengths and angles and refined using a riding model. Disorder was handled by modelling the occupancies of the individual orientations using free variables to refine the respective occupancy of the affected fragments (PART).[66] In some cases, the distances between arbitrary atom pairs were restrained to possess the same value using the SADI instruction. Disordered positions for the methyl ester moiety in **16** were refined using 60/40 split positions. Hydrogen atoms for the disordered methyl ester moiety were located in the difference map and refined freely. Disordered positions for the oxygen of an acyl moiety in **20** were refined using 60/40 split positions. Compound **1** was refined as a 2-component inversion twin (BASF 0.50). The heavily disordered solvents of crystallization for compounds **2** (acetone), **23** (acetone) and **4p** (chloroform) were removed from the refinement by using the “squeeze” option available in the PLATON program suite.[67-68]

All crystal structures representations were made with the program Mercury[69] with all non-carbon atoms displayed as 30% spheres. CIF files were edited, validated, and formatted either with the programs Encifer[70] or Olex2.[63] CCDC **2541768-2541778** contain the supplementary crystallographic data for compounds **1-4**, **4p**, **14-16**, **20**, **21**, **23**. These data can be obtained free of charge from The Cambridge Crystallographic Data Centre *via* www.ccdc.cam.ac.uk/data_request/cif. Tables S1 and S2 contain crystallographic data and details of measurements and refinement for the mentioned compounds.

Table S1. Crystallographic data and details of measurements for compounds **1**-**4** and **4p**.
R1= Σ/ |F$_o$|- |F$_c$|/|Σ|F$_d$; wR2 = [Σ$_w$(F$_o$^{2}-F$_2$^{2})^2/Σ$_w$(F$_o$^{2})^2]$^{1/2}$

**Compound** (CCDC Nr.)	**1** (2541778)	**2** (2541773)	**3** (2541777)	**4** (2541775)	**4p** (2541774)
Formula	$C_{33}H_{36}GeO_3$	$C_{39}H_{38}GeO_4·C_4H_8O$	$C_{36}H_{40}GeO_4$	$C_{37}H_{37}GeN_3O_4$	$C_{53}H_{65}GeN_4O_4P·C_3H_6O$
Fw (g·mol^{-1})	553.21	715.39	609.27	660.28	983.72
a (Å)	17.8018(2)	25.98672(10)	13.7703(1)	8.0634(1)	14.5905(1)
b (Å)	11.9726(1)	11.61008(4)	15.8028(1)	14.2254(1)	15.5774(1)
c (Å)	26.5393(2)	25.02644(9)	14.7799(1)	14.7951(1)	15.8870(1)
α (°)	90	90	90	95.895 (1)	111.363(1)
β (°)	90	90.4627(3)	93.836(1)	105.452(1)	98.128(1)
γ (°)	90	90	90	92.376(1)	114.871(1)
V (Å^3)	5656.42(9)	7550.43 (5)	3209.04(4)	1622.88(3)	2859.42(4)
Z	8	8	4	2	2
Crystal size (mm)	0.14 × 0.05 × 0.05	0.12 × 0.1 × 0.06	0.10 × 0.06 × 0.05	0.19 × 0.11 × 0.1	0.19 × 0.15 × 0.08
Crystal habit	Block, yellow	Plate, yellow	Block, yellow	Block, yellow	Block, orange
Crystal system	Orthorhombic	Monoclinic	Monoclinic	Triclinic	Triclinic
Space group	*Pna*2$_1$	*C*2/*c*	*P*2$_1$/*c*	*P*-1	*P*-1
ρ_{calc} (Mg·m^{-3})	1.299	1.259	1.261	1.351	1.143
μ (mm^{-1})	1.71	1.44	1.58	0.99	1.35
T (K)	100(2)	100(2)	100(2)	100(2)	100(2)
θ range (°)	3.3–77.3 (Cu *K*α)	3.4–77.5 (Cu *K*α)	3.2–77.3 (Cu *K*α)	2.6–35.8 (Mo *K*α)	3.2–79.3 (Cu *K*α)
F(000)	2320	3008	1280	688	1160
T_{min}, T_{max}	0.773, 1.000	0.837, 1.000	0.935, 1.000	0.468, 1.000	0.280, 0.875
R_{int}	0.040	0.042	0.025	0.044	0.041
No. of measured, independent, and observed [$I > 2\sigma(I)$] reflections	111316, 11219, 10646	288754, 7972, 7680	33968, 6566, 5996	228157, 10797, 9786	122223, 12111, 11267
independent reflections	11219	7972	6566	10797	12111
No. of parameters, restraints	686,1	451, 0	379, 0	415, 0	623, 0
$\Delta\rho_{max}$, $\Delta\rho_{min}$ (e·Å^{-3})	0.43, -0.42	0.44, -0.40	0.30, -0.25	0.56, -0.33	1.50, -0.52
Absolute structure parameter	0.501(19)	—	—	—	—
GooF	1.05	1.04	1.04	1.06	1.05
R1, wR2 (all data)	R1 = 0.0325 wR2 = 0.0767	R1 = 0.0322 wR2 = 0.0841	R1 = 0.0300 wR2 = 0.0730	R1 = 0.0338 wR2 = 0.0746	R1 = 0.0402 wR2 = 0.1093
R1, wR2 (>2*σ*)	R1 = 0.0303 wR2 = 0.0757	R1 = 0.0312 wR2 = 0.0833	R1 = 0.0271 wR2 = 0.0745	R1 = 0.0291 wR2 = 0.0730	R1 = 0.0382 wR2 = 0.1076

Table S2. Crystallographic data and details of measurements for compounds **14-16**, **20**, **21**, **23**.
R1= Σ/ $|F_o|$- $|F_c|$/|Σ|F_d; wR2 = $[\Sigma_w(F_o^2\text{-}F_2^2)^2/\Sigma_w(F_o^2)^2]^{1/2}$

**Compound** (CCDC Nr.)	**14** (2541771)	**15** (2541772)	**16** (2541768)	**20** (2541769)	**21** (2541776)	**23** (2541770)
Formula	$C_{45}H_{43}GeN_3O_4$	$C_{46}H_{43}GeN_3O_6$	$C_{47}H_{45}GeN_3O_6$	$C_{45}H_{43}GeN_3O_4$	$C_{46}H_{43}GeN_3O_6$	$C_{51}H_{54}BGeN_3O_6 \cdot C_3H_6O$
Fw (g·mol^{-1})	762.41	806.42	820.45	762.41	806.42	946.45
a (Å)	16.1118(2)	16.12335(11)	11.5694(3)	16.3681(6)	14.8042(2)	7.4287(1)
b (Å)	14.1237(1)	15.97499(13)	13.6545(4)	14.7037(1)	14.3253(2)	13.7741(1)
c (Å)	33.4980(4)	15.19507(9)	14.0139(2)	16.970(3)	37.6217(7)	26.3011(1)
α (°)	90	90	73.946(2)	90	90	82.659(1)
β (°)	91.913(1)	96.3082(6)	76.455(2)	112.055(13)	90	82.124(1)
γ (°)	90	90	72.609(3)	90	90	80.273(1)
V (Å^3)	7618.50(14)	3890.10 (5)	2002.33(9)	3785.3(8)	7978.6(2)	2612.38(4)
Z	8	4	2	4	8	2
Crystal size (mm)	0.12 × 0.08 × 0.01	0.23 × 0.18 × 0.15	0.07 × 0.06 × 0.04	0.13 × 0.07 × 0.06	0.14 × 0.12 × 0.03	0.27 × 0.06 × 0.03
Crystal habit	Block, yellow	Block, yellow	Block, yellow	Block, yellow	Block, yellow	Block, yellow
Crystal system	Monoclinic	Monoclinic	Triclinic	Monoclinic	Orthorhombic	Triclinic
Space group	*I*2/*a*	*P*2$_1$/*c*	*P*-1	*P*2$_1$/*n*	*Pbca*	*P*-1
ρ_{calc} (Mg·m^{-3})	1.329	1.377	1.361	1.338	1.343	1.203
μ (mm^{-1})	1.47	1.51	1.48	1.48	1.47	1.21
T (K)	100(2)	100(2)	100(2)	100(2)	100(2)	100(2)
θ range (°)	3.4–78.7 (Cu *K*α)	3.9–78.6 (Cu *K*α)	3.3–77.4 (Cu *K*α)	3.2–77.4 (Cu *K*α)	3.8–78.3 (Cu *K*α)	3.2–78.3 (Cu *K*α)
F(000)	3184	1680	856	1592	3360	996
T_{min}, T_{max}	0.715, 1.000	0.712, 1.000	0.981, 0.981	0.715, 1.000	0.761, 1.000	0.675, 1.000
R_{int}	0.042	0.043	0.054	0.032	0.057	0.040
No. of measured, independent, and observed [$I > 2\sigma(I)$] reflections	67649, 8162, 7139	67912, 8362, 7891	35618, 8367, 7071	82473, 8022, 7328	37921, 8424, 6158	112013, 11075, 10347
independent reflections	8162	8362	8367	8022	8424	11075
No. of parameters, restraints	487, 0	515, 0	575, 4	496, 0	514, 0	610, 0
$\Delta\rho_{max}$, $\Delta\rho_{min}$ (e·Å^{-3})	0.37, -0.50	0.32, -0.51	0.62, -0.79	0.29, -0.33	0.39, -0.53	0.49, -0.53
Absolute structure parameter	—	—	—	—	—	—
GooF	1.07	1.05	1.07	1.04	1.03	1.04
R1, wR2 (all data)	R1 = 0.0427 wR2 = 0.1032	R1 = 0.0343 wR2 = 0.0825	R1 = 0.0578 wR2 = 0.1306	R1 = 0.0303 wR2 = 0.0734	R1 = 0.0646 wR2 = 0.0975	R1 = 0.0378 wR2 = 0.0984
R1, wR2 (>2*σ*)	R1 = 0.0372 wR2 = 0.1000	R1 = 0.0324 wR2 = 0.0815	R1 = 0.0486 wR2 = 0.1359	R1 = 0.0273 wR2 = 0.0720	R1 = 0.0415 wR2 = 0.0907	R1 = 0.0355 wR2 = 0.0969

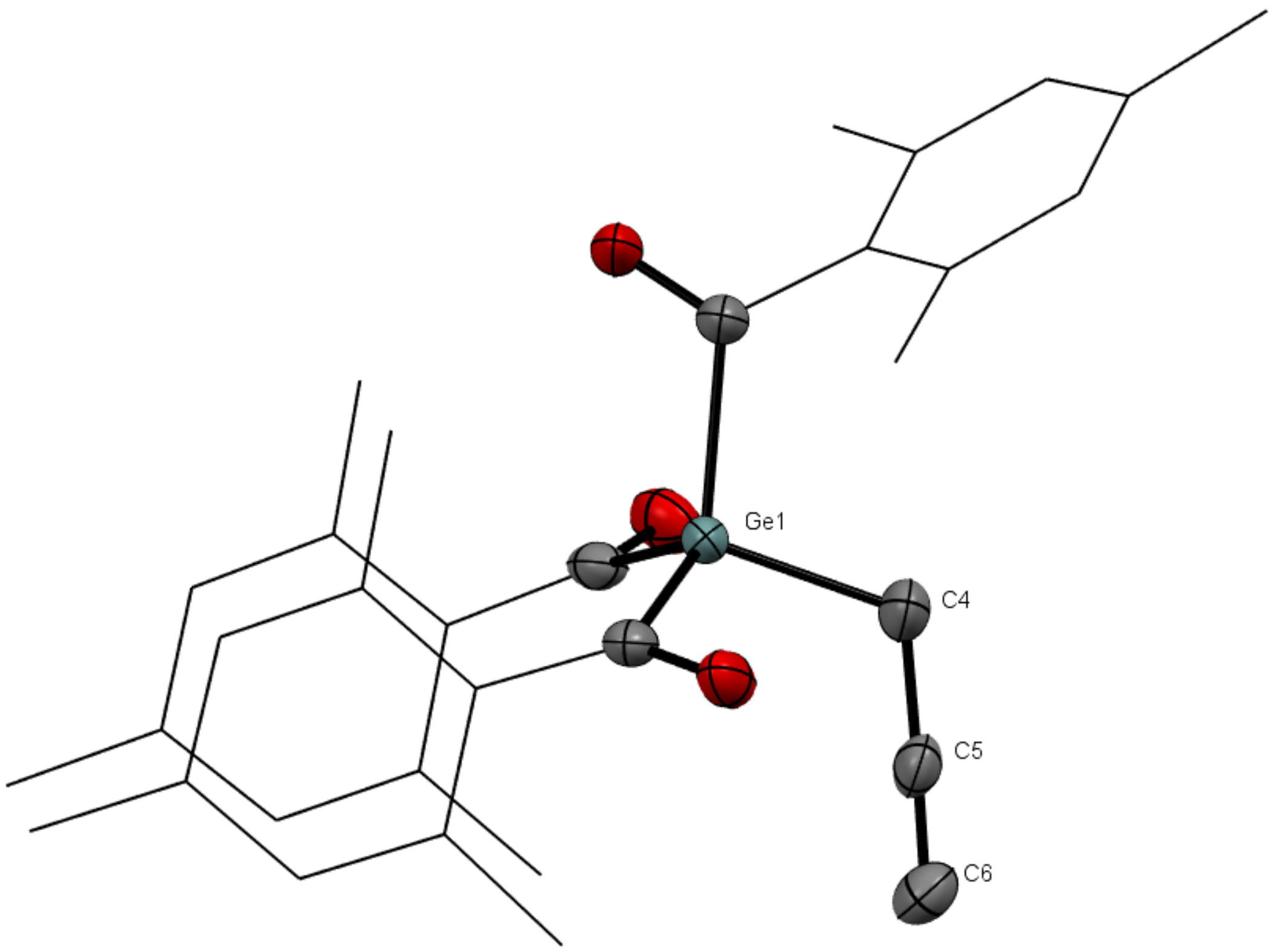

Figure S1. ORTEP representation of compound **1**. Mesityl groups are displayed in wireframe style for clarity.

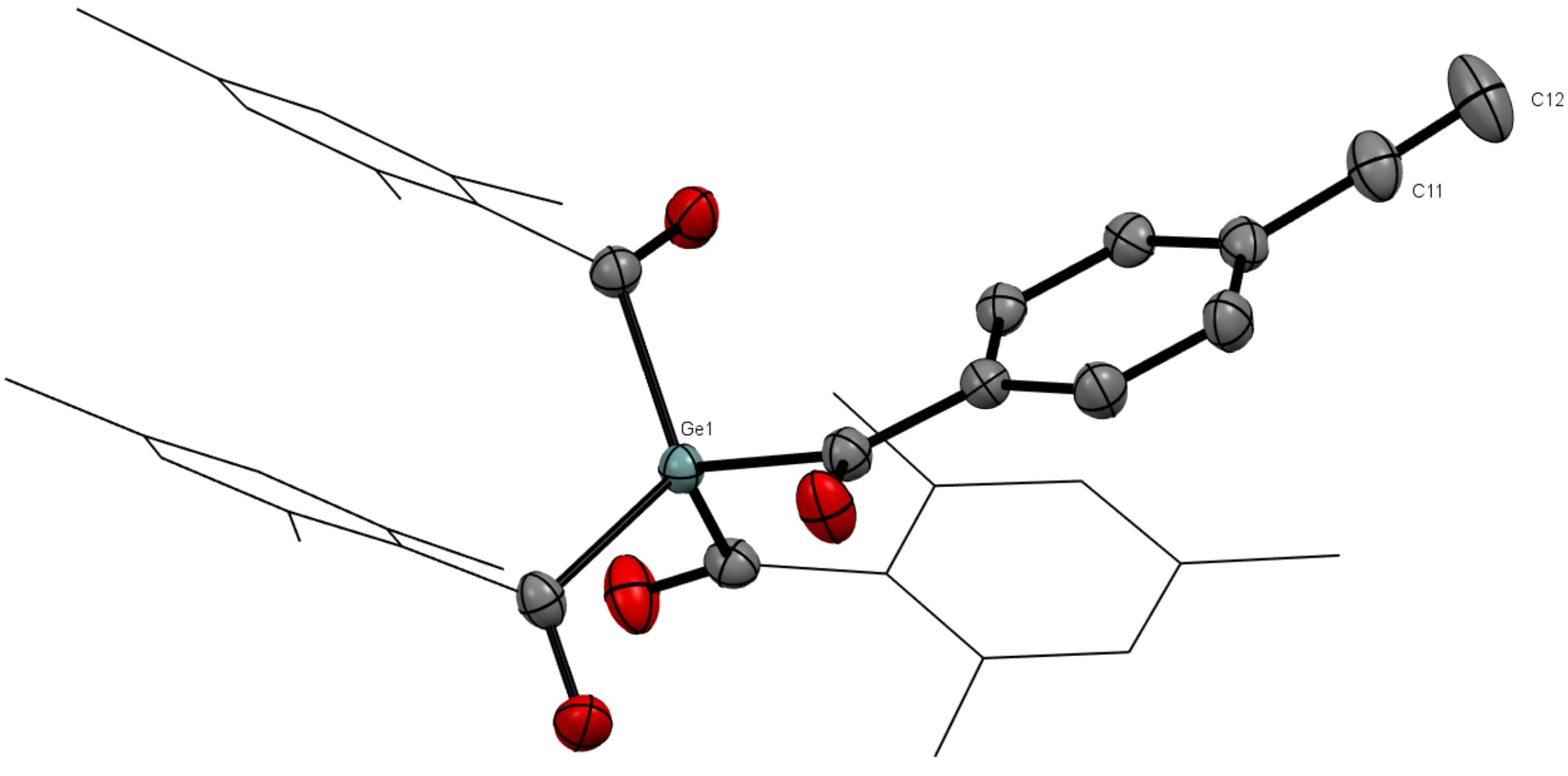

Figure S2. ORTEP representation of compound **2**. Mesityl groups are displayed in wireframe style for clarity.

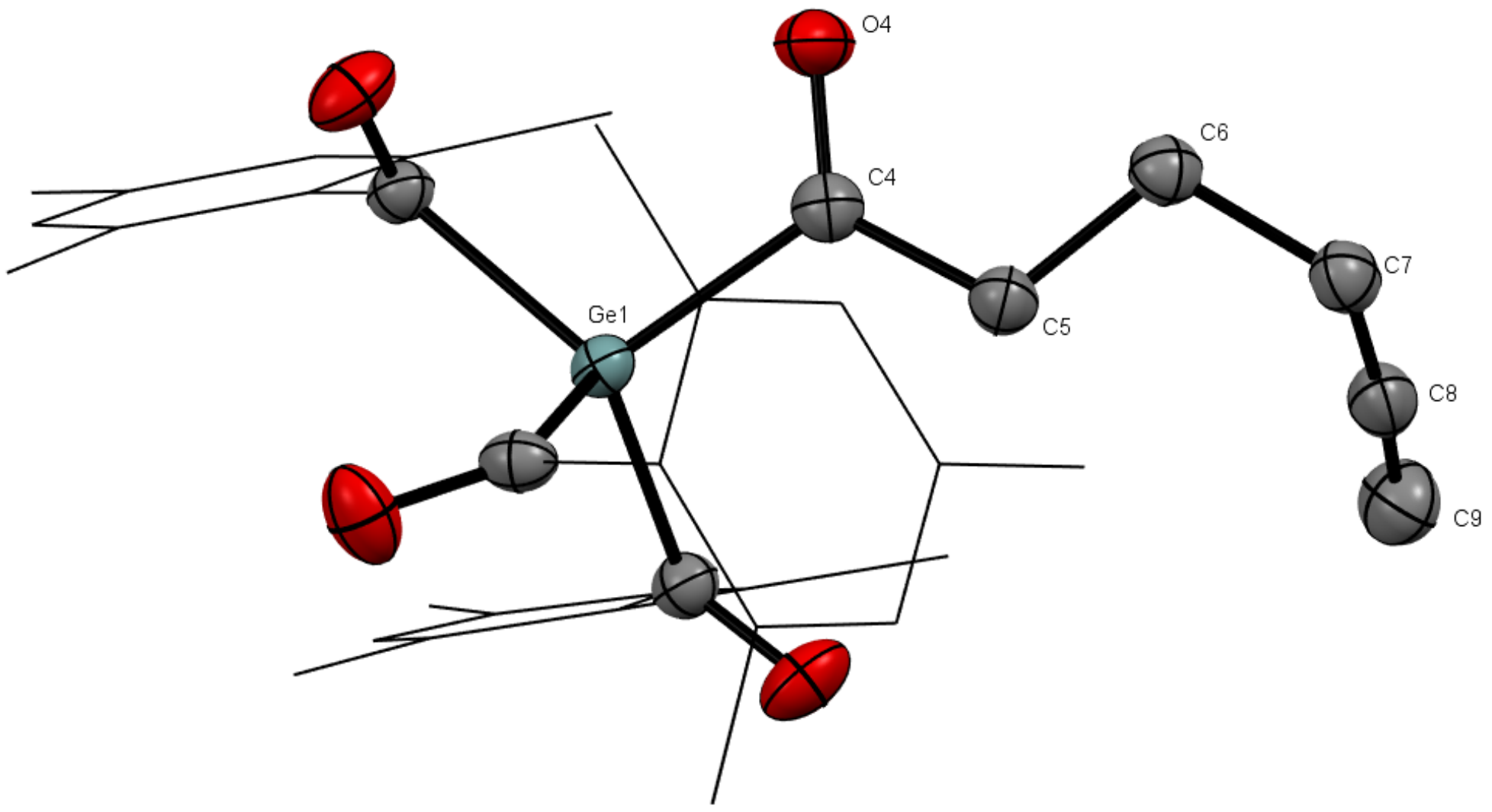

Figure S3. ORTEP representation of compound **3**. Mesityl groups are displayed in wireframe style for clarity.

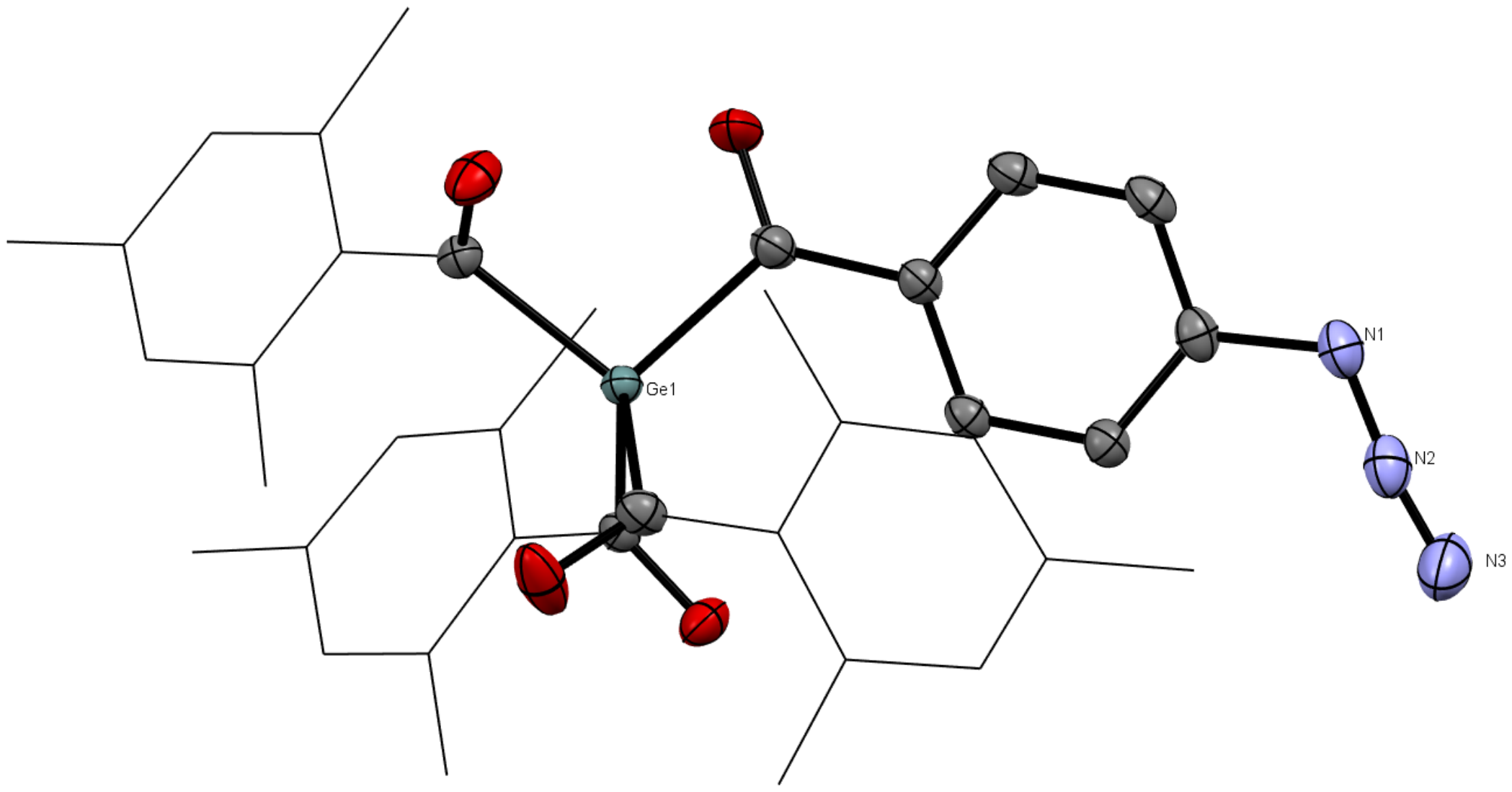

Figure S4. ORTEP representation of compound **4**. Mesityl groups are displayed in wireframe style for clarity.

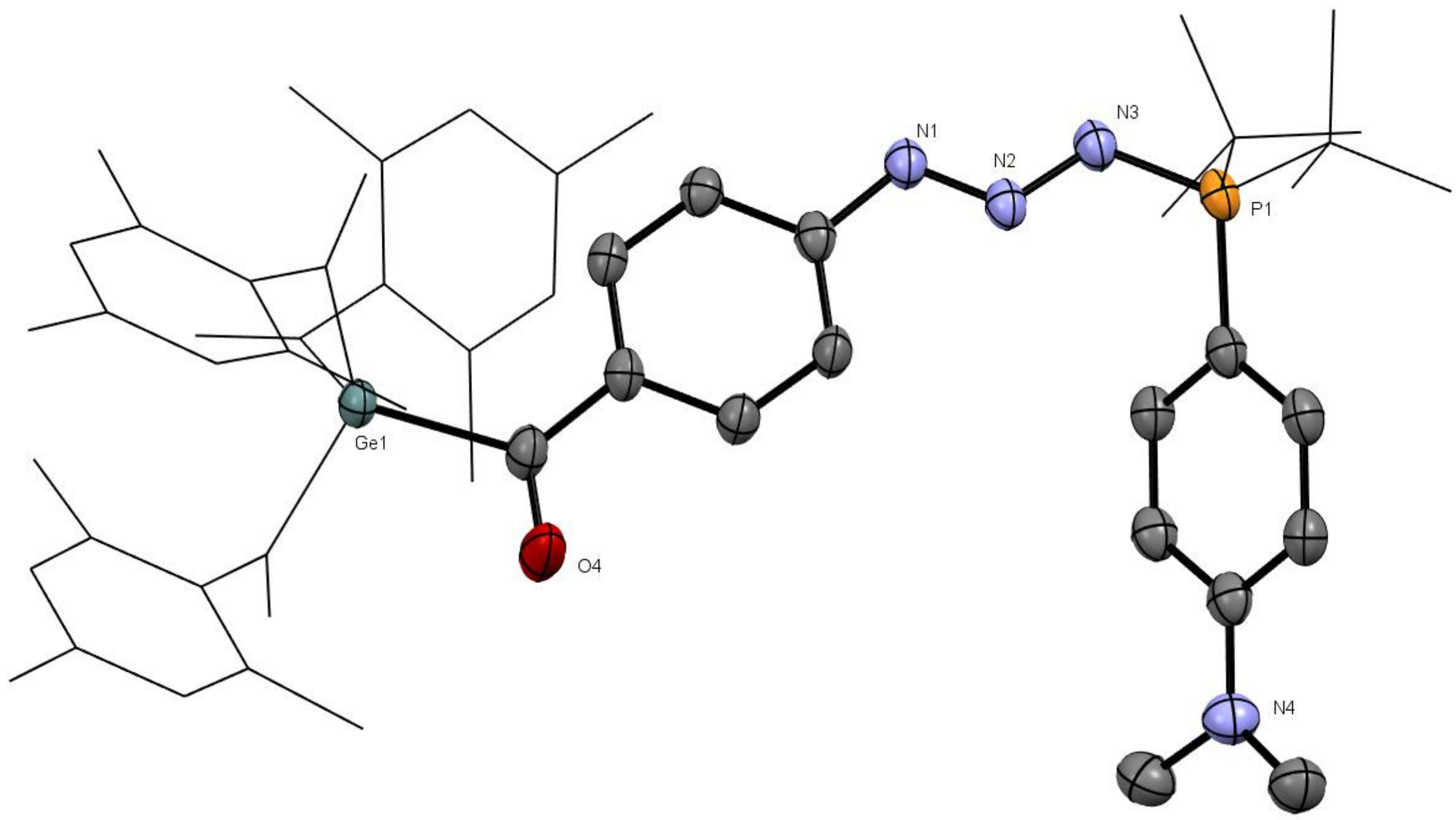

Figure S5. ORTEP representation of compound **4p**. Mesityl and *t*-butyl groups are displayed in wireframe style for clarity.

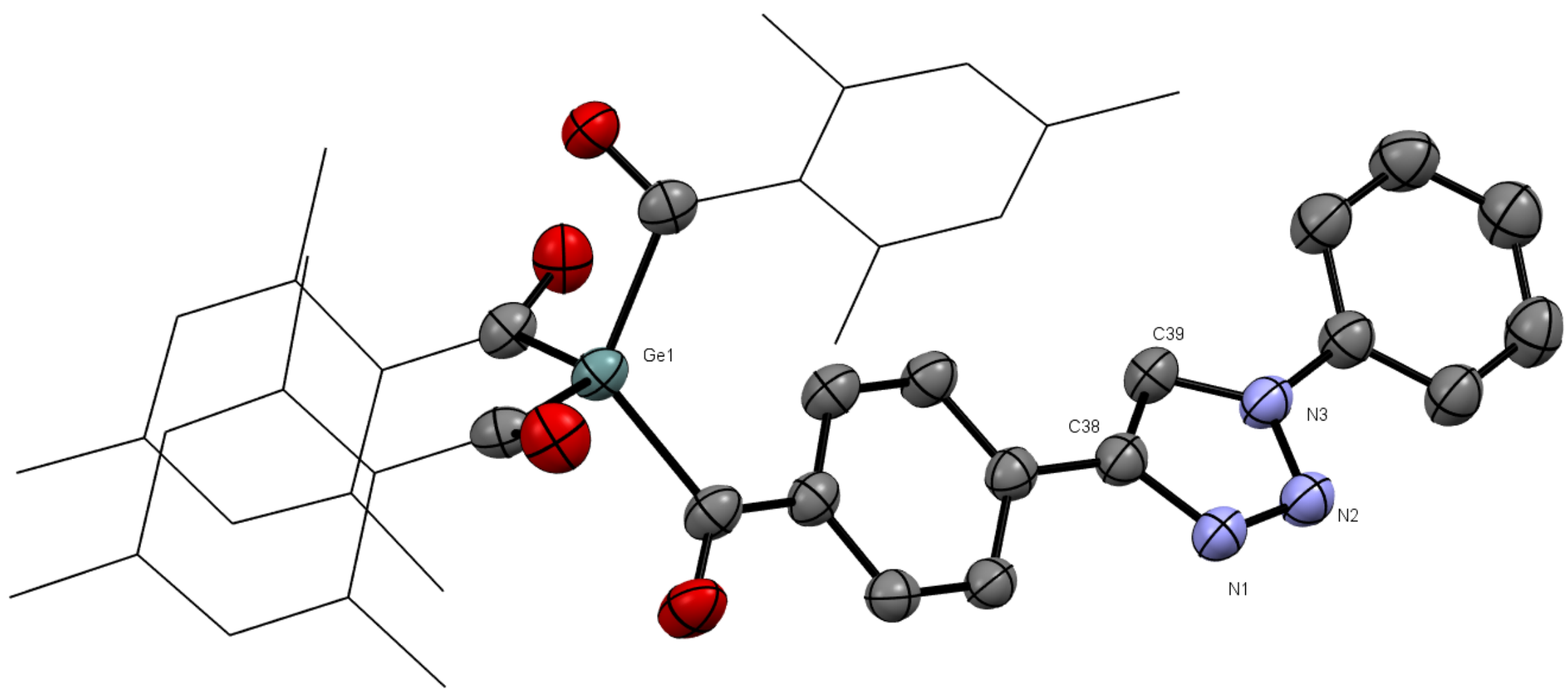

Figure S6. ORTEP representation of compound **14**. Mesityl groups are displayed in wireframe style for clarity.

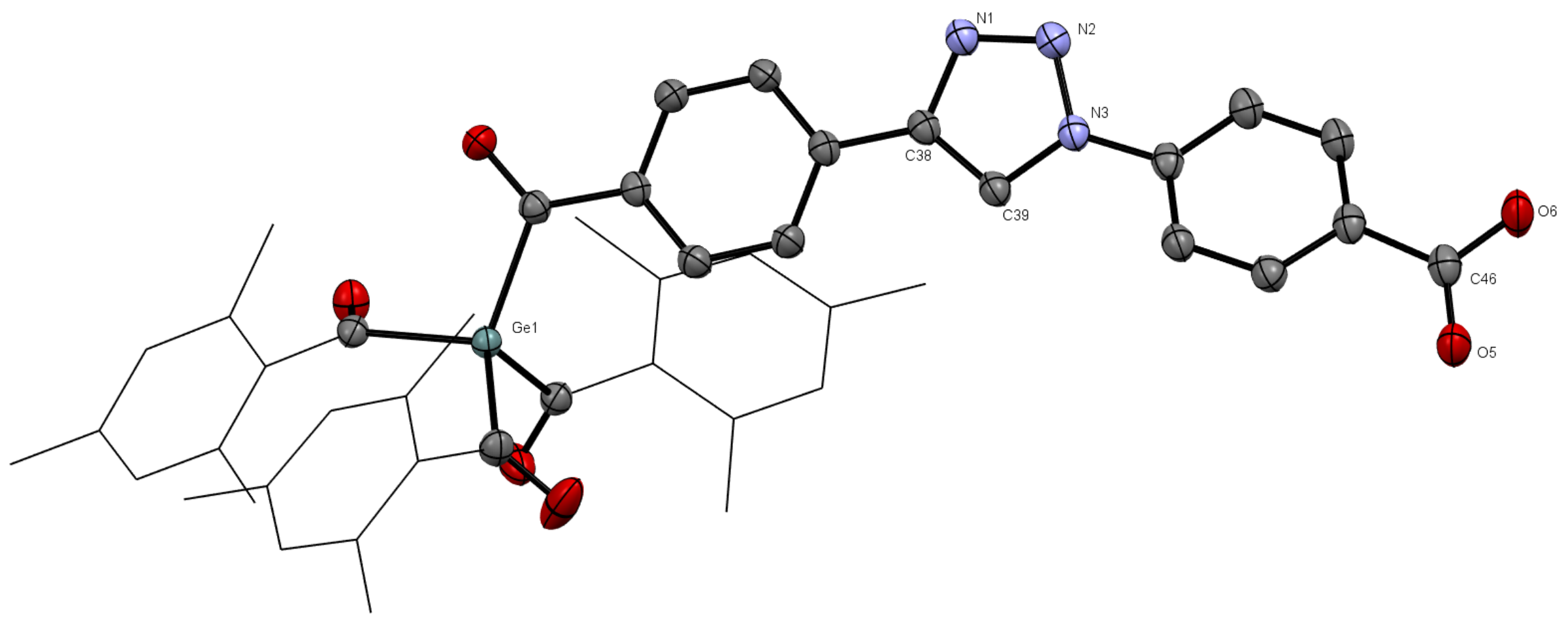

Figure S7. ORTEP representation of compound **15**. Mesityl groups are displayed in wireframe style for clarity.

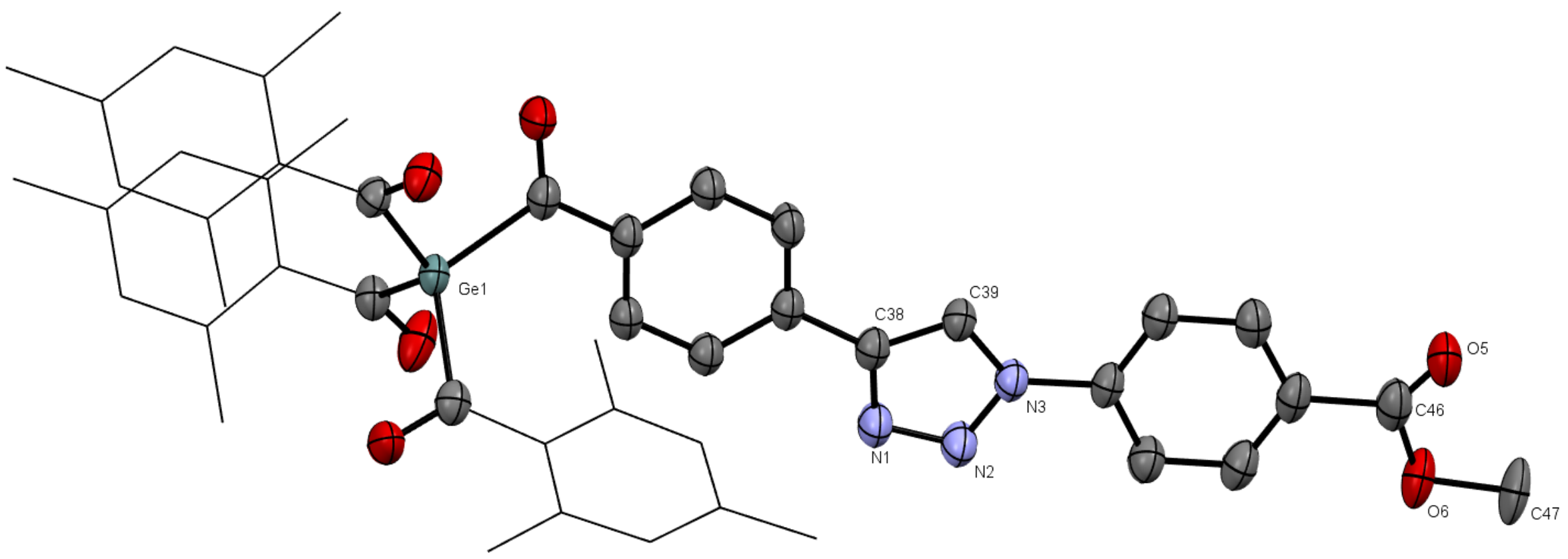

Figure S8. ORTEP representation of compound **16**. Mesityl groups are displayed in wireframe style for clarity.

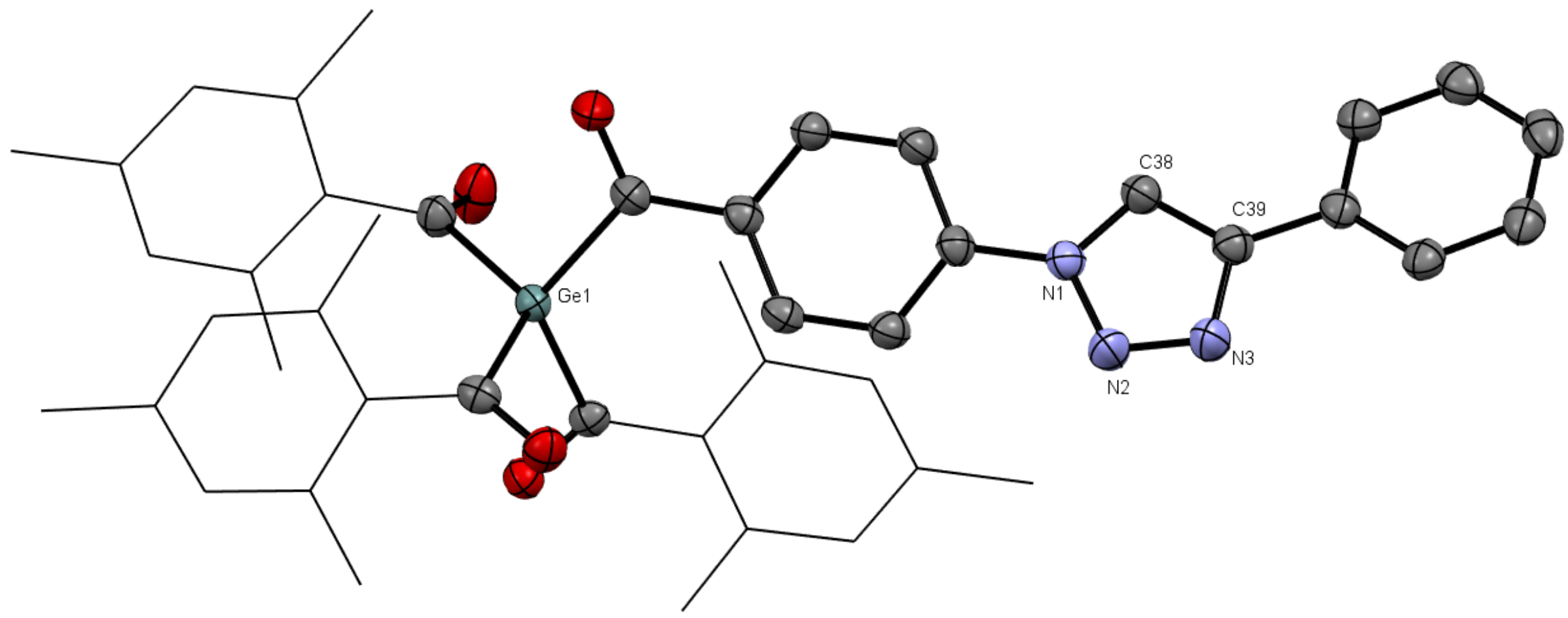

Figure S9. ORTEP representation of compound **20**. Mesityl groups are displayed in wireframe style for clarity.

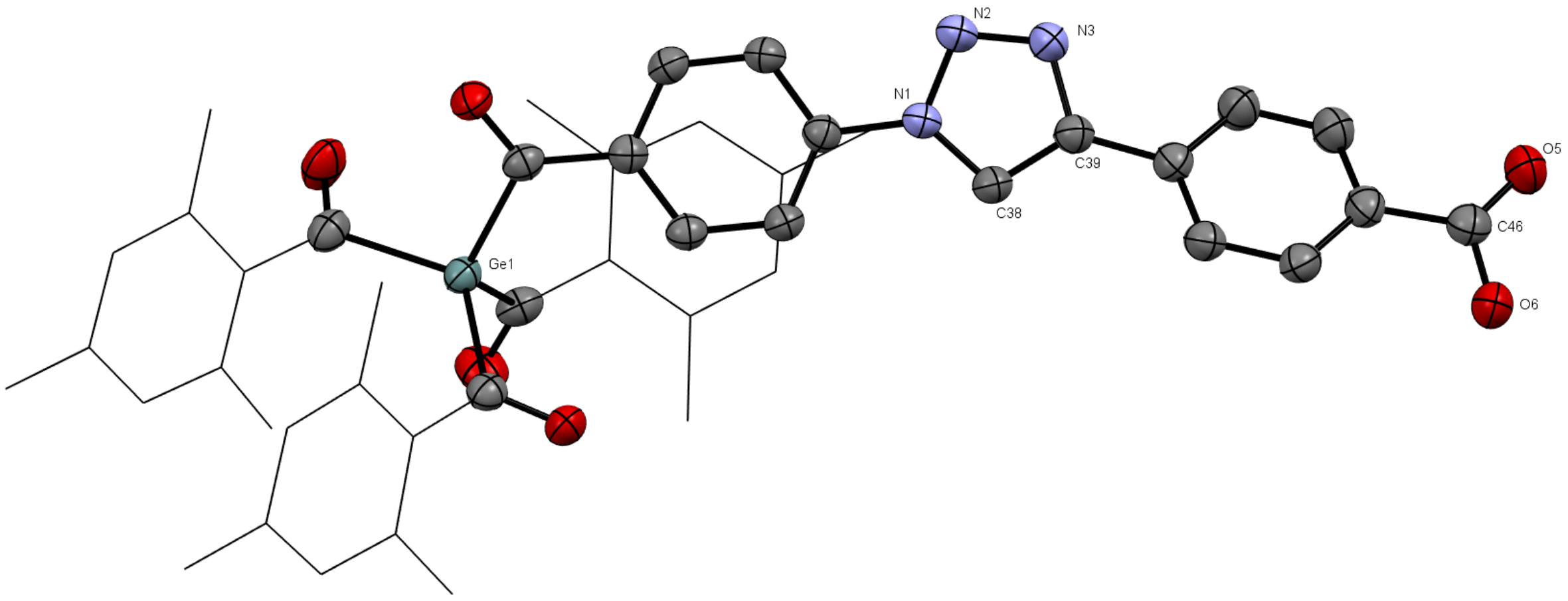

Figure S10. ORTEP representation of compound **21**. Mesityl groups are displayed in wireframe style for clarity.

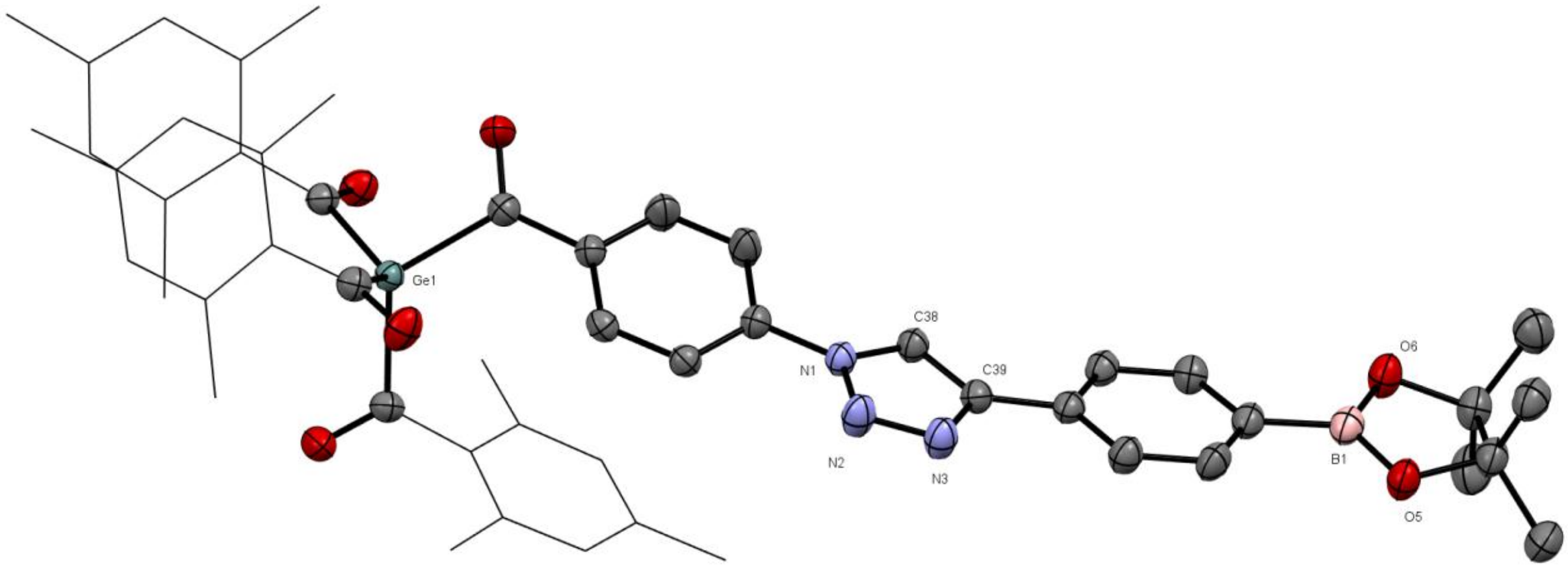

Figure S11. ORTEP representation of compound **23**. Mesityl groups are displayed in wireframe style for clarity.

Cytotoxicity

Cell culture
Human colorectal adenocarcinoma HT29 cells were used for all cytotoxicity experiments. Cells were cultured in Minimum Essential Medium (MEM; Thermo Fisher, 31095-029) supplemented with 10% fetal calf serum (FCS), 1% penicillin/streptomycin (P/S), 1% non-essential amino acids (NEAA; Gibco, 11140-035), and 1% sodium pyruvate (Gibco, 11360-039). Cells were maintained under standard cell culture conditions (37 °C, 5% CO_2, humidified chamber).

Cytotoxicity assay
Compounds were provided as 100 µM stock solutions dissolved in 99.5% water and 0.5% DMSO (10 mL each). Working concentrations of 0.1, 1, 10, and 50 µM were freshly prepared in complete MEM prior to application. Vehicle controls consisted of medium containing the corresponding percentage of DMSO without compound.

After overnight attachment, cells were treated with the four concentrations of each compound for 24 h or 48 h. Each treatment condition was performed in triplicate.

Cytotoxicity was assessed using the EZ4U proliferation and cytotoxicity assay (Biomedica, Vienna, Austria), following the manufacturer's instructions. For both 24 h and 48 h timepoints, 200 µL of fresh medium and 25 µL EZ4U reagent were added to each well, followed by a 2 h incubation. Absorbance was recorded using a Tecan SPARK multimode microplate reader (Tecan Group Ltd., Männedorf, Switzerland).

Acellular controls confirmed that the compounds did not interfere with the assay readout. Absorbance values were normalized to untreated control cells and reported as percentage viability. The experimental design ensured reliable statistical analysis, with three independent replicates ($n = 3$) performed.

All statistical analyses were carried out using GraphPad Prism (version 6.01; GraphPad Software, La Jolla, CA, USA). Group comparisons were performed using two-way ANOVA. Results were considered statistically significant at $p < 0.05$ (* $p < 0.05$, ** $p < 0.01$, *** $p < 0.001$, **** $p < 0.0001$).

Isothermal titration calorimetry (ITC)

Membrane partitioning of organogermanium compounds **17–19** was characterized by ITC using a VP-ITC instrument (MicroCal, Northampton, USA) at 25°C. Organogermanium compounds were dissolved in dimethyl sulfoxide (DMSO) and diluted with triple-distilled water to the desired concentration and a final DMSO concentration of 5% (*v*/*v*). Large unilamellar vesicles (LUVs) were prepared by hydrating the zwitterionic, singly unsaturated phospholipid 1-palmitoyl-2-oleoyl-*sn*-glycero-3-phosphocholine (POPC, from Lipoid, Ludwigshafen, Germany) in triple-distilled water containing 5% (*v*/*v*) DMSO, followed by extrusion (≥35 passes) through two stacked polycarbonate membranes with 100-nm pores (Cytiva, Freiburg, Germany) using a LiposoFast extruder (Avestin, Ottawa, Canada) at room temperature. Vesicle size was confirmed by dynamic light scattering on a Zetasizer Nano S90 (Malvern Panalytical, Malvern, UK). ITC experiments were performed by titrating POPC LUVs (10 mM or 20 mM in the syringe) into compound solutions (0.05 mM in the calorimeter cell) using 10-µL injections. Raw thermograms were integrated using NITPIC for automated baseline correction and peak integration.[71] Integrated heats were analyzed using a membrane partition equilibrium model implemented in Microsoft Excel, as described previously.[72-73] The intrinsic partition coefficient (K_0) and the standard molar enthalpy of transfer ($\Delta H°$) were treated as adjustable parameters, while the lipid accessibility factor (γ) was set to 0.5, assuming that these amphiphilic organogermanium compounds do not permeate the lipid bilayer on the ITC timescale.[72-73] All experiments were performed in duplicate, and 95% confidence intervals were determined from sum-of-squared-residual (SSR) projections as detailed elsewhere.[74]

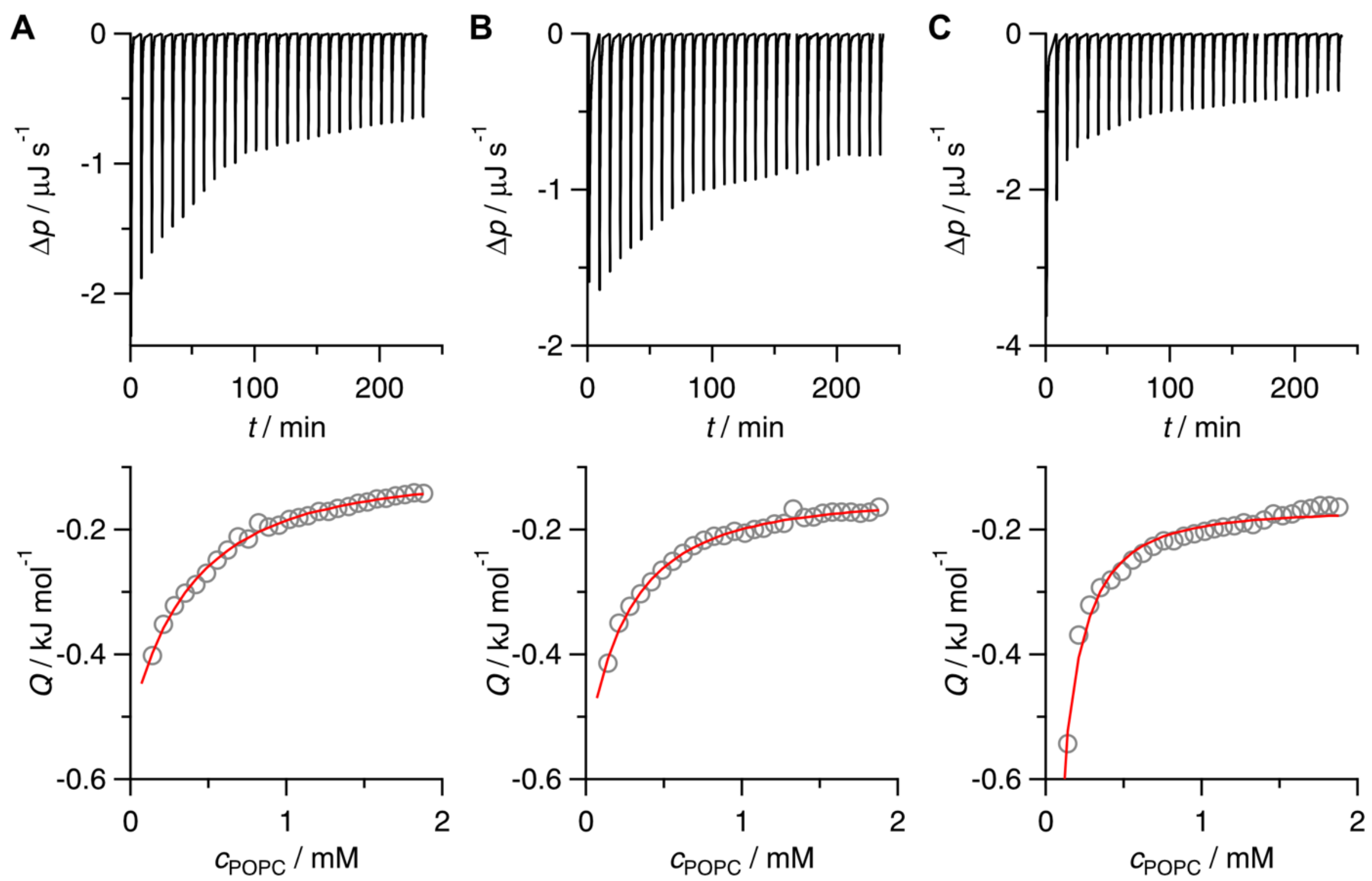

Figure S12. Membrane partitioning of organogermanium compounds characterized by ITC. (A) **18**, (B) **17**, (C) **19**. Upper panels: raw thermograms showing differential heating power (Δp) versus time (t) upon titration of POPC LUVs into compound solutions. Lower panels: integrated heats of reaction (Q) as a function of lipid concentration (c_{POPC}). Open circles represent experimental data; solid lines are best

fits to a membrane partition equilibrium model.[72-73] Experiments were performed at 25°C in 95/5 (*v*/*v*) H_2O/DMSO mixtures with 0.05 mM compound in the cell and 20 mM POPC LUVs in the syringe.

Intrinsic partition coefficients (K_0) and standard molar transfer enthalpies ($\Delta H°$) were obtained from ITC experiments by nonlinear least-squares fitting to a membrane partition model;[72-73] values in brackets denote 95% confidence intervals determined from SSR projections.[74] Standard molar Gibbs energies were calculated as $\Delta G° = -RT \ln K_0$, with confidence intervals propagated from K_0. Entropic contributions were obtained from the Gibbs–Helmholtz relation, $-T\Delta S° = \Delta G° - \Delta H°$; confidence intervals for $-T\Delta S°$ were estimated conservatively by combining the extreme bounds of $\Delta G°$ and $\Delta H°$, assuming parameter independence. Because K_0 and $\Delta H°$ are typically positively correlated in ITC experiments, the true uncertainties in $-T\Delta S°$ are likely narrower than reported. Experiments were performed at 25°C (298.15 K) in 95/5 (*v*/*v*) H_2O/DMSO mixtures.

NMR Spectroscopy

Prop-2-yn-1-yltris(2,4,6-trimethylbenzoyl)germane (1)

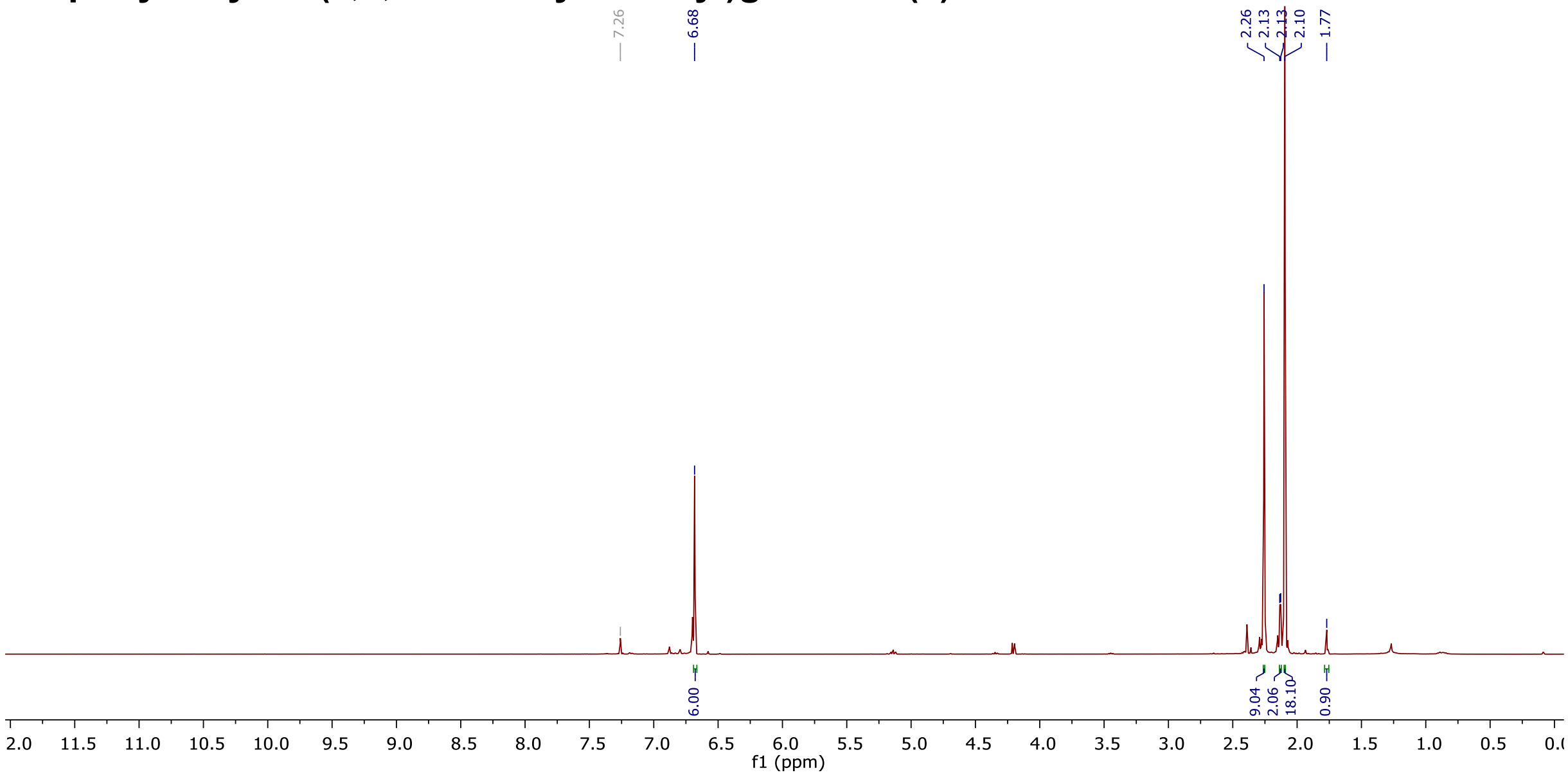

Figure S13. ^{1}H NMR of compound **1** in $CDCl_3$.

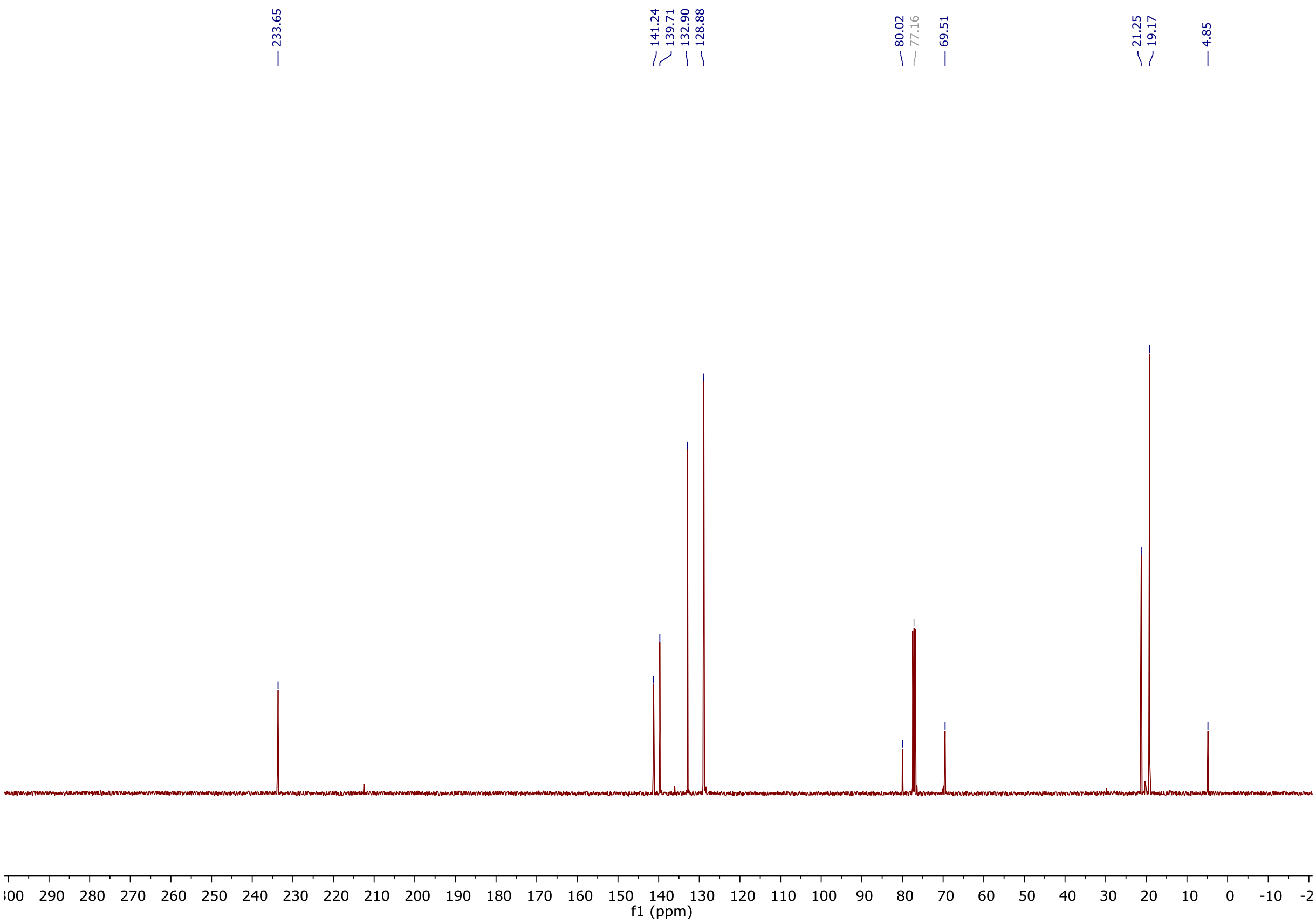

Figure S14. ^{13}C NMR of compound **1** in $CDCl_3$.

Tris(2,4,6-trimethylbenzoyl)(4-ethynylbenzoyl)germane (2)

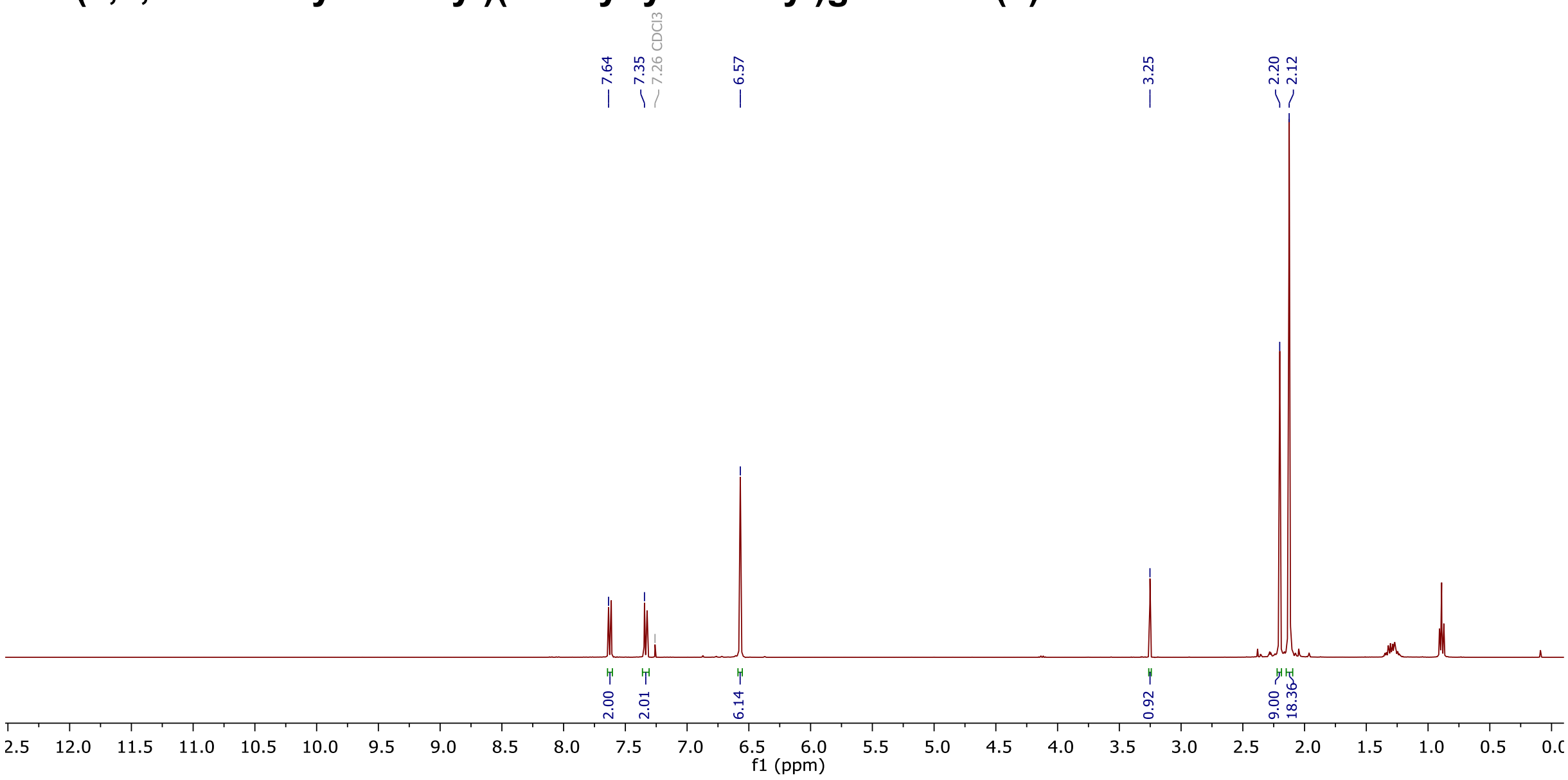

Figure S15. ^{1}H NMR of compound **2** in $CDCl_3$.

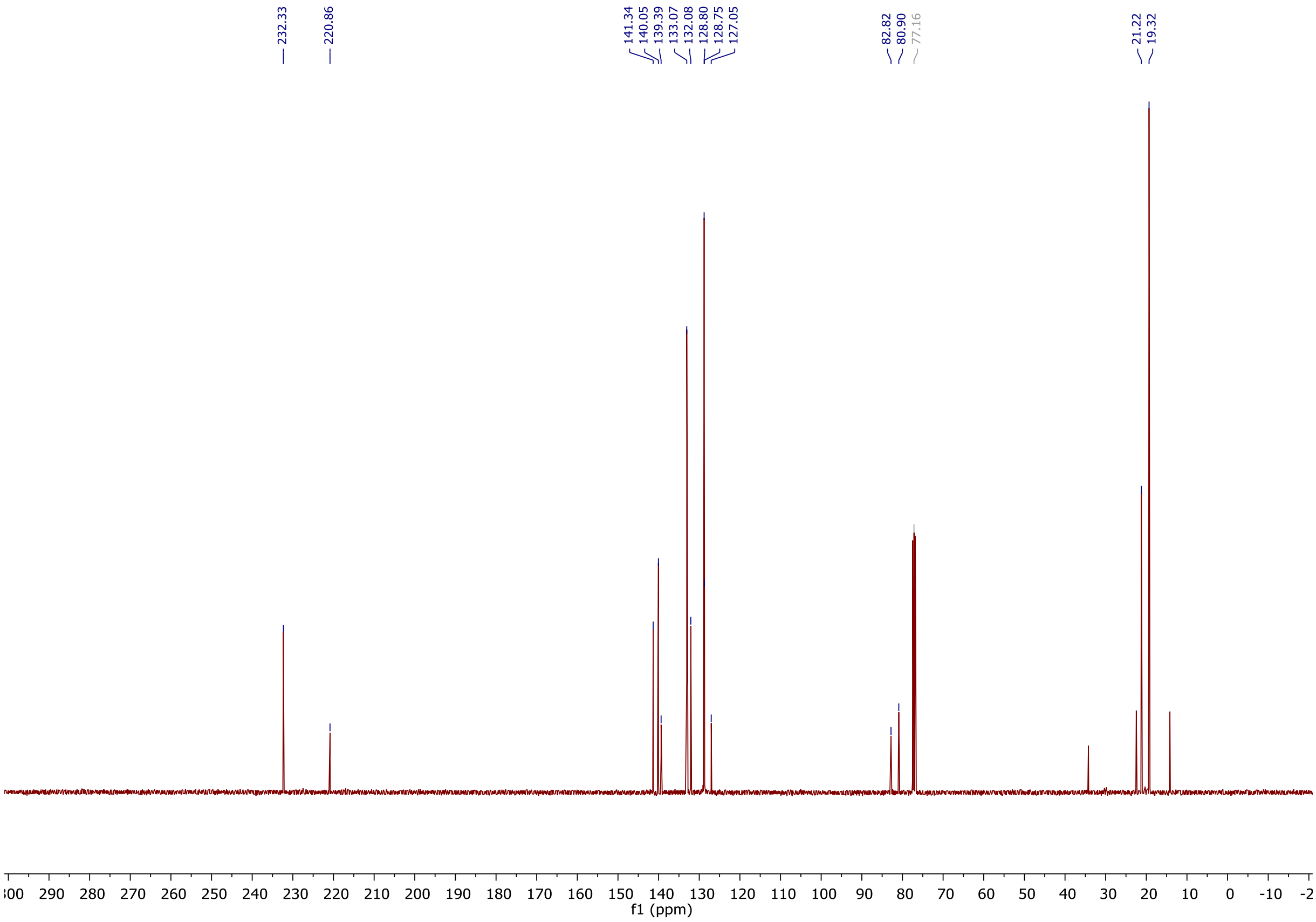

Figure S16. ^{13}C NMR of compound **2** in $CDCl_3$.

Tris(2,4,6-trimethylbenzoyl)(hex-5-ynoyl)germane (3)

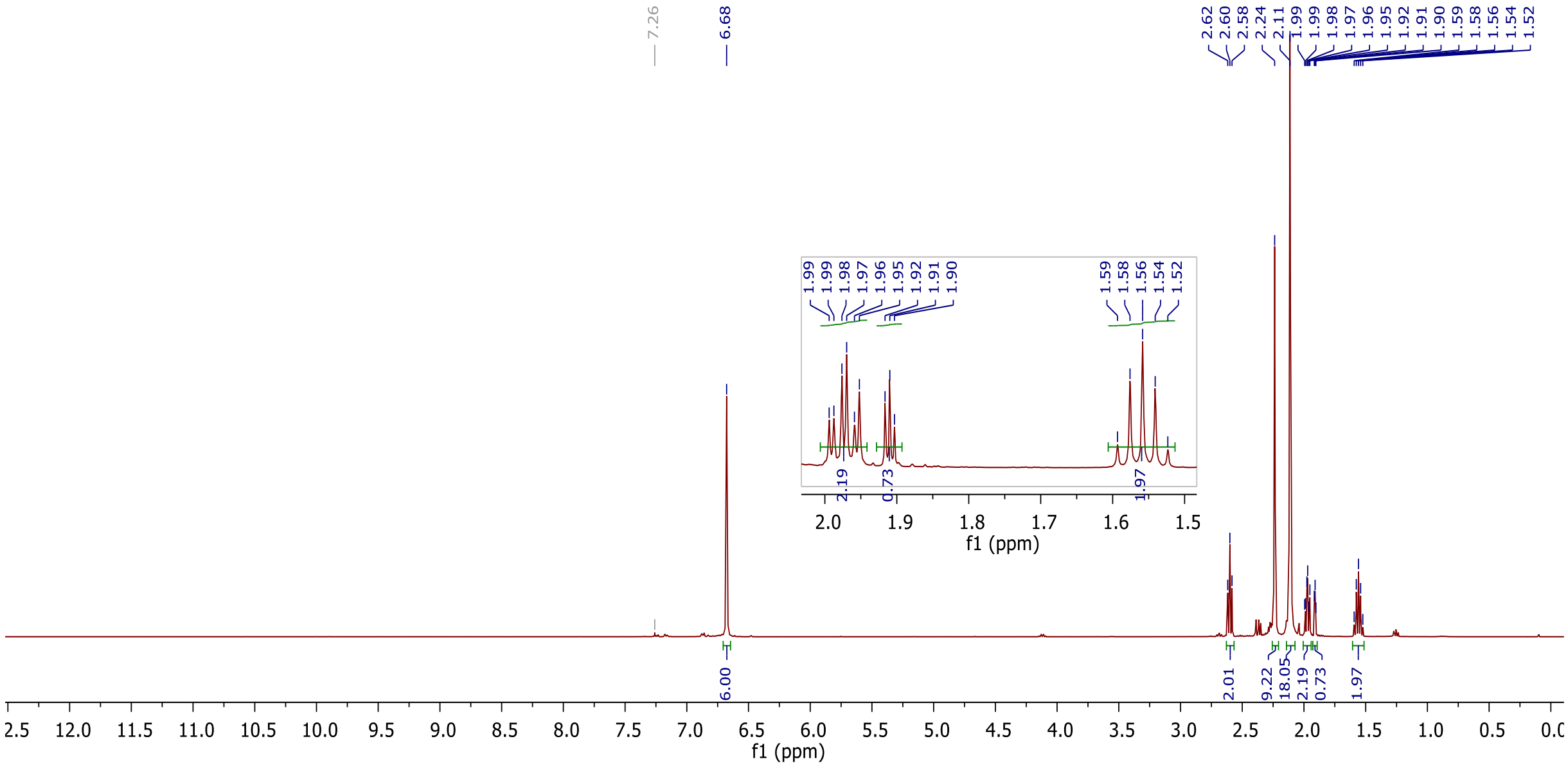

Figure S17. ^{1}H NMR of compound **3** in $CDCl_3$.

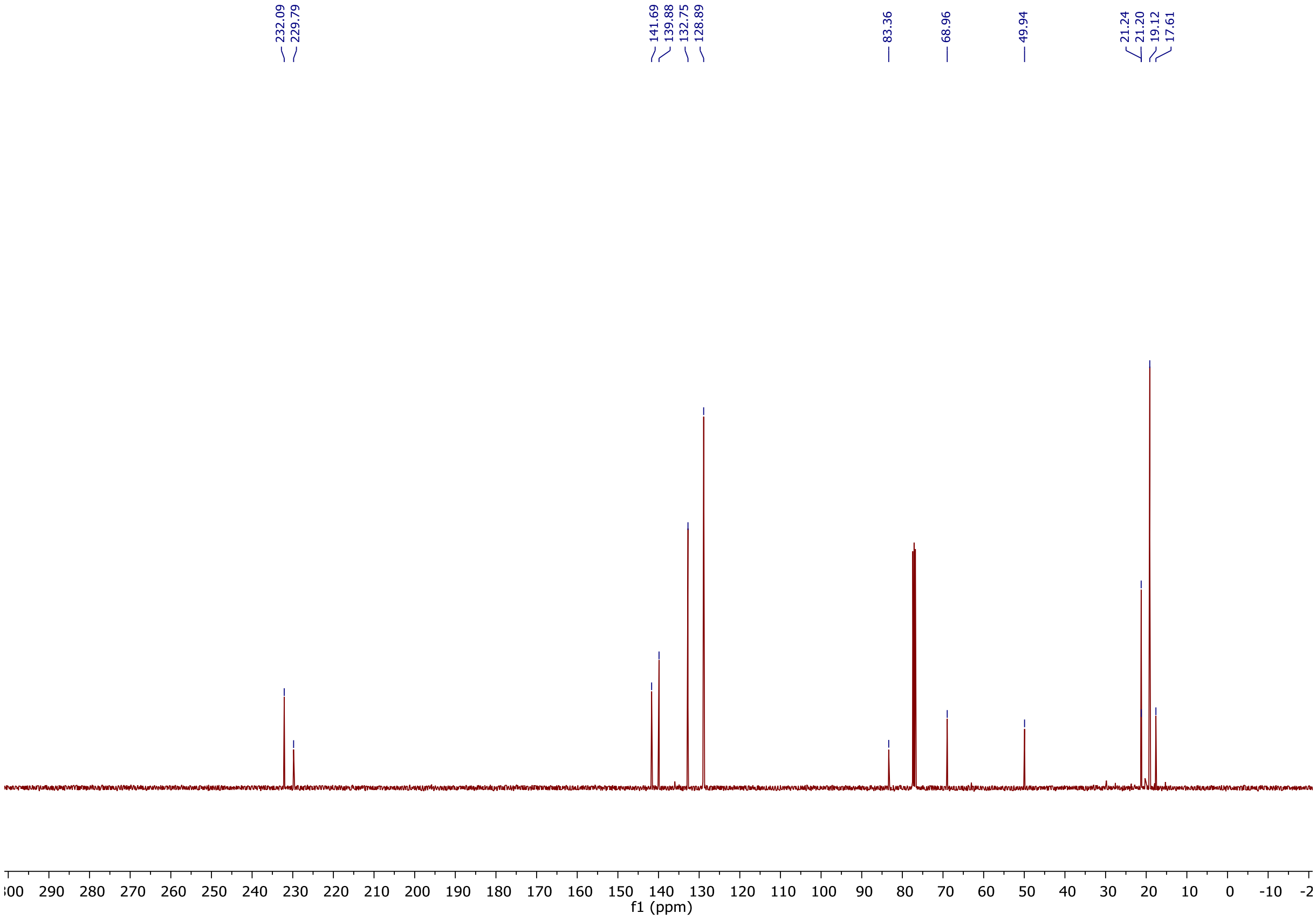

Figure S18. ^{13}C NMR of compound **3** in $CDCl_3$.

Tris(2,4,6-trimethylbenzoyl)(4-azidobenzoyl)germane (4)

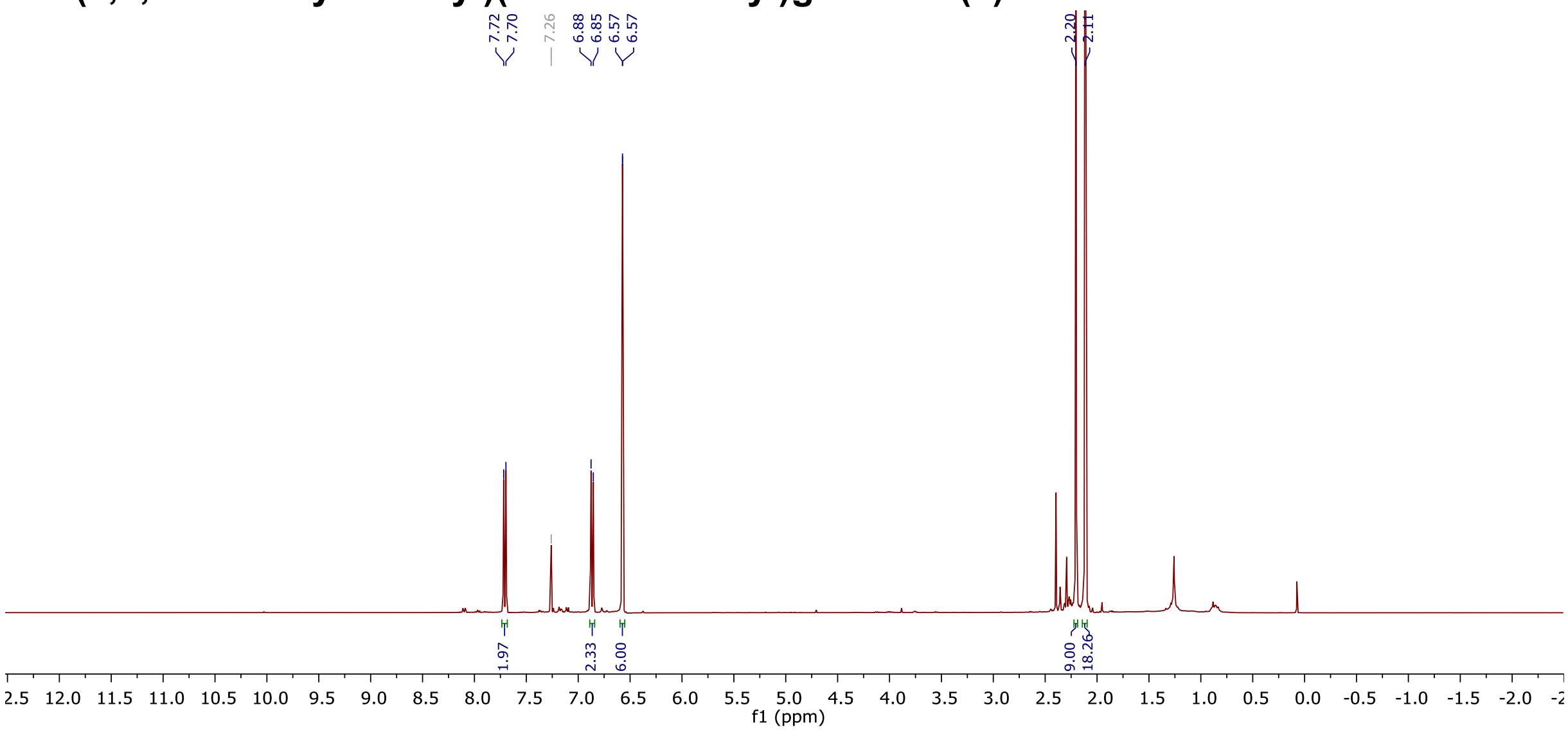

Figure S19. ^{1}H NMR of compound **4** in $CDCl_3$.

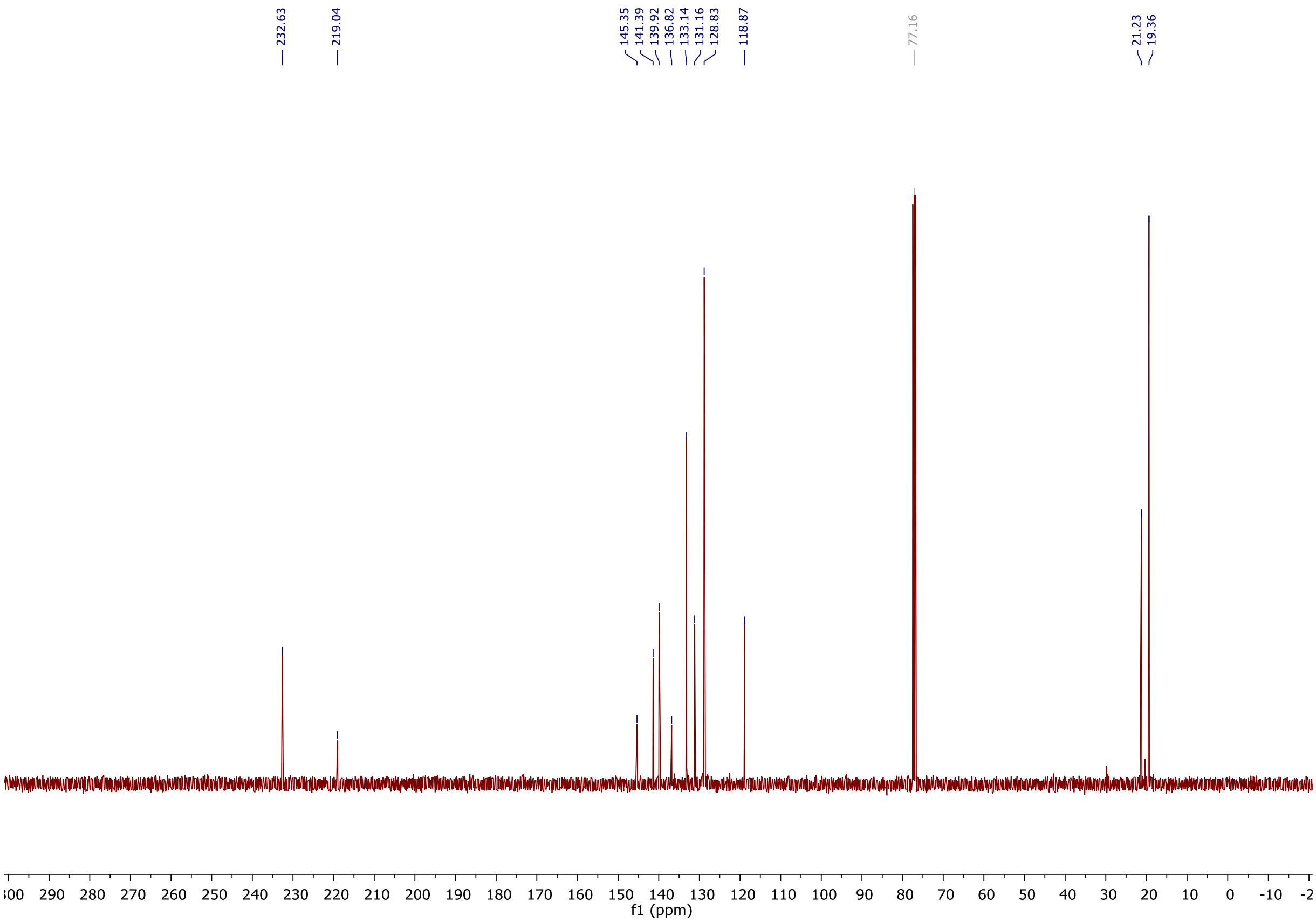

Figure S20. ^{13}C NMR of compound **4** in $CDCl_3$.

Tris(2,4,6-trimethylbenzoyl)(4-(1-phenyl-1,2,3-triazol-4-yl)benzoyl)germane (5)

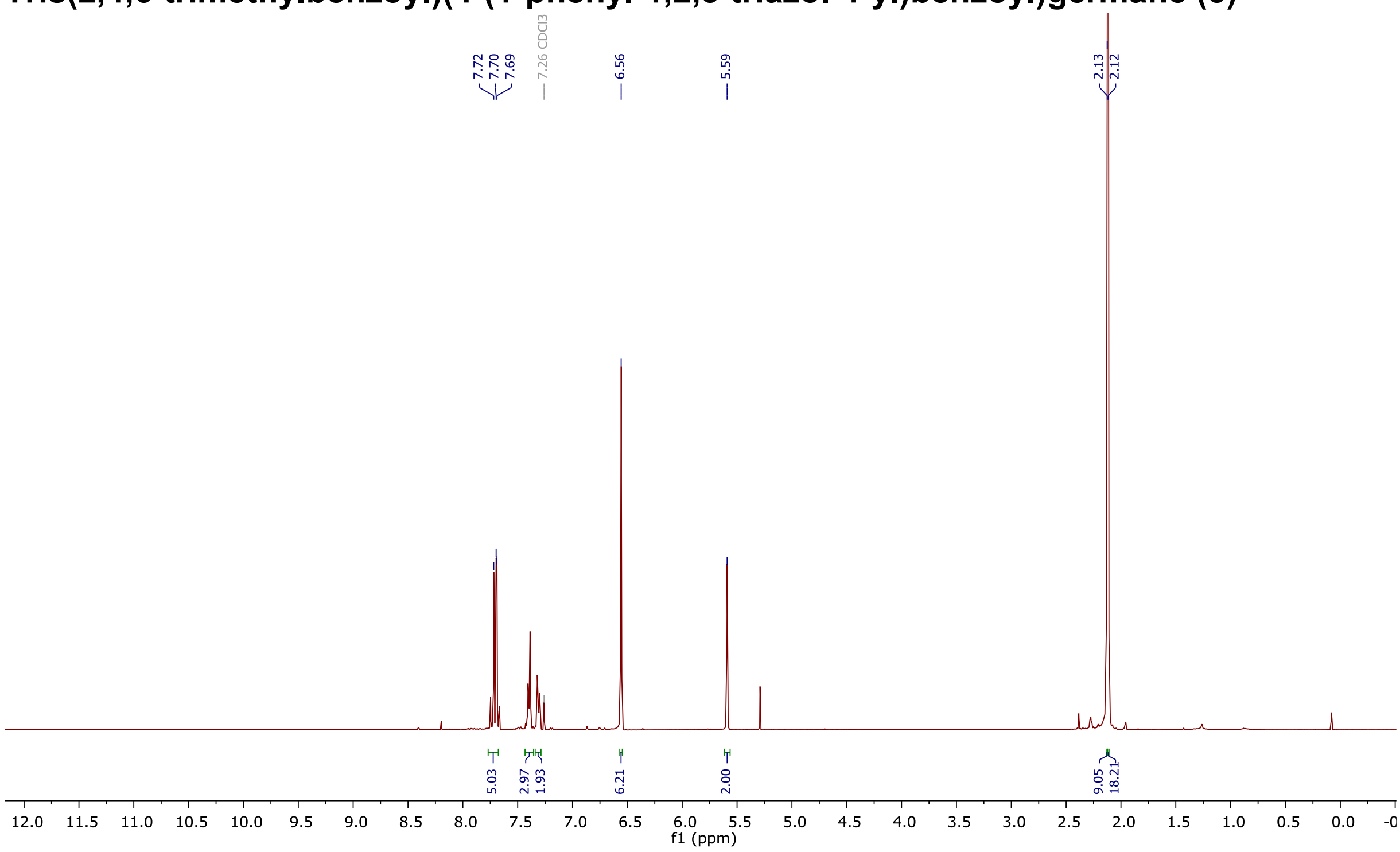

Figure S21. ^{1}H NMR of compound **5** in $CDCl_3$.

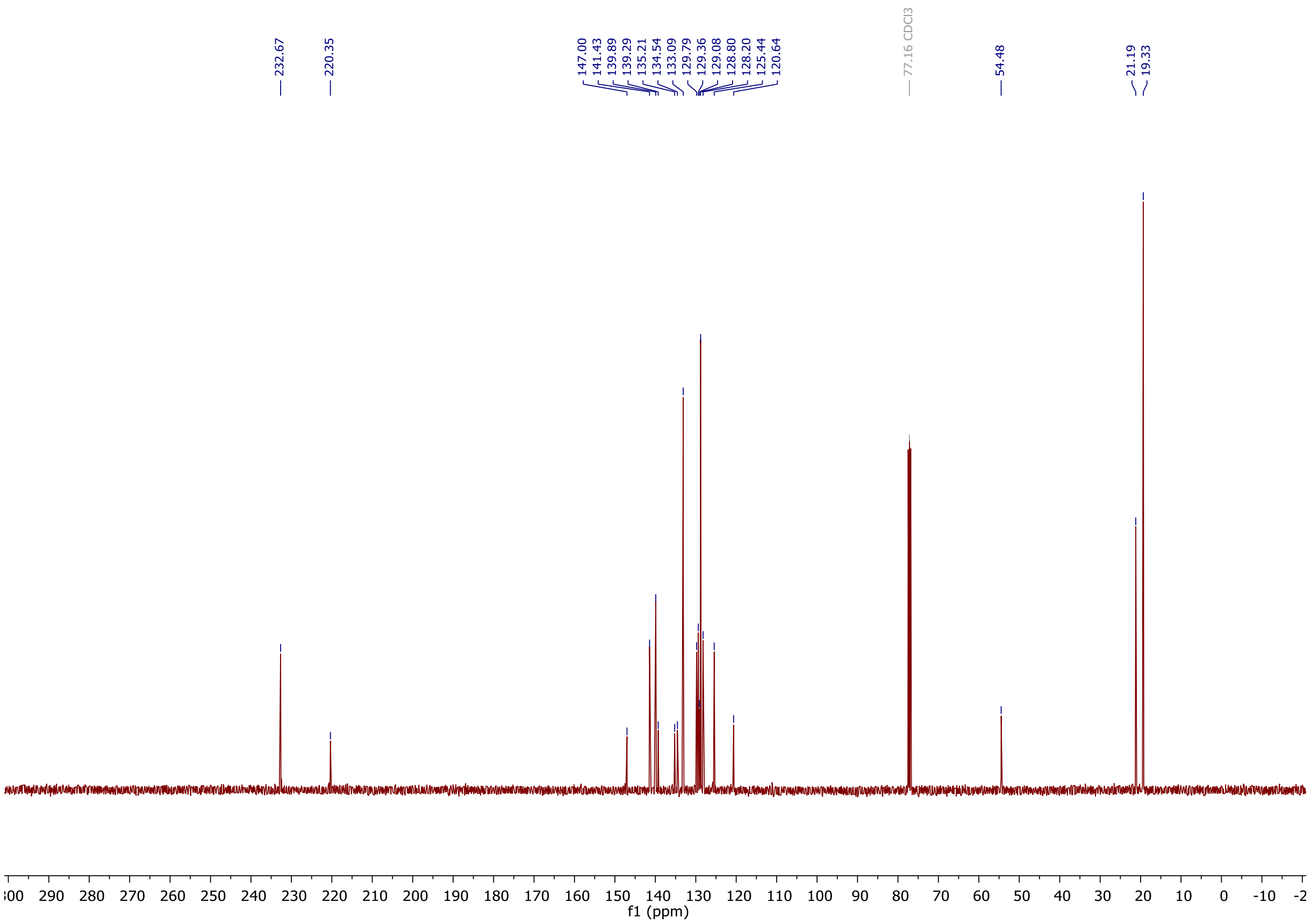

Figure S22. ^{13}C NMR of compound **5** in $CDCl_3$.

Tris(2,4,6-trimethylbenzoyl)(1-(1-benzyl-1,2,3-triazol-4-yl)methyl)germane (6)

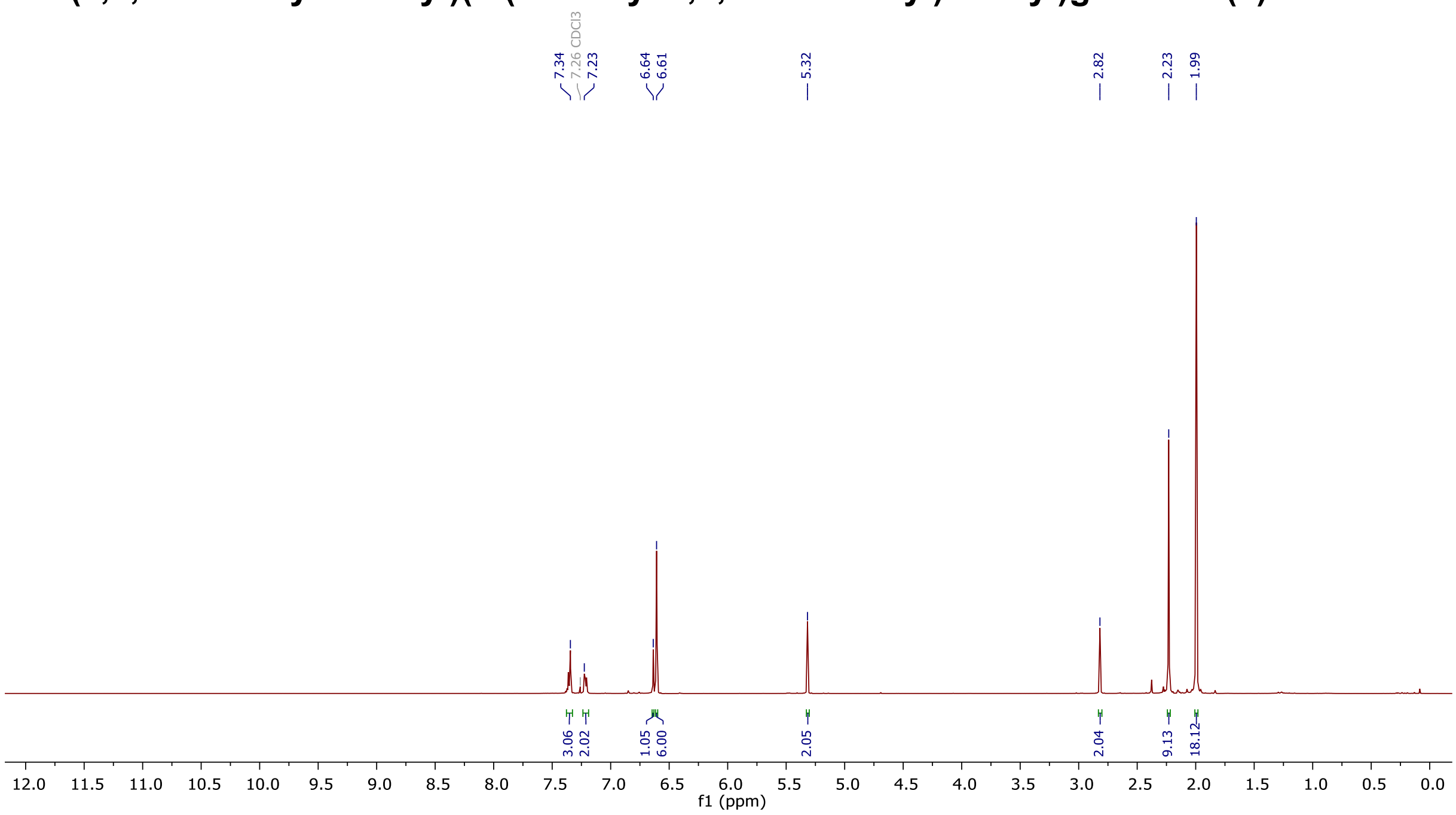

Figure S23. ^{1}H NMR of compound **6** in $CDCl_3$.

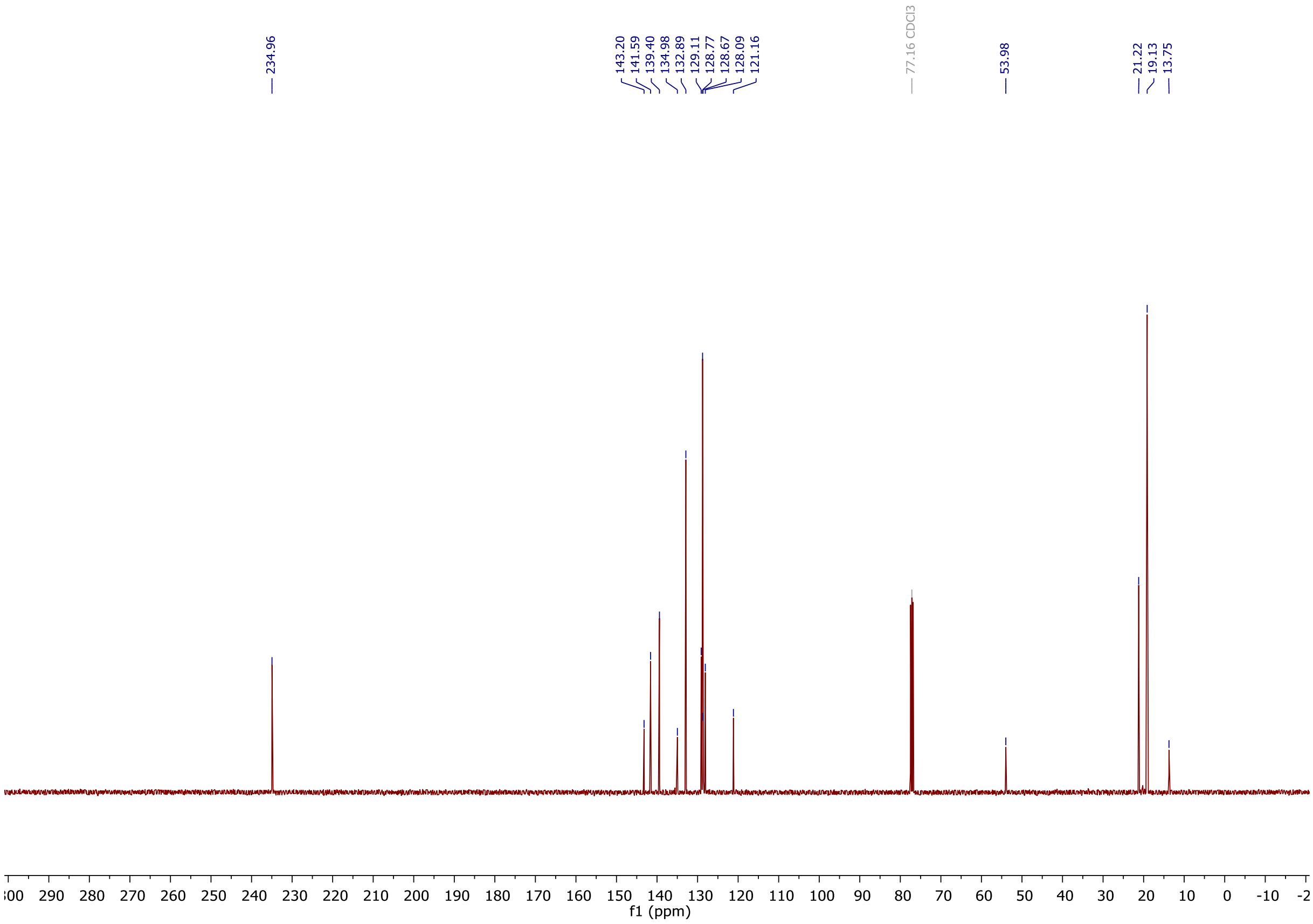

Figure S24. ^{13}C NMR of compound **6** in $CDCl_3$.

Tris(2,4,6-trimethylbenzoyl)(4-(1-benzyl-1,2,3-triazol-4-yl)butanoyl)germane (7)

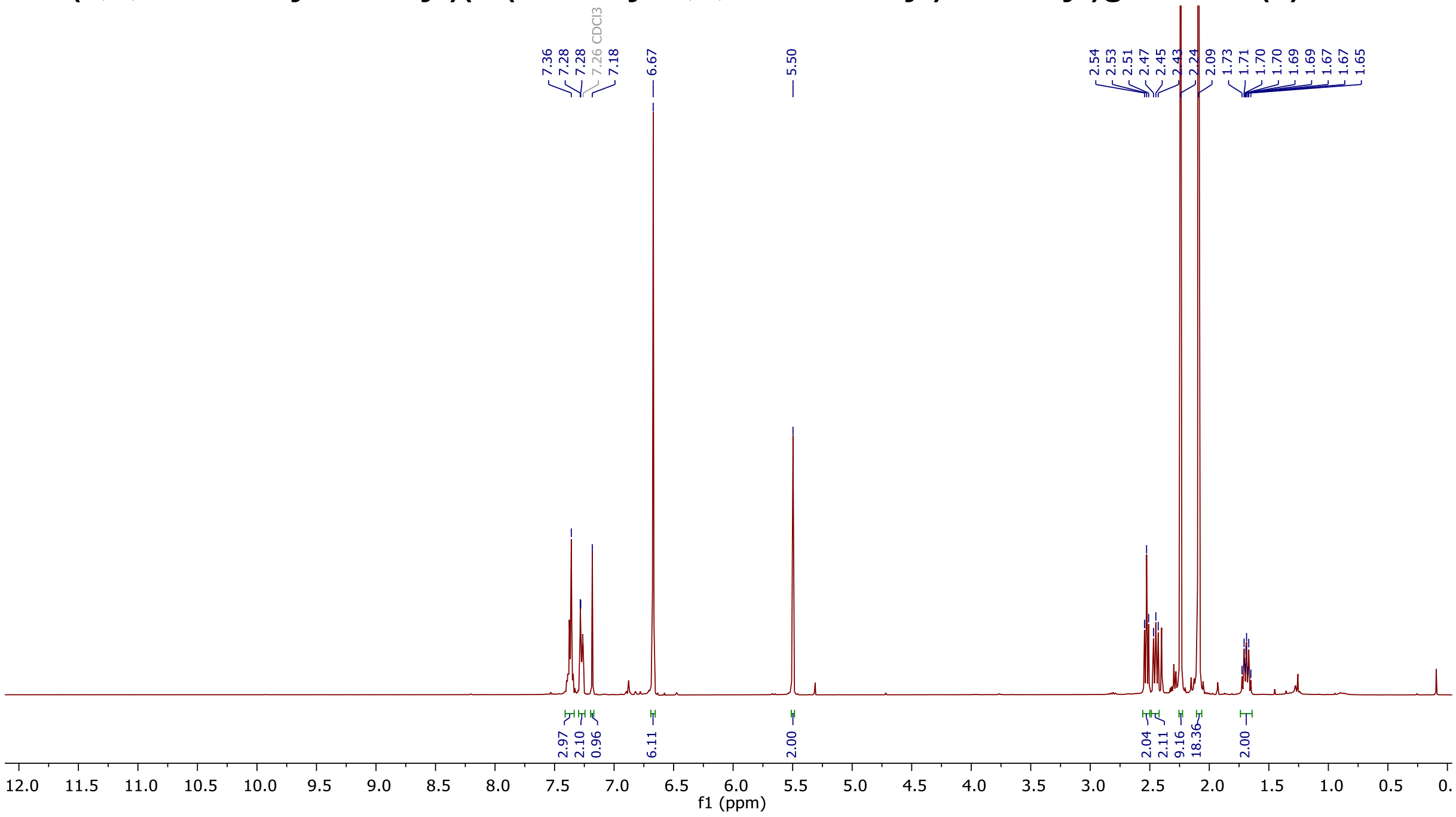

Figure S25. ^{1}H NMR of compound **7** in $CDCl_3$.

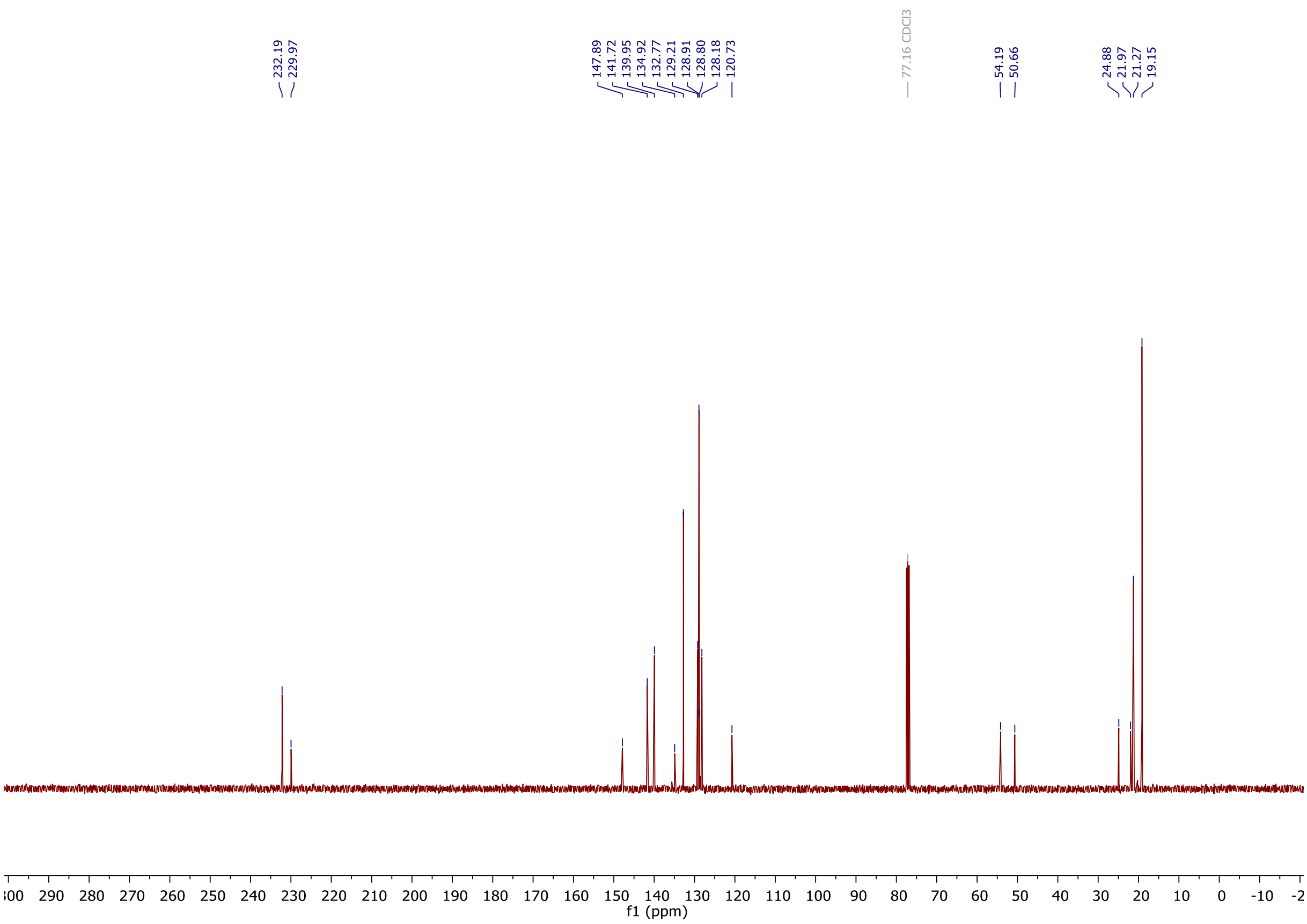

Figure S26. ^{13}C NMR of compound **7** in $CDCl_3$.

Tris(2,4,6-trimethylbenzoyl)(4-(1-(4-methylbenzyl)-1,2,3-triazol-4-yl)benzoyl)germane (8)

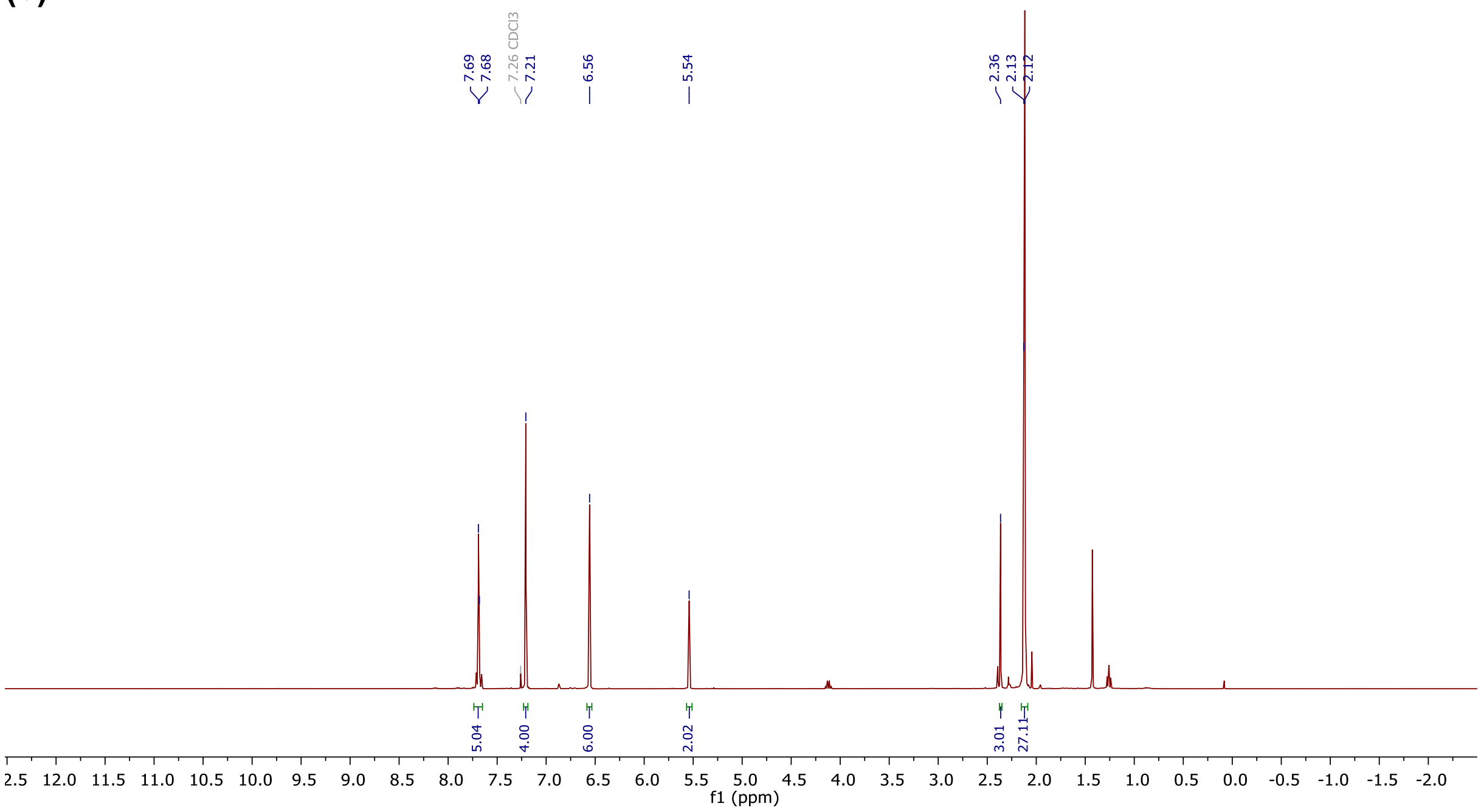

Figure S27. ^{1}H NMR of compound **8** in $CDCl_3$.

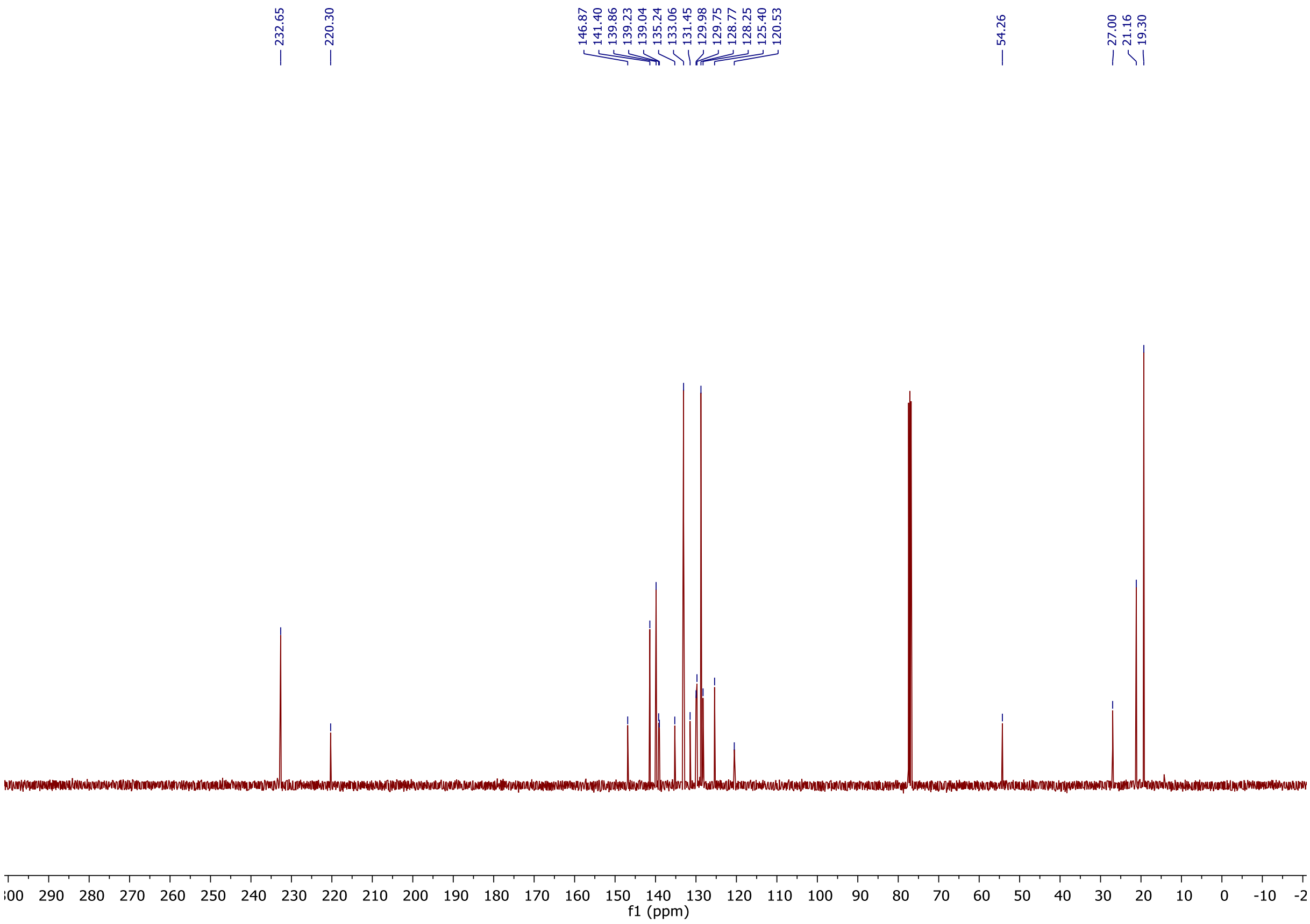

Figure S28. ^{13}C NMR of compound **8** in $CDCl_3$.

Tris(2,4,6-trimethylbenzoyl)(4-(1-(2-nitrobenzyl)-1,2,3-triazol-4-yl)benzoyl)germane (9)

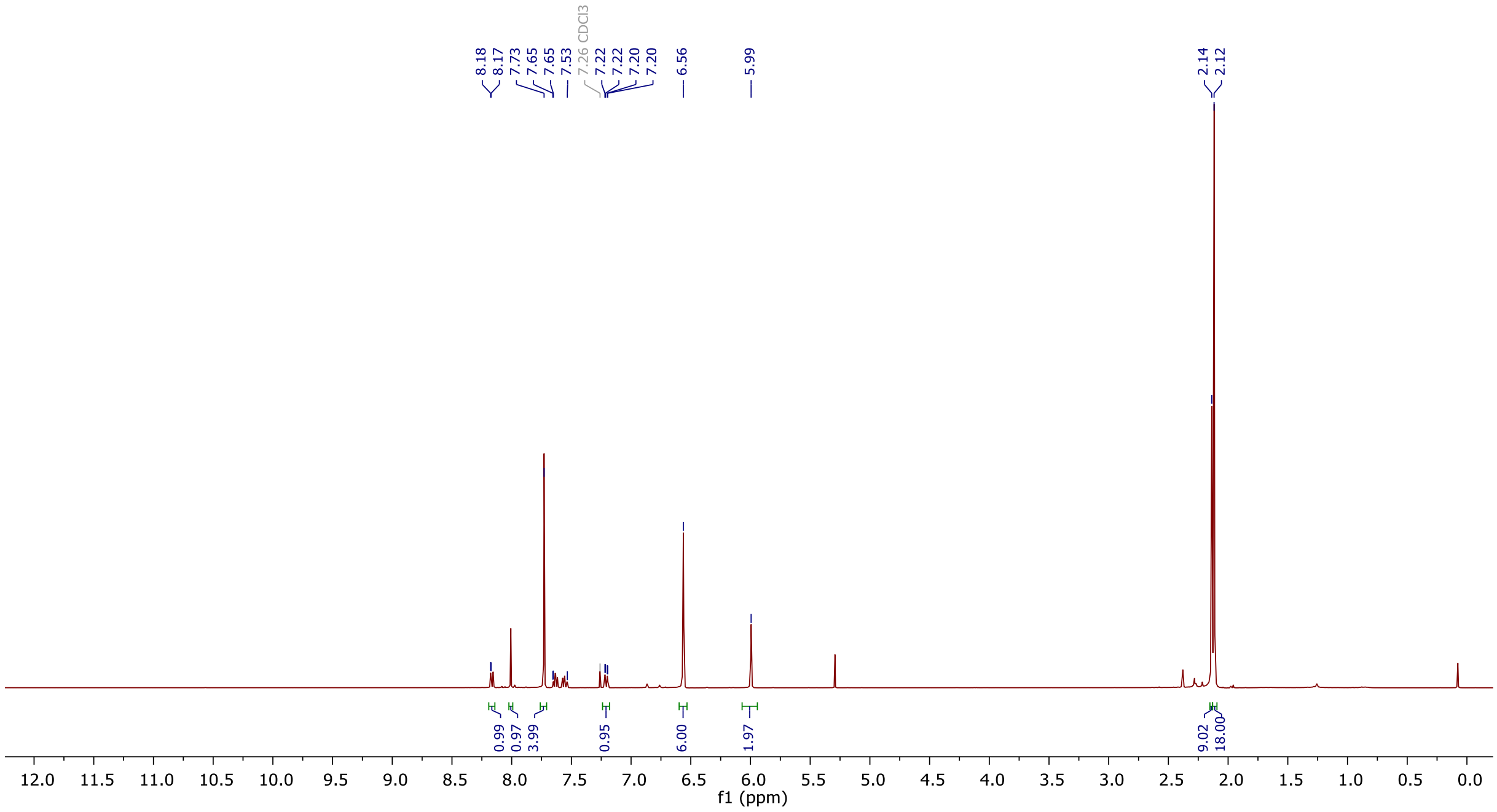

Figure S29. ^{1}H NMR of compound **9** in $CDCl_3$.

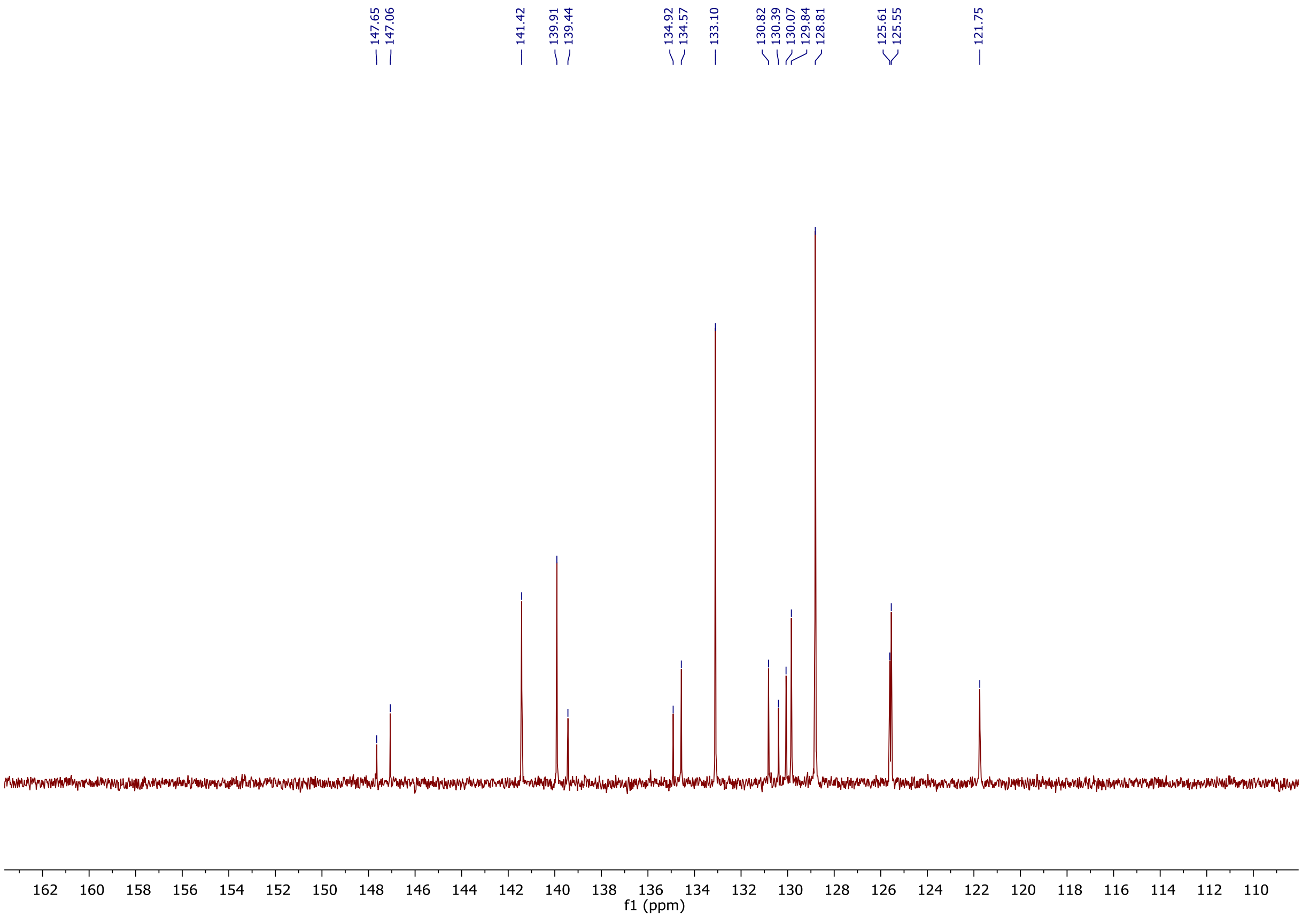

Figure S30. ^{13}C NMR of compound **9** in $CDCl_3$.

Tris(2,4,6-trimethylbenzoyl)(4-(1-(4-(dimethylamino)benzyl)-1,2,3-triazol-4-yl)benzoyl)germane (10)

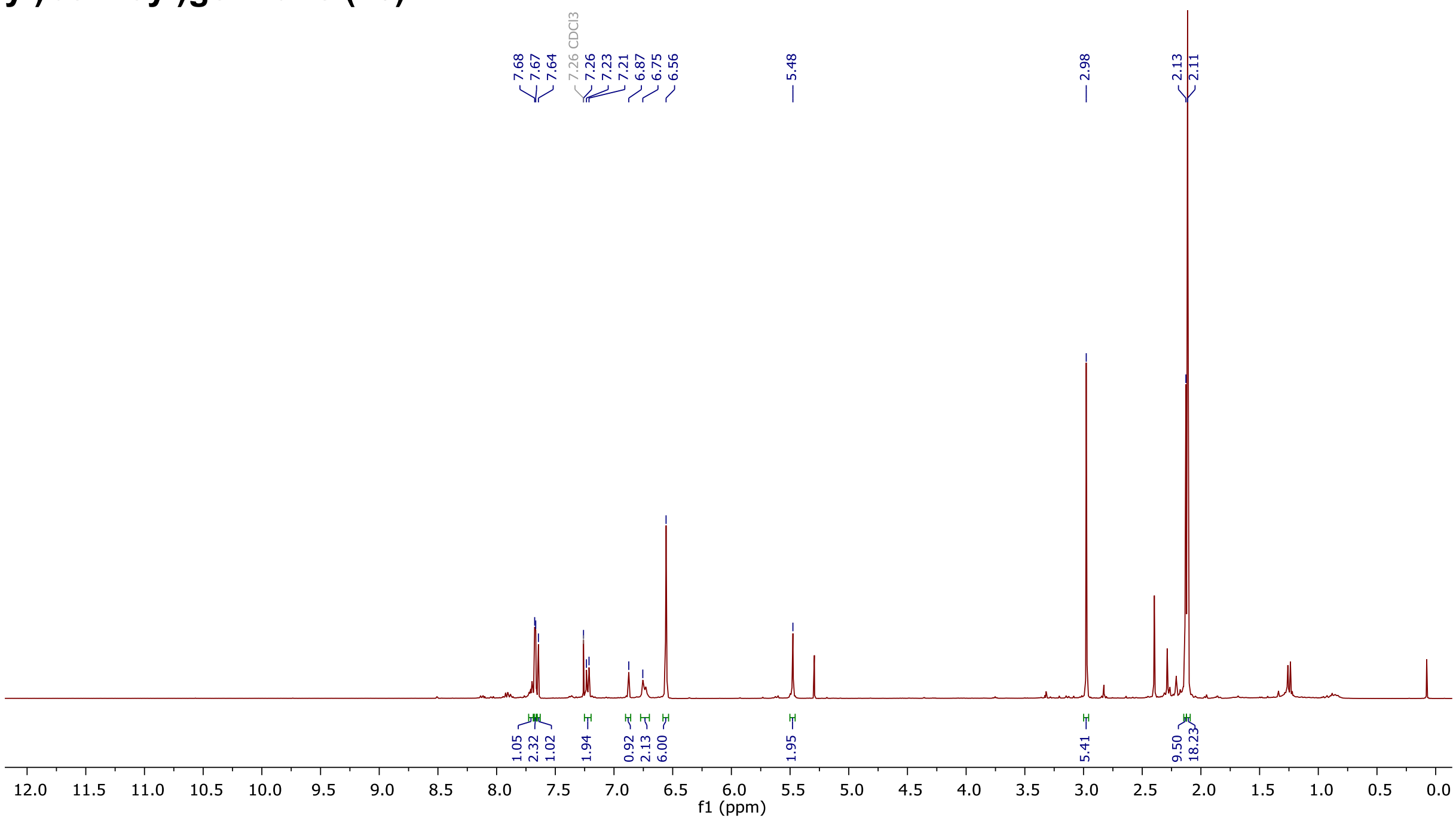

Figure S31. ^{1}H NMR of compound **10** in $CDCl_3$.

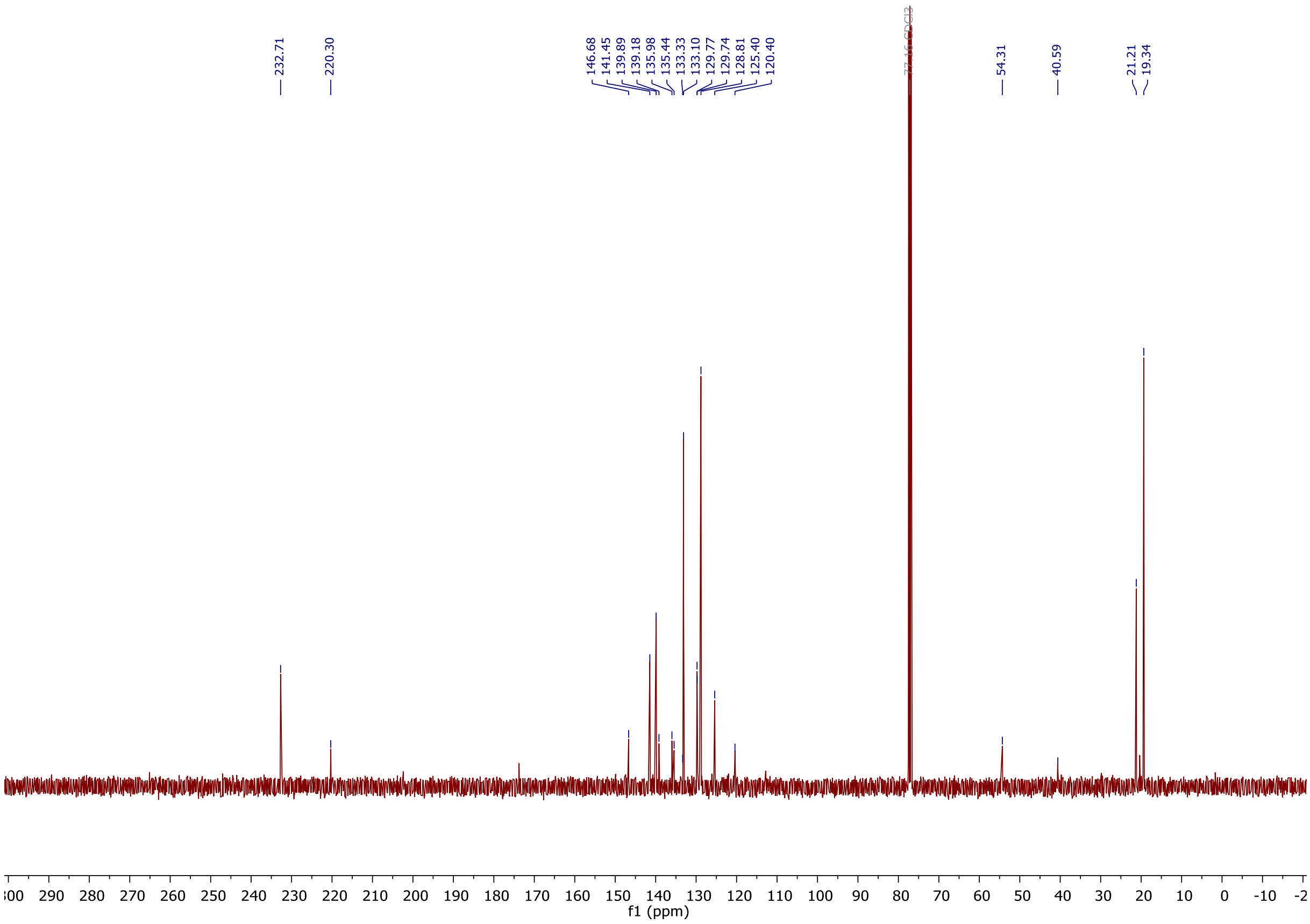

Figure S32. ^{13}C NMR of compound **10** in $CDCl_3$.

Tris(2,4,6-trimethylbenzoyl)(4-(1-(6-(methoxycarbonyl)hexyl)-1,2,3-triazol-4-yl)benzoyl)germane (11)

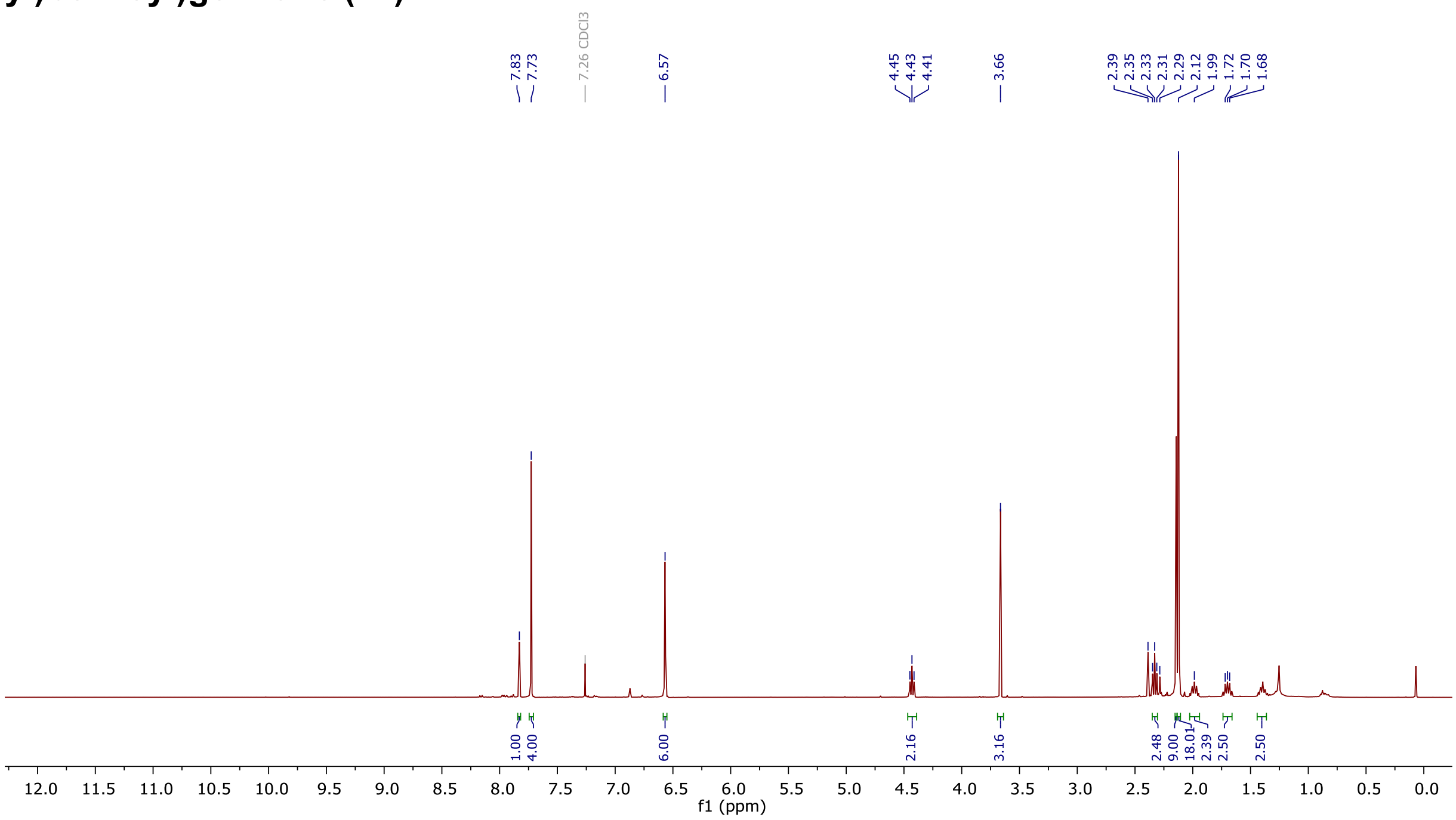

Figure S33. ^{1}H NMR of compound **11** in $CDCl_3$.

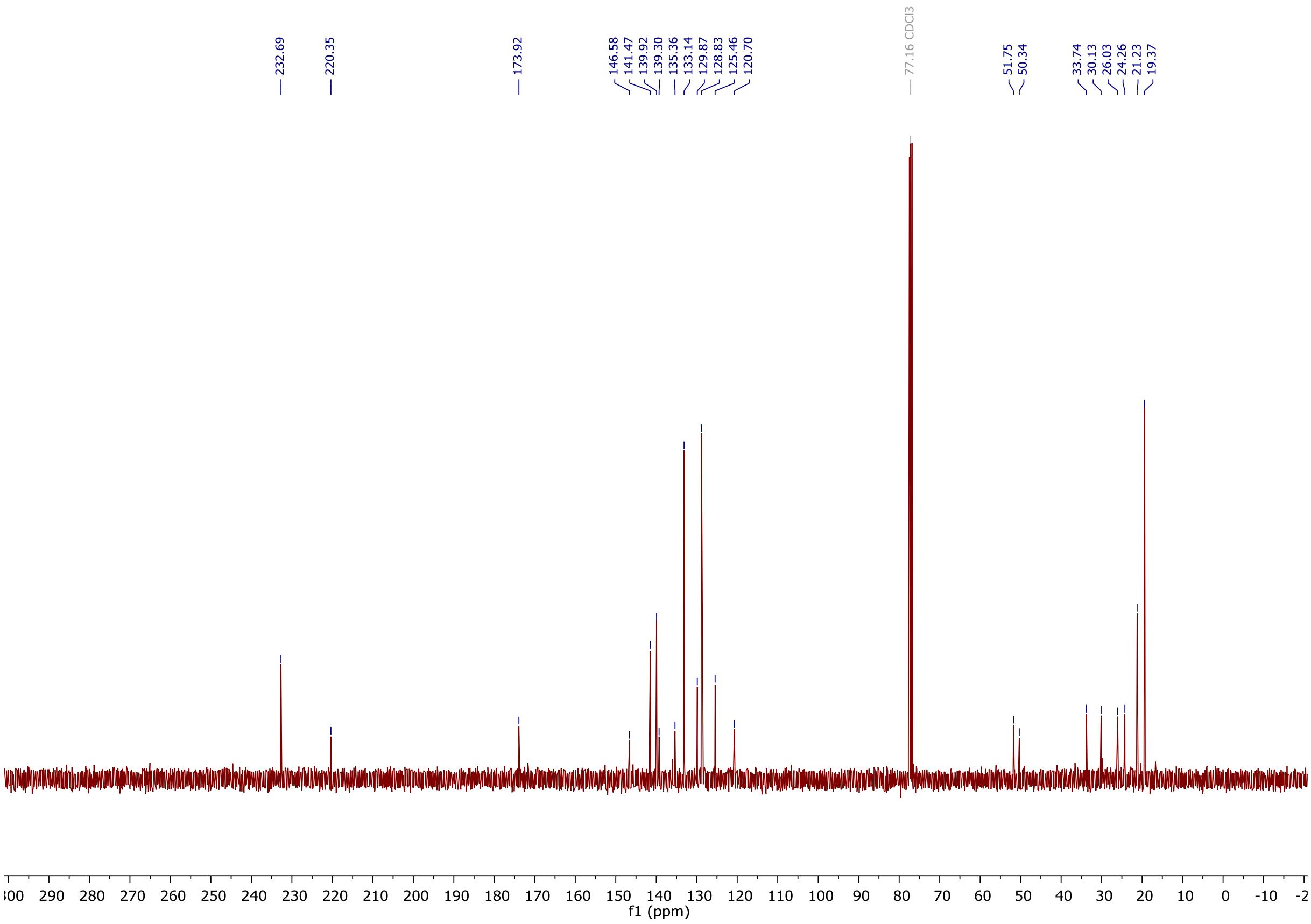

Figure S34. ^{13}C NMR of compound **11** in $CDCl_3$.

Tris(2,4,6-trimethylbenzoyl)(1-(1-(6-(methoxycarbonyl)hexyl)-1,2,3-triazol-4-yl)methyl)germane (12)

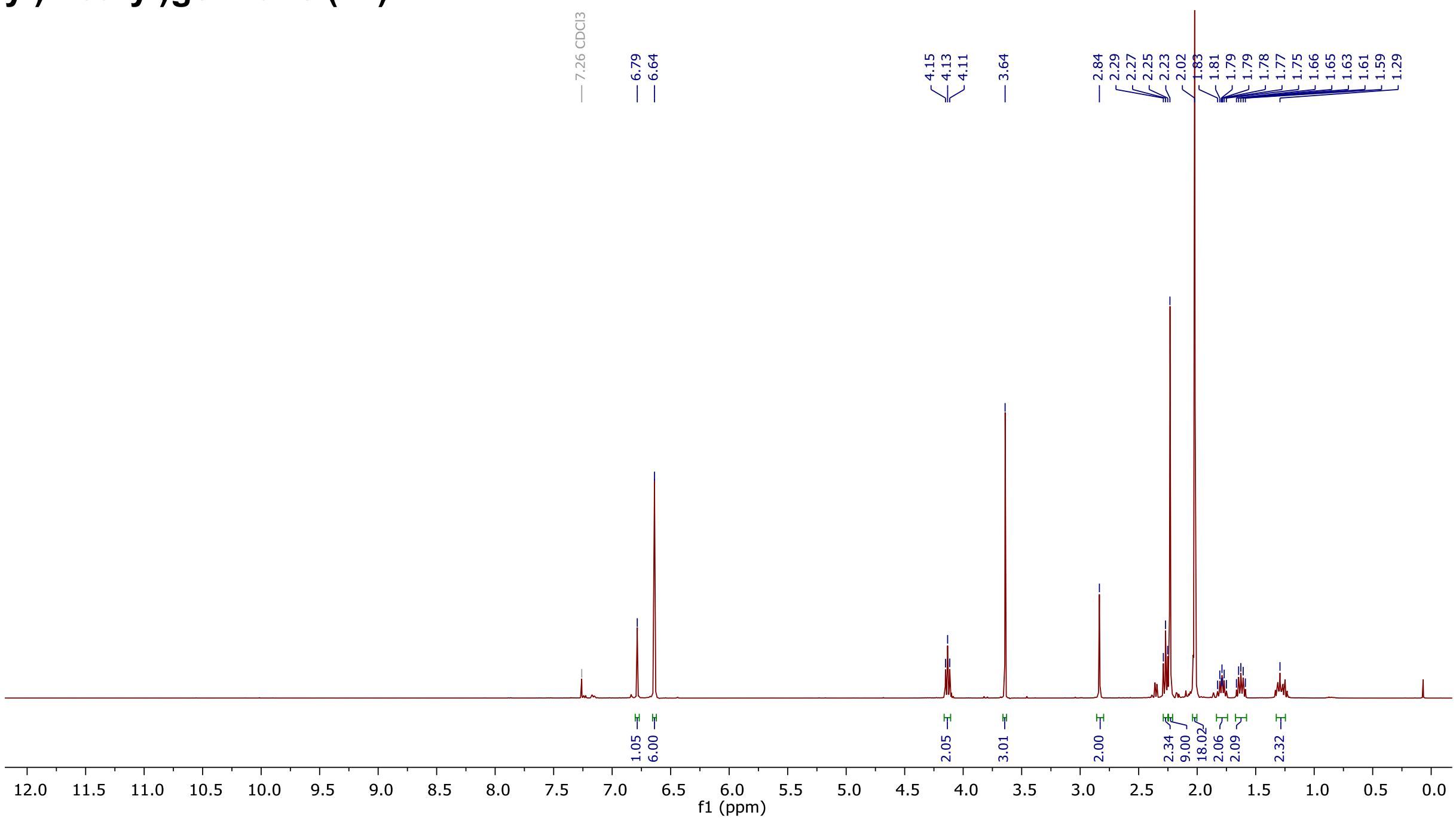

Figure S35. ^{1}H NMR of compound **12** in $CDCl_3$.

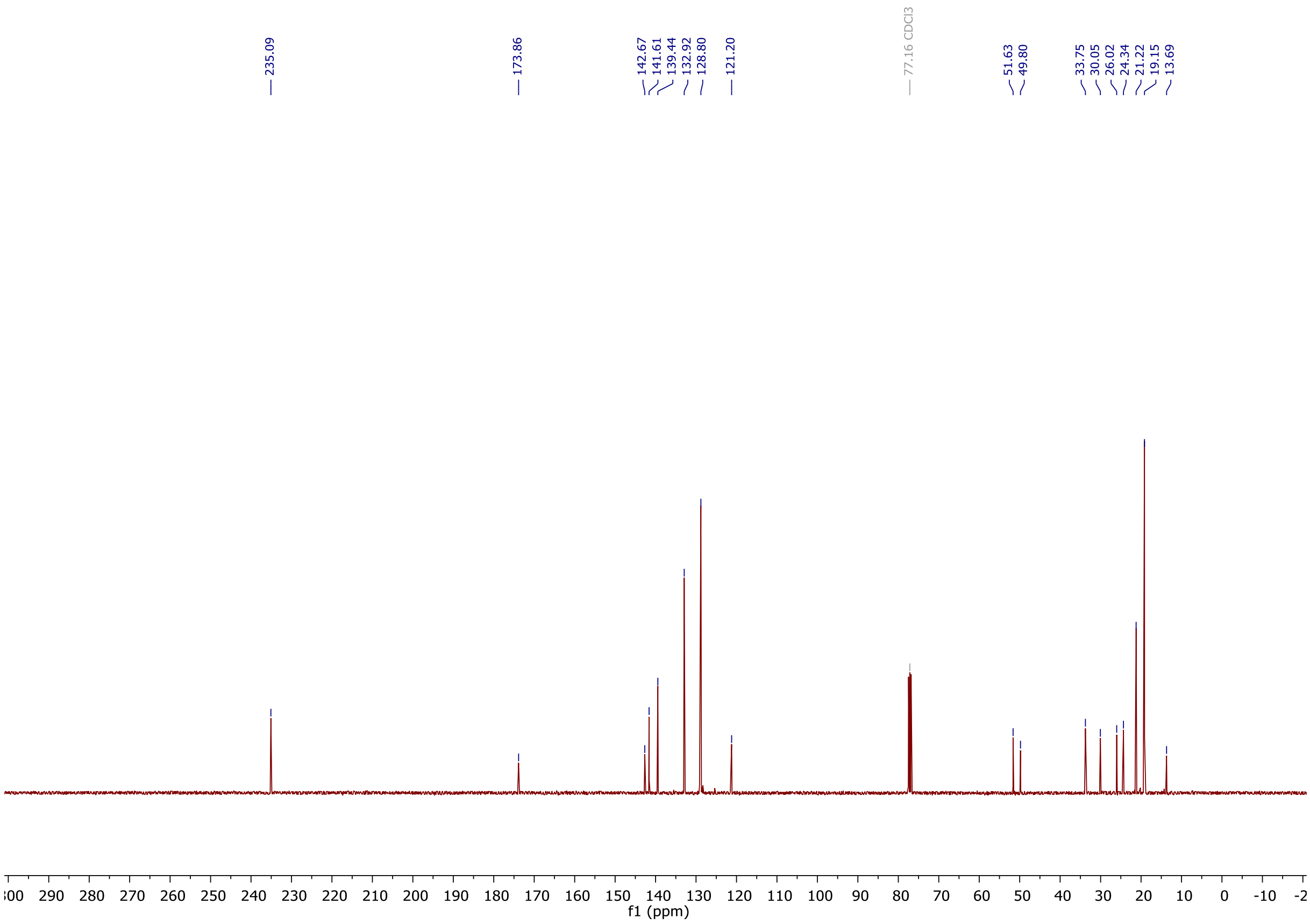

Figure S36. ^{13}C NMR of compound **12** in $CDCl_3$.

Tris(2,4,6-trimethylbenzoyl)(4-(1-(6-(methoxycarbonyl)hexyl)-1,2,3-triazol-4-yl)butanoyl)germane (13)

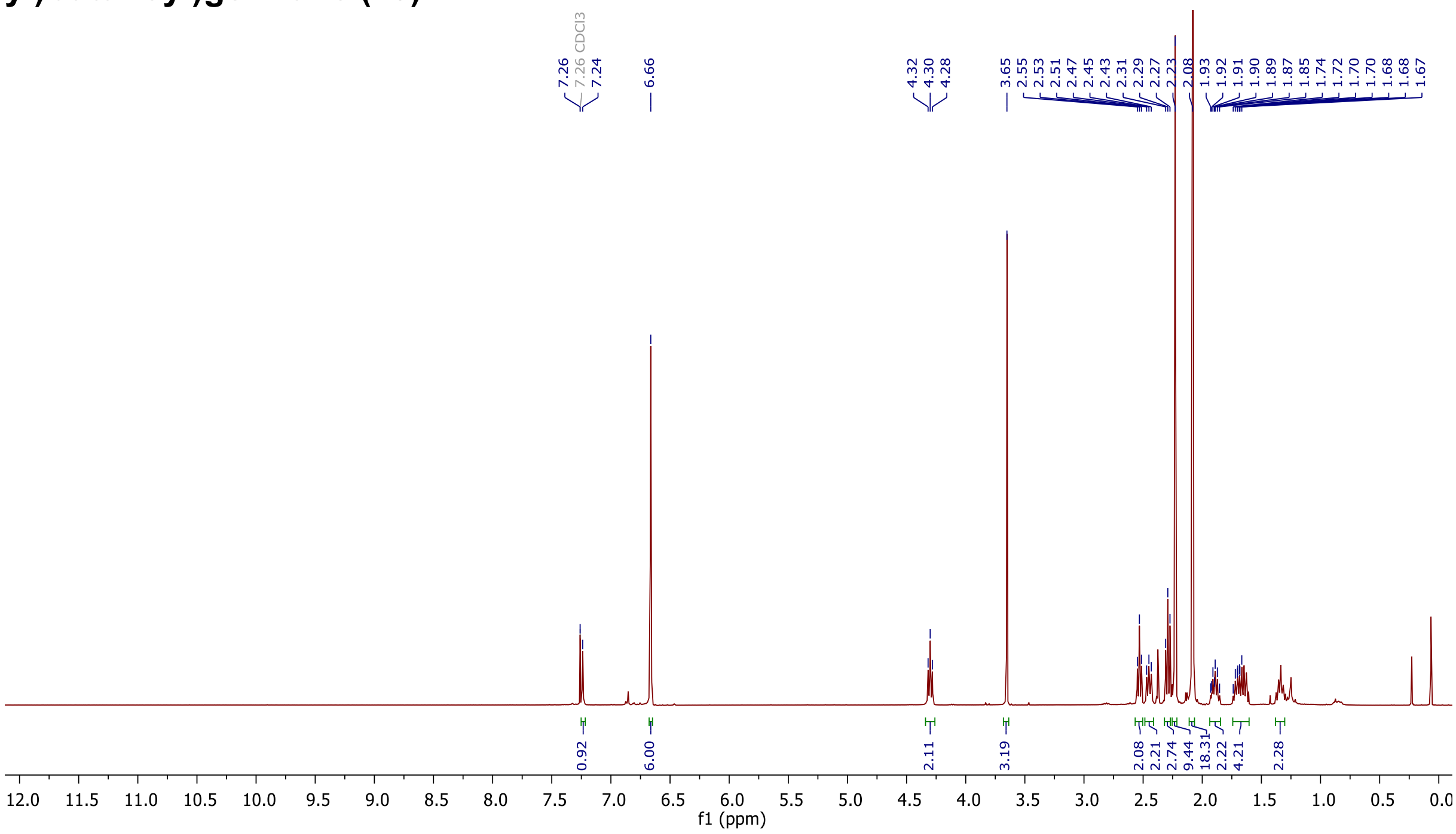

Figure S37. ^1H NMR of compound **13** in $CDCl_3$.

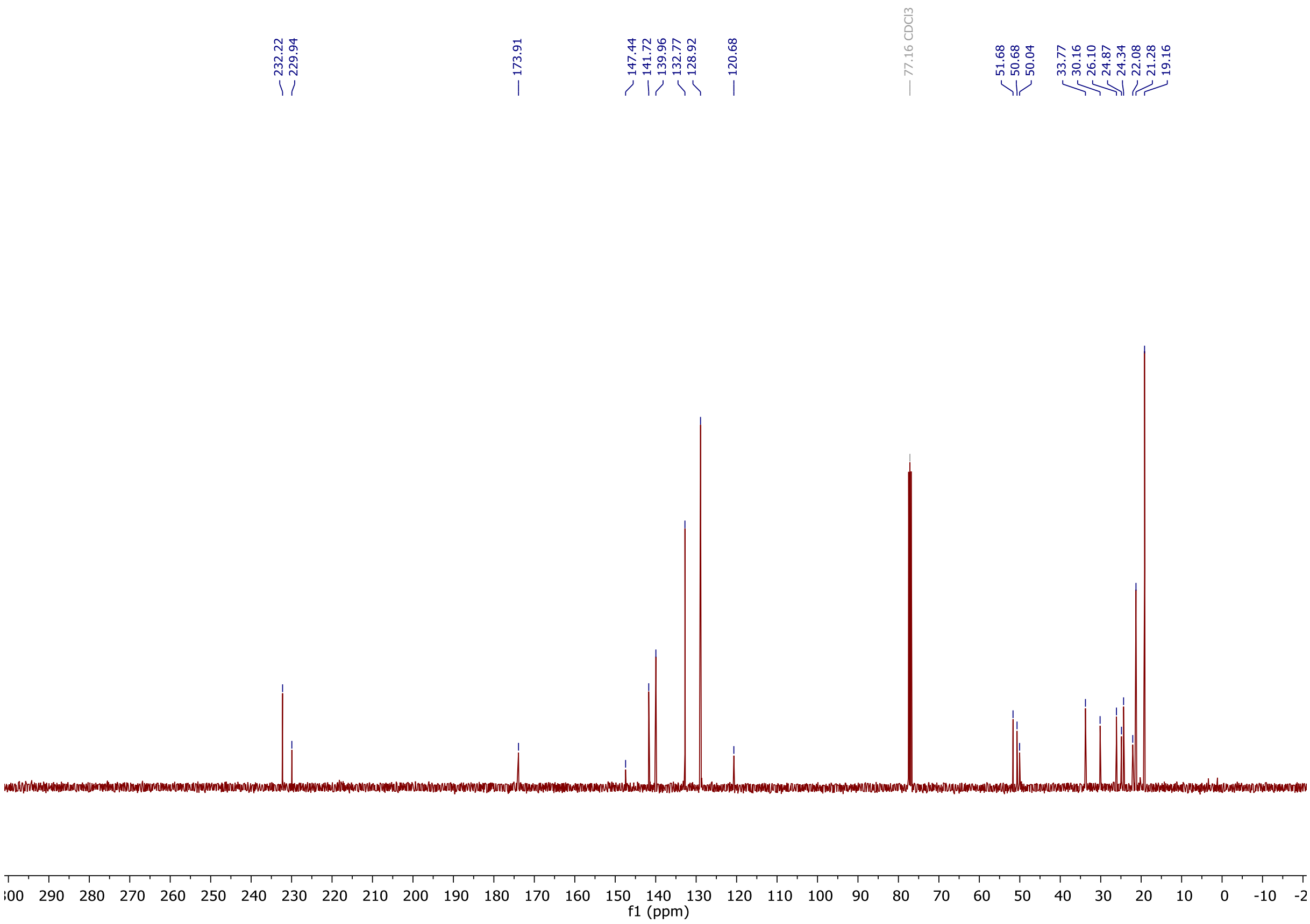

Figure S38. ^{13}C NMR of compound **13** in $CDCl_3$.

Tris(2,4,6-trimethylbenzoyl)(4-(1-phenyl-1,2,3-triazol-4-yl)benzoyl)germane (14)

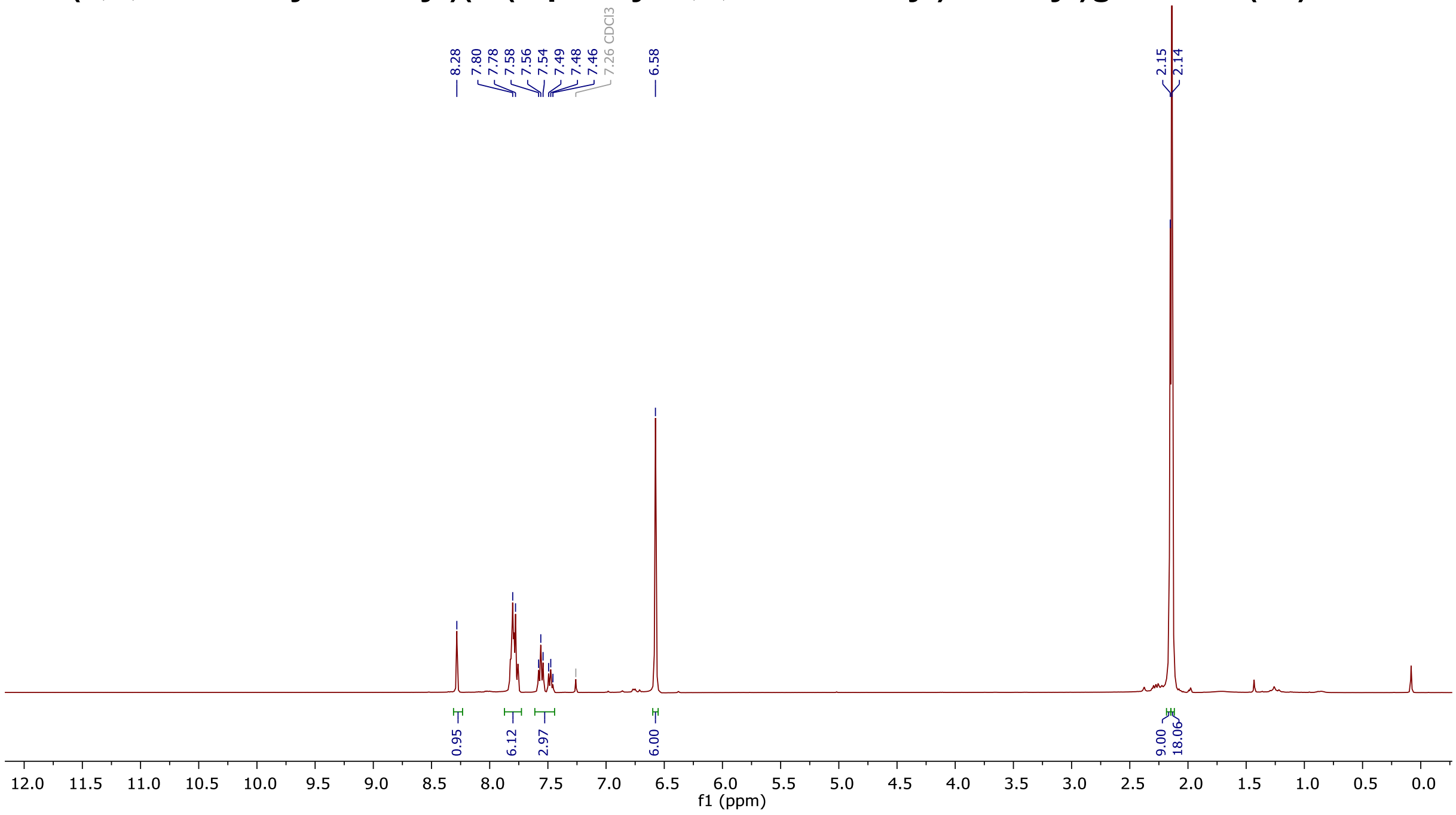

Figure S39. ^{1}H NMR of compound **14** in $CDCl_3$.

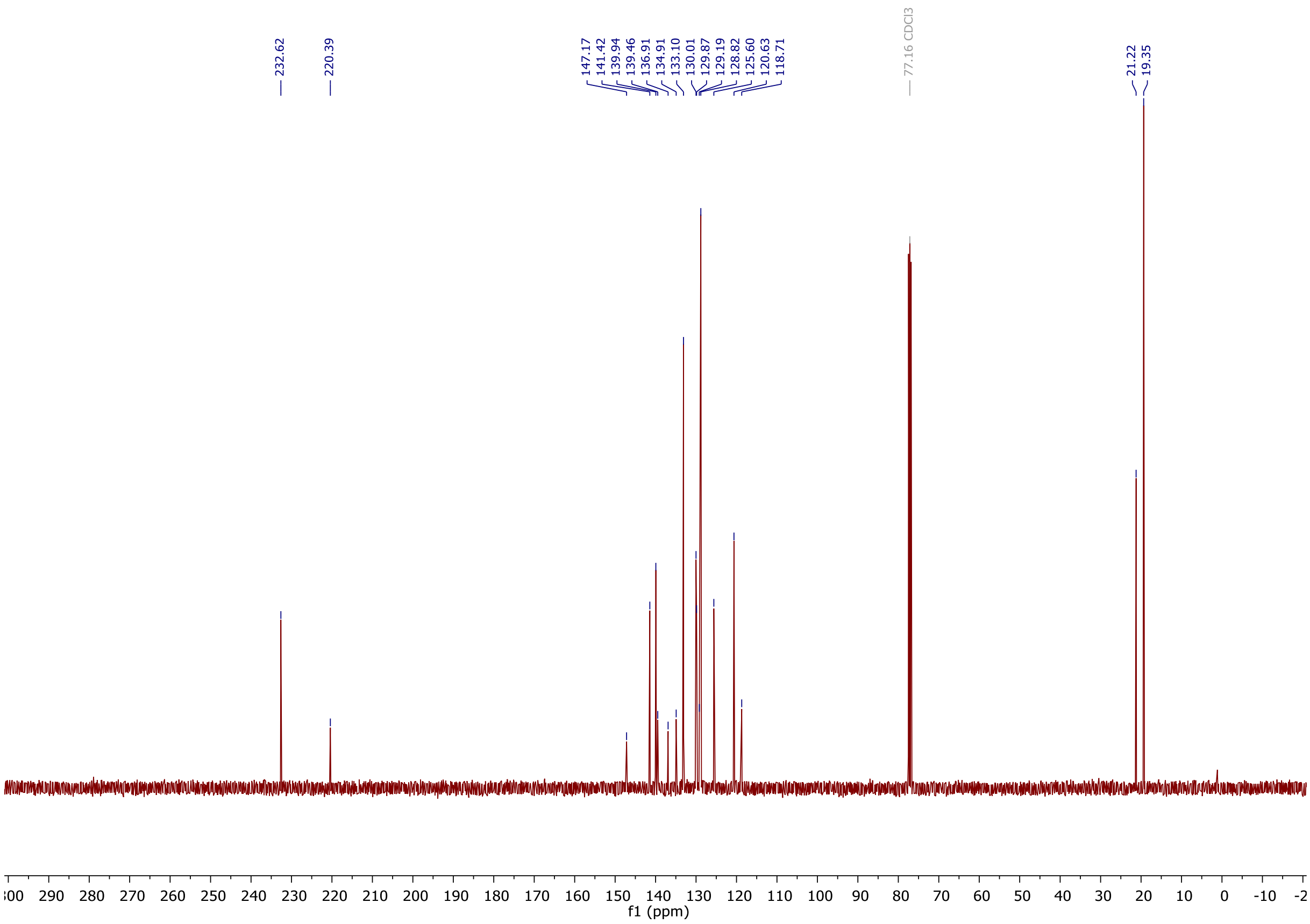

Figure S40. ^{13}C NMR of compound **14** in $CDCl_3$.

Tris(2,4,6-trimethylbenzoyl)(4-(1-(4-carboxyphenyl)-1,2,3-triazol-4-yl)benzoyl)germane (15)

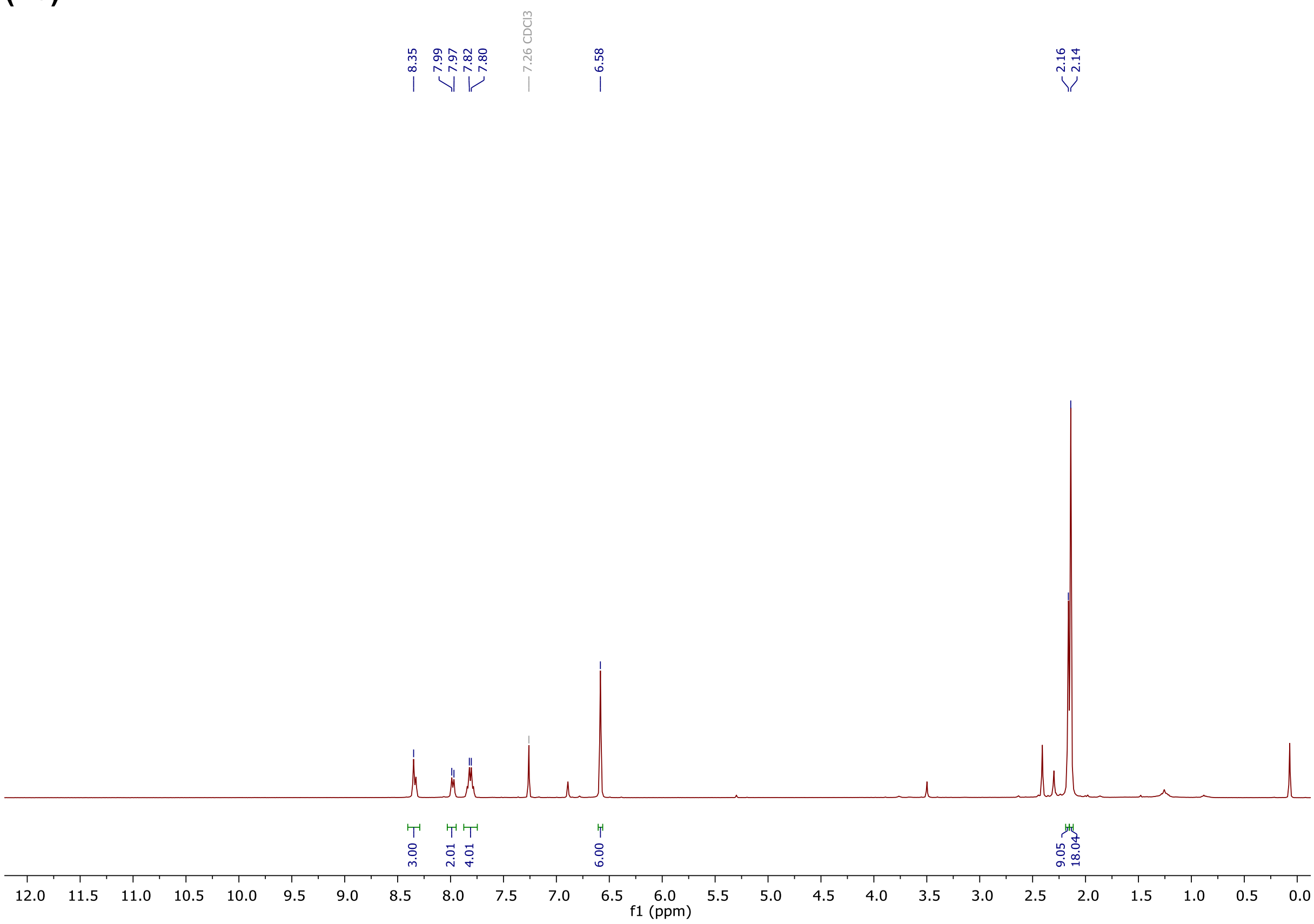

Figure S41. ^{1}H NMR of compound **15** in $CDCl_3$.

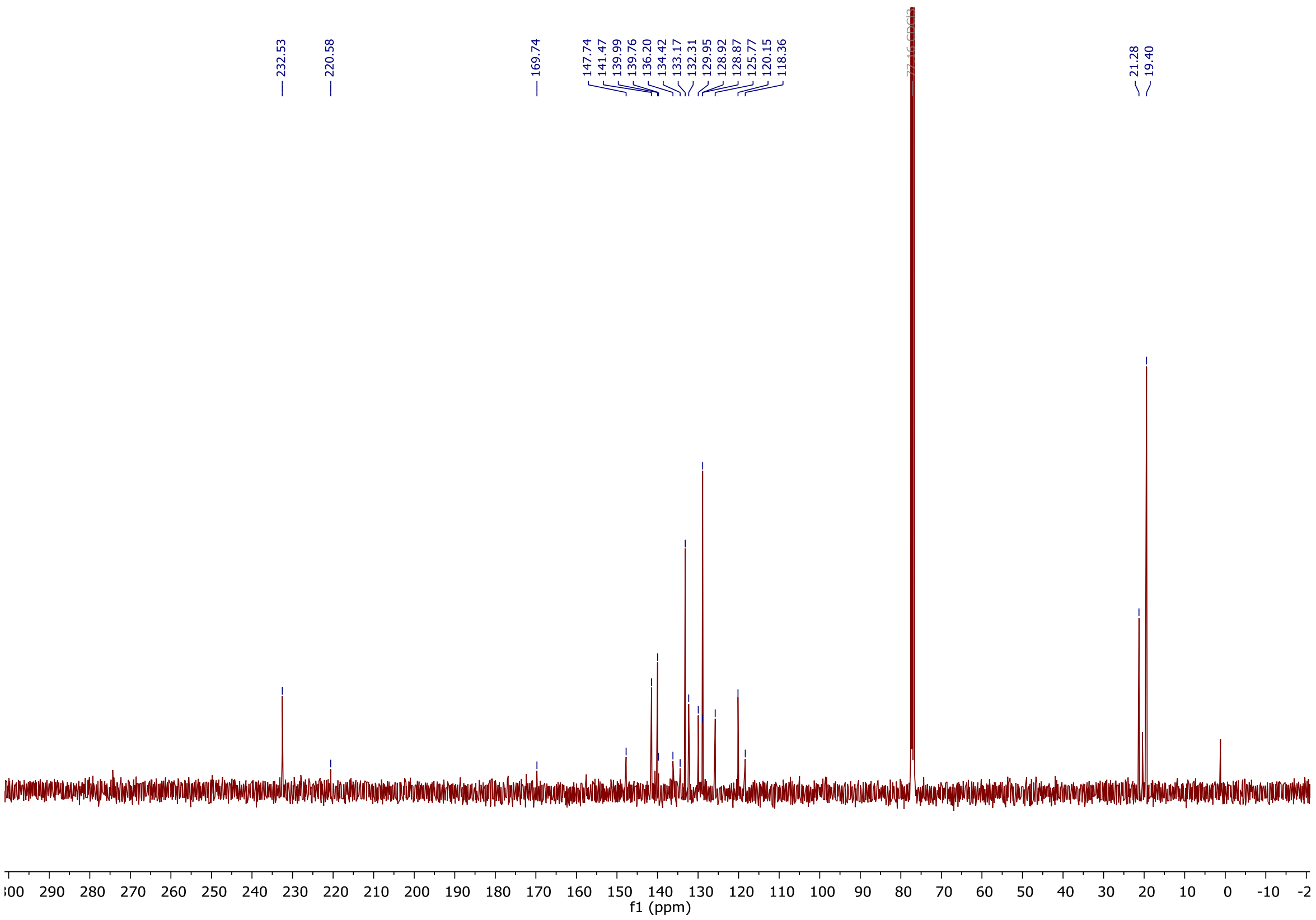

Figure S42. ^{13}C NMR of compound **15** in $CDCl_3$.

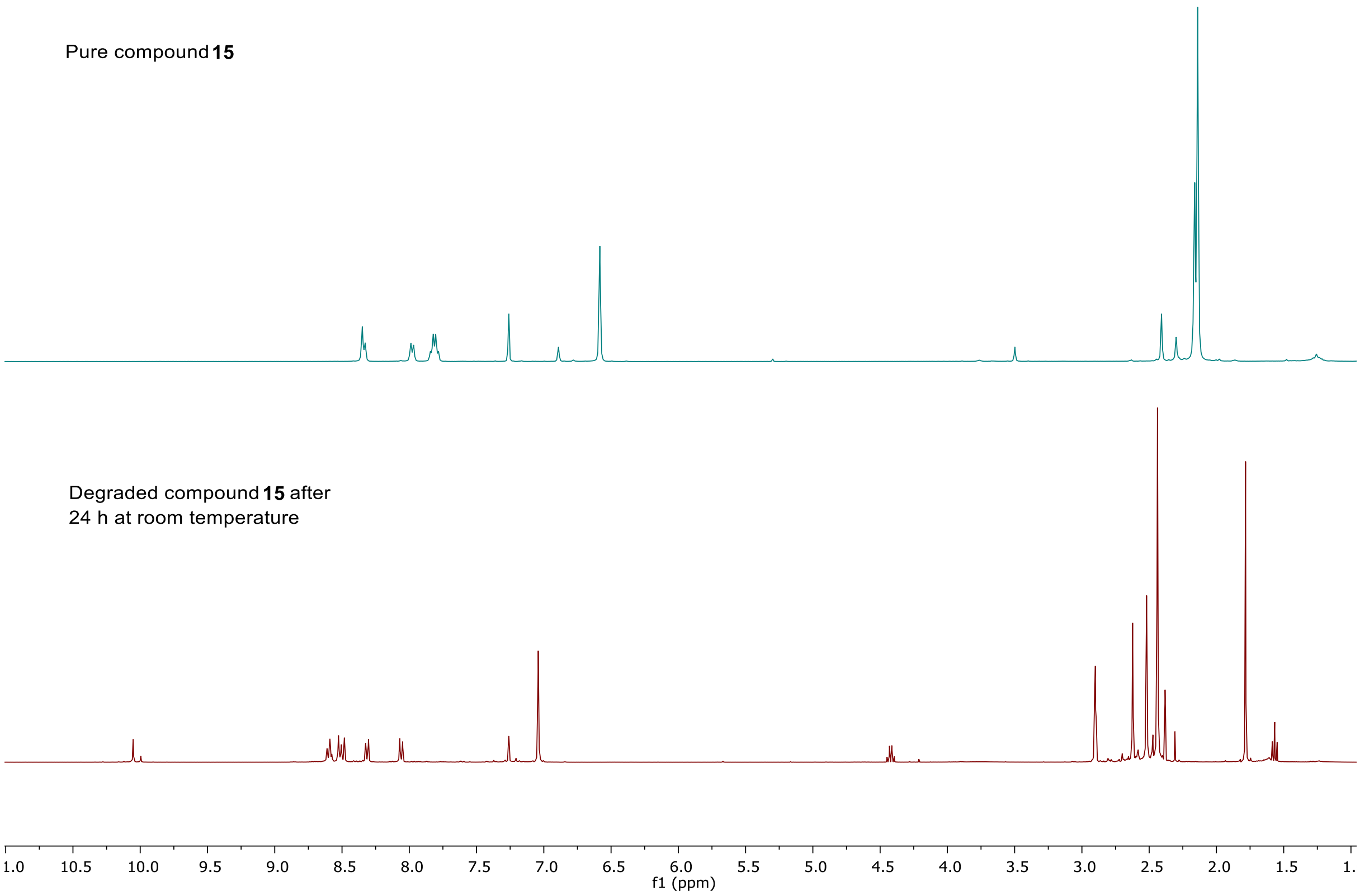

Figure S43. ^{1}H NMR representation of the degradation of compound **15**.

Tris(2,4,6-trimethylbenzoyl)(4-(1-(4-(methoxycarbonyl)phenyl)-1,2,3-triazol-4-yl)benzoyl)germane (16)

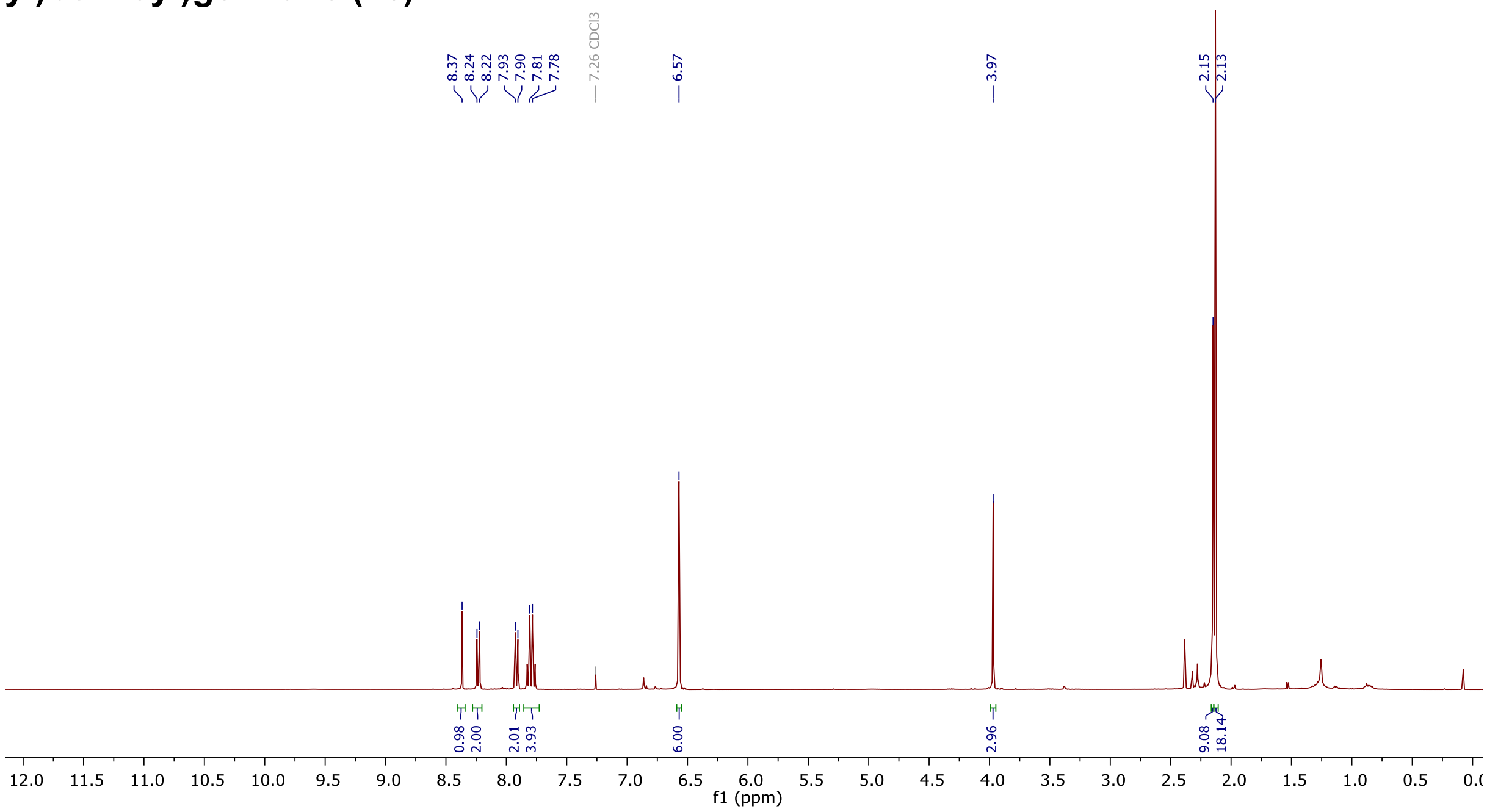

Figure S44. ^{1}H NMR of compound **16** in $CDCl_3$.

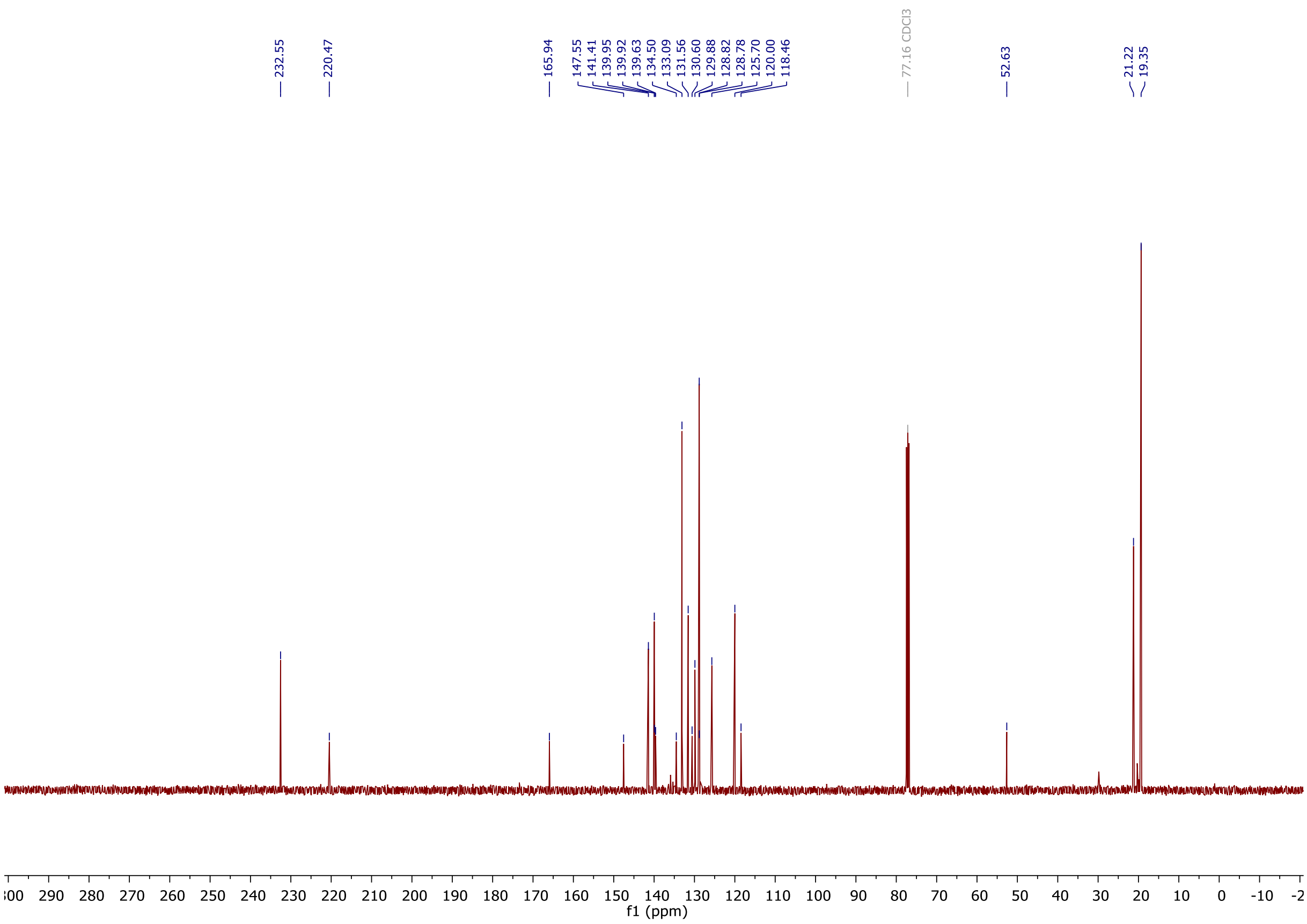

Figure S45. ^{13}C NMR of compound **16** in $CDCl_3$.

Tris(2,4,6-trimethylbenzoyl)(4-(1-(6-deoxy-6-(methyl-α-D-glucopyranosyl)-1,2,3-triazol-4-yl)benzoyl)germane (17)

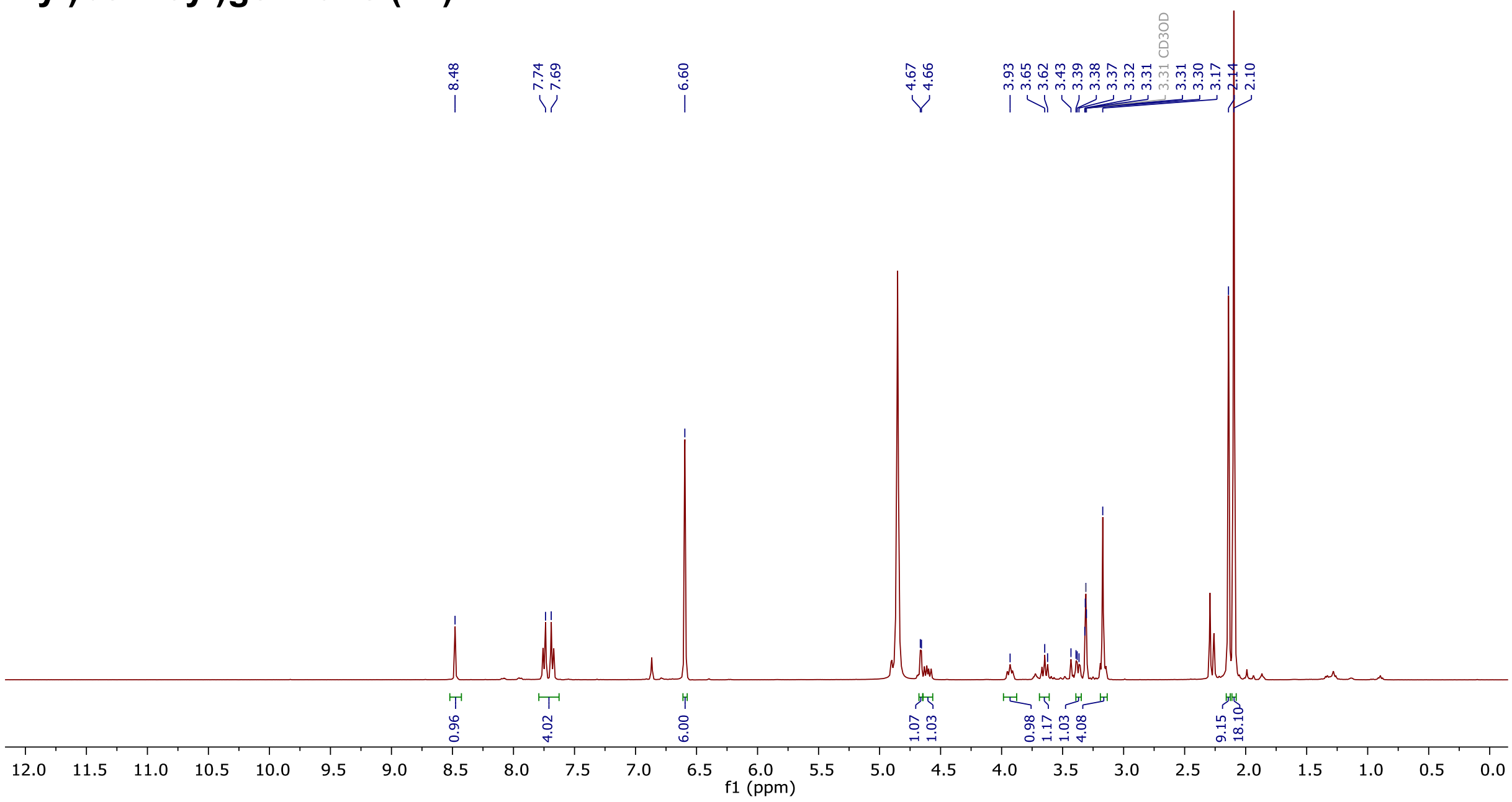

Figure S46. ^{1}H NMR of compound **17** in MeOD.

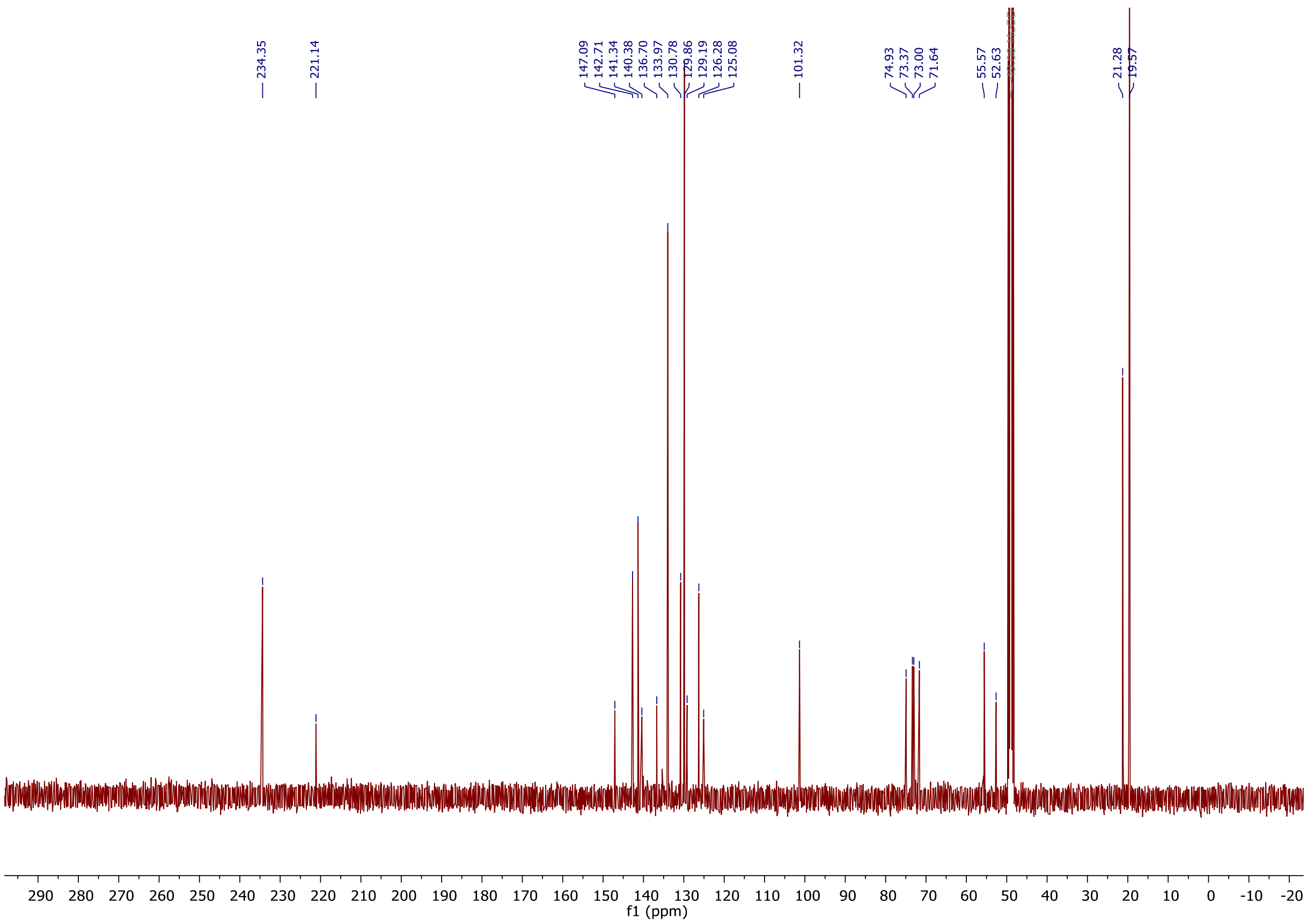

Figure S47. ^{13}C NMR of compound **17** in MeOD.

Tris(2,4,6-trimethylbenzoyl)(1-(6-deoxy-6-(methyl-α-D-glucopyranosyl)-1,2,3-triazol-4-yl)methyl)germane (18)

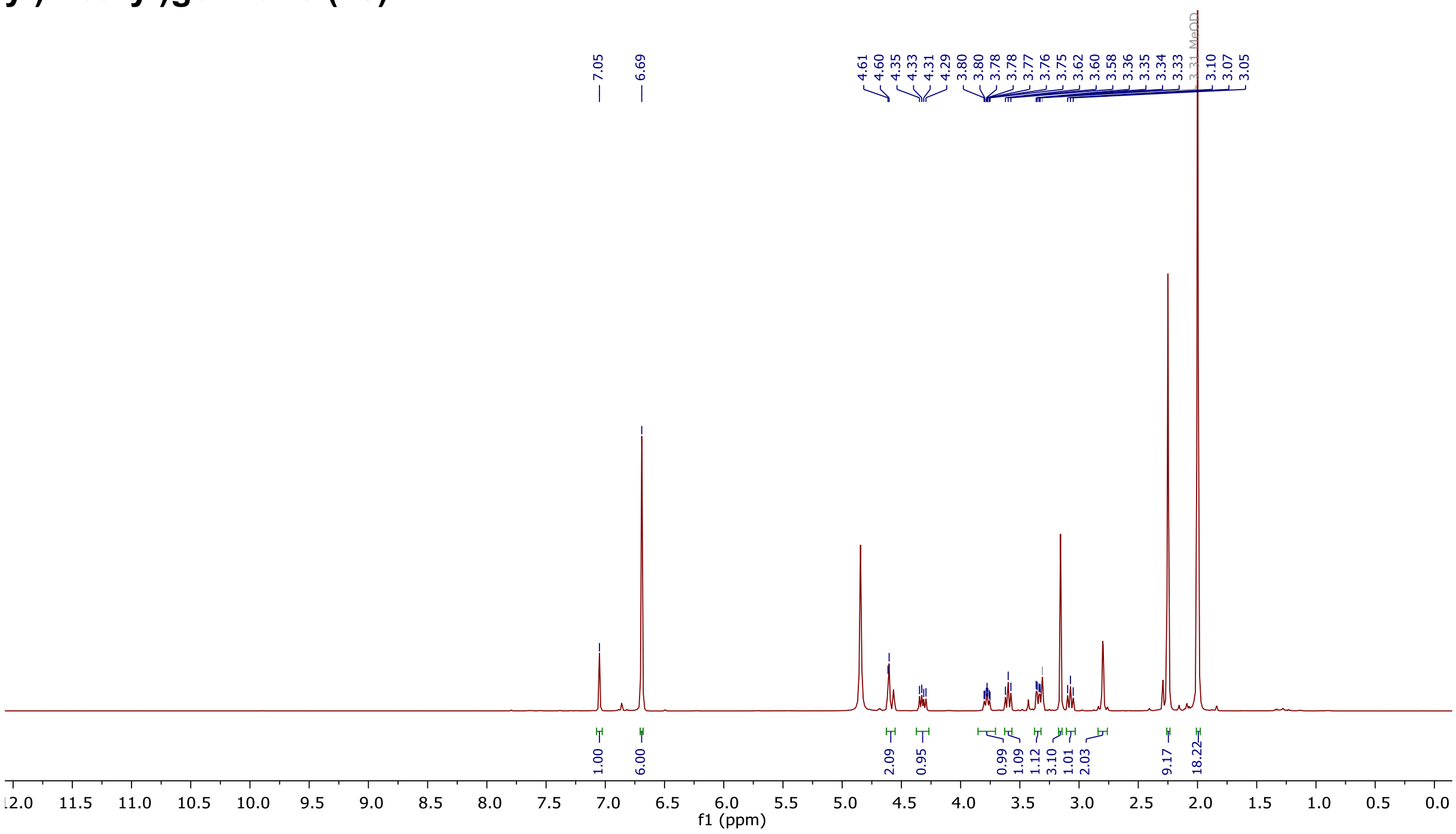

Figure S48. ^{1}H NMR of compound **18** in MeOD.

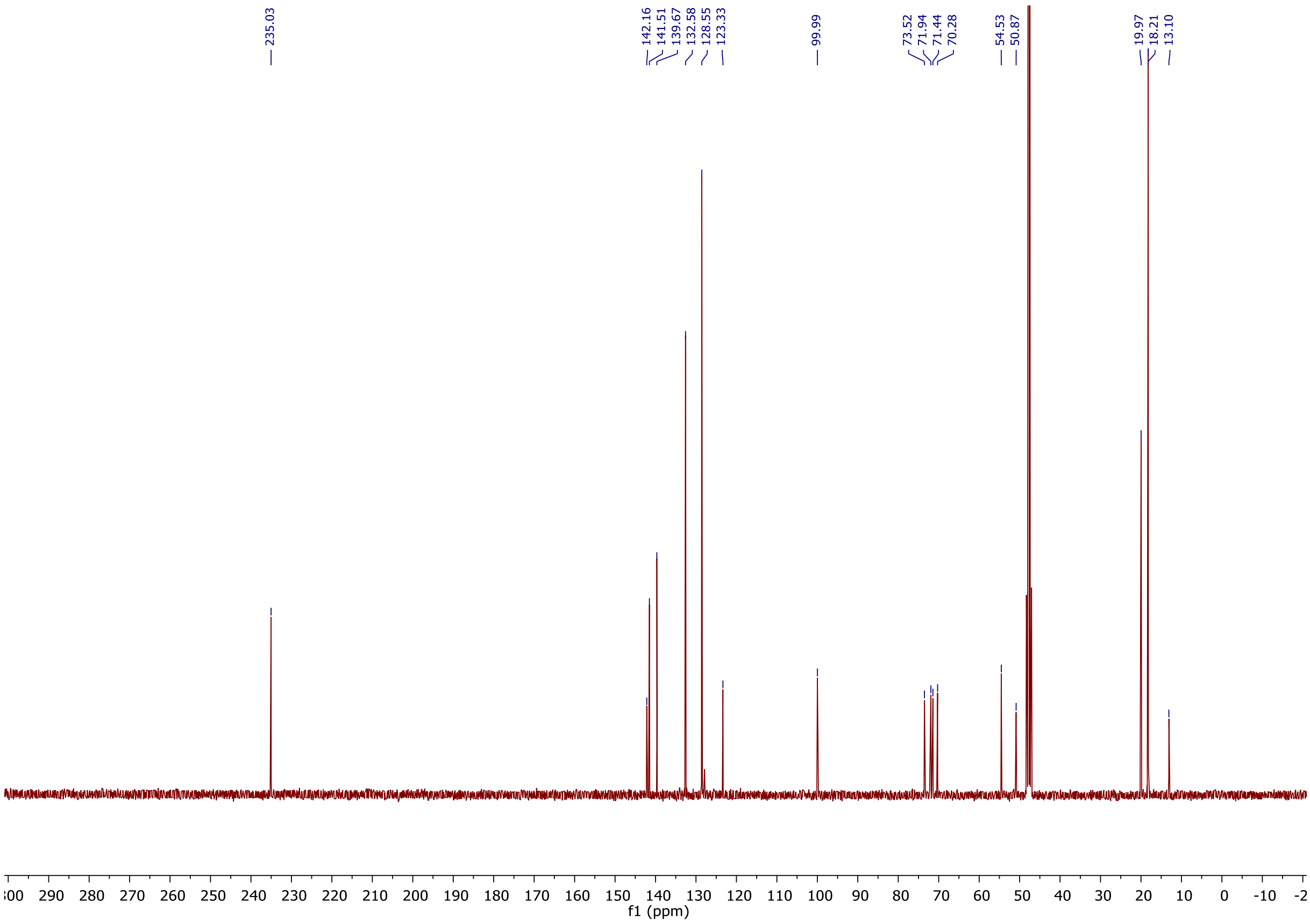

Figure S49. ^{13}C NMR of compound **18** in MeOD.

Tris(2,4,6-trimethylbenzoyl)(4-(1-(6-deoxy-6-(methyl-α-D-glucopyranosyl)-1,2,3-triazol-4-yl)butanoyl)germane (19)

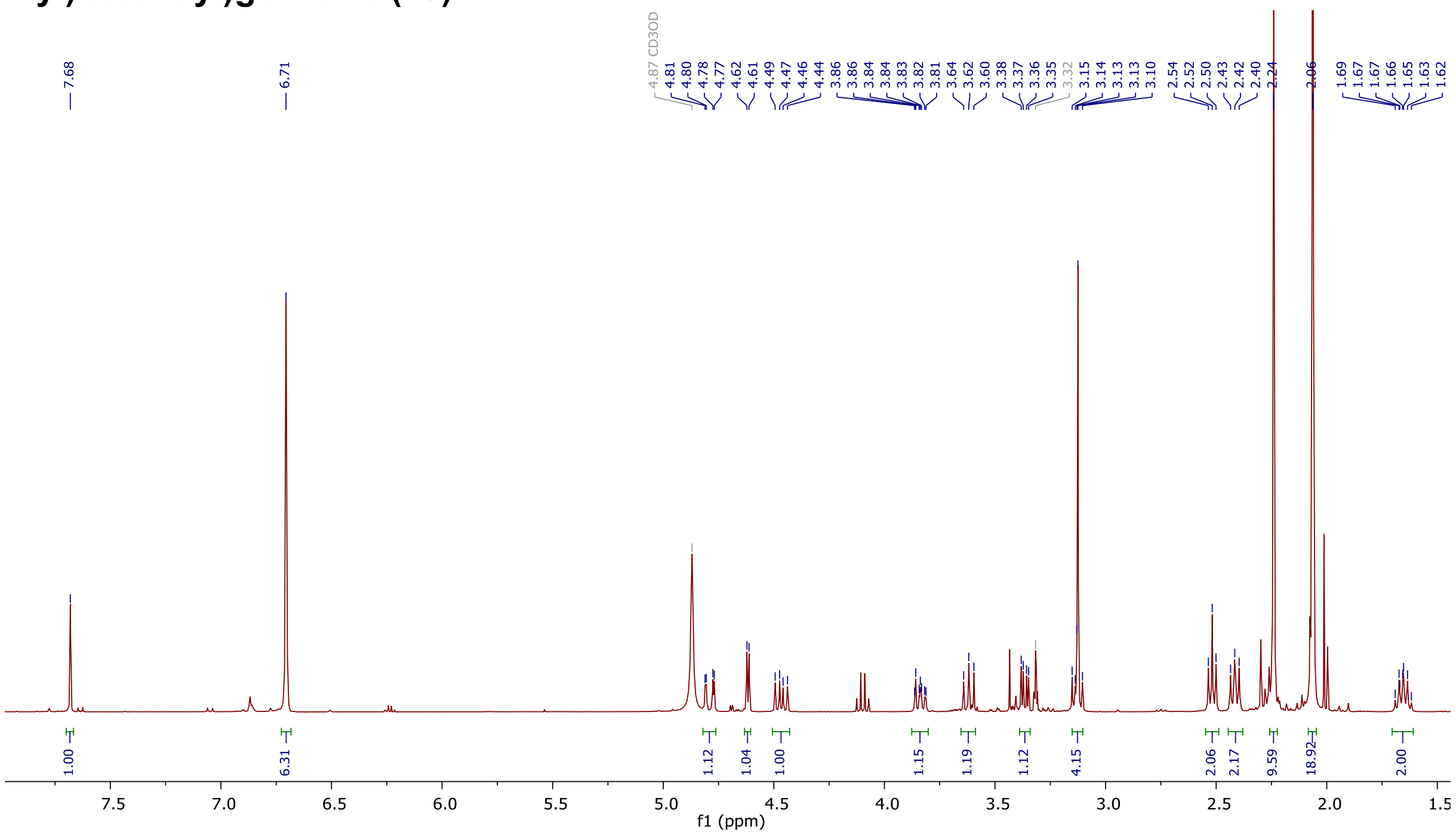

Figure S50. ^{1}H NMR of compound **19** in MeOD.

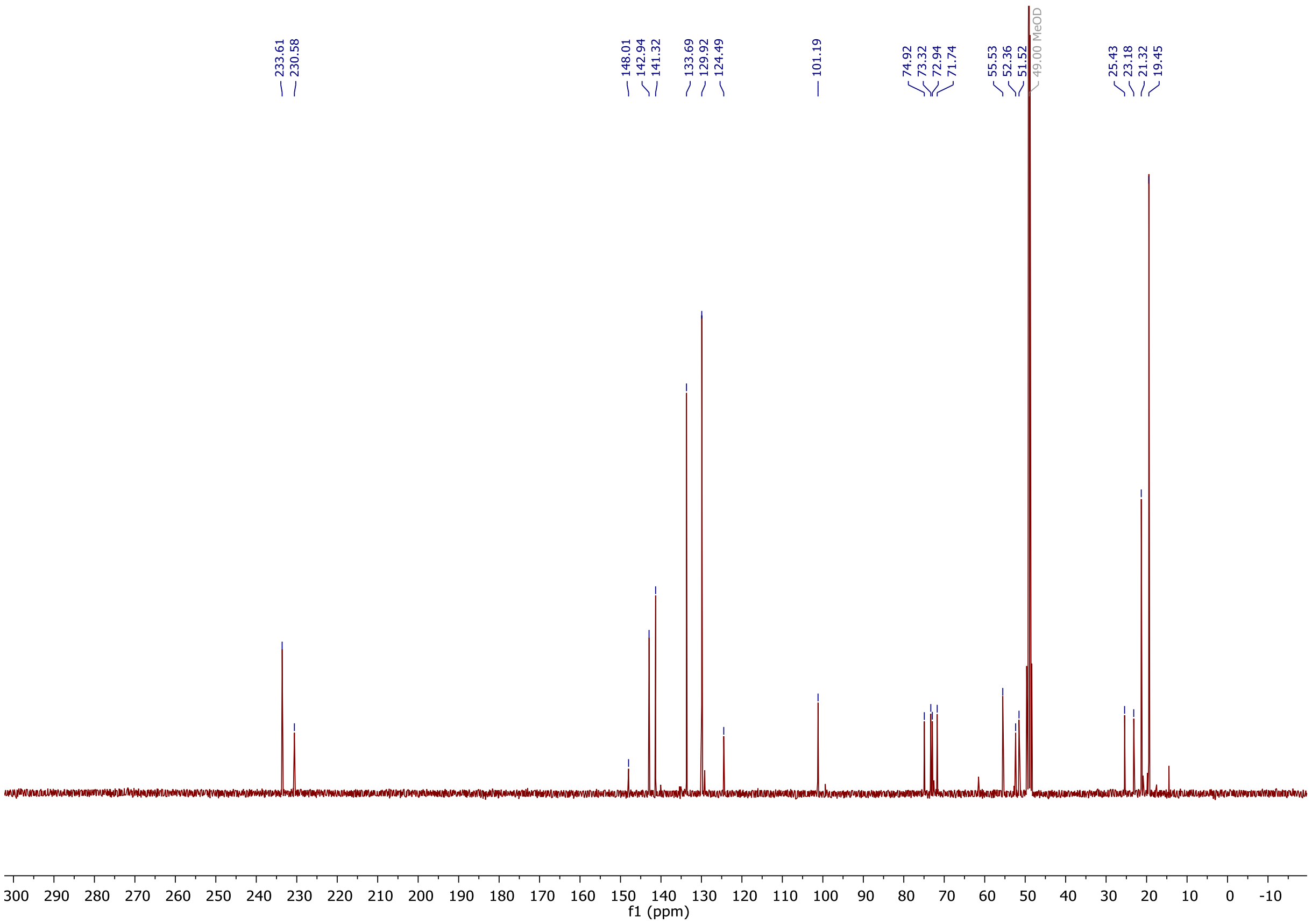

Figure S51. ^{13}C NMR of compound **19** in MeOD.

Tris(2,4,6-trimethylbenzoyl)(4-(1-phenyl-1,2,3-triazol-4-yl)benzoyl)germane (20)

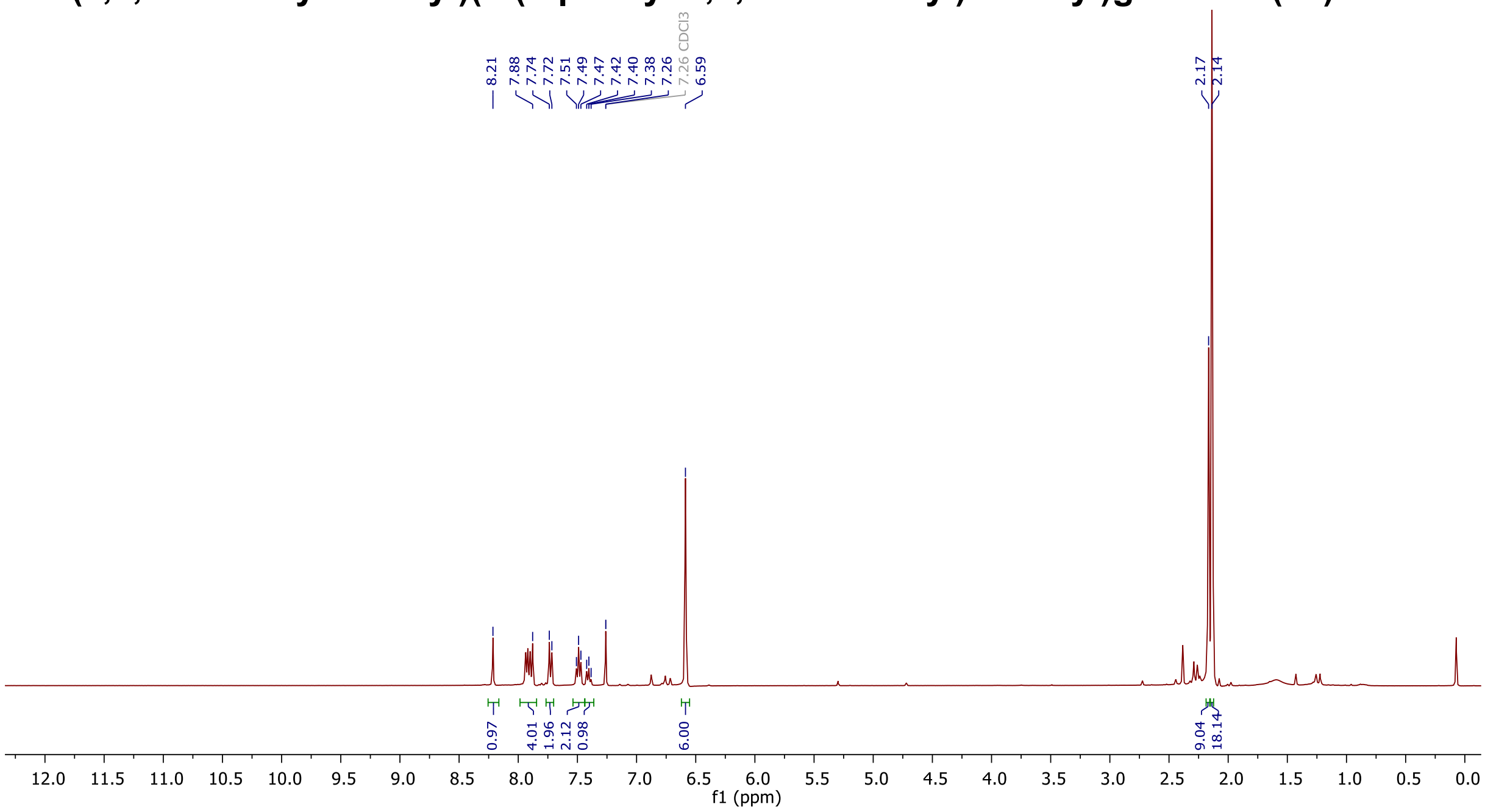

Figure S52. ^{1}H NMR of compound **20** in $CDCl_3$.

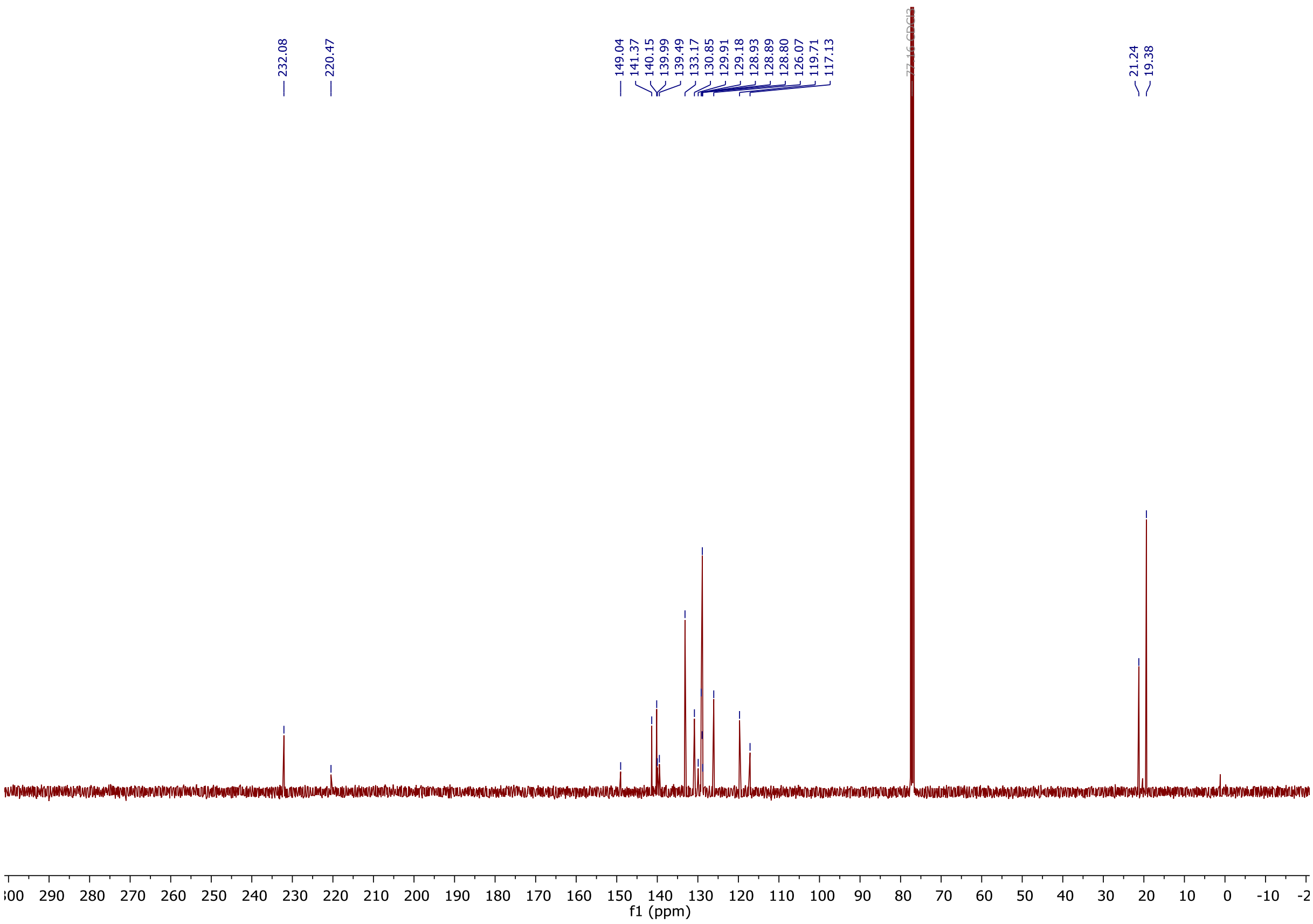

Figure S53. ^{13}C NMR of compound **20** in $CDCl_3$.

Tris(2,4,6-trimethylbenzoyl)(4-(4-(4-carboxyphenyl)-1,2,3-triazol-1-yl)benzoyl)germane (21)

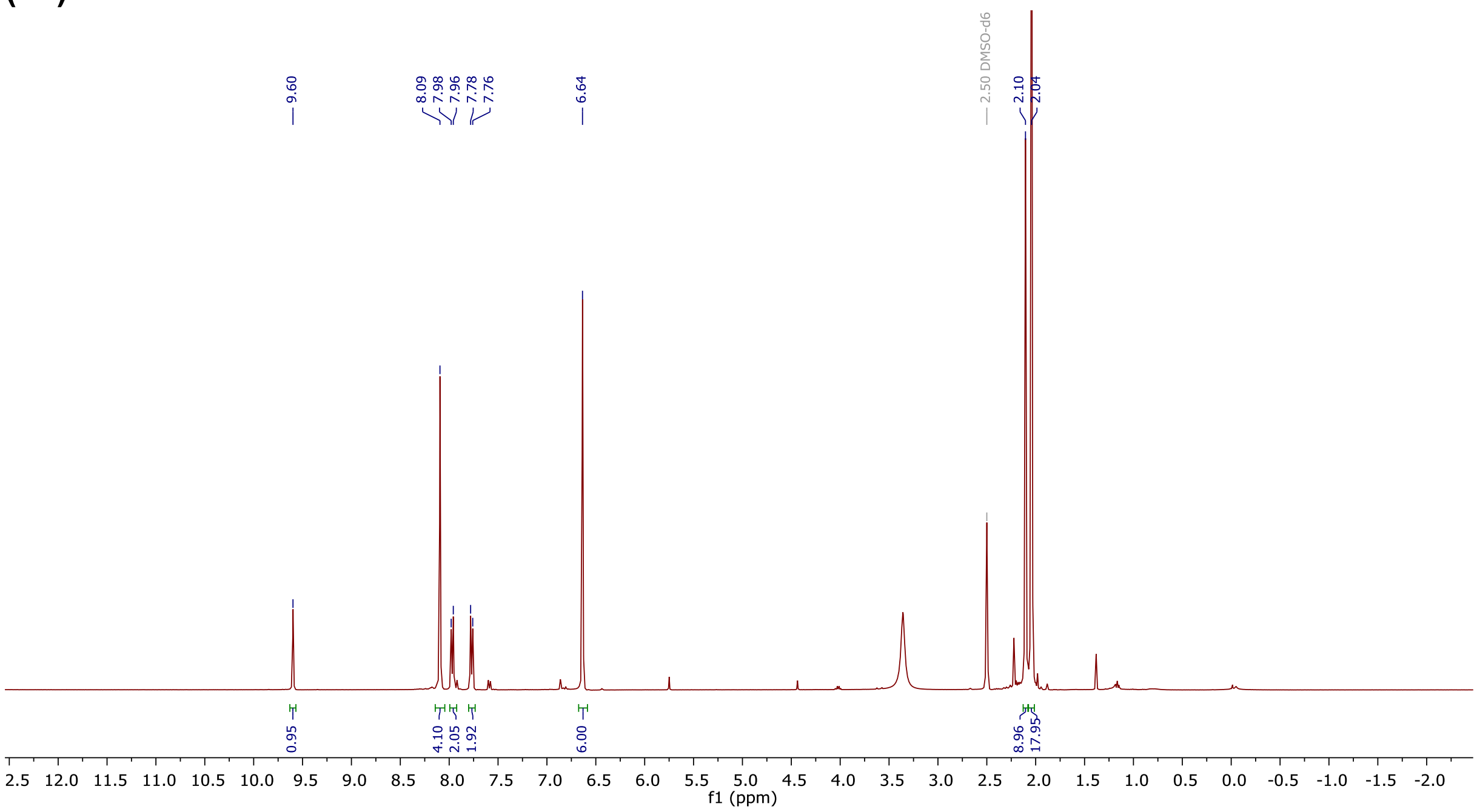

Figure S54. ^{1}H NMR of compound **21** in DMSO-d6.

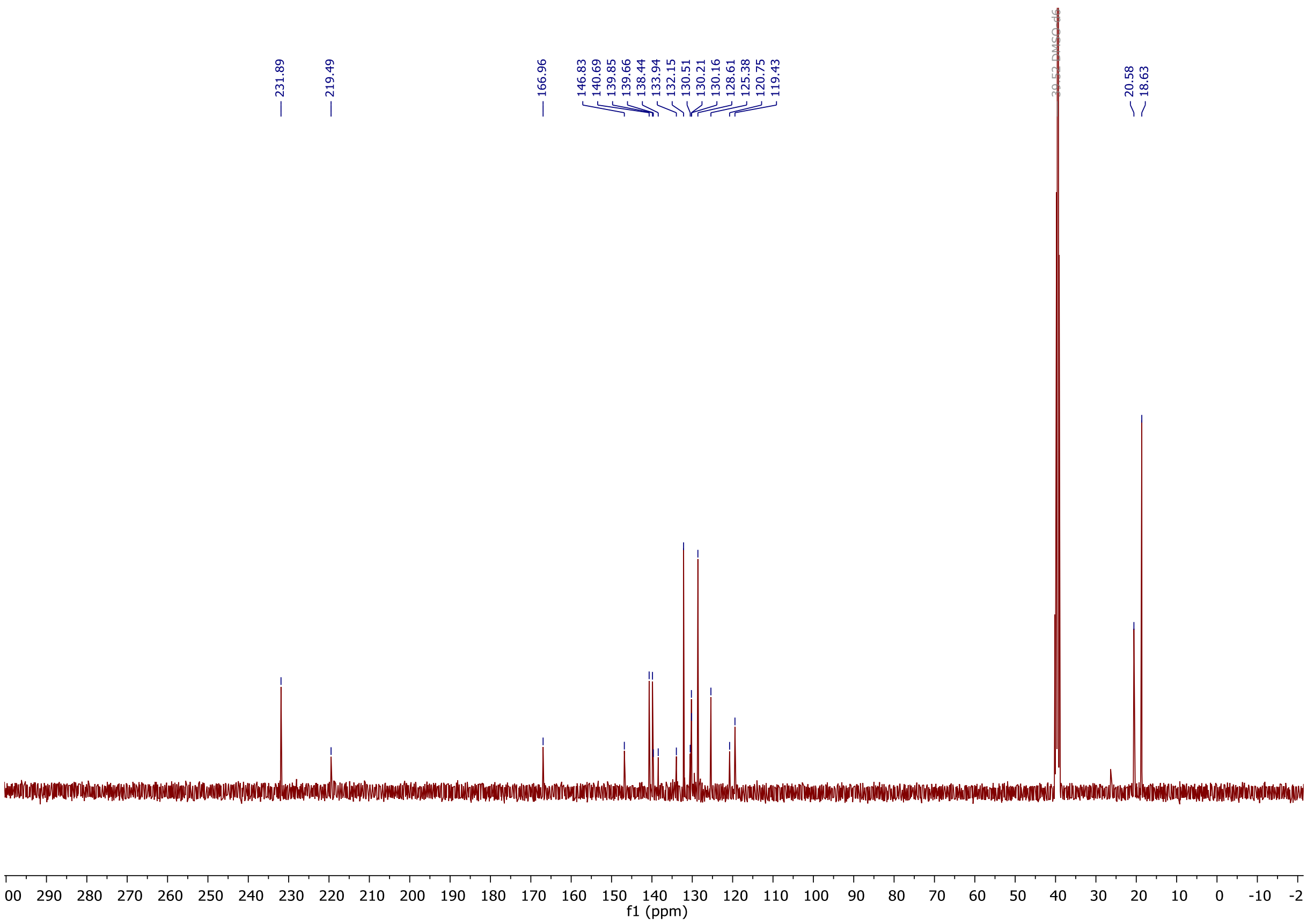

Figure S55. ^{13}C NMR of compound **21** in DMSO-d6.

Tris(2,4,6-trimethylbenzoyl)(4-(4-(4-(methoxycarbonyl)phenyl)-1,2,3-triazol-1-yl)benzoyl)germane (22)

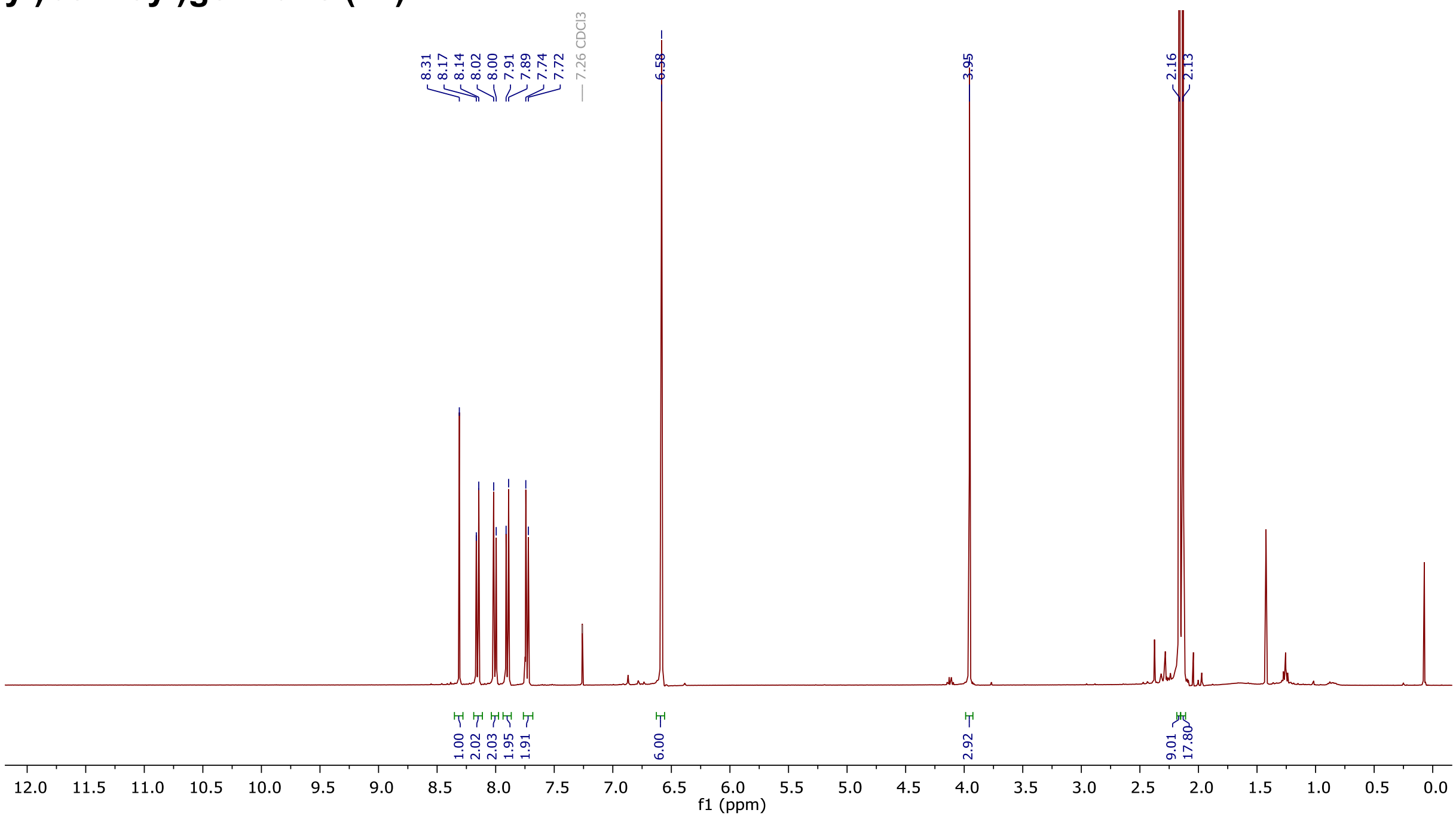

Figure S56. ^{1}H NMR of compound **22** in $CDCl_3$.

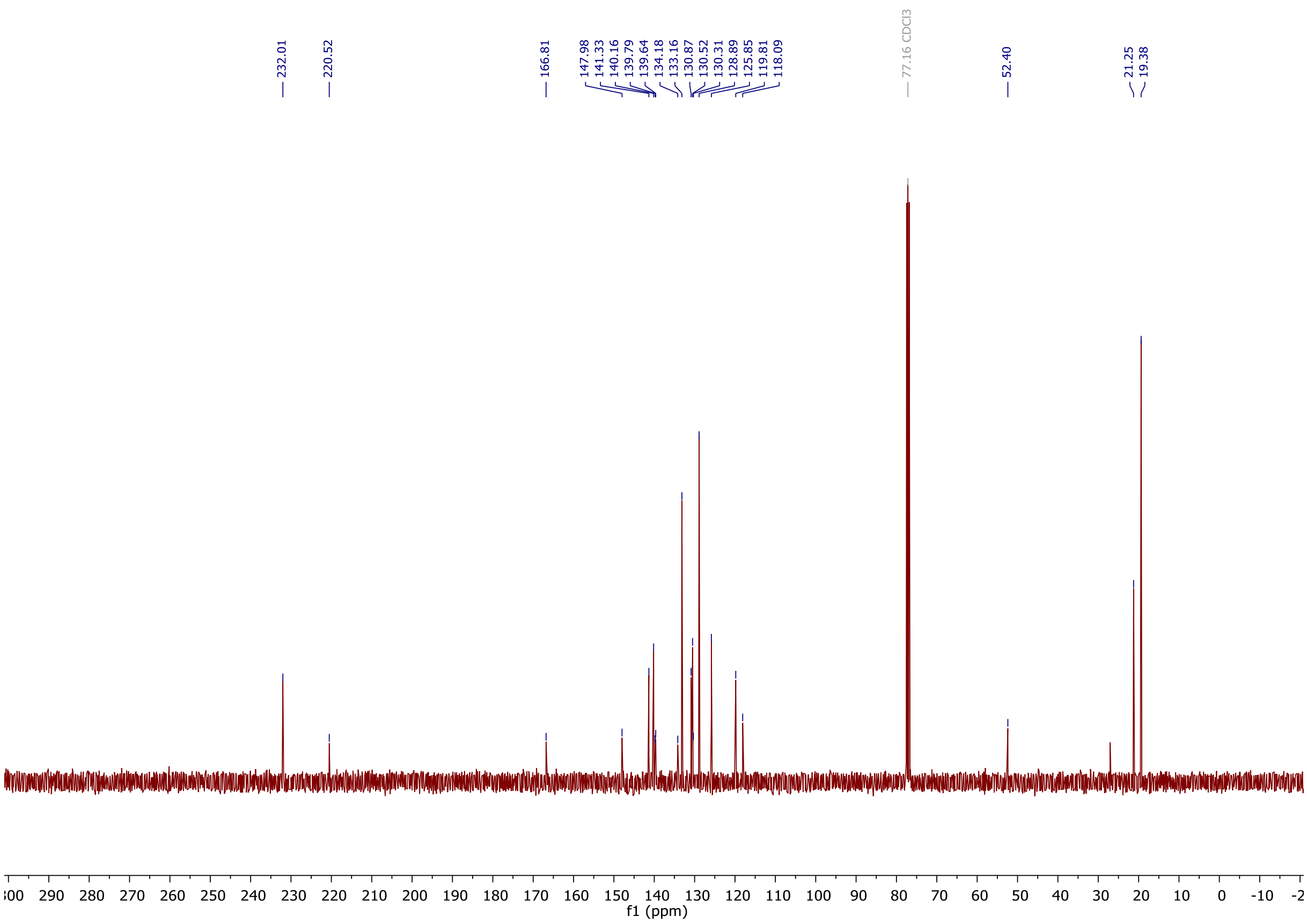

Figure S57. ^{13}C NMR of compound **22** in $CDCl_3$.

Tris(2,4,6-trimethylbenzoyl)(4-(4-(4-(pinacolboronyl)phenyl)-1,2,3-triazol-1-yl)benzoyl)germane (23)

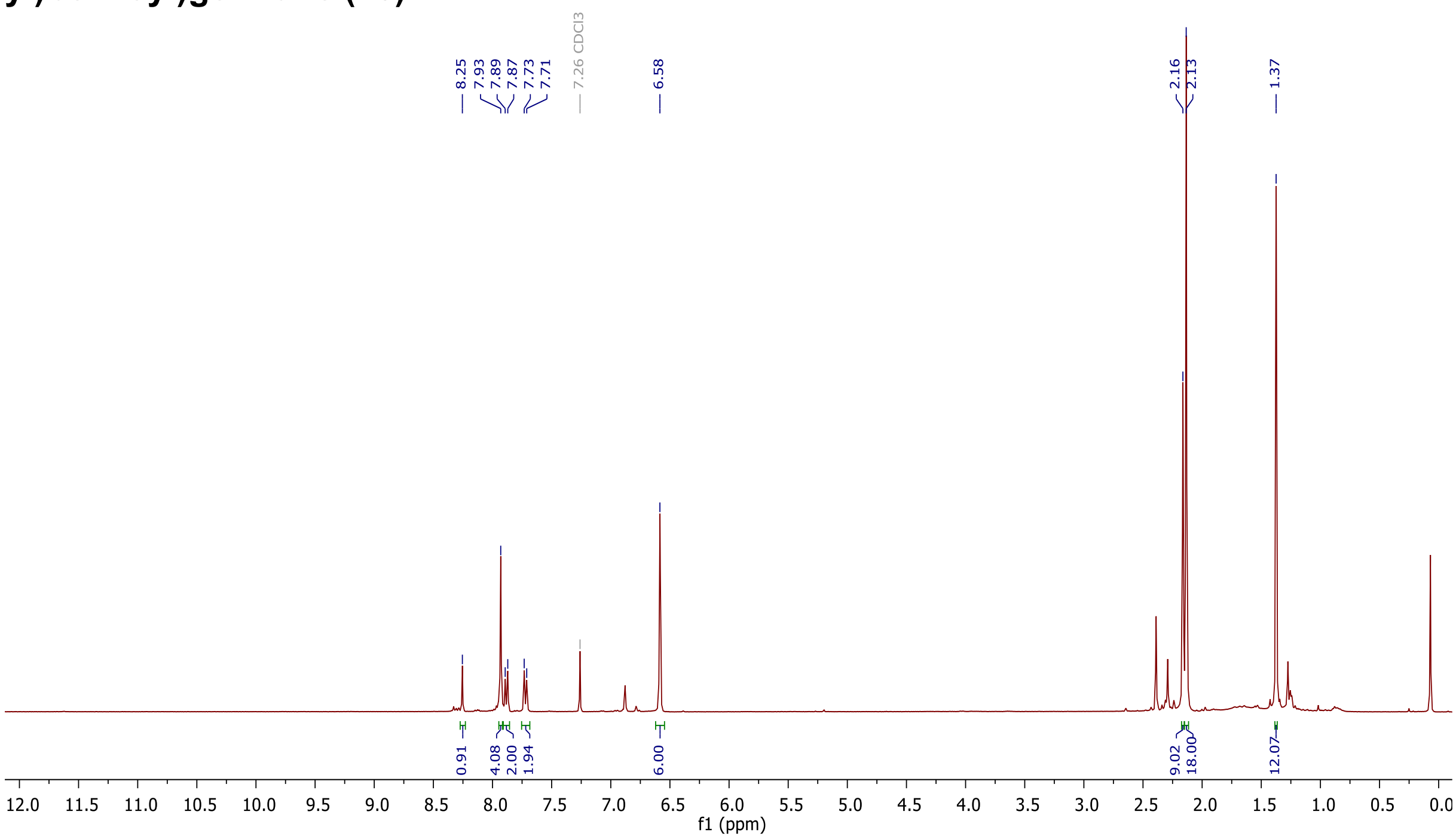

Figure S58. ^{1}H NMR of compound **23** in $CDCl_3$.

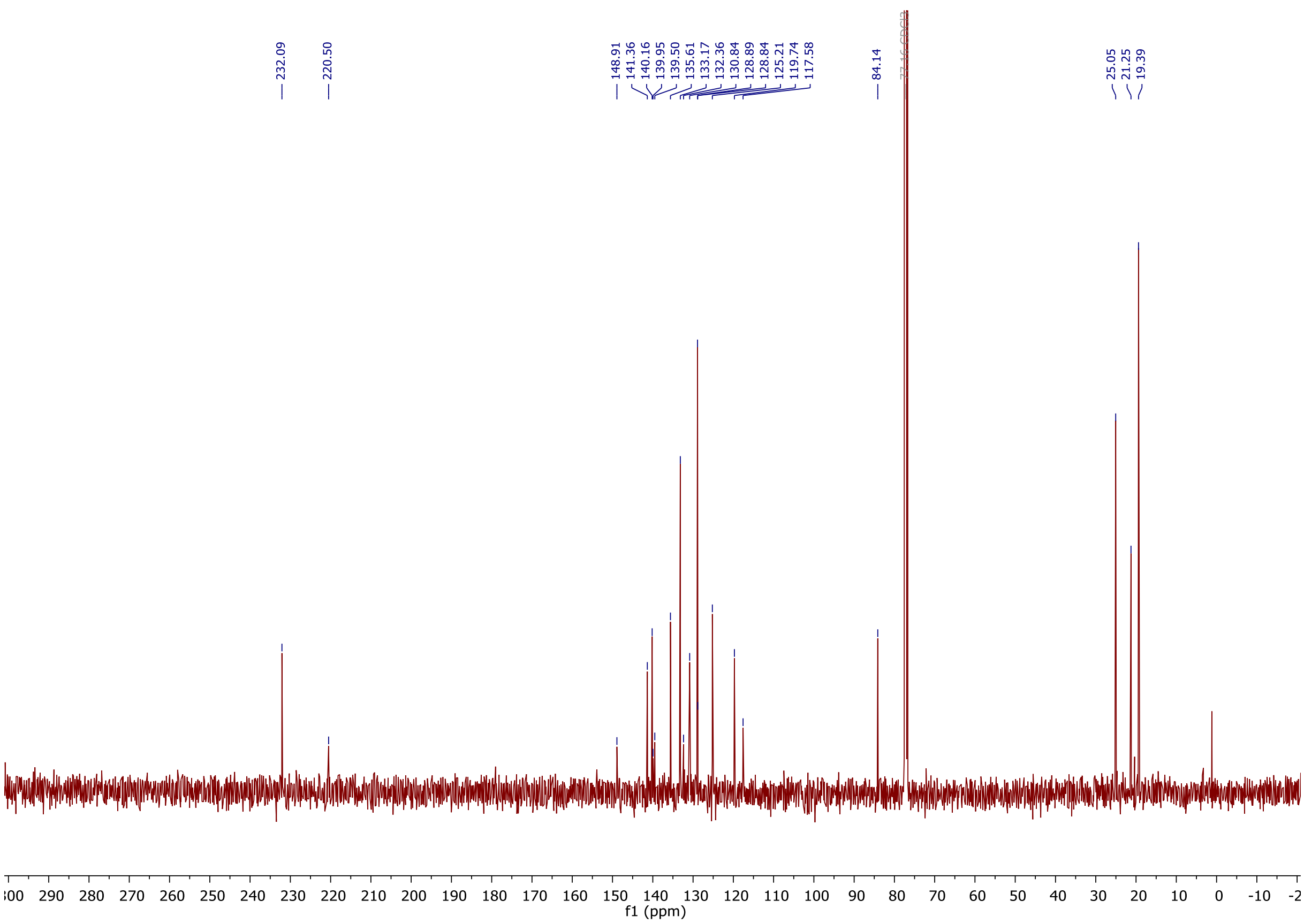

Figure S59. ^{13}C NMR of compound **23** in $CDCl_3$.

Tris(2,4,6-trimethylbenzoyl)(4-(4-(tris(2,4,6-trimethylbenzoyl)germanyl)-1,2,3-triazol-1-yl)benzoyl)germane (24)

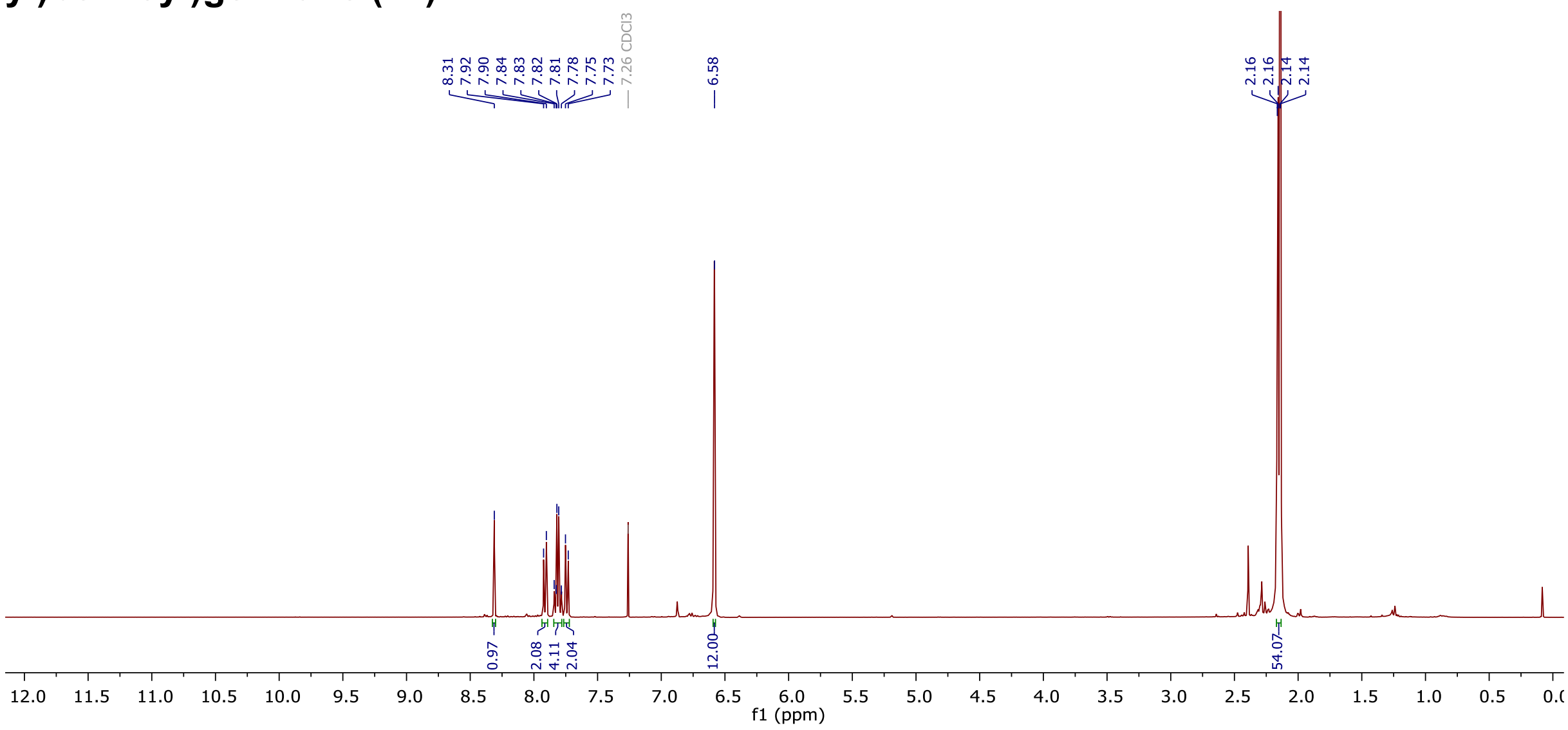

Figure S60. ^{1}H NMR of compound **24** in $CDCl_3$.

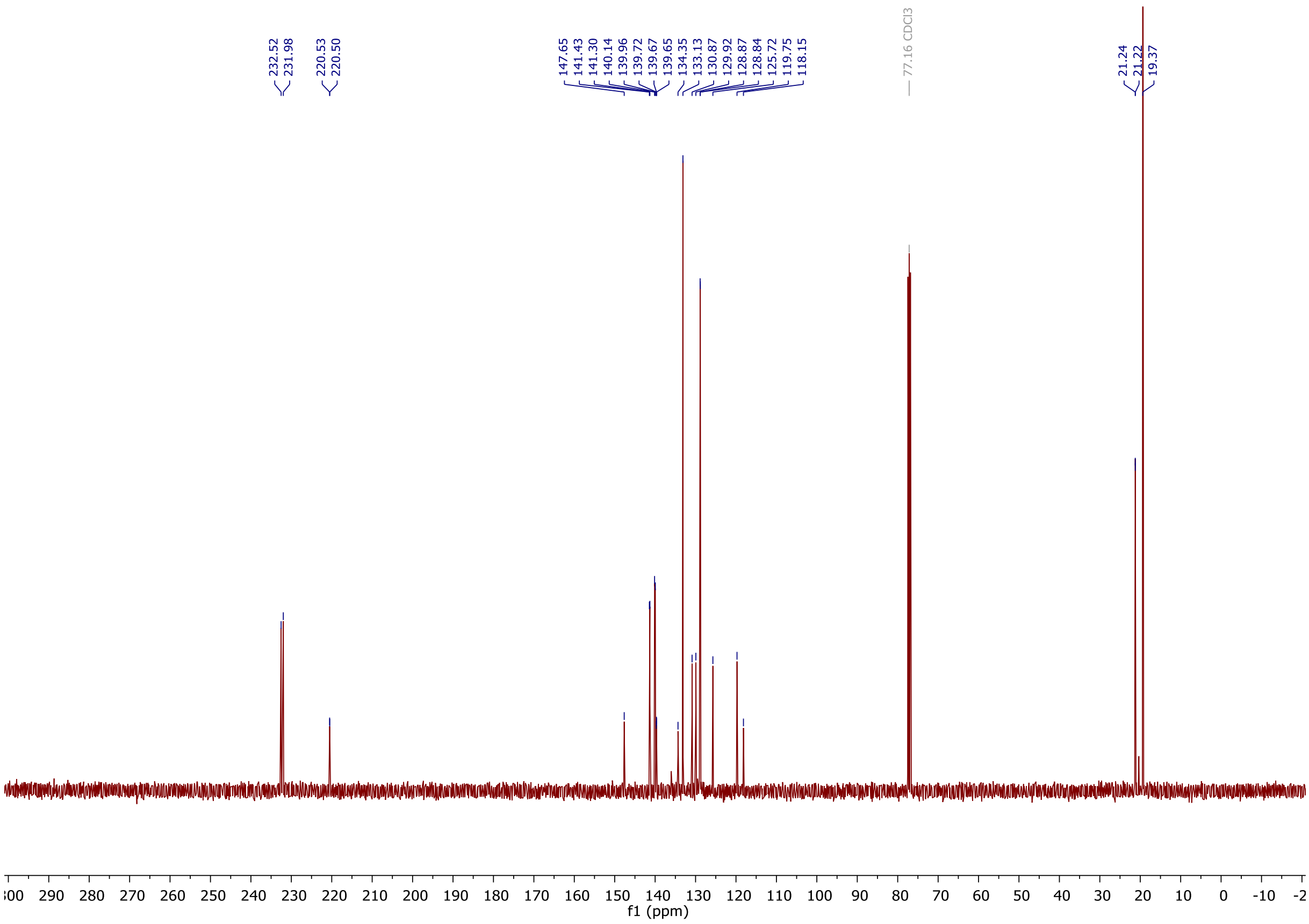

Figure S61. ^{13}C NMR of compound **24** in $CDCl_3$.

Tris(2,4,6-trimethylbenzoyl)((1-(4-(tris(2,4,6-trimethylbenzoyl)germanyl)benzoyl)-1,2,3-triazol-4-yl)methyl)germane (25)

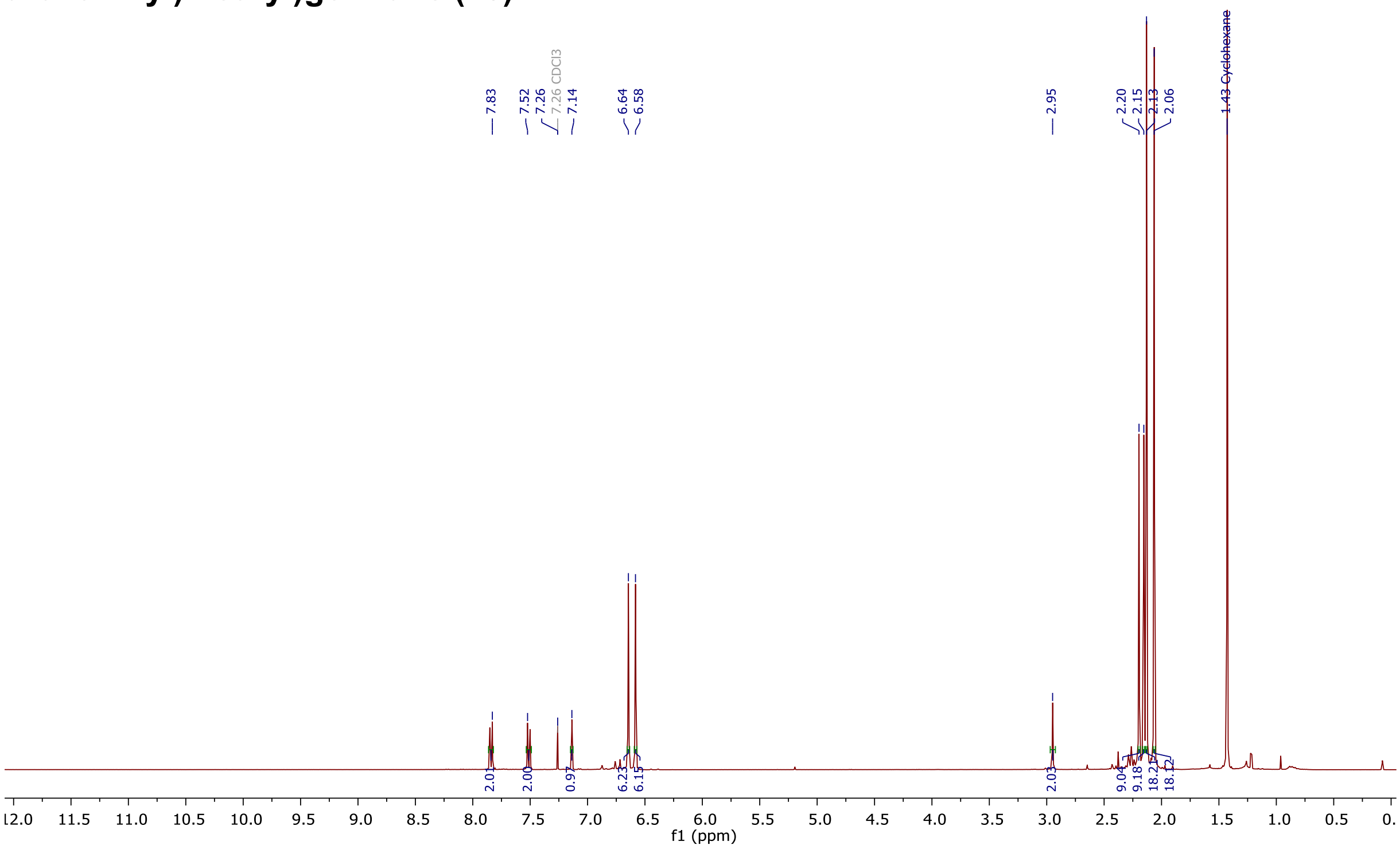

Figure S62. ^1H NMR of compound **25** in $CDCl_3$.

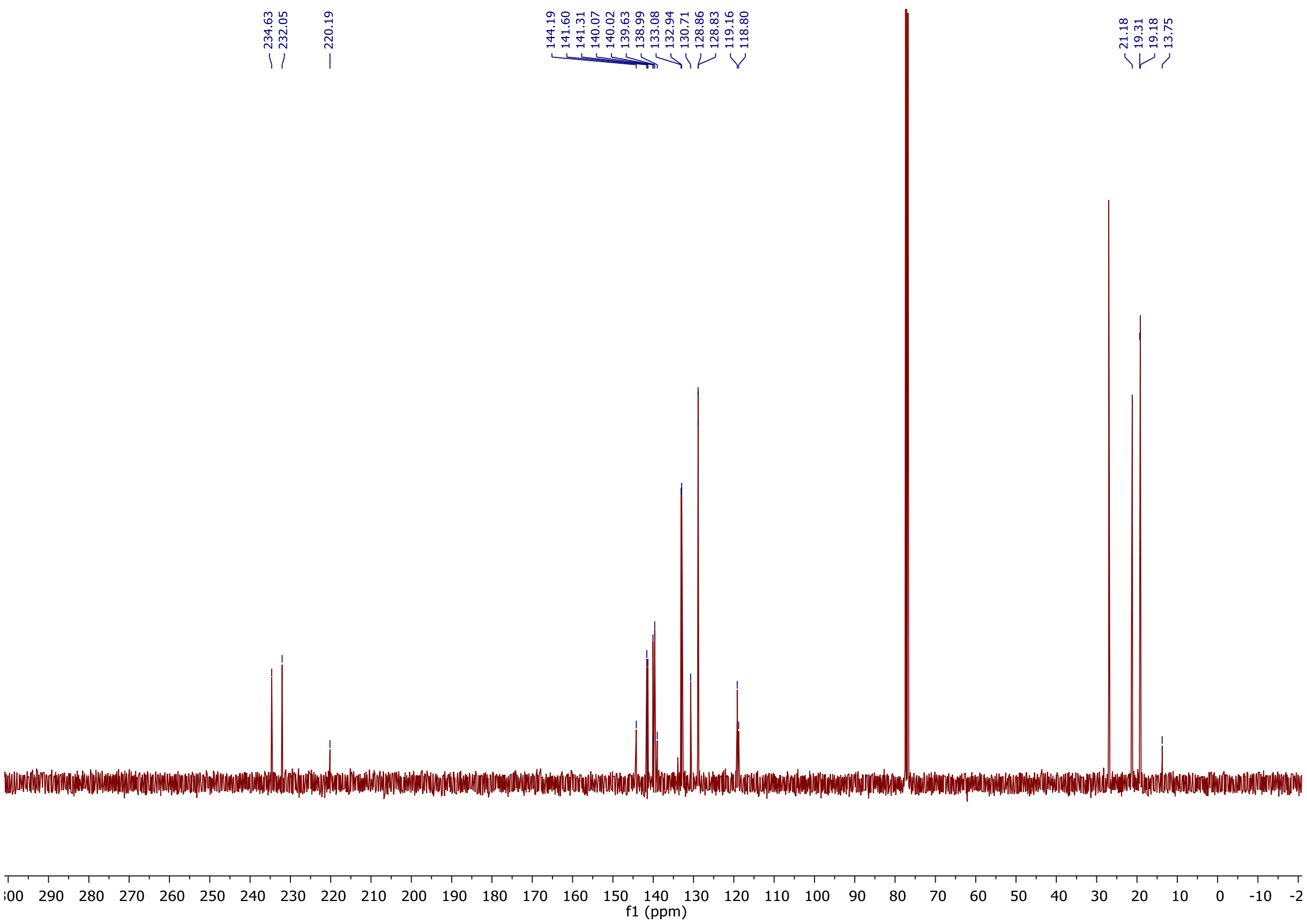

Figure S63. ^{13}C NMR of compound **25** in $CDCl_3$.

Tris(2,4,6-trimethylbenzoyl)((1-(4-(tris(2,4,6-trimethylbenzoyl)germanyl)benzoyl)-1,2,3-triazol-4-yl)butanoyl)germane (26)

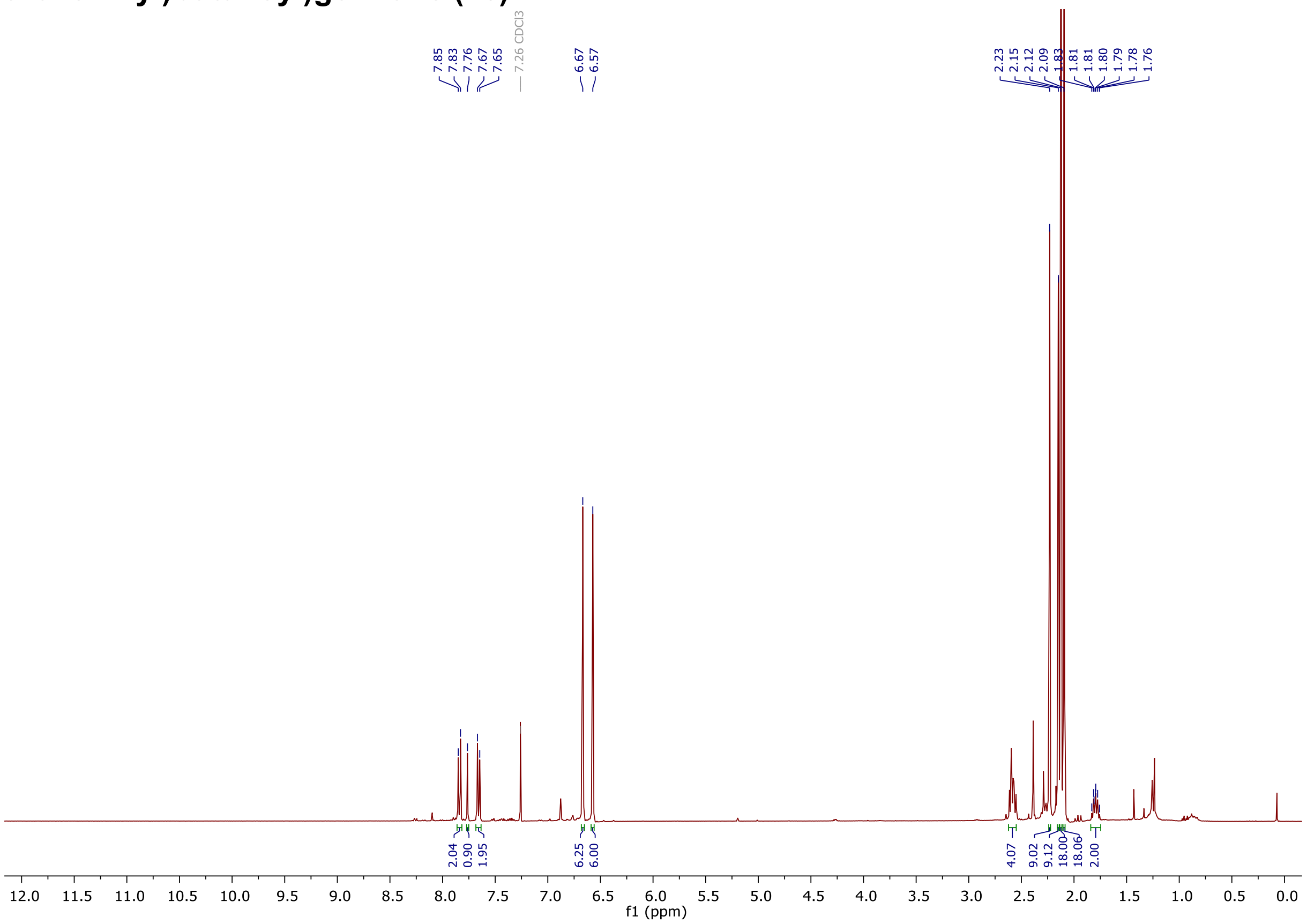

Figure S64. ^{1}H NMR of compound **26** in $CDCl_3$.

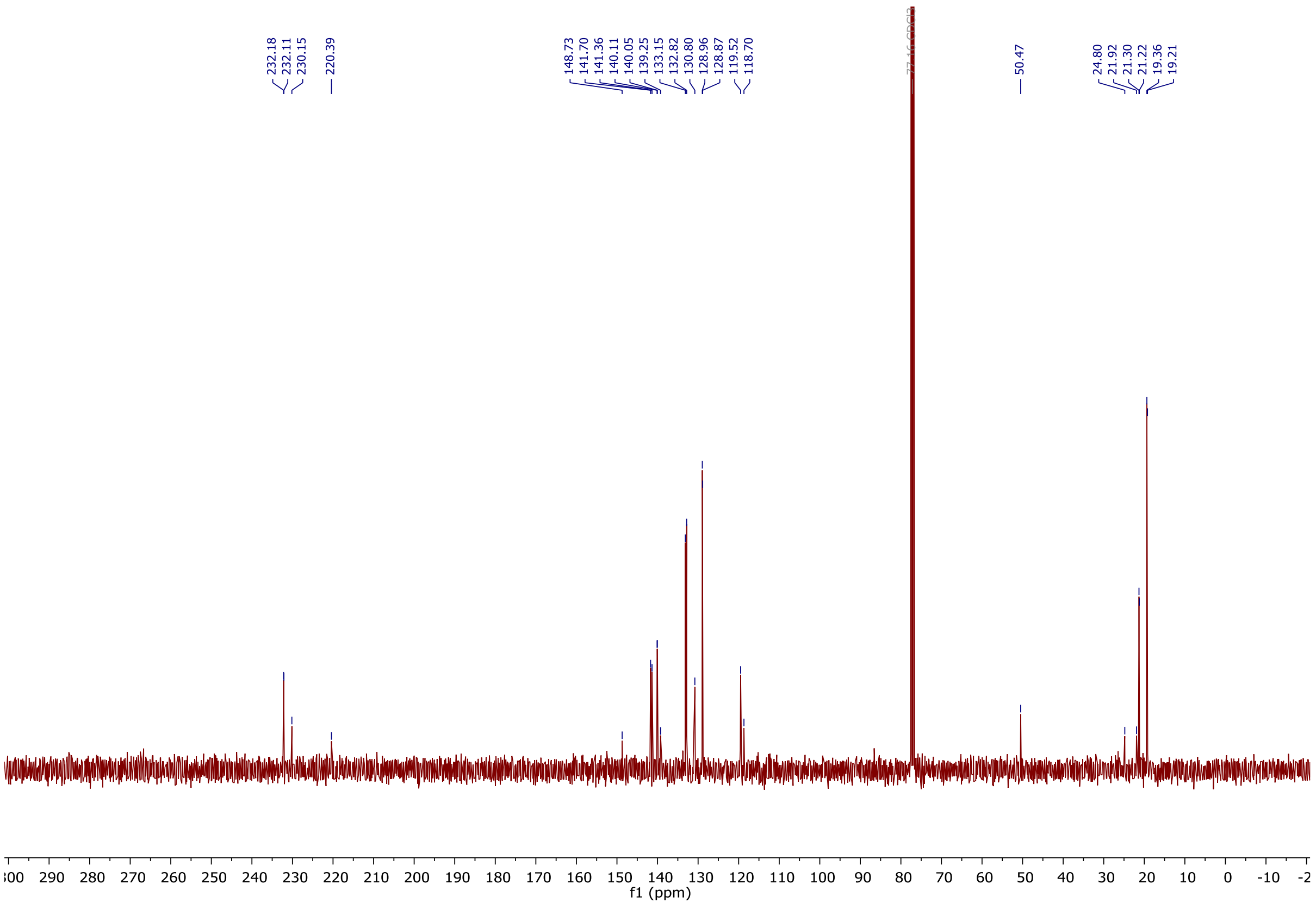

Figure S65. ^{13}C NMR of compound **26** in $CDCl_3$.

UV-Vis Spectroscopy

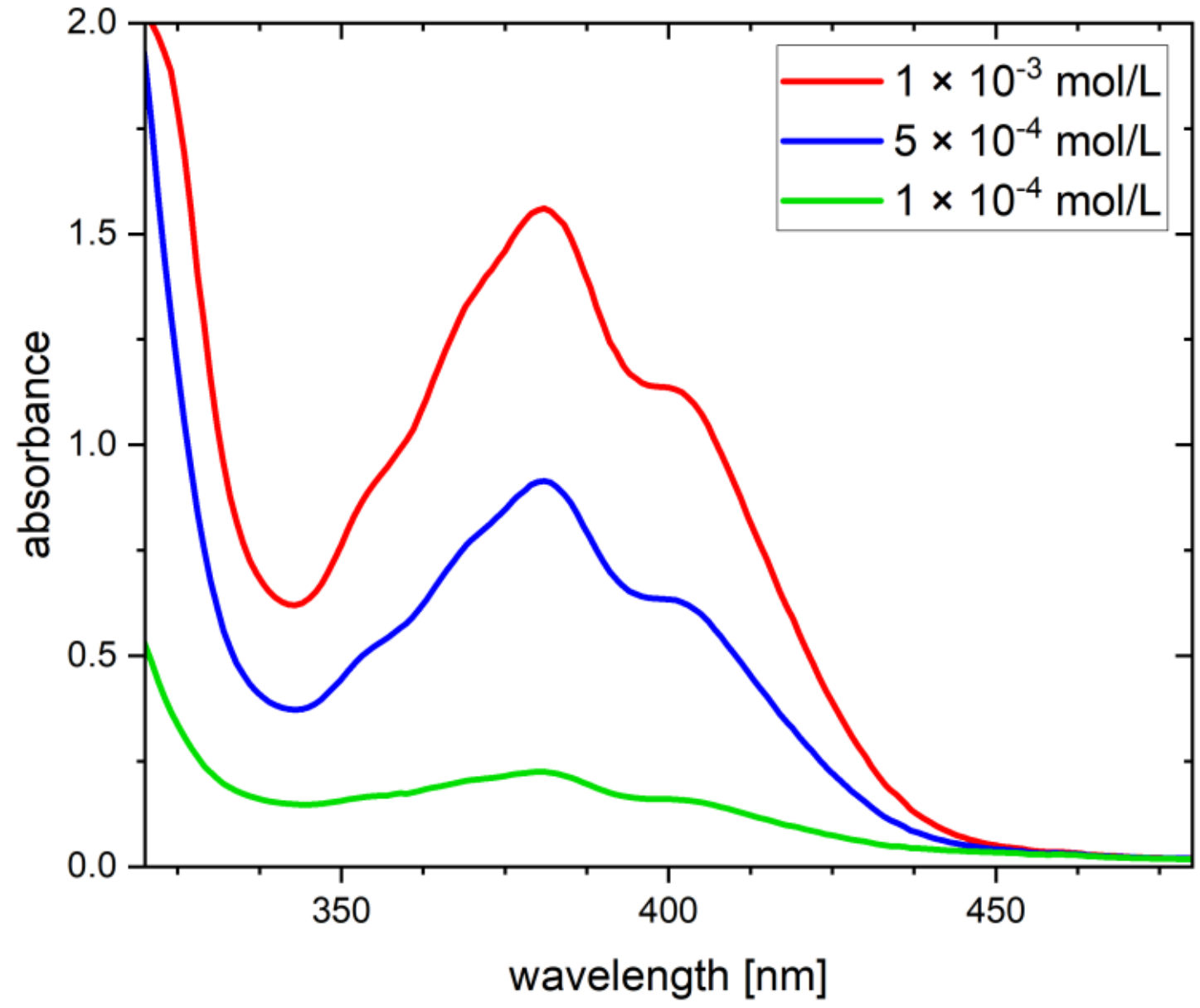

Figure S66. UV-vis spectra of **1** at different concentrations in THF.

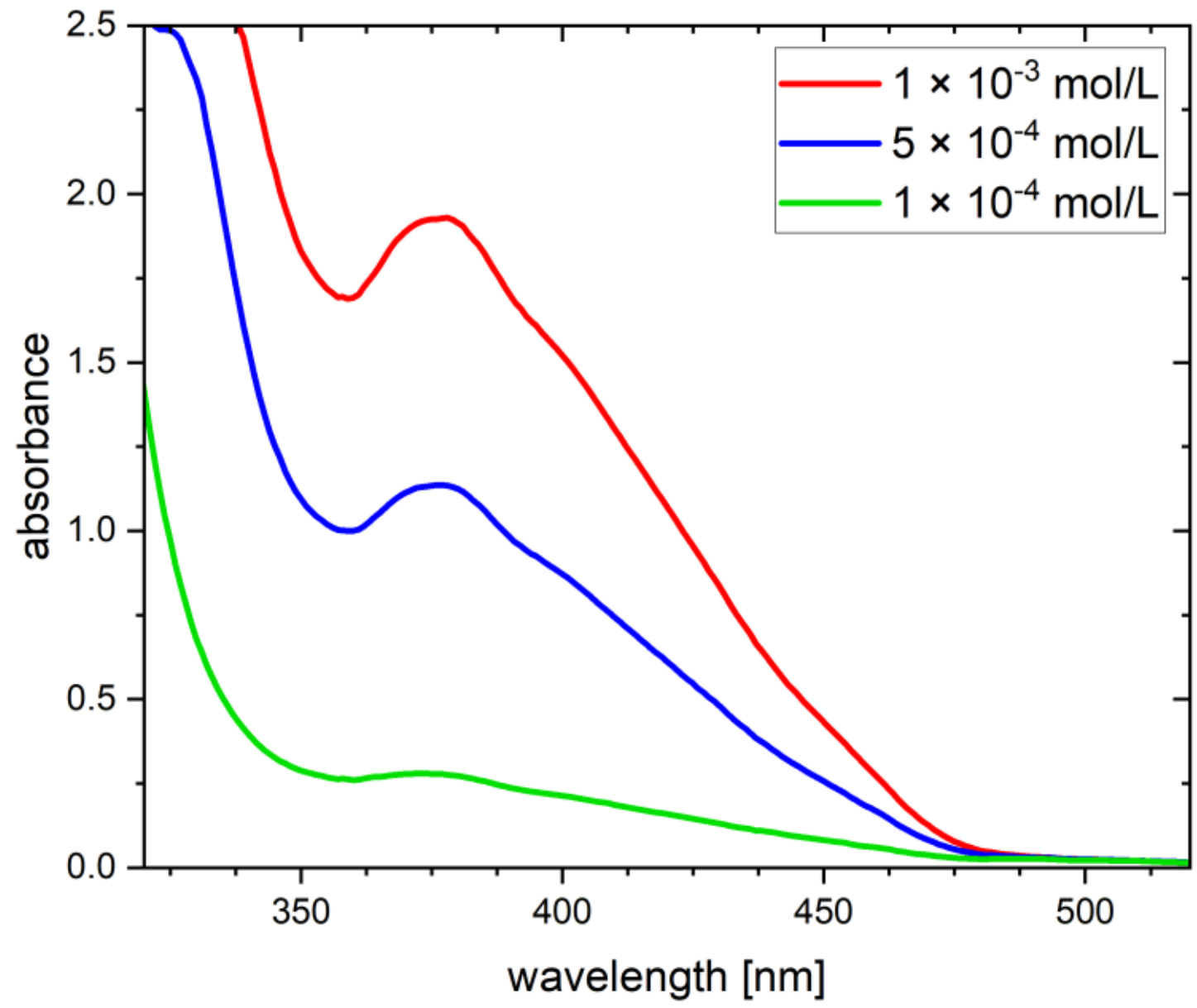

Figure S67. UV-vis spectra of **2** at different concentrations in THF.

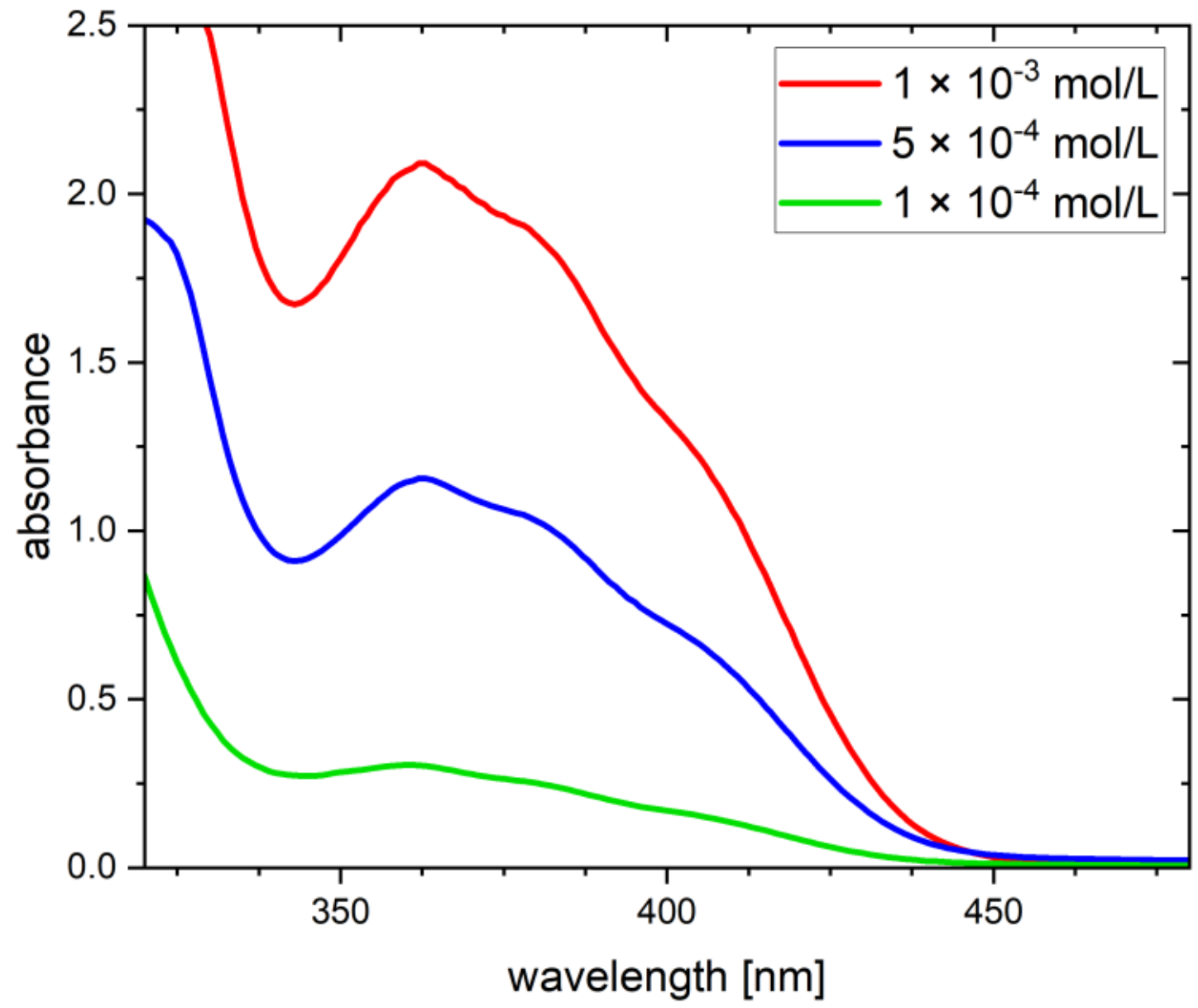

Figure S68. UV-vis spectra of **3** at different concentrations in THF.

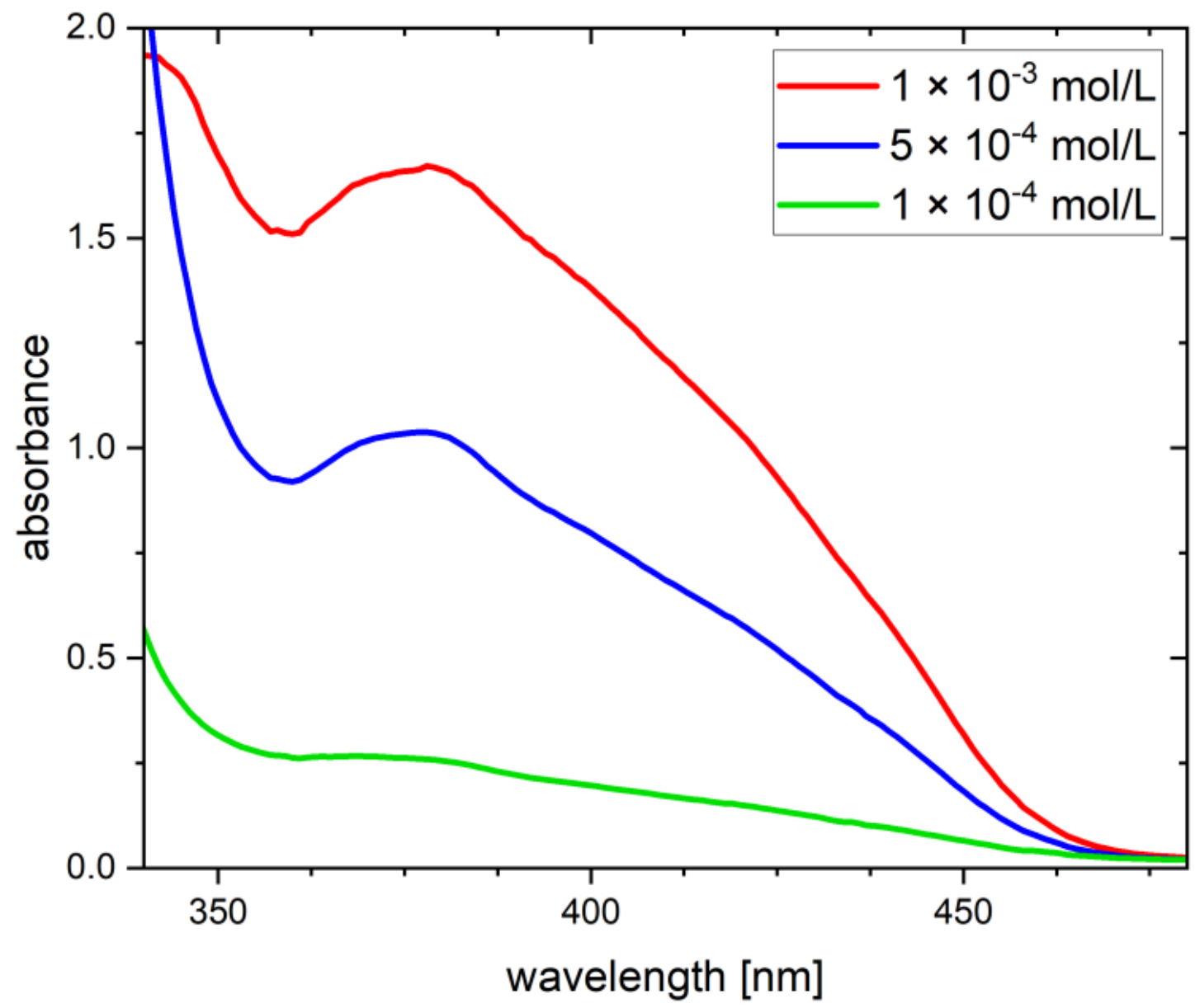

Figure S69. UV-vis spectra of **4** at different concentrations in THF.

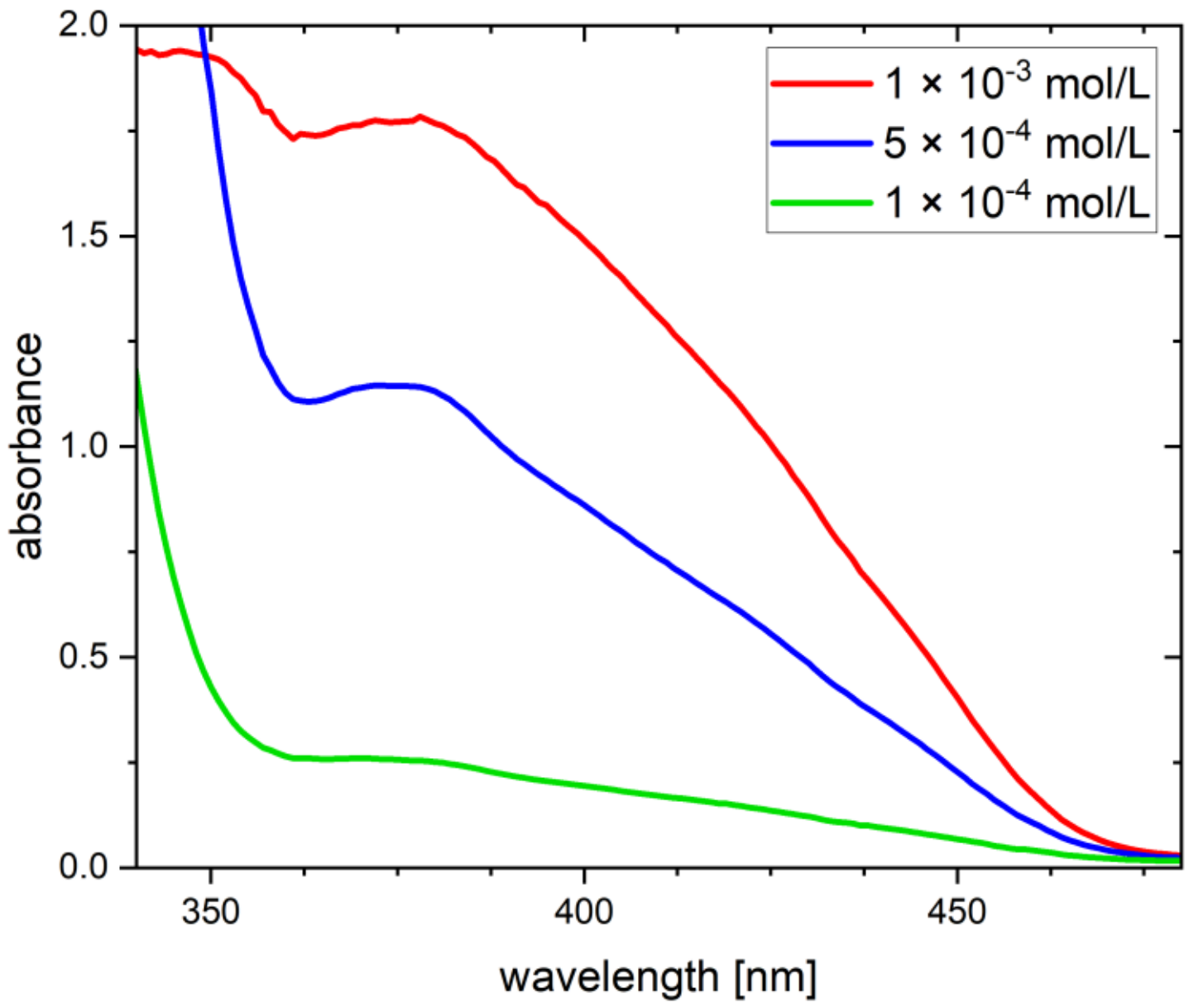

Figure S70. UV-vis spectra of **5** at different concentrations in THF.

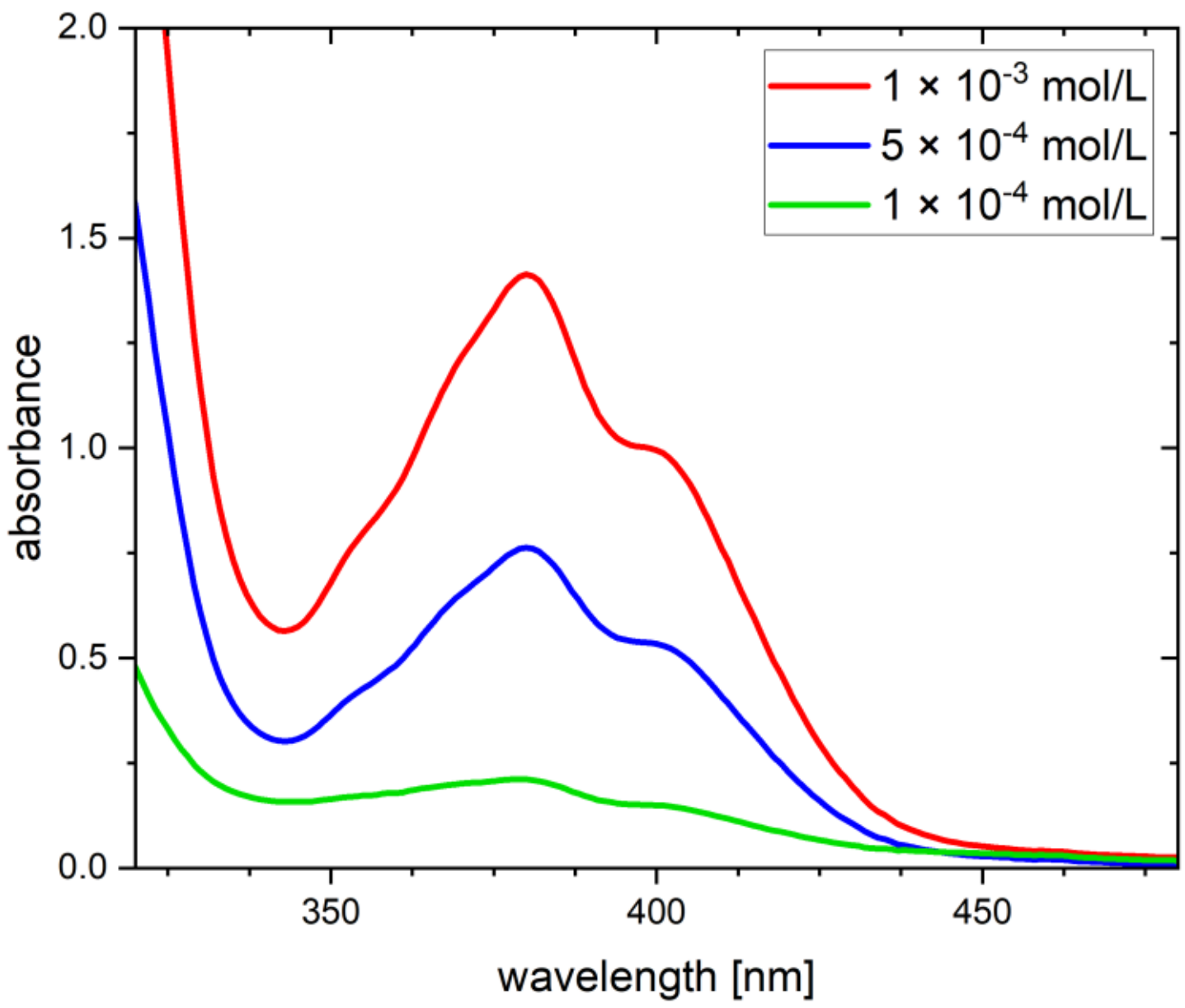

Figure S71. UV-vis spectra of **6** at different concentrations in THF.

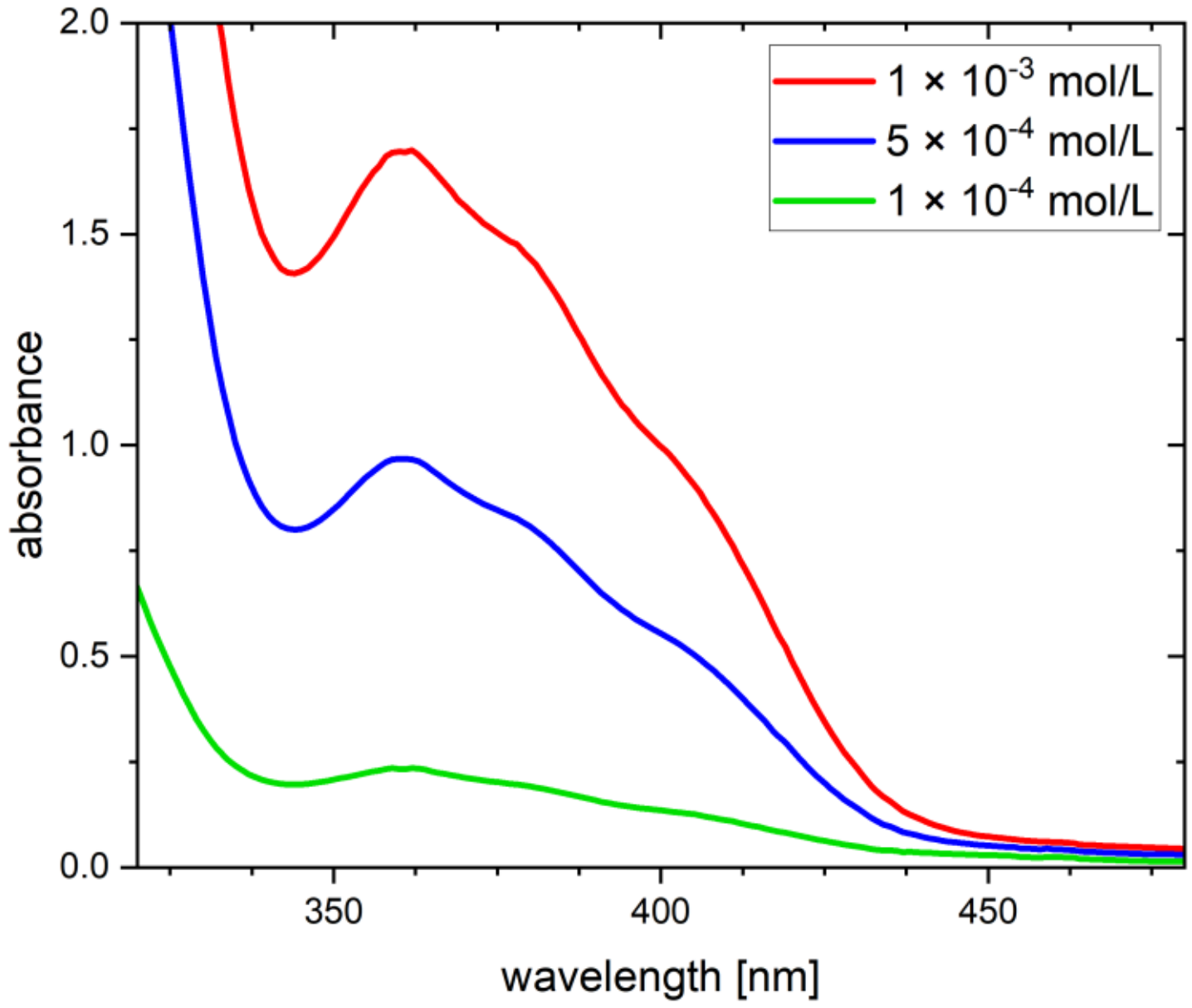

Figure S72. UV-vis spectra of **7** at different concentrations in THF.

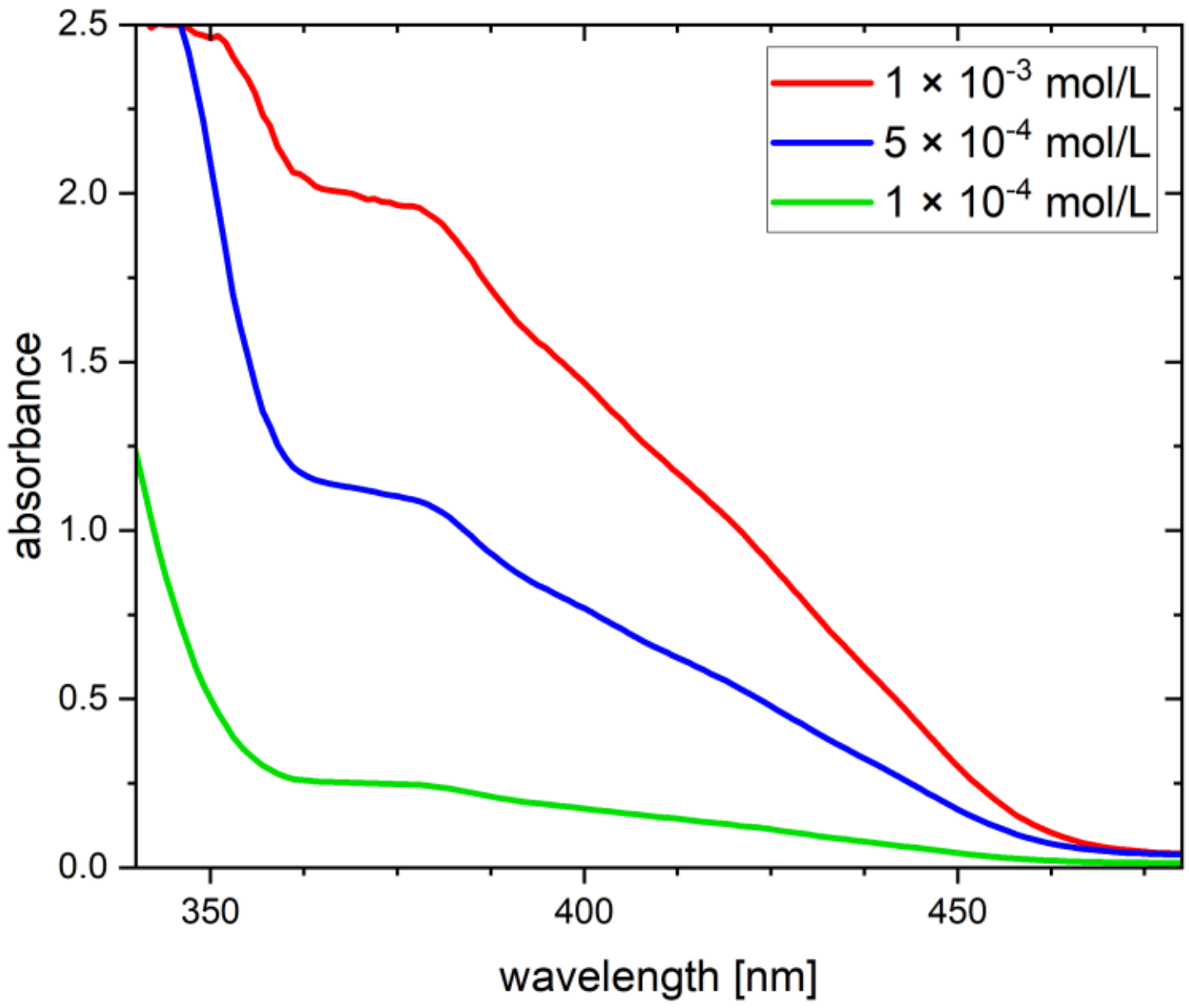

Figure S73. UV-vis spectra of **8** at different concentrations in THF.

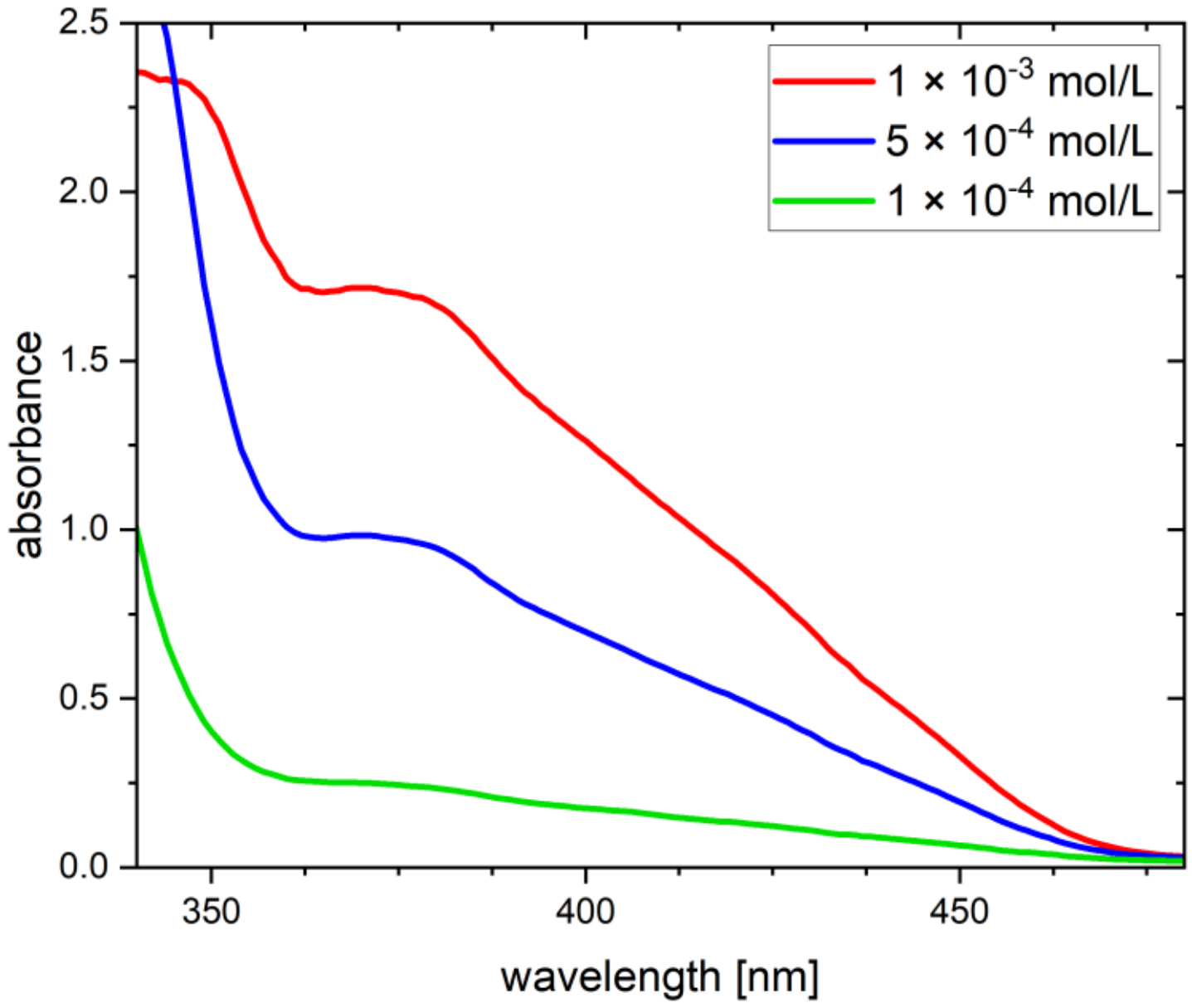

Figure S74. UV-vis spectra of **9** at different concentrations in THF.

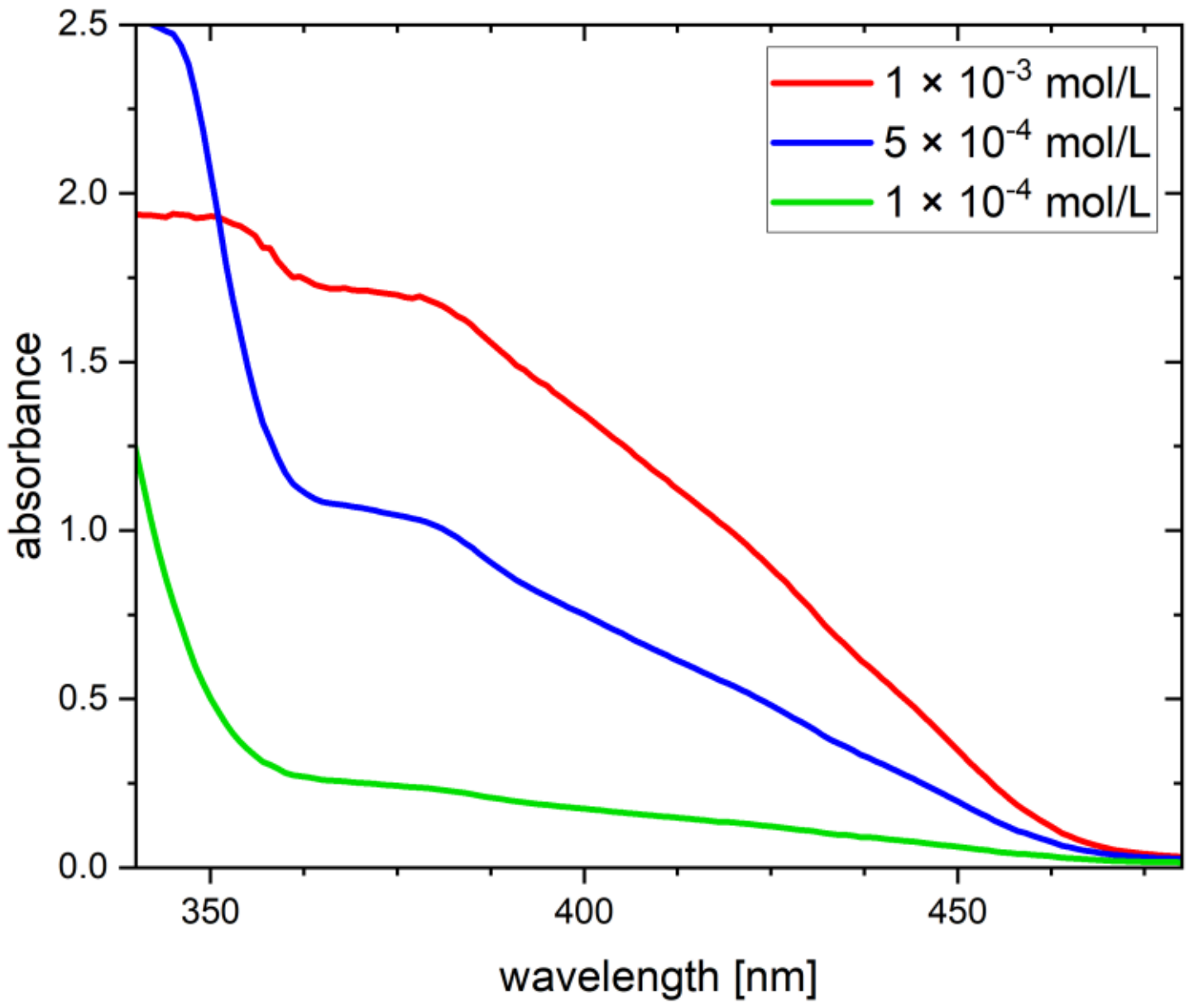

Figure S75. UV-vis spectra of **10** at different concentrations in THF.

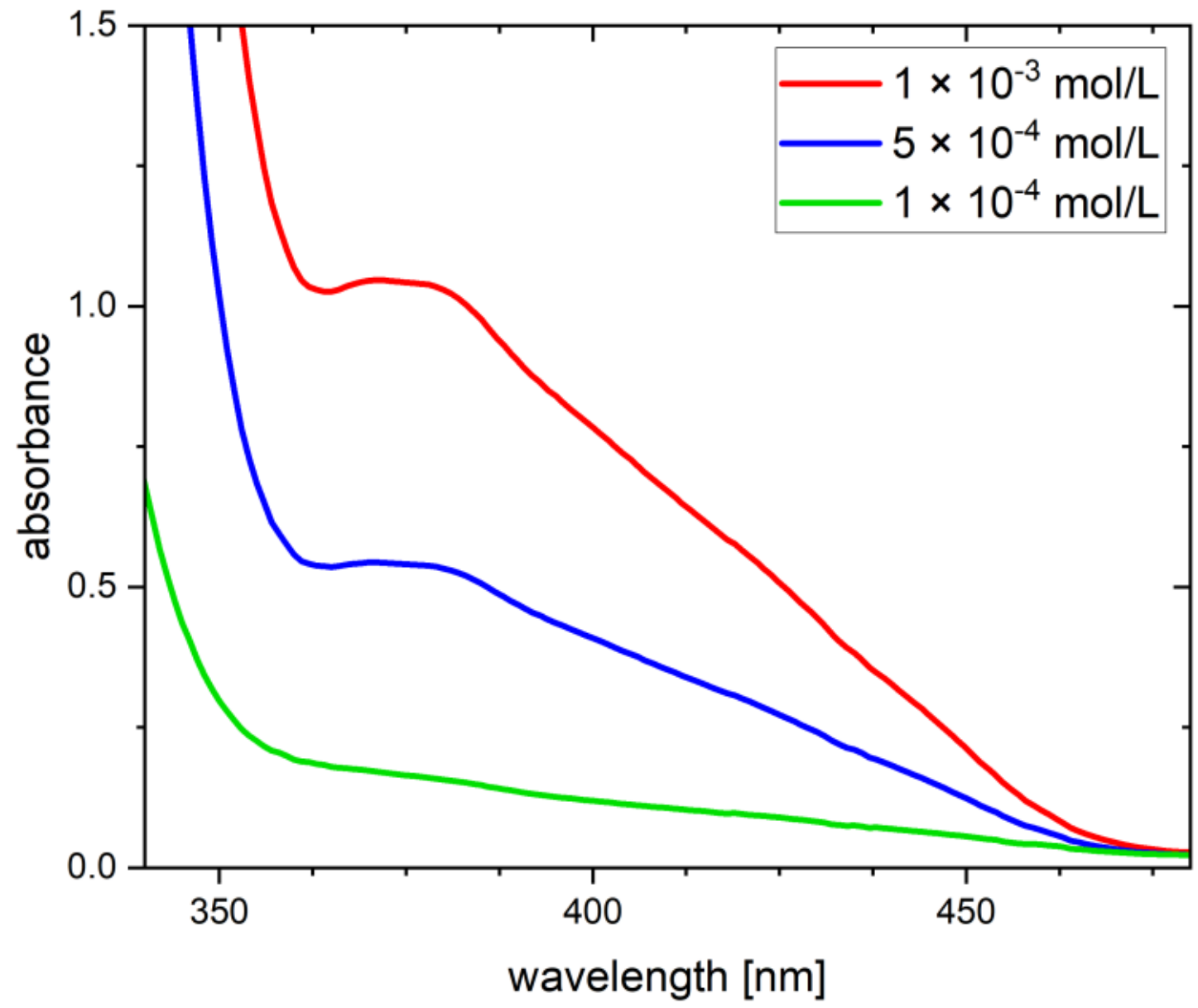

Figure S76. UV-vis spectra of **11** at different concentrations in THF.

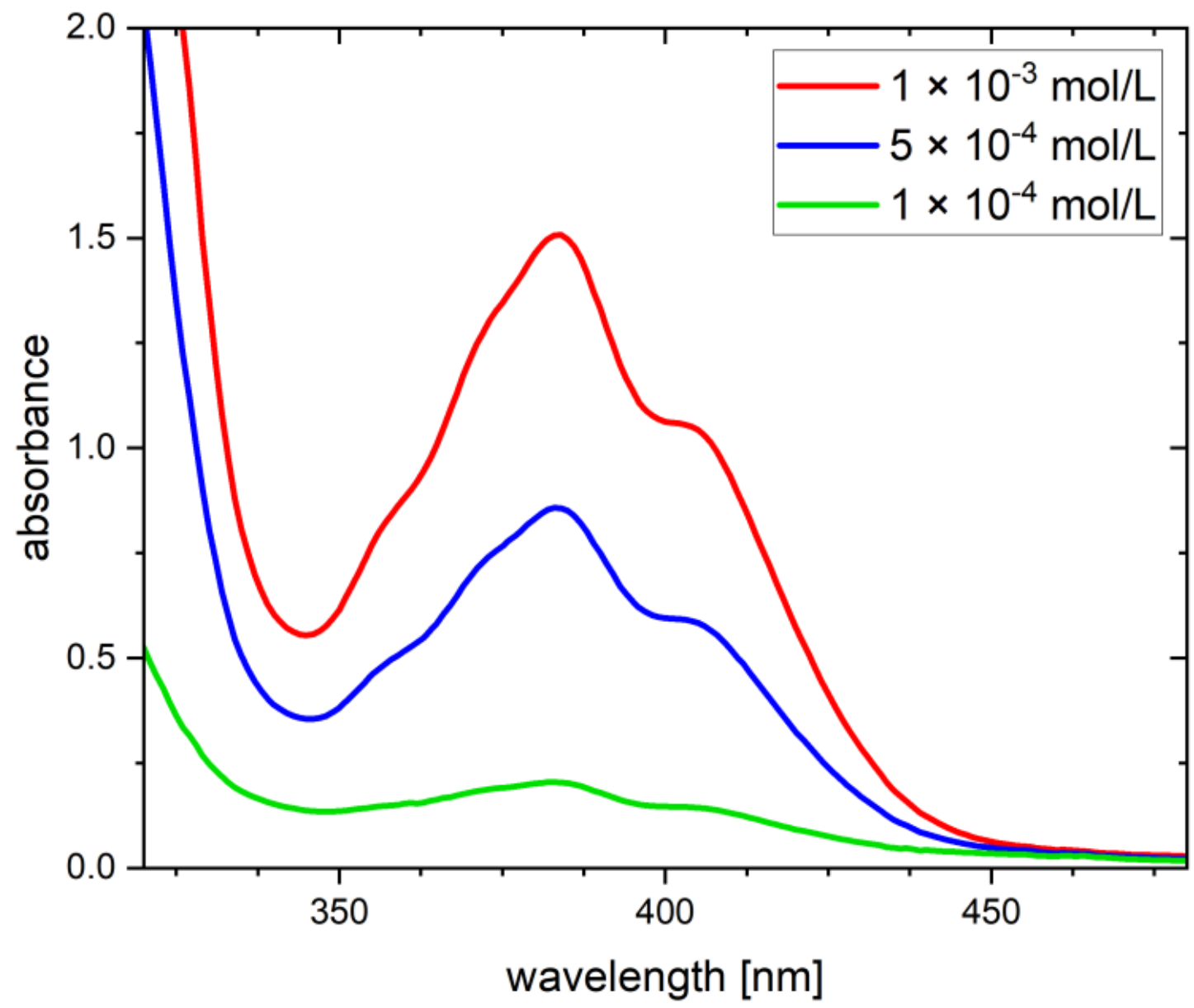

Figure S77. UV-vis spectra of **12** at different concentrations in THF.

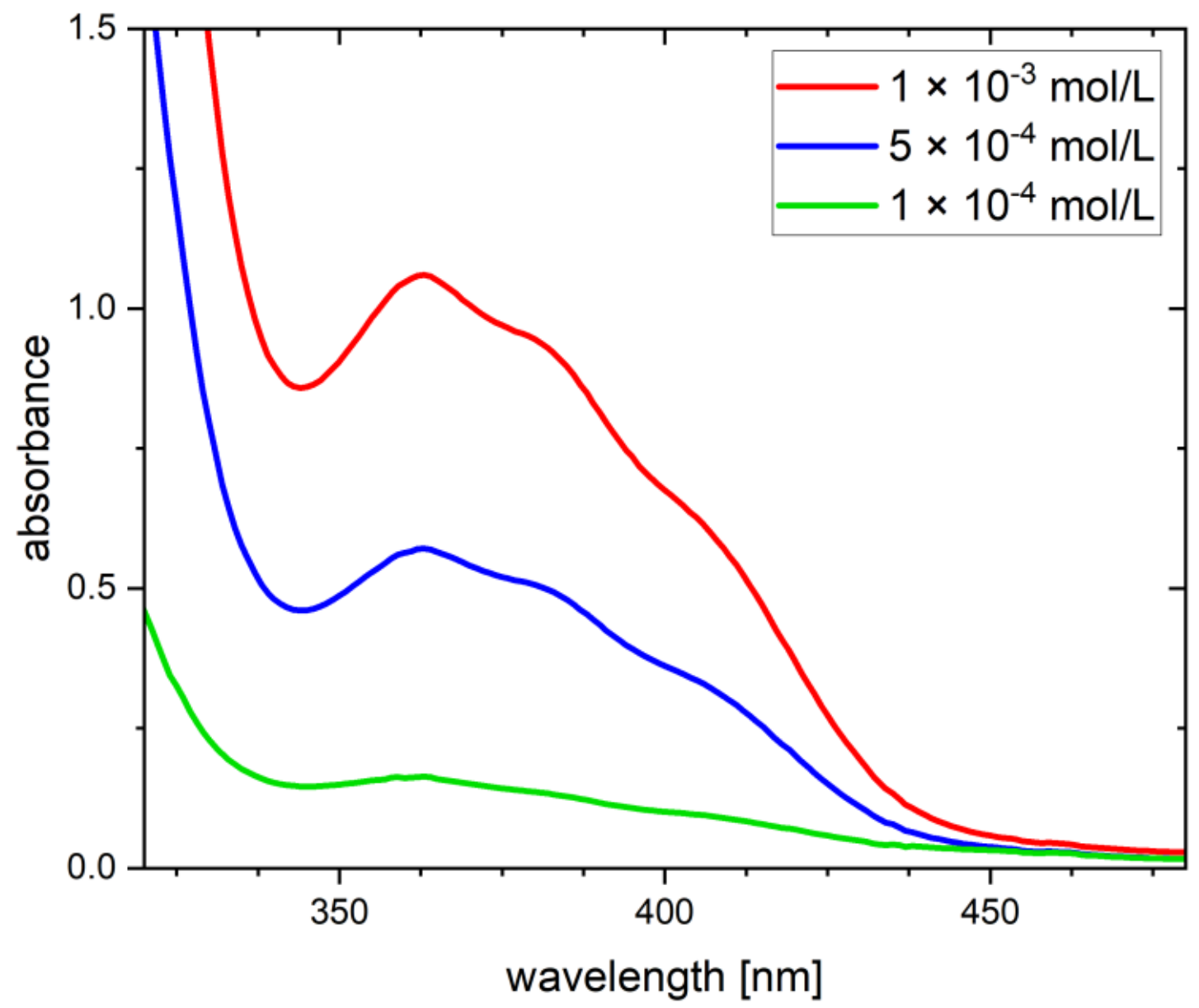

Figure S78. UV-vis spectra of **13** at different concentrations in THF.

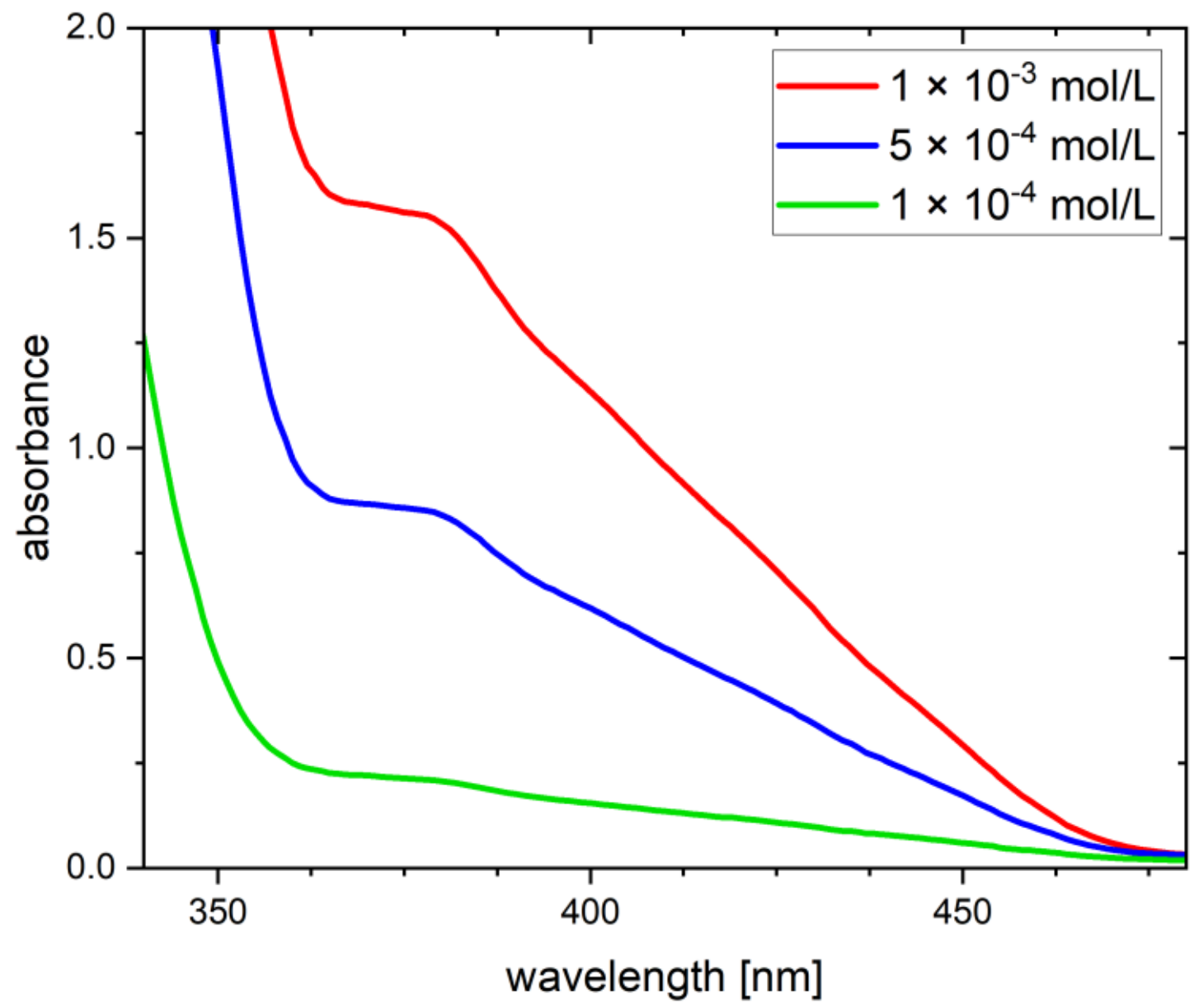

Figure S79. UV-vis spectra of **14** at different concentrations in THF.

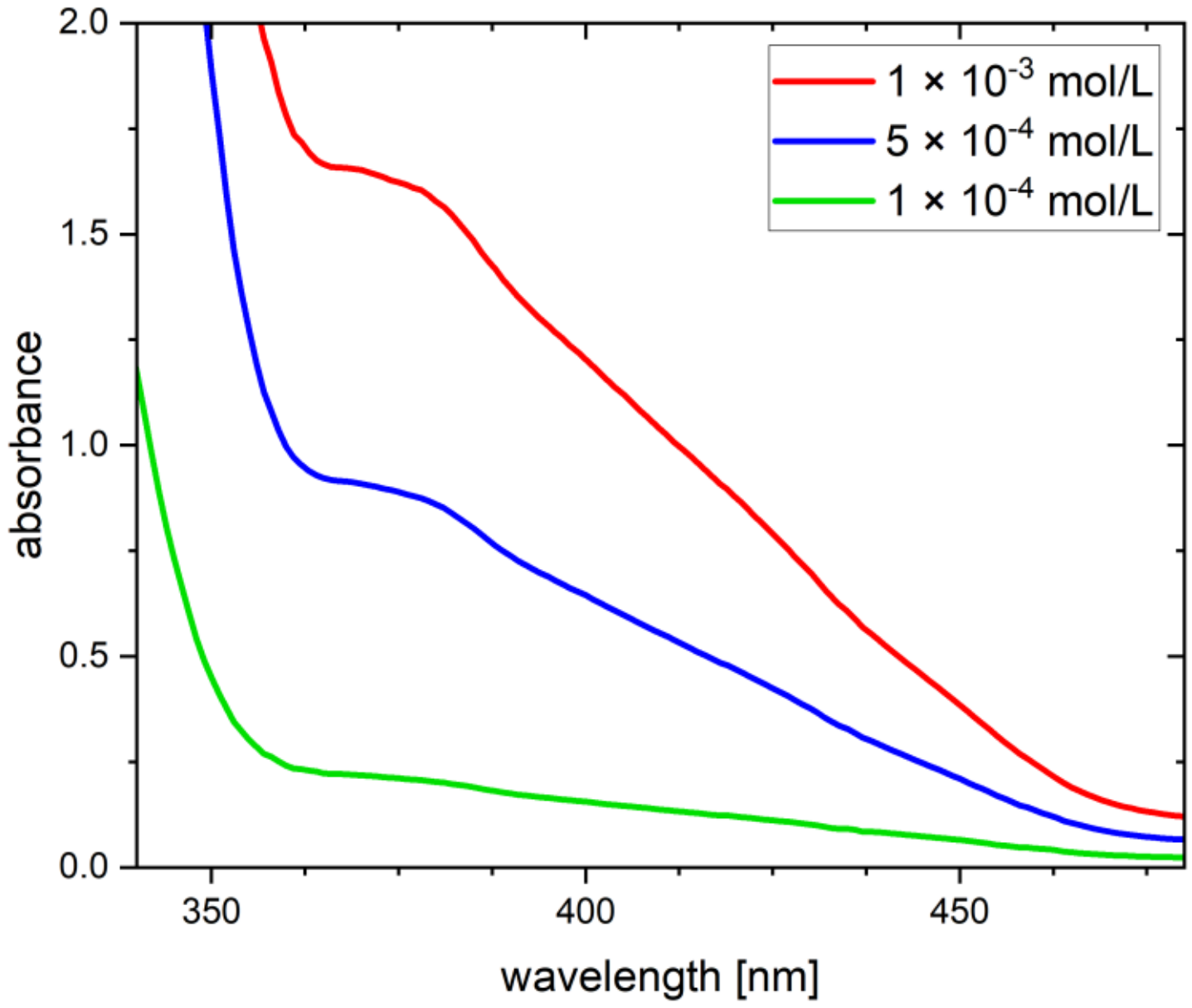

Figure S80. UV-vis spectra of **15** at different concentrations in THF.

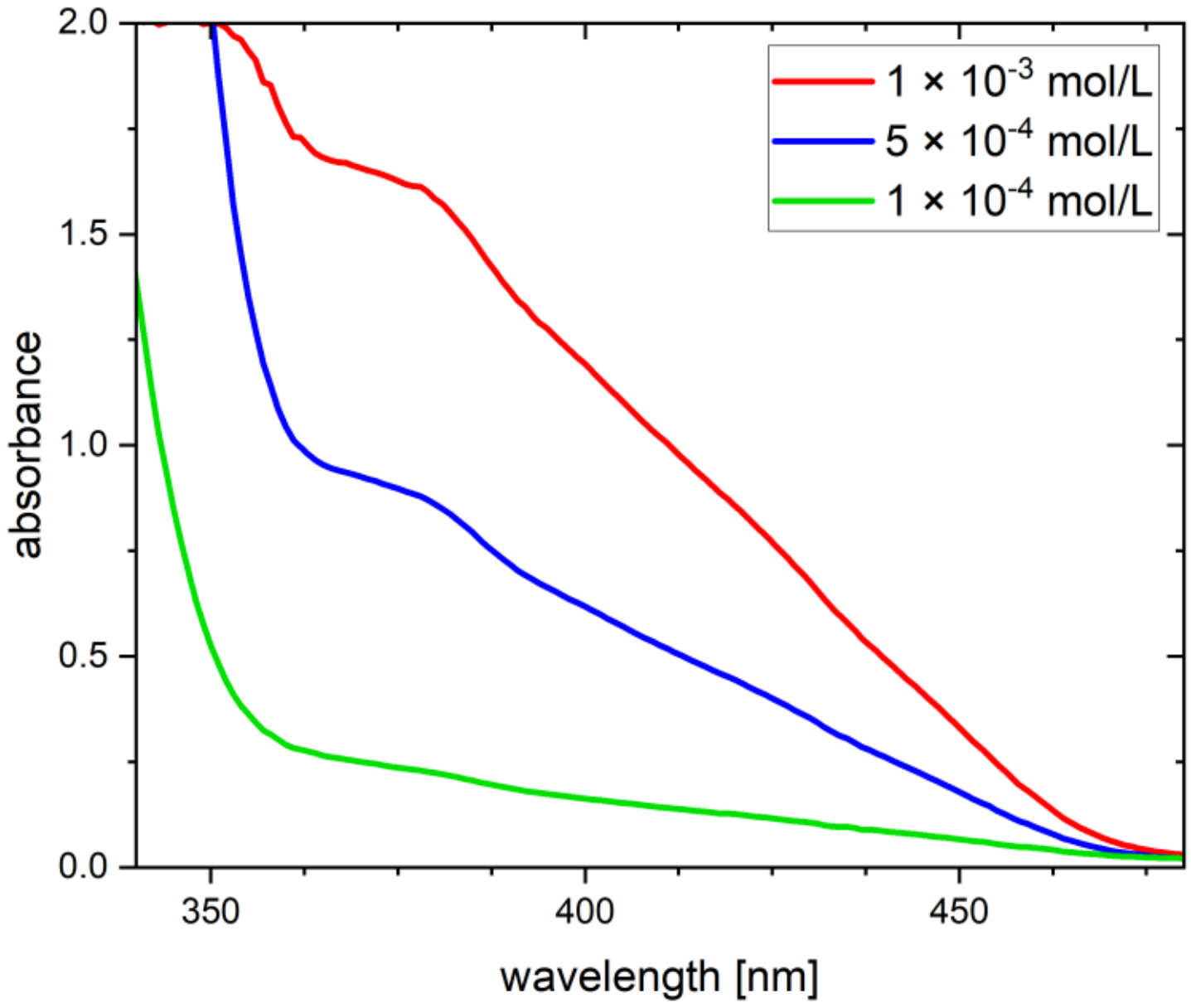

Figure S81. UV-vis spectra of **16** at different concentrations in THF.

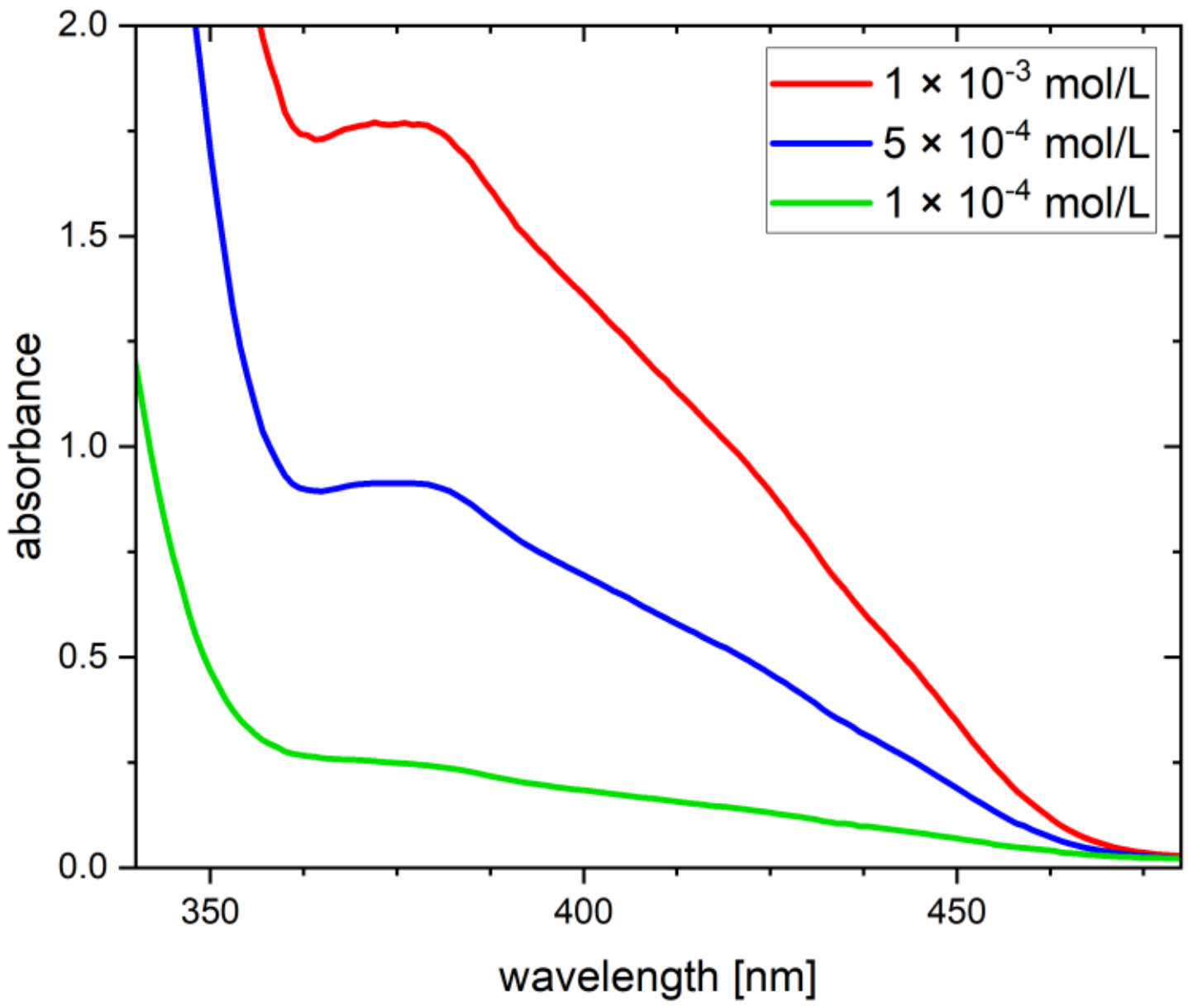

Figure S82. UV-vis spectra of **17** at different concentrations in THF.

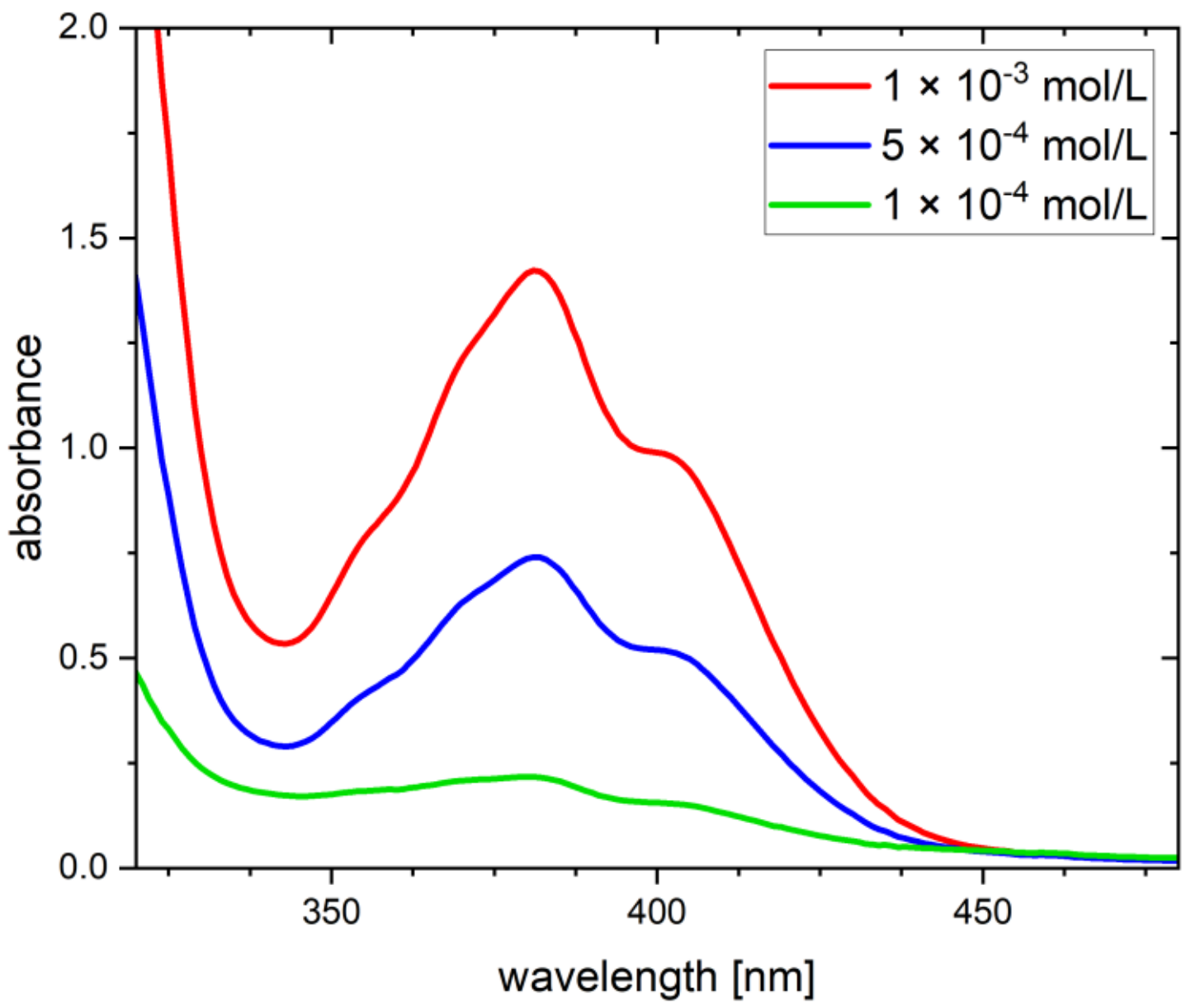

Figure S83. UV-vis spectra of **18** at different concentrations in THF.

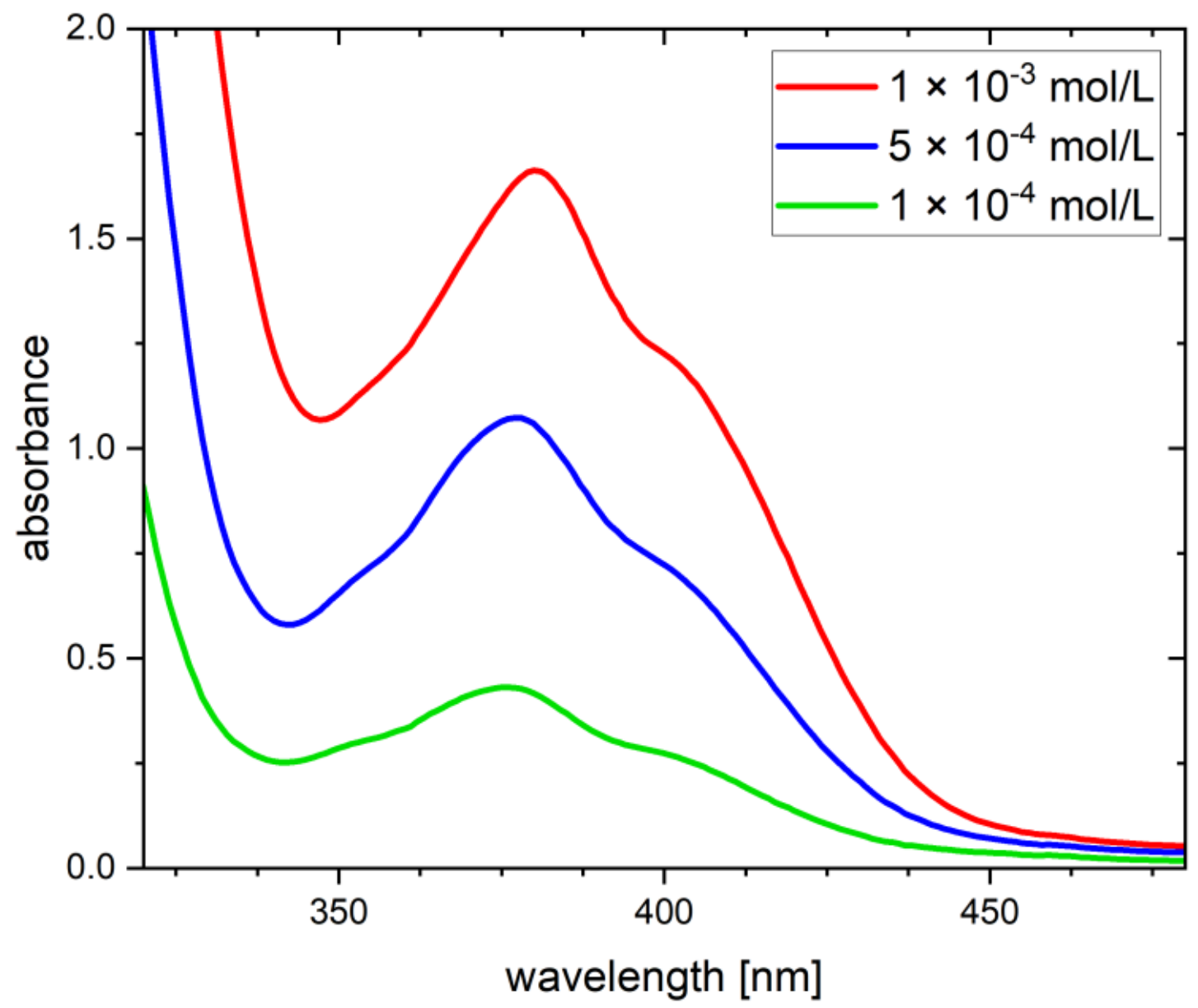

Figure S84. UV-vis spectra of **19** at different concentrations in THF.

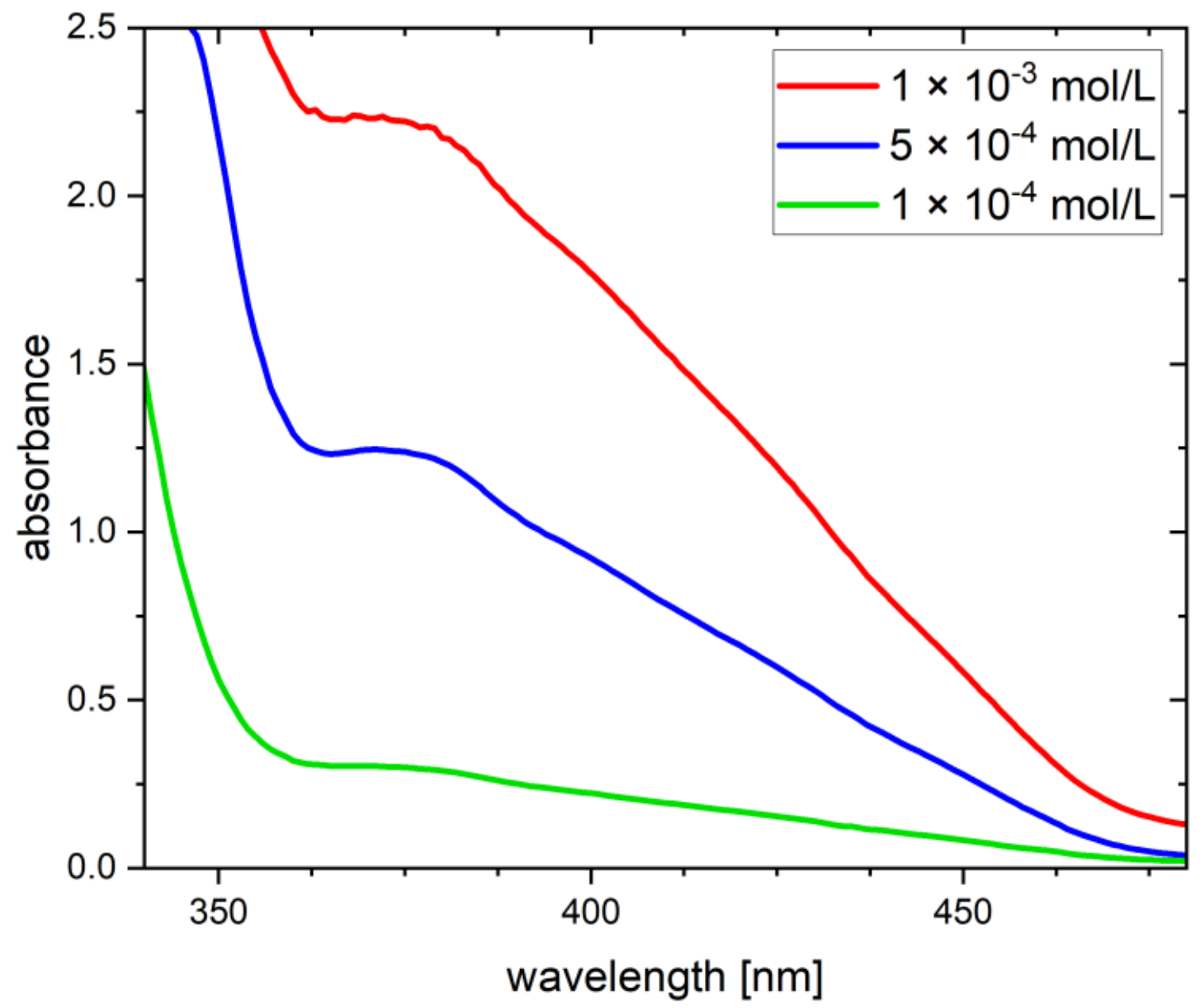

Figure S85. UV-vis spectra of **20** at different concentrations in THF.

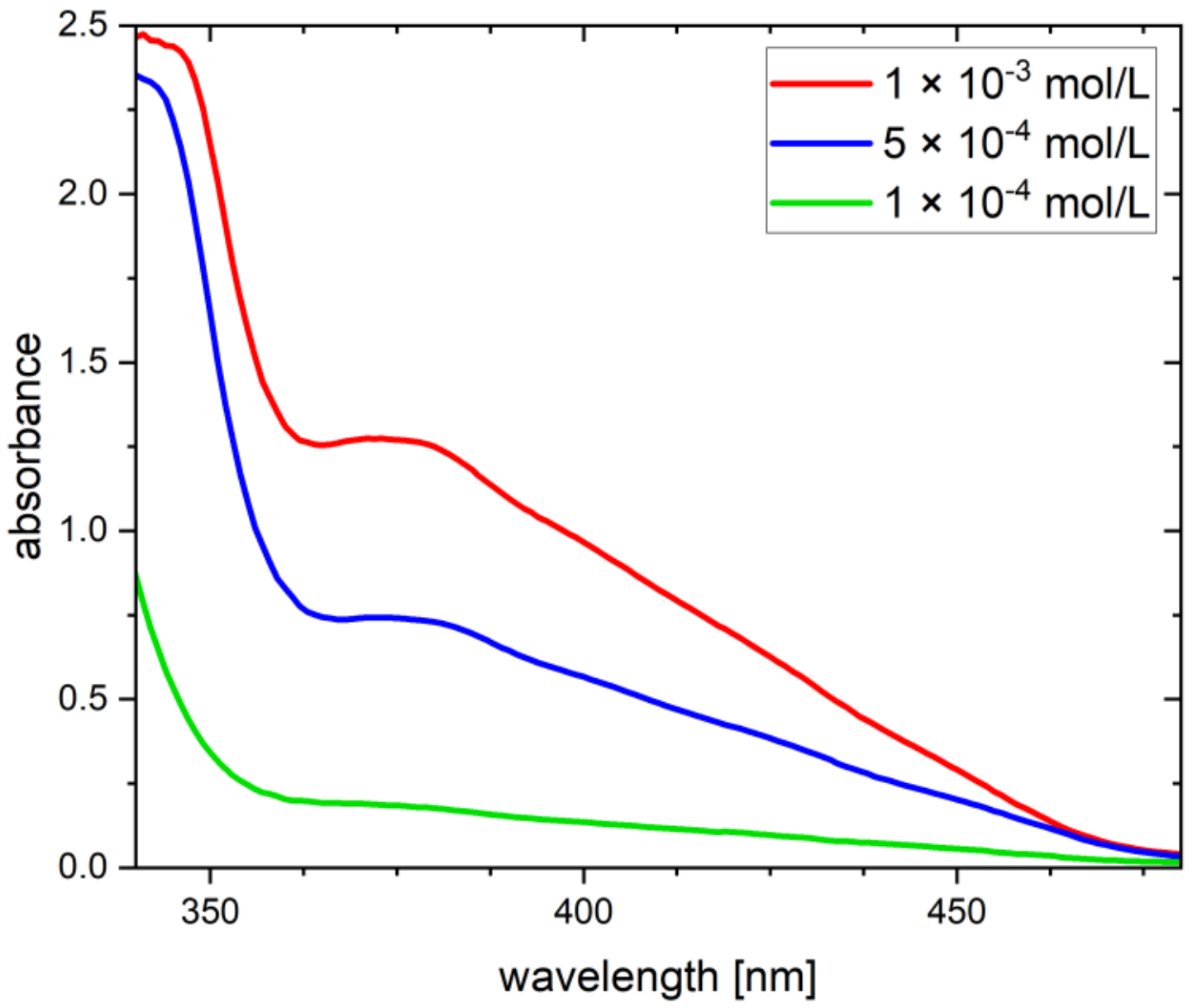

Figure S86. UV-vis spectra of **21** at different concentrations in THF.

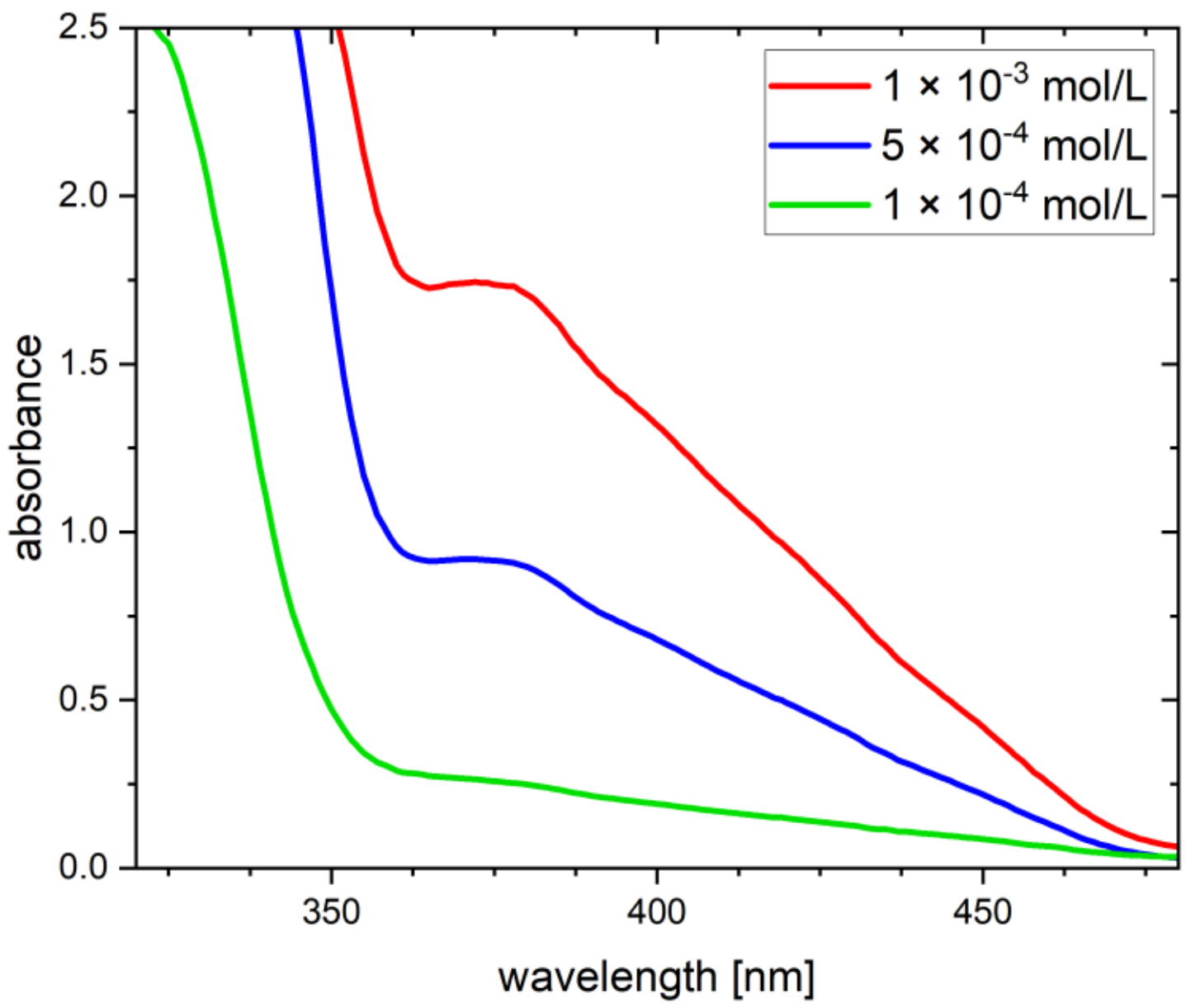

Figure S87. UV-vis spectra of **22** at different concentrations in THF.

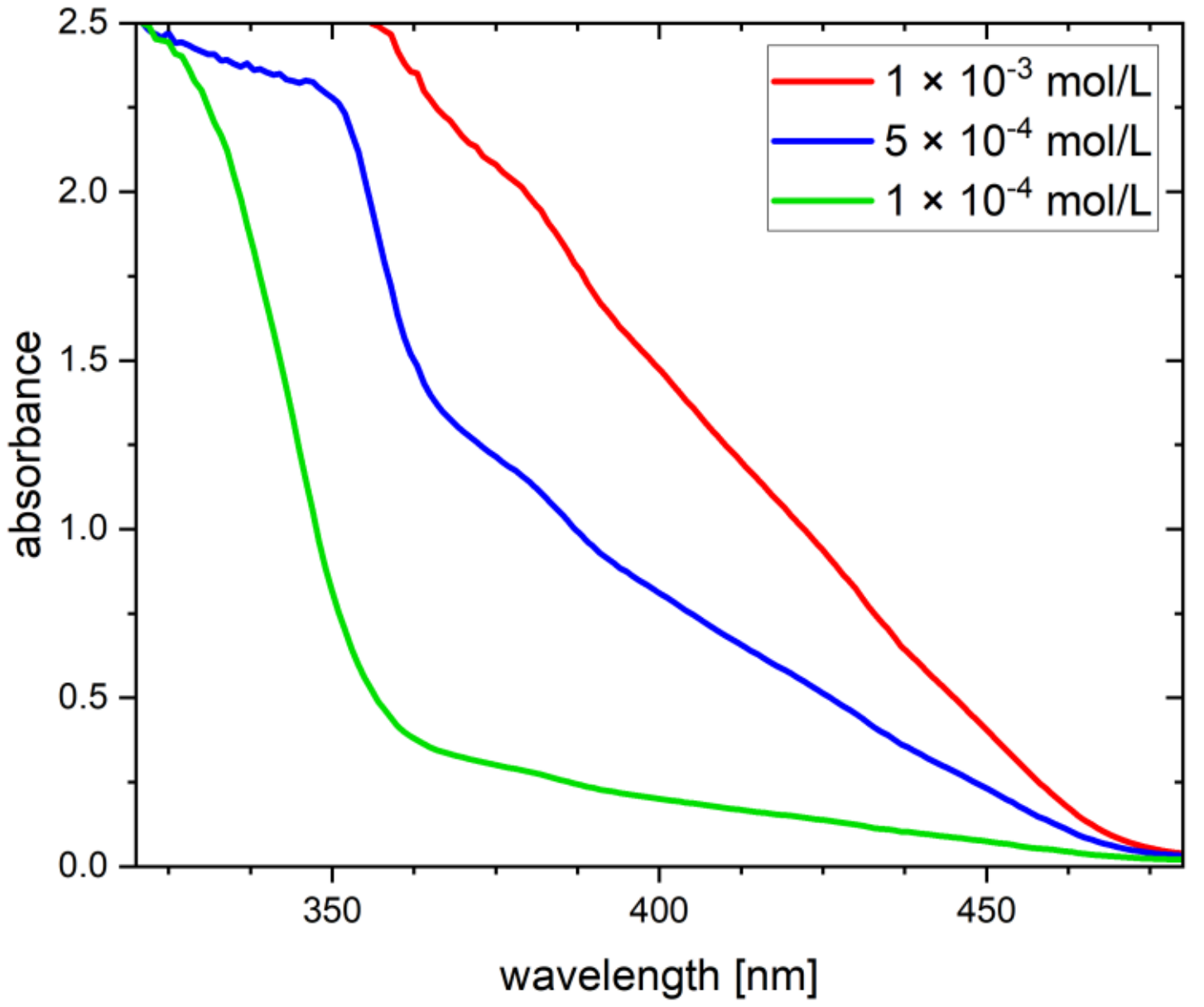

Figure S88. UV-vis spectra of **23** at different concentrations in THF.

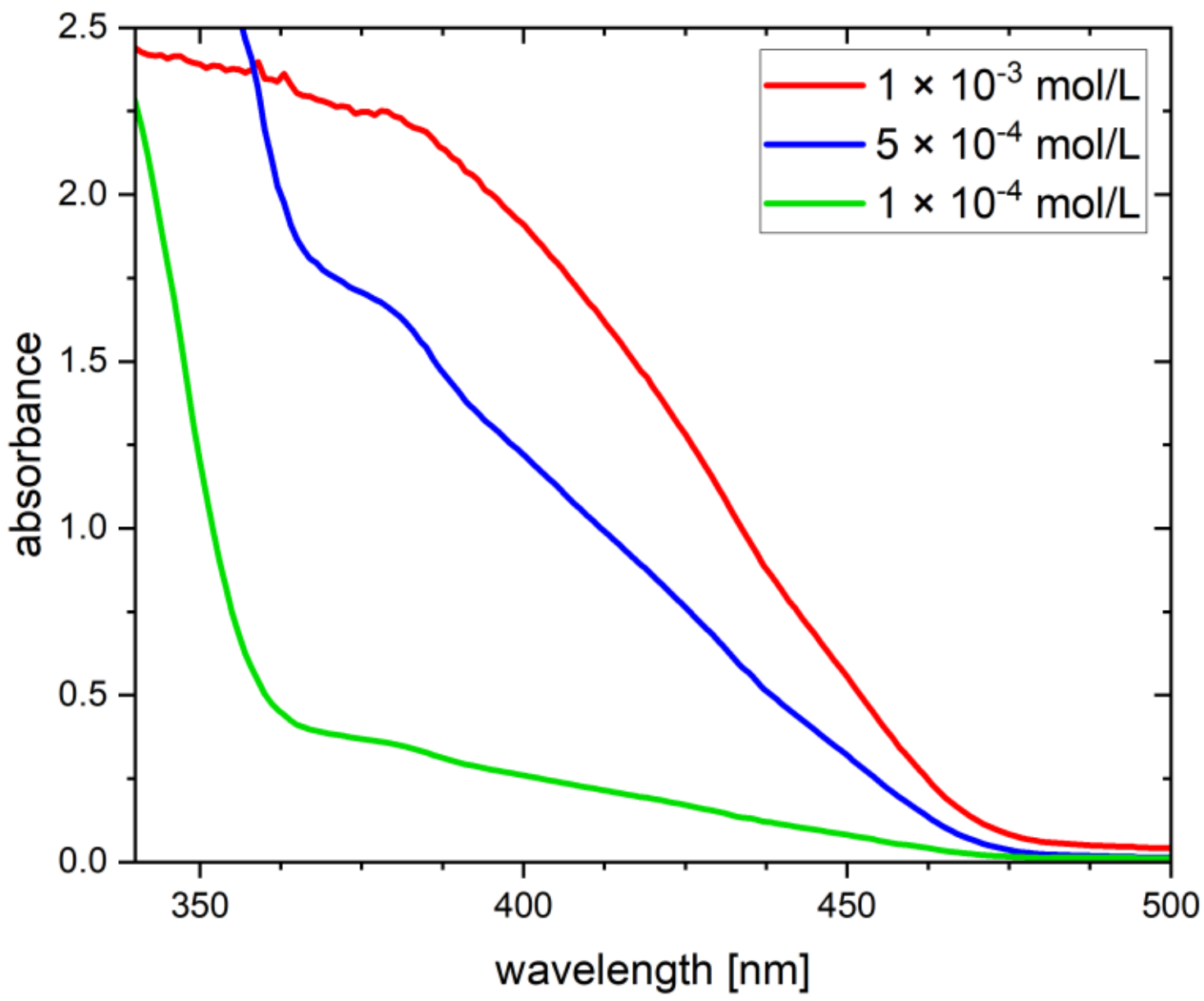

Figure S89. UV-vis spectra of **24** at different concentrations in THF.

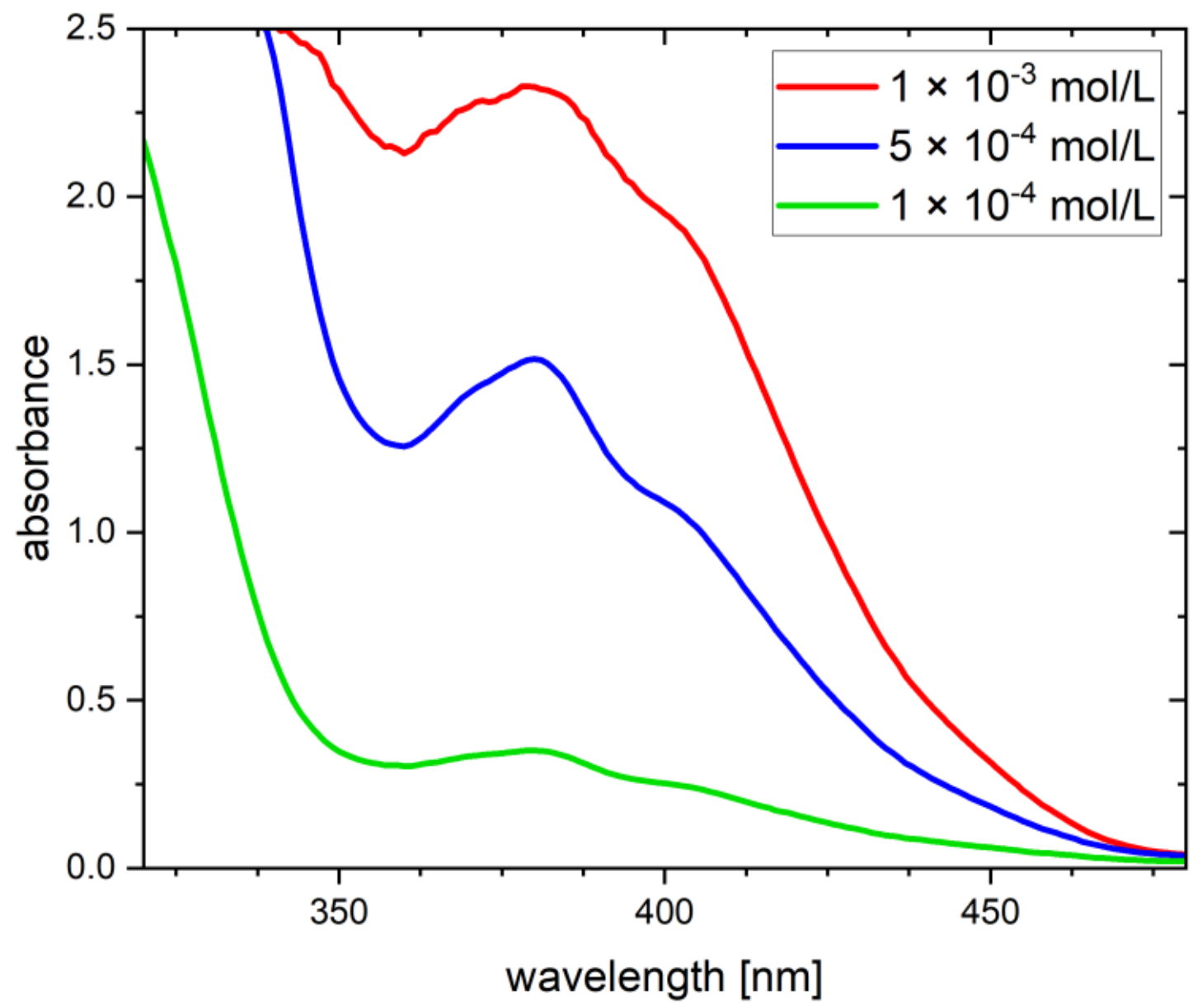

Figure S90. UV-vis spectra of **25** at different concentrations in THF.

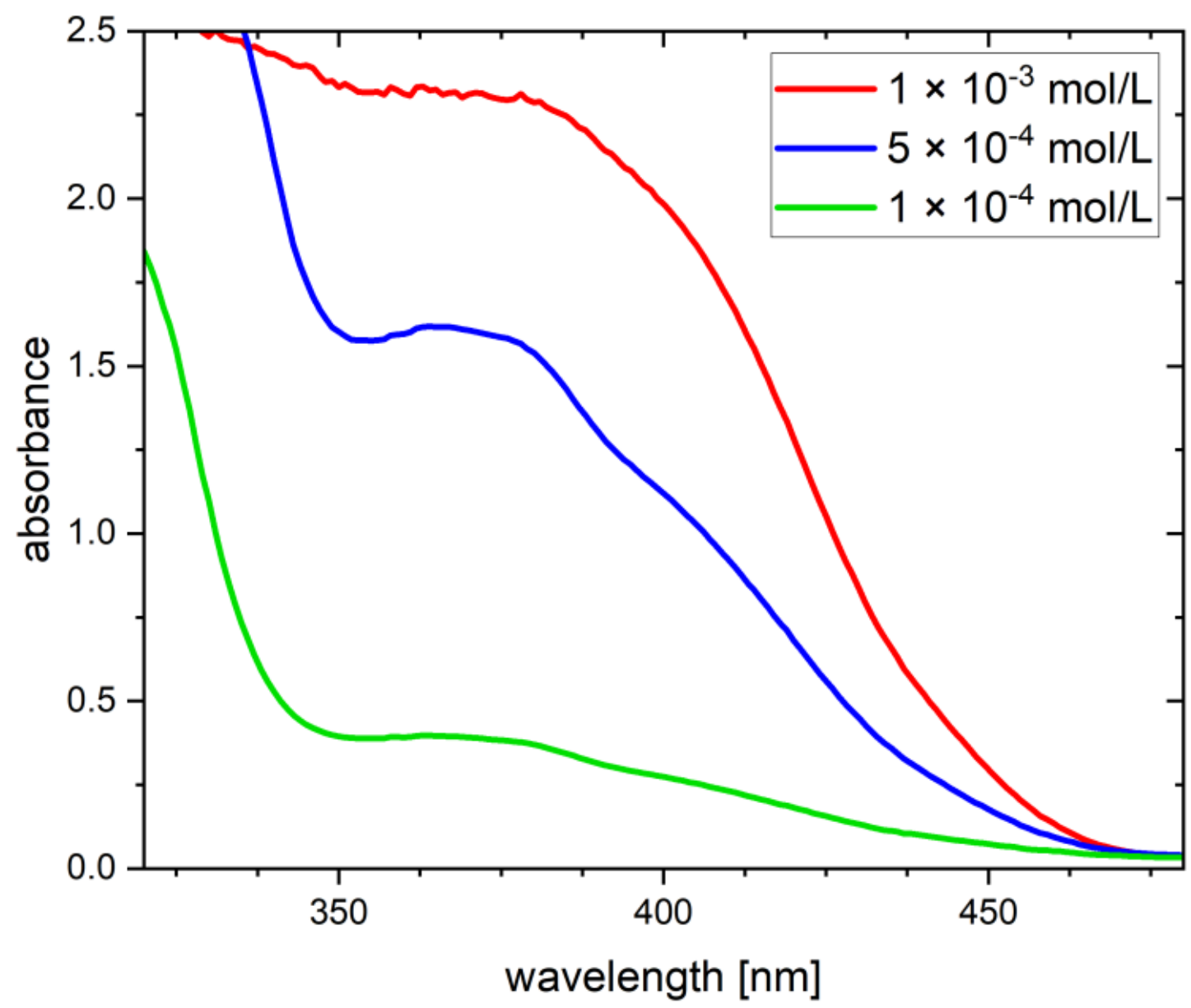

Figure S91. UV-vis spectra of **26** at different concentrations in THF.

Photo-DSC

The reactivity of the synthesized germanium photoinitiators was evaluated using Photo-Differential Scanning Calorimetry (Photo-DSC), with 10 mg of photoinitiator accurately weighed for each measurement. The resulting data were assessed in terms of the peak values, which reflect the maximum heat flow generated by the samples, and the area under the curve, which provides insight into the reaction kinetics—specifically, the time required to reach a defined conversion level (typically 95%). Photo-DSC analyses were performed using a Netzsch DSC 204 F1 Phoenix under isothermal conditions. The samples were exposed to two consecutive illumination cycles, each lasting 10 minutes. To account for the thermal contribution of the light source, the heat flow signal recorded during the second illumination cycle—conducted after completion of the photopolymerization reaction—was subtracted from the first cycle. Illumination was provided by a mercury medium-pressure lamp (OmniCure S2000, Lumen Dynamics), emitting within the spectral range of 250–650 nm at an intensity of 240 mW cm^{-2}.

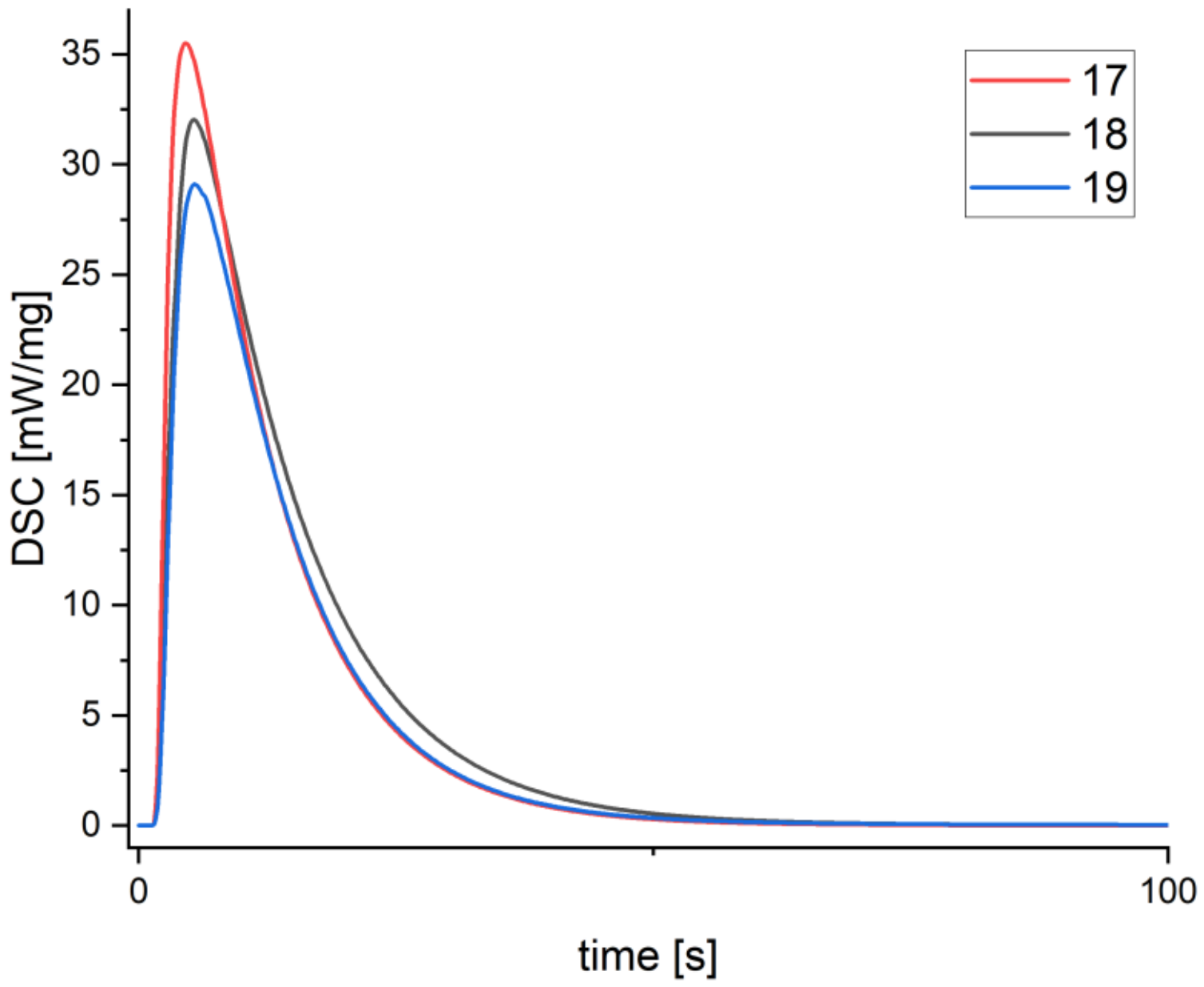

Figure S92. Photo-DSC curves of compounds **17–19**.

Photo-CIDNP

Photo-CIDNP and respective ^{1}H-NMR spectra were recorded on a Bruker Avance DPX spectrometer operating at 200 MHz for ^{1}H. Photo-CIDNP experiments were performed using a SpitLight 400 Nd:YAG laser (Innolas, Germany) operating at 355 nm, with a pulse duration of 8 ns, as the excitation source.

Background-free photo-CIDNP spectra were acquired using the following pulse sequence: background presaturation – laser flash – variable delay – observation RF pulse – signal acquisition. The variable delay between the laser flash and the detection pulse was adjusted to probe the evolution of spin polarization.

Prior to measurements, all samples were degassed by bubbling with nitrogen for 5 minutes to remove dissolved oxygen and minimize triplet quenching.

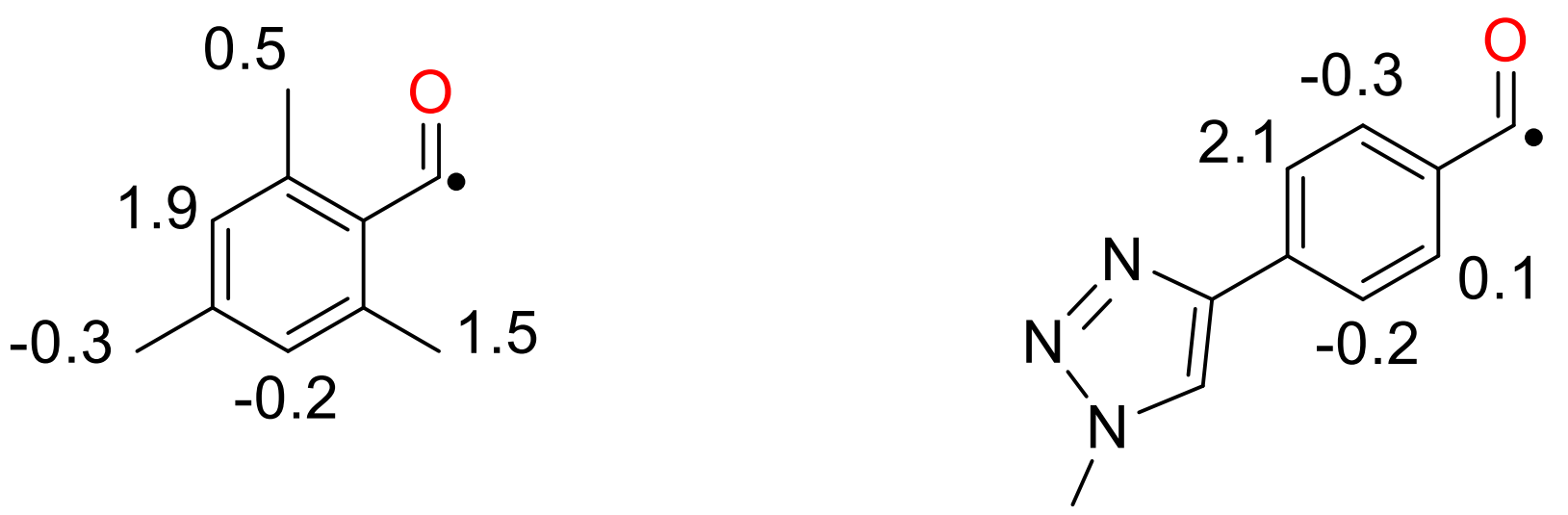

Figure S93. ^{1}H hyperfine coupling constants (Gauss) in selected free radicals calculated on B3LYP/TZVP level of theory.

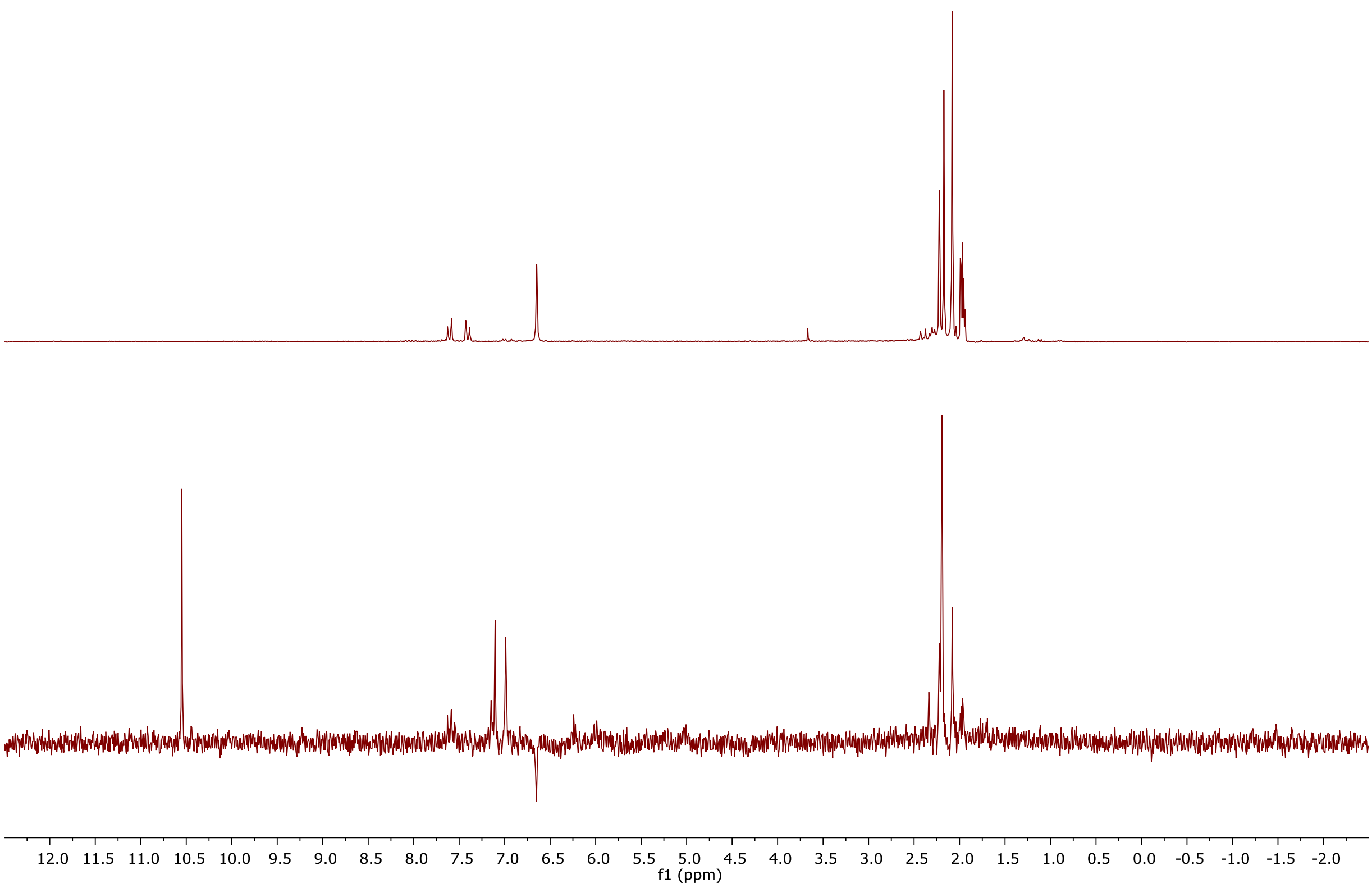

Figure S94. ^{1}H NMR (top) and photo-CIDNP of **2** recorded in Acetonitrile-d_3.

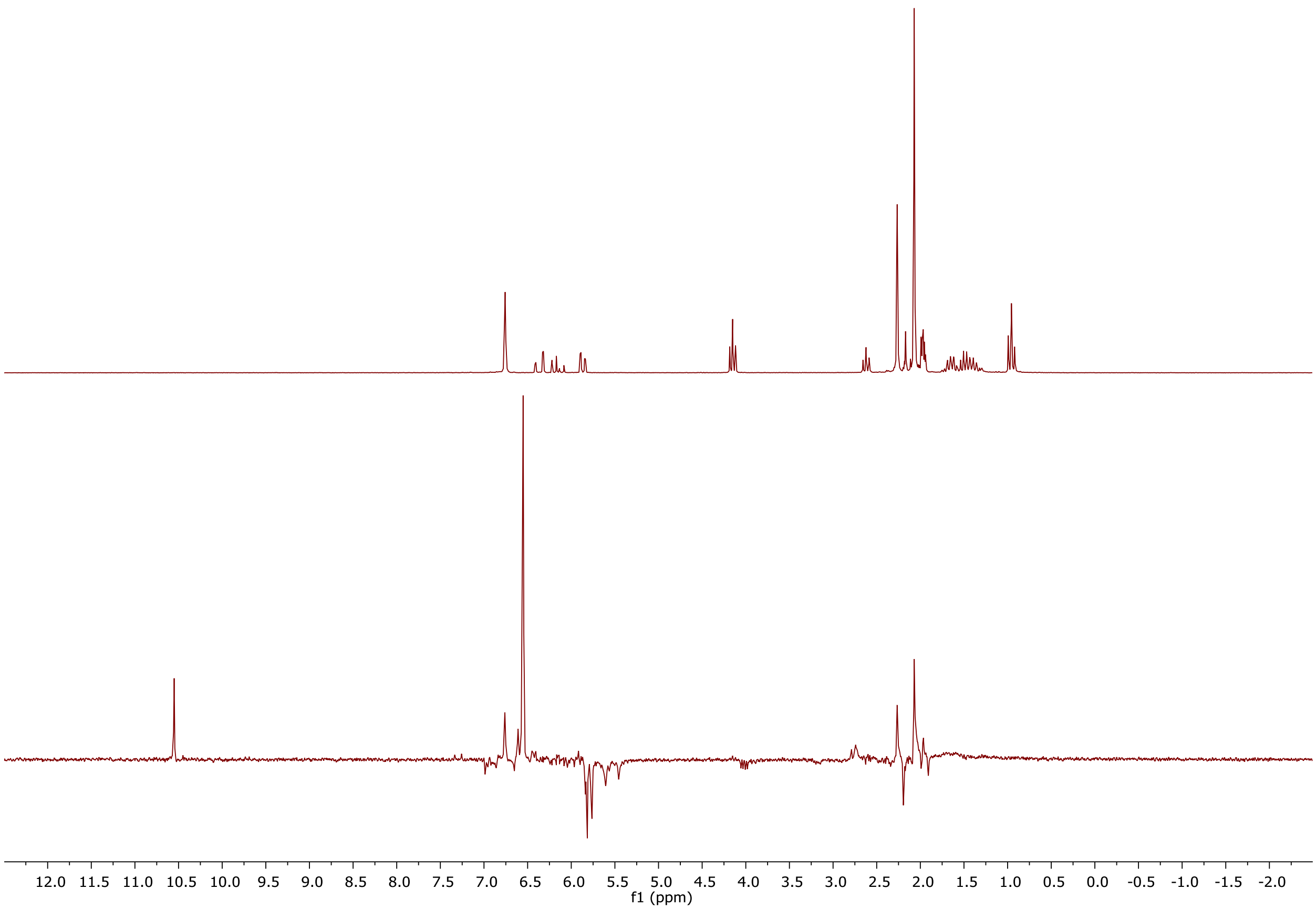

Figure S95. ^{1}H NMR (top) and photo-CIDNP of **2** recorded in Acetonitrile-d_3 in the presence of quencher (butyl acrylate).

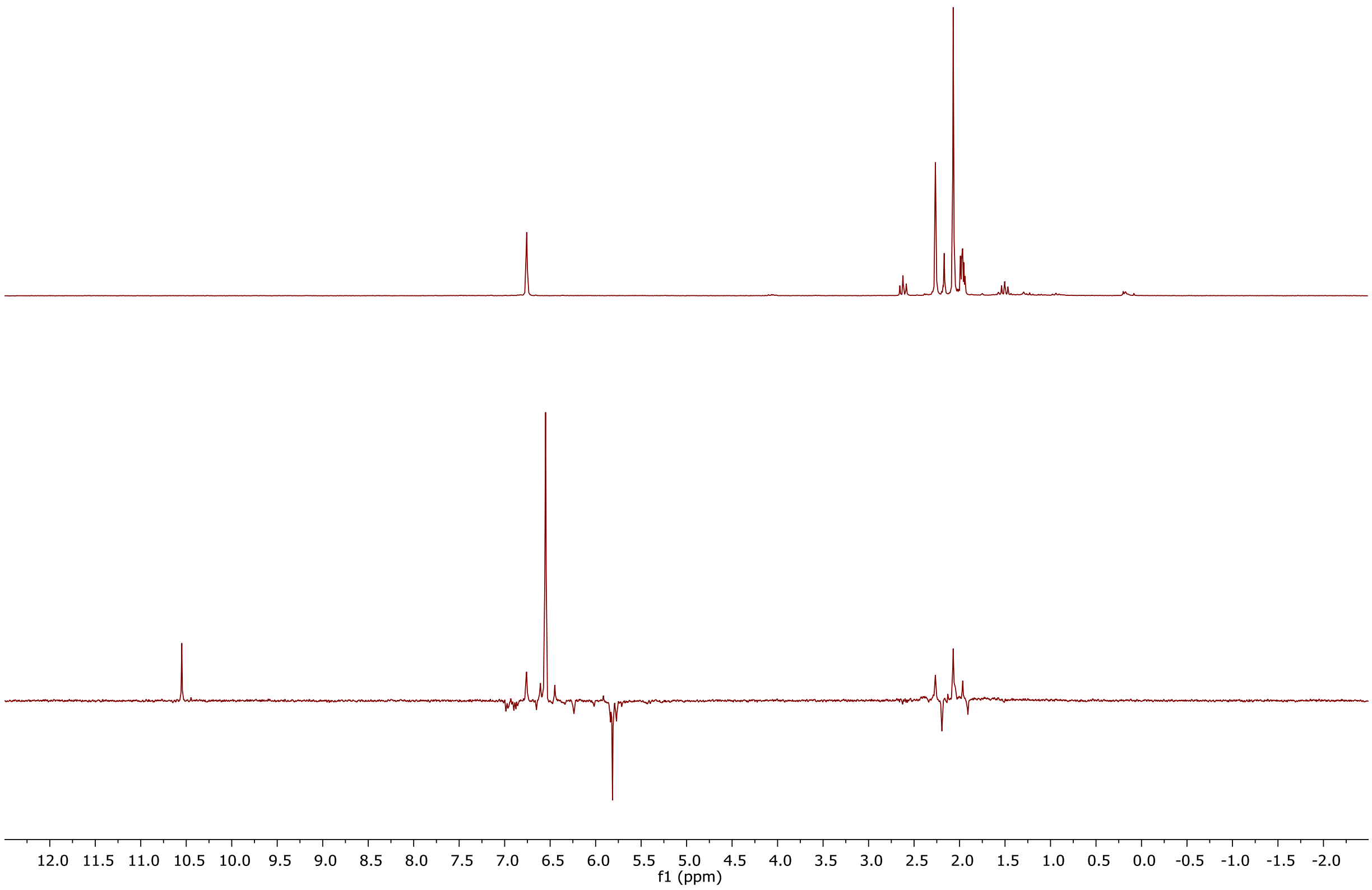

Figure S96. ^{1}H NMR (top) and photo-CIDNP of **3** recorded in Acetonitrile-d_3.

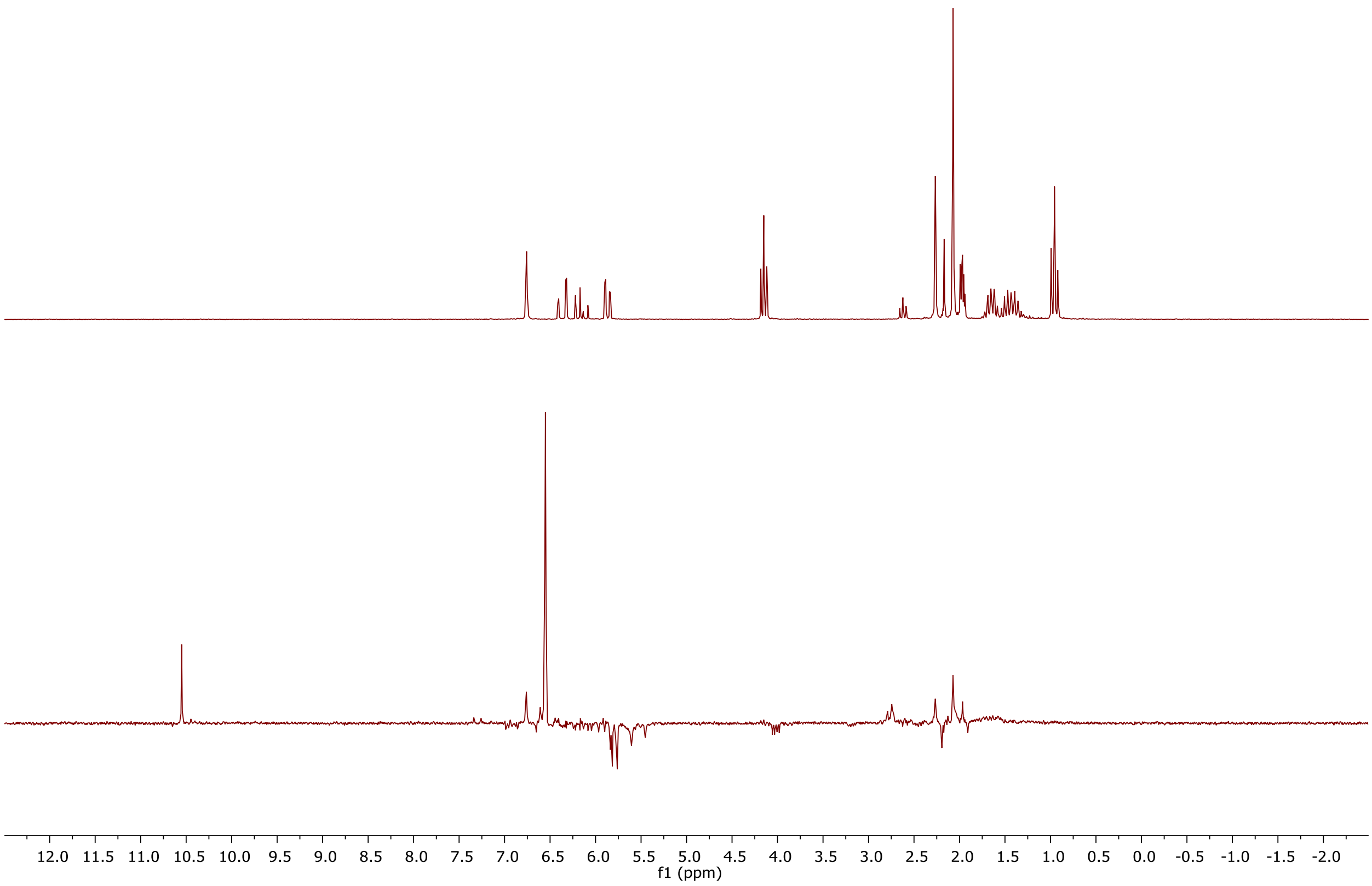

Figure S 97. ^{1}H NMR (top) and photo-CIDNP of **3** recorded in Acetonitrile-d_3 in the presence of quencher (butyl acrylate).

DFT Calculations

Computational Methods

TDDFT single point computations were performed on the Xray geometries of compounds **1**, **3**, **4** and **14**. **20** vertical excitations were computed applying the CAM-B3LYP/def2-TZVP/CPCM(THF) method.[75-77] Gaussian broadening of 2500 cm^{-1} was applied for the simulation of the UV/Vis spectra. The program Gaussian16 was used for all calculations.[78] The natural transition orbitals (NTOs) were drawn with the program MultiWfn.[79]

Simulated Absorption Spectrum:

Table S3. Computed CAM-B3LYP/def2-TZVP/CPCM(THF) S_1-S_6 vertical excitation wavelengths (in nm) and oscillator strengths (in brackets) of the xray structures of **1**, **3**, **4**, **14** and **20**.

	**1**	**3**	**4**	**14**	**20**
S_1	389.15 (0.0100)	385.33 (0.0017)	391.63 (0.0154)	402.2 (0.0176)	389.89 (0.0116)
S_2	373.48 (0.0038)	371.40 (0.0034)	376.45 (0.0015)	379.62 (0.0035)	377.24 (0.0012)
S_3	364.13 (0.0112)	361.07 (0.0071)	365.18 (0.0055)	372.46 (0.0037)	371.02 (0.0047)
S_4	280.35 (0.4065)	343.79 (0.0213)	359.89 (0.0083)	362.28 (0.0141)	362.68 0.0142)
S_5	275.31 (0.0571)	280.45 (0.2023)	297.75 (0.0011)	296.52 (1.0873)	290.96 (0.4057)
S_6	273.43 (0.1654)	276.03 (0.2216)	292.19 (0.7838)	290.1 (0.1375)	288.53 (0.6760)

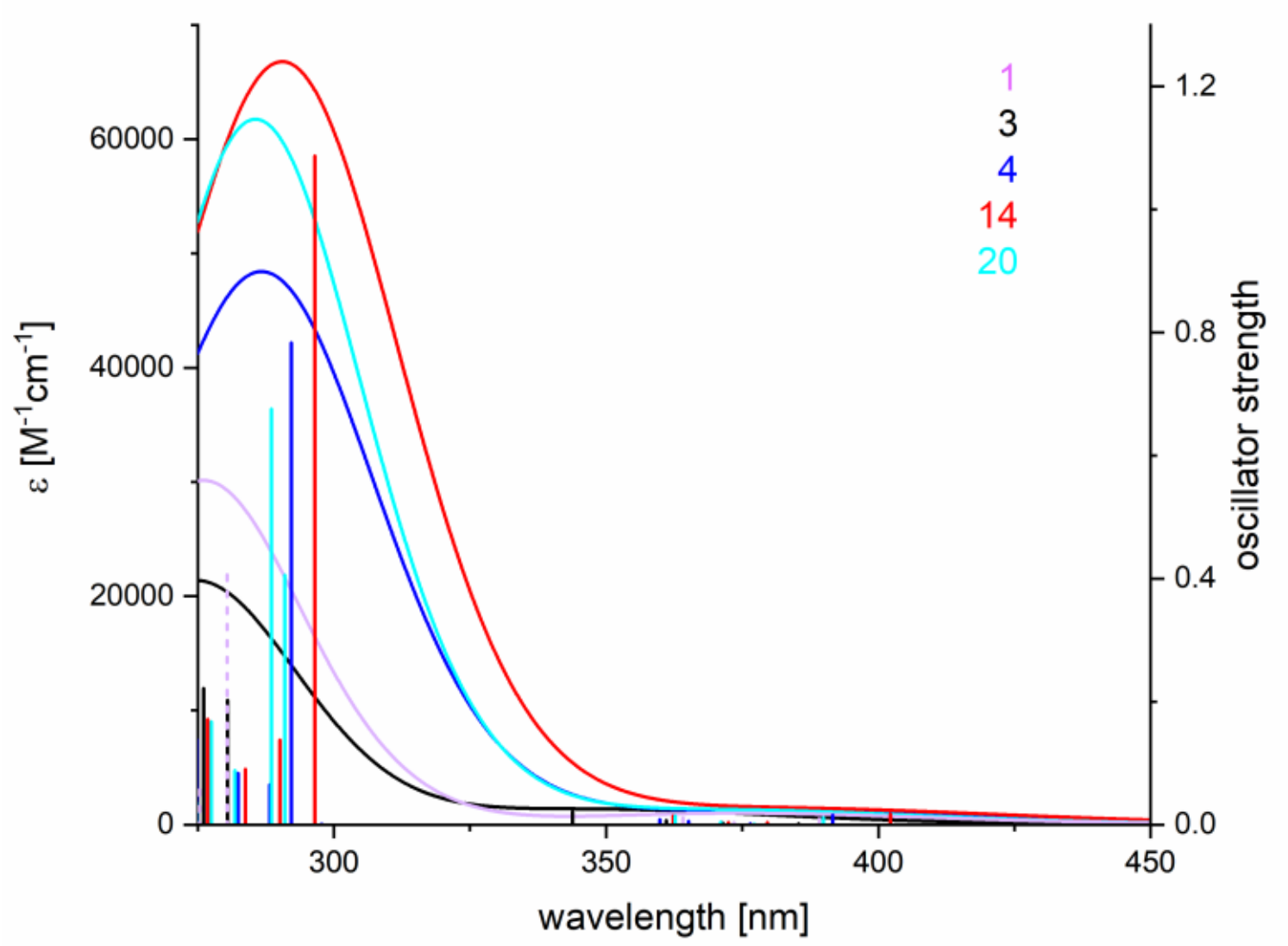

Figure S98. Simulated absorption spectrum of xray geometries **1**, **3**, **4**, **14** and **20** using the CAM-B3LYP/def2-TZVP/CPCM(THF) method. A Gaussian broadening of 2500 cm^{-1} was applied for the simulation of the spectrum.

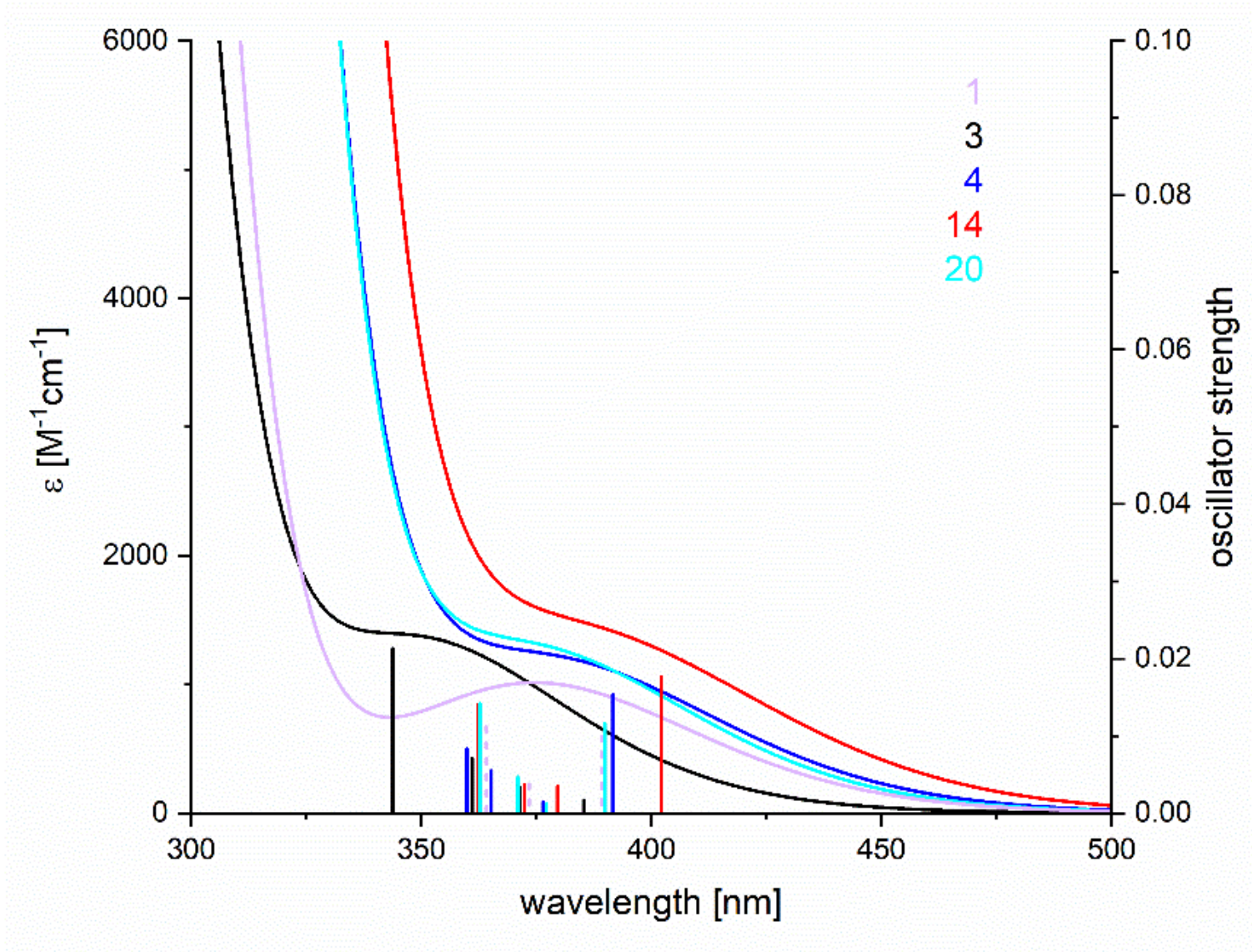

Figure S99. Increased view to the long-wavelength band of the simulated absorption spectrum of compounds **1**, **3**, **4**, **14** and **20**. CAM-B3LYP/def2-TZVP/CPCM(THF) method with a Gaussian broadening of 2500 cm^{-1}.

Table S4. NTOs and their coefficients (c>0.1) for the respective excitations S_1-S_5 of **1**. The first three excitations are n-π* transitions, S_4 and S_5 are π-π* transitions. Contour values of the NTOs are 0.04 a.u.

1	c(NTO)	from		to
**S_1**	0.17	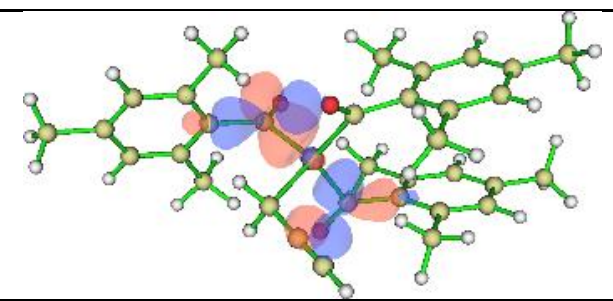	→	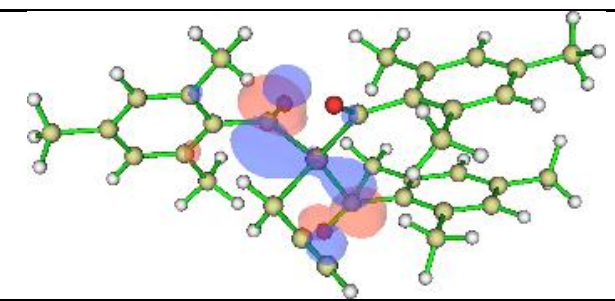
	0.75	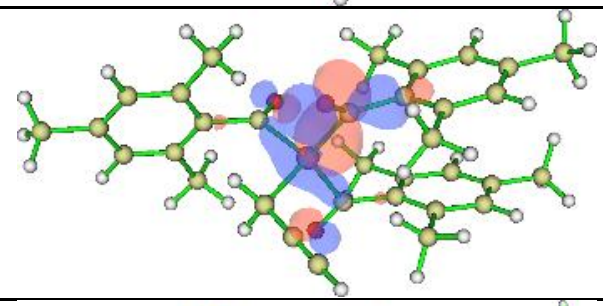	→	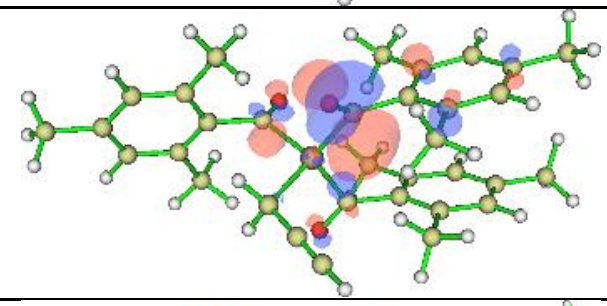
**S_2**	0.39	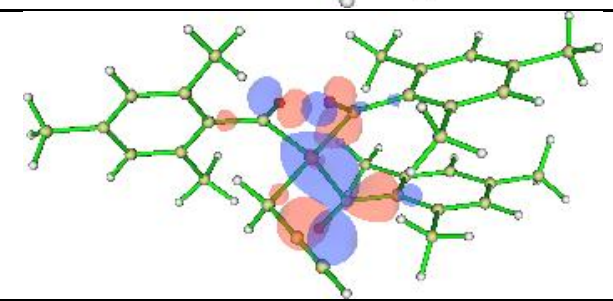	→	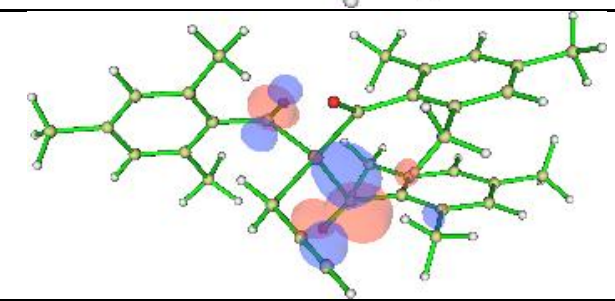
	0.60	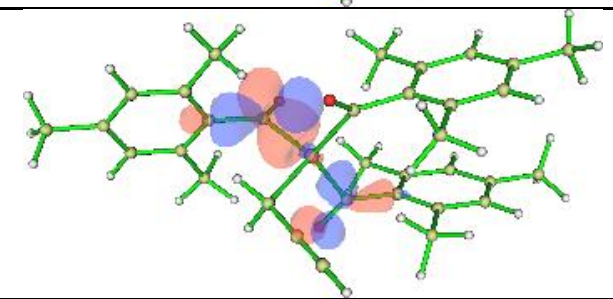	→	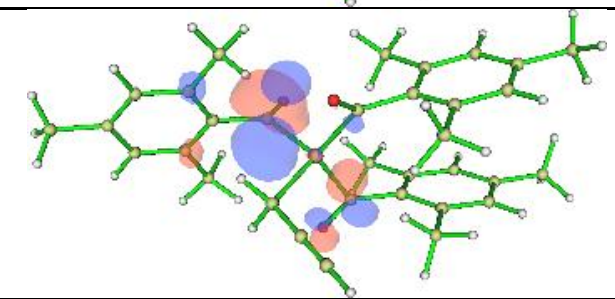
**S_3**	0.17	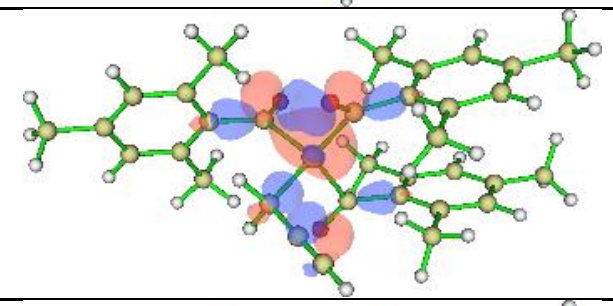	→	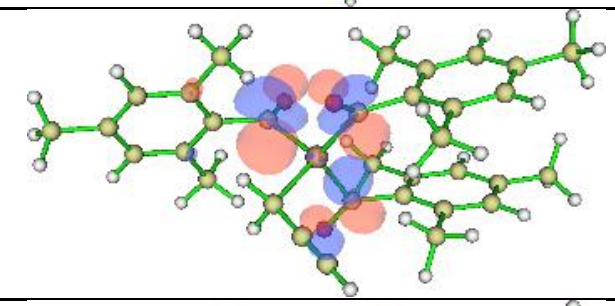
	0.30	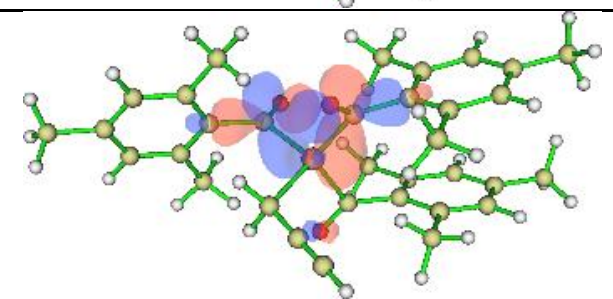	→	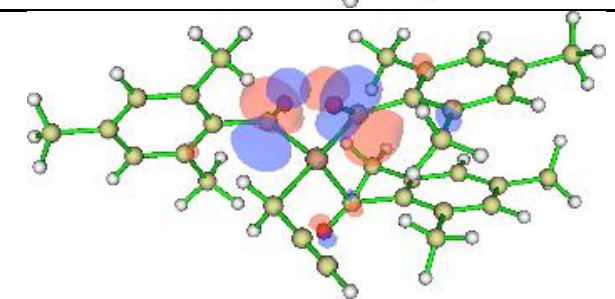
	0.75	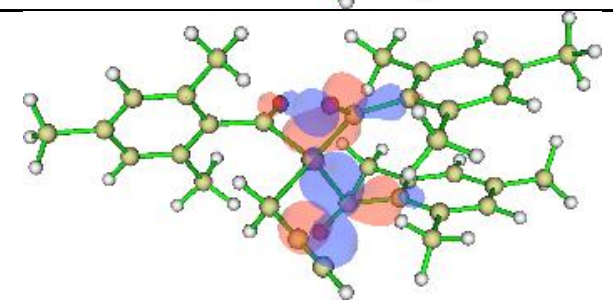	→	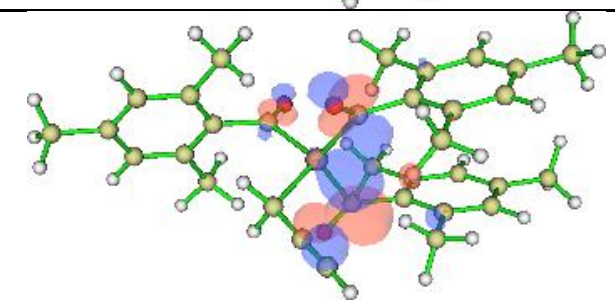
**S_4**	0.24	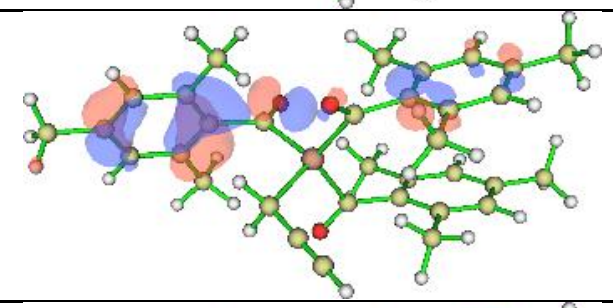	→	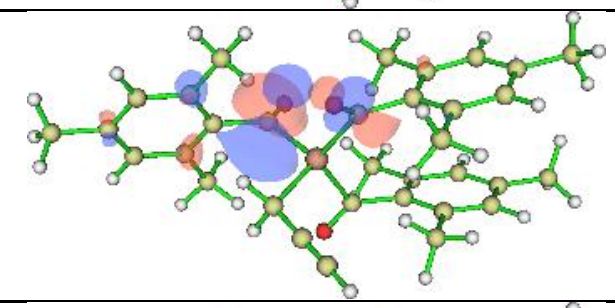
	0.68	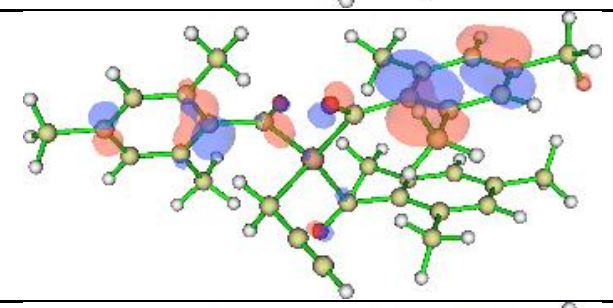	→	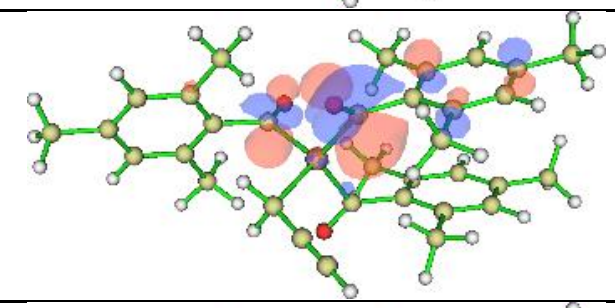
**S_5**	0.29	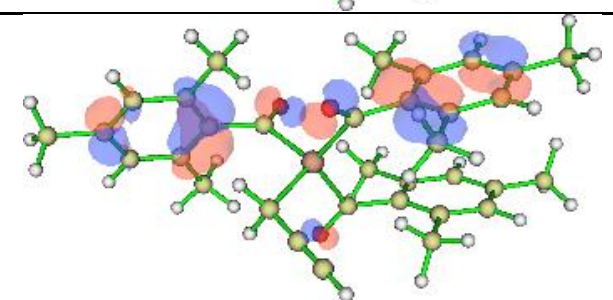	→	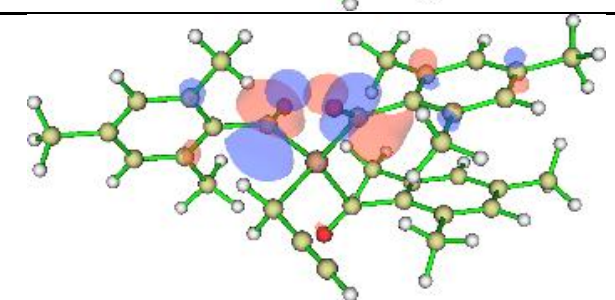
	0.63	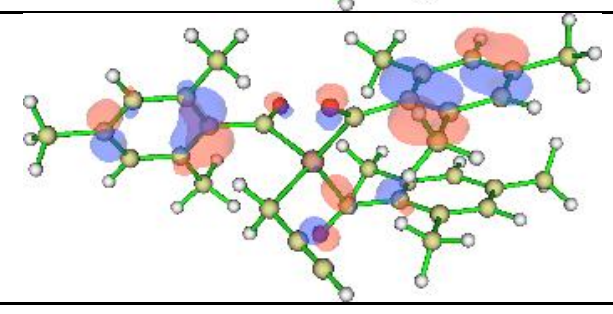	→	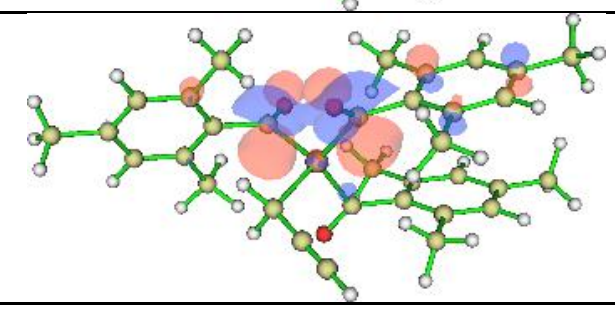

Table S5. NTOs and their coefficients (c>0.1) for the respective excitations S_1-S_5 of **3**. The first four excitations are n-π* transitions, S_5 and S_6 are π-π* transitions. Contour values of the NTOs are 0.04 a.u.

**3**	c(NTO)	from		to
**S_1**	0.18	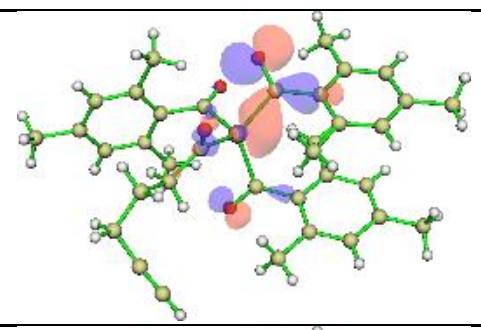	→	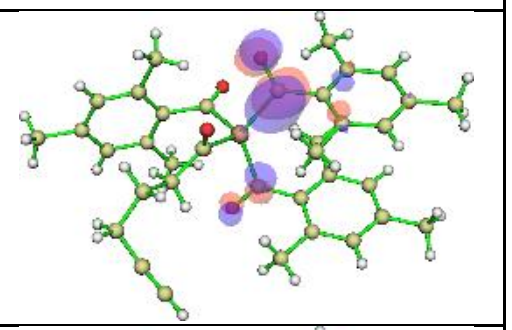
	0.77	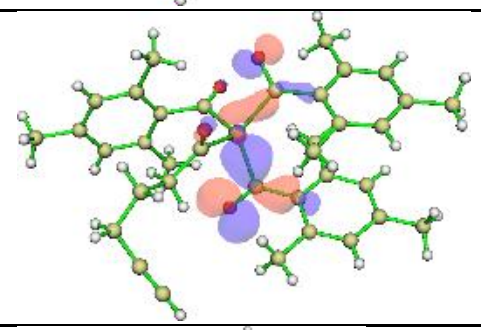	→	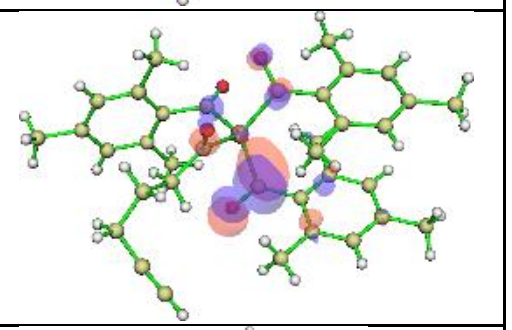
**S_2**	0.14	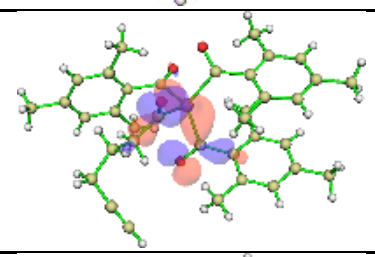	→	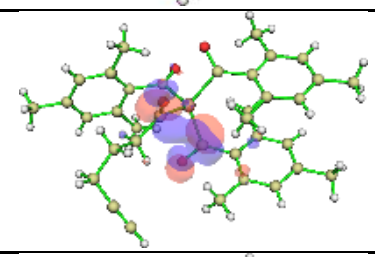
	0.19	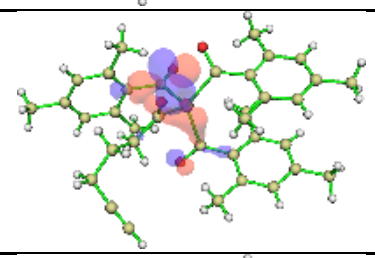	→	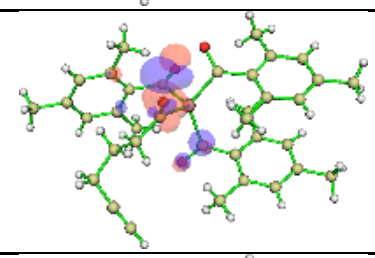
	0.64	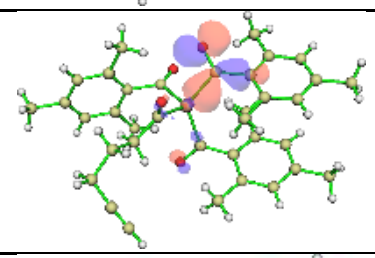	→	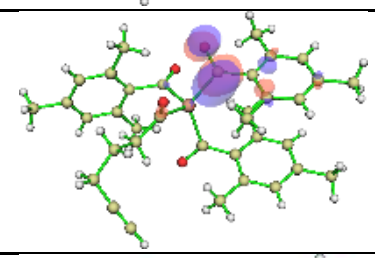
**S_3**	0.18	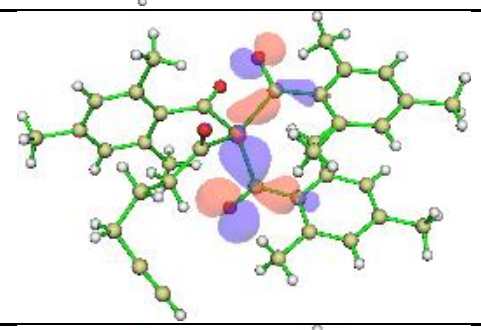	→	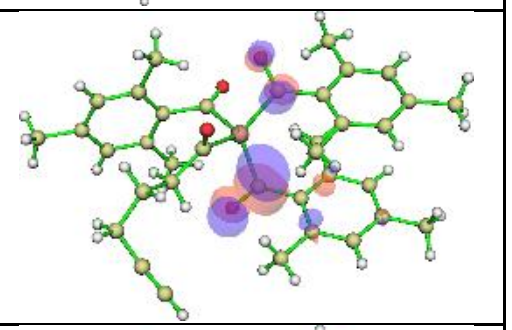
	0.75	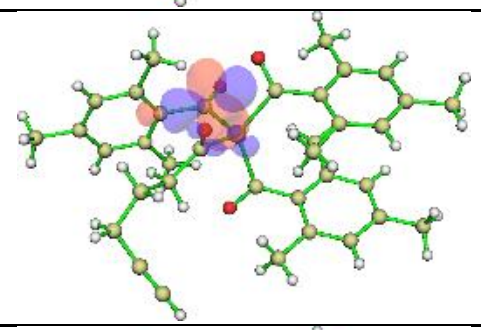	→	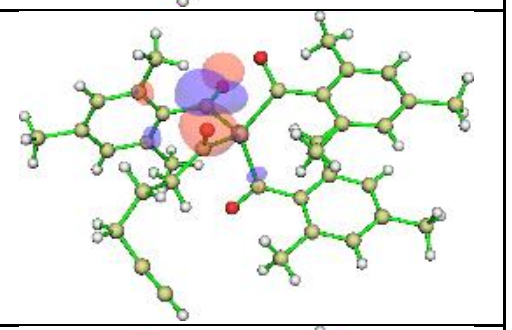
**S_4**	0.85	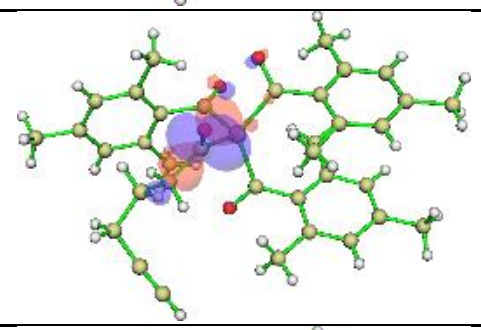	→	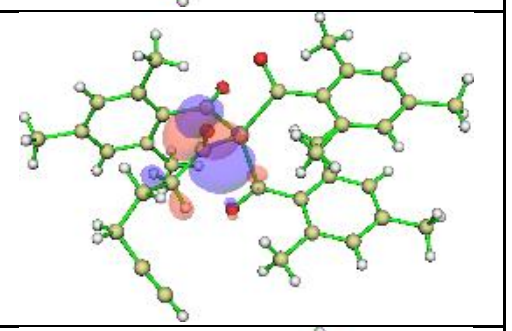
**S_5**	0.89	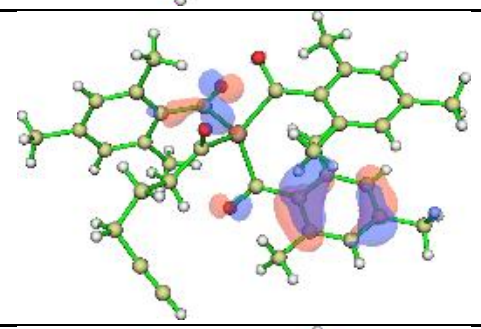	→	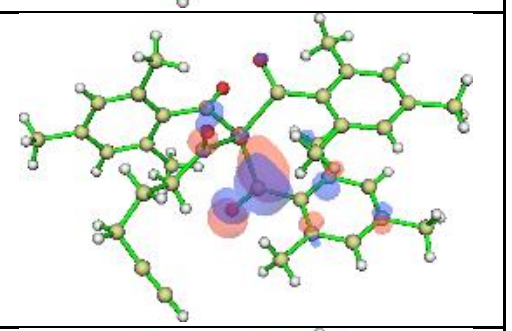
**S_6**	0.90	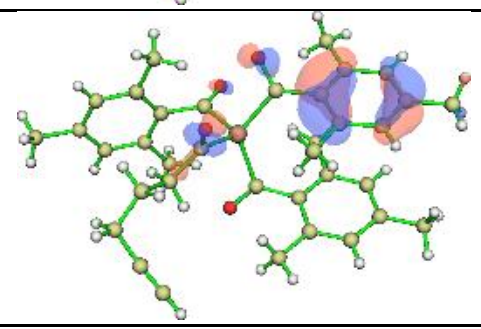	→	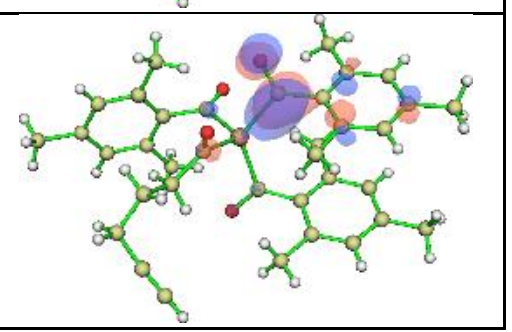

Table S6. NTOs and their coefficients (c>0.1) for the respective excitations S_1-S_5 of **4**. The first four excitations are n-π* transitions, S_5 and S_6 are π-π* transitions. Contour values of the NTOs are 0.04 a.u.

4	c(NTO)	from		to
**S_1**	0.25	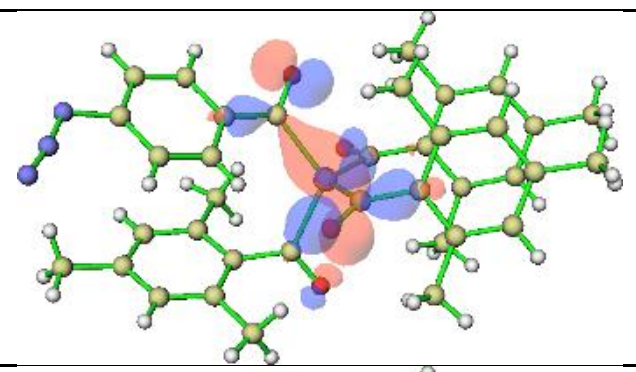	→	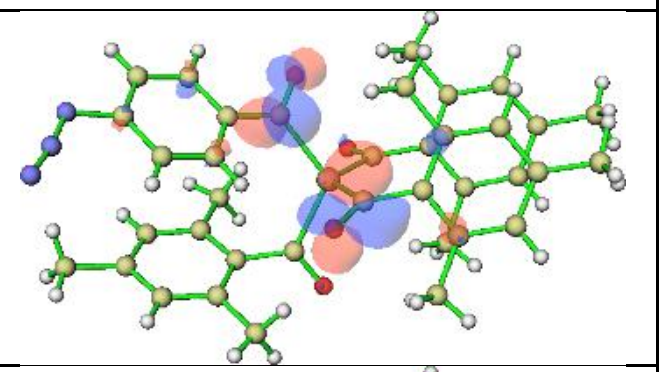
	0.71	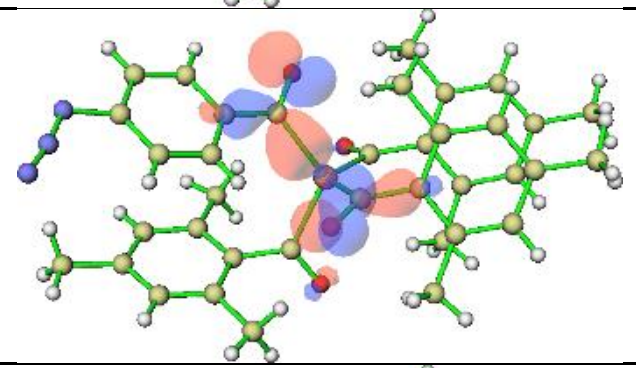	→	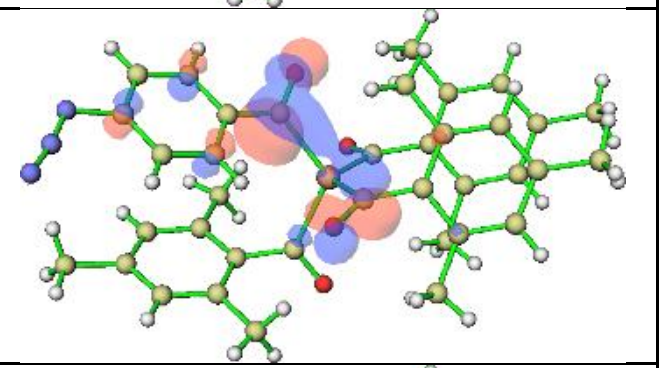
**S_2**	0.15	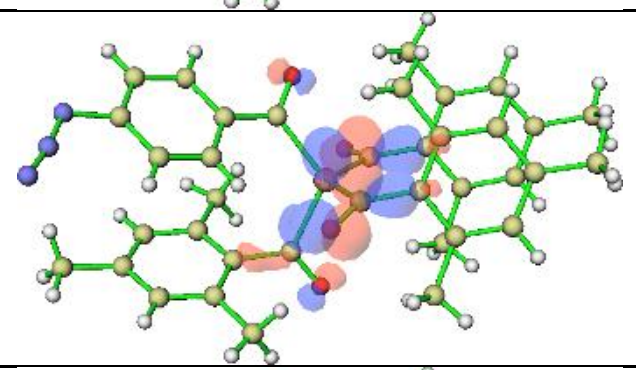	→	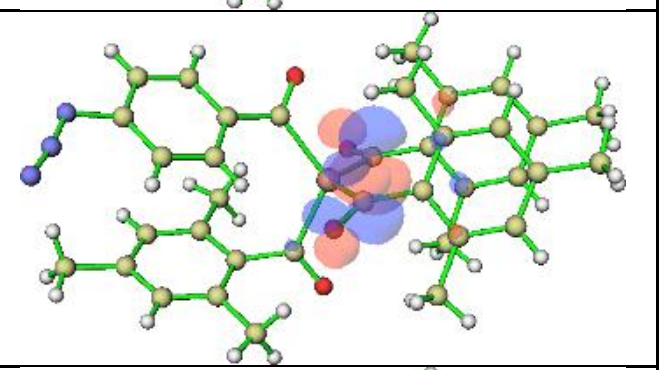
	0.34	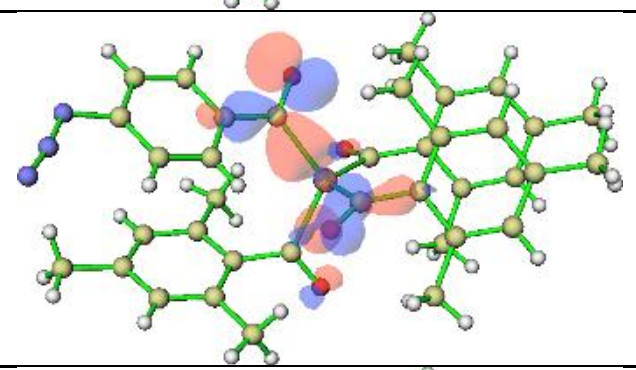	→	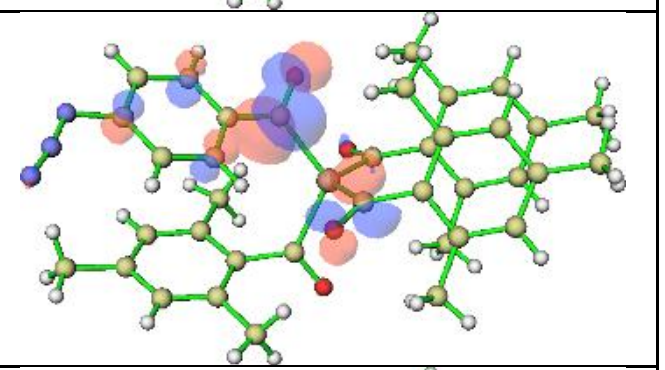
	0.50	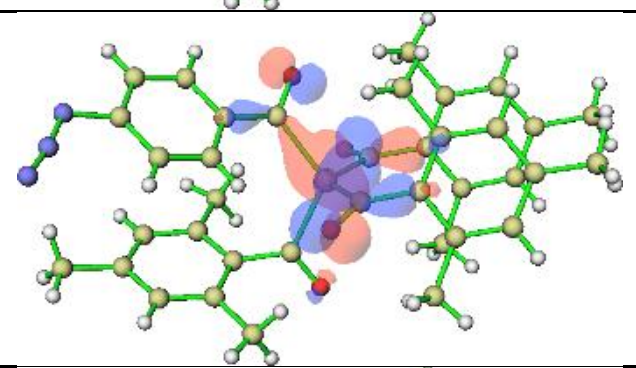	→	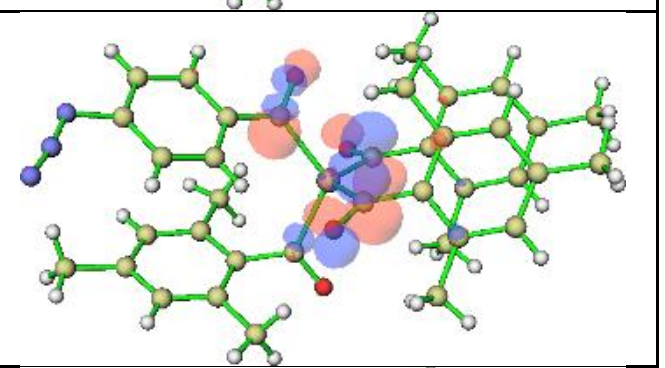
**S_3**	0.12	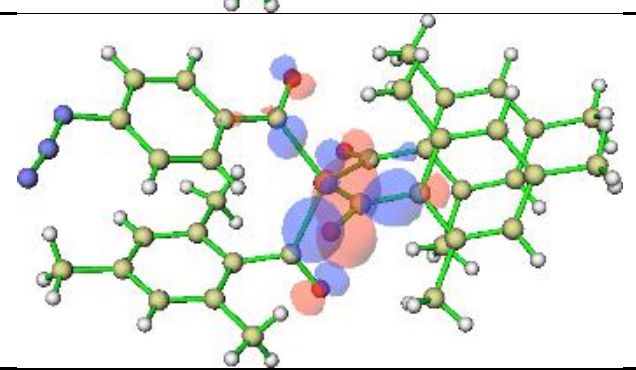	→	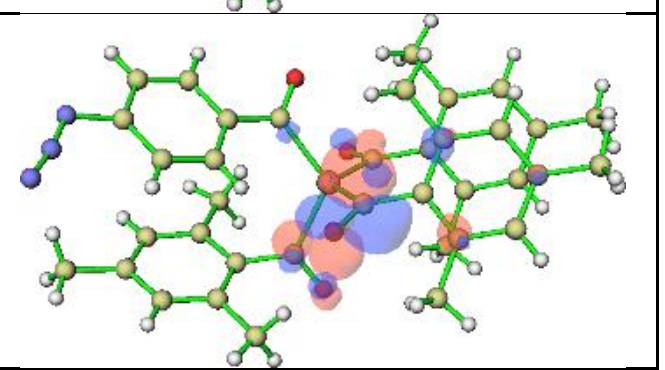
	0.35	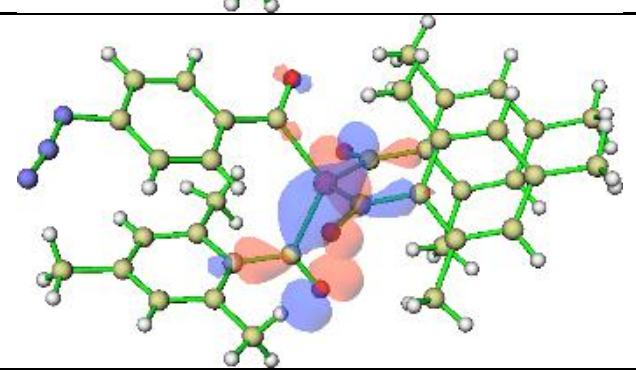	→	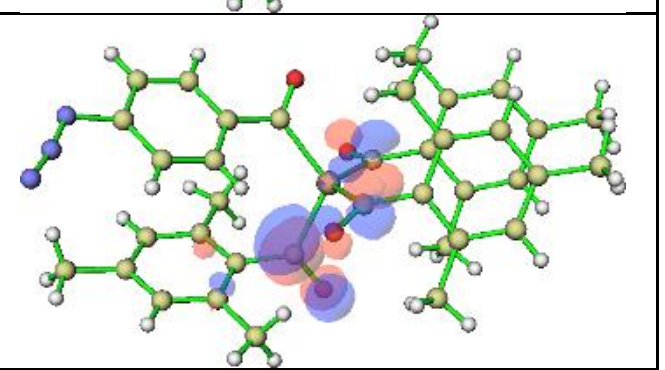
	0.53	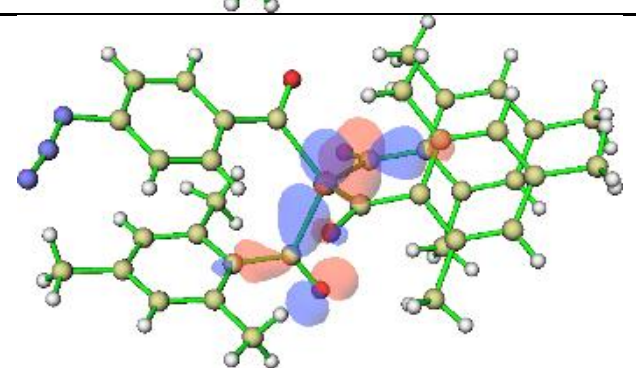	→	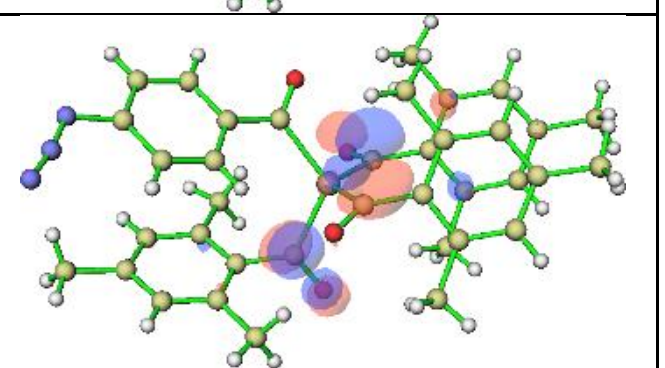
**S_4**	0.10	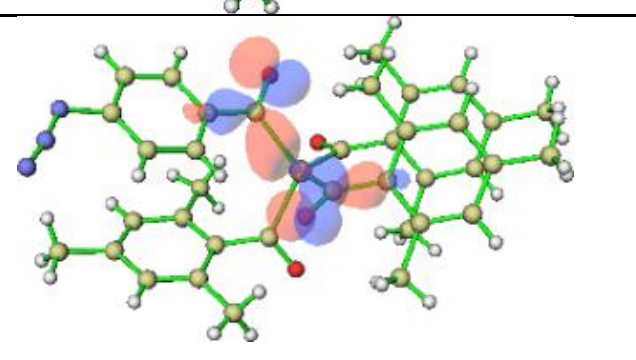	→	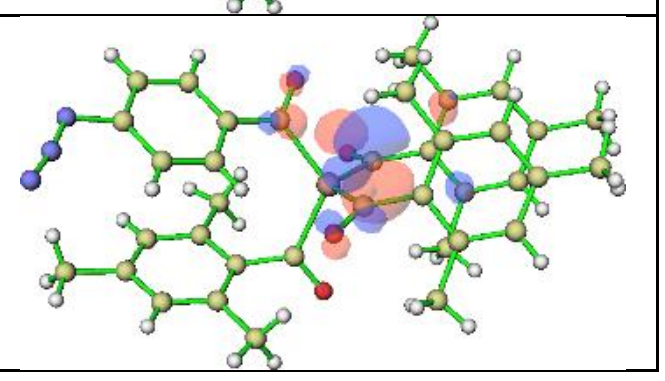

	0.26	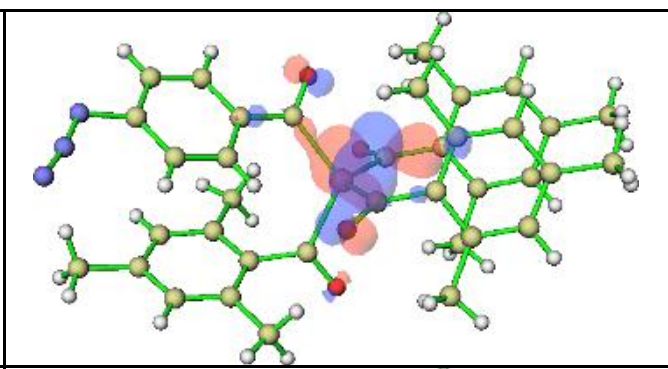	→	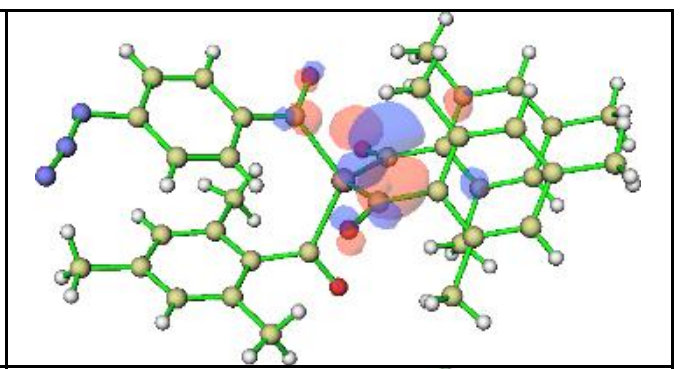
	0.59	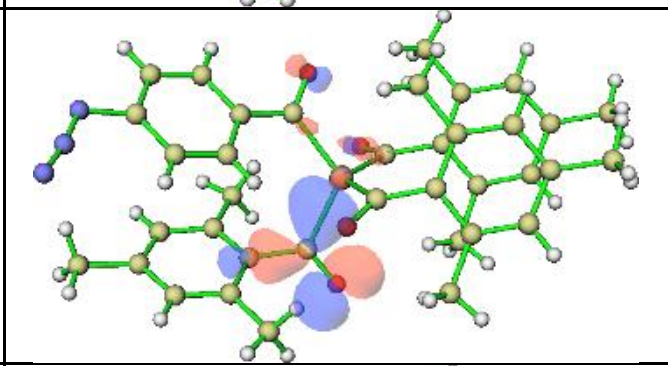	→	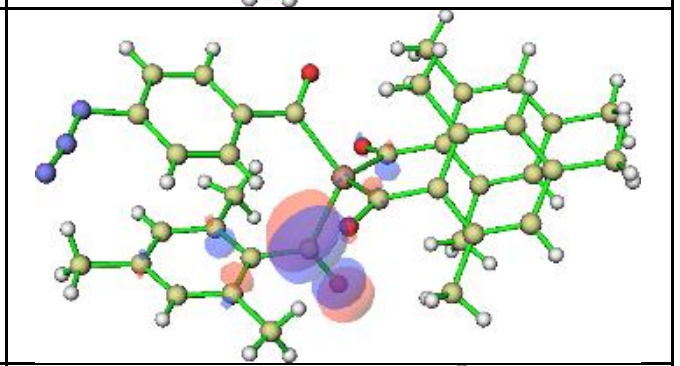
**S**$_5$	1.00	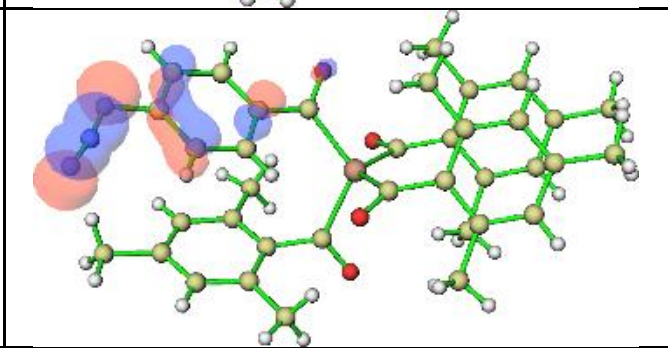	→	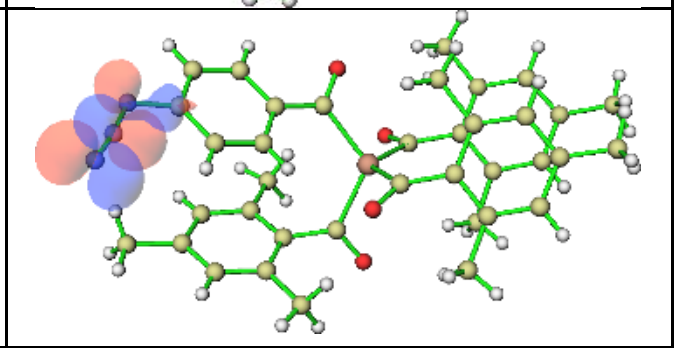
**S**$_6$	0.87	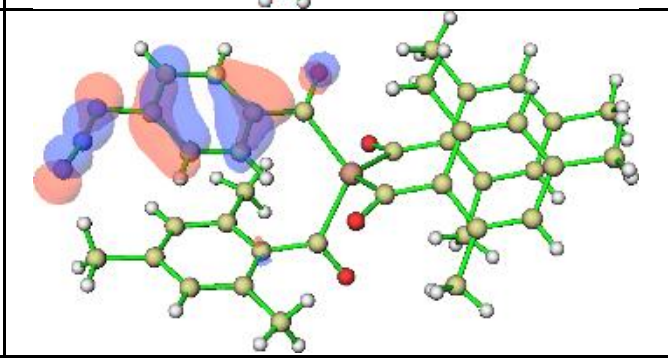	→	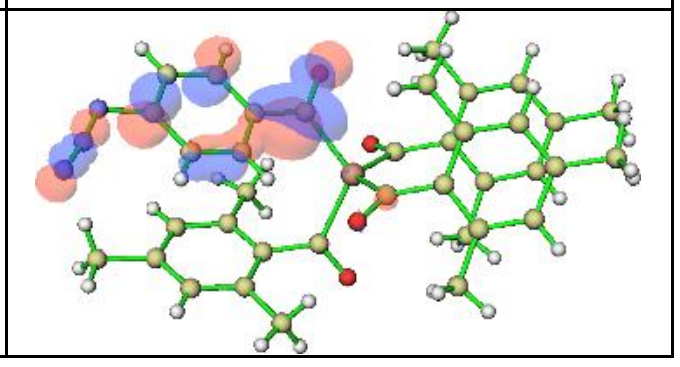

Table S7. NTOs and their coefficients (c>0.1) for the respective excitations S$_1$-S$_5$ of **14**. The first four excitations are n-π* transitions, S$_5$ and S$_6$ are π-π* transitions. Contour values of the NTOs are 0.04 a.u.

**14**	**c(NTO)**	**from**		**to**
**S**$_1$	0.17	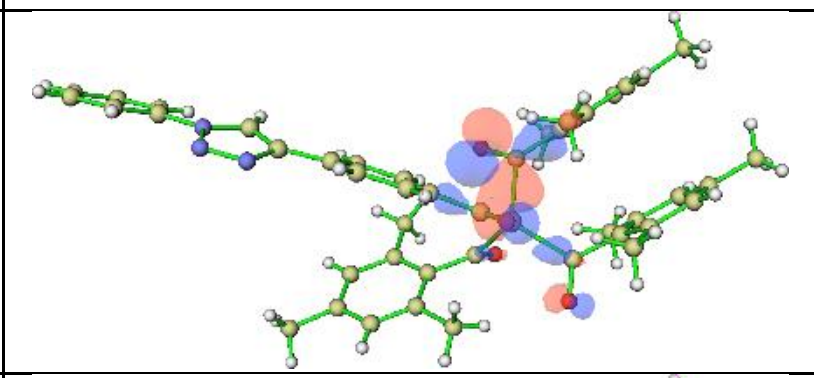	→	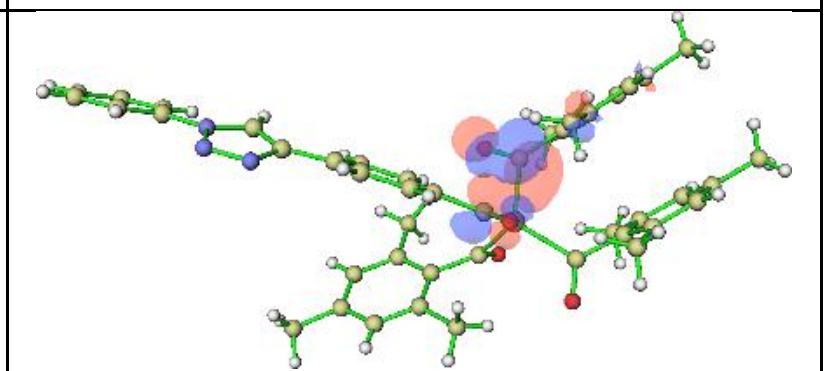
	0.81	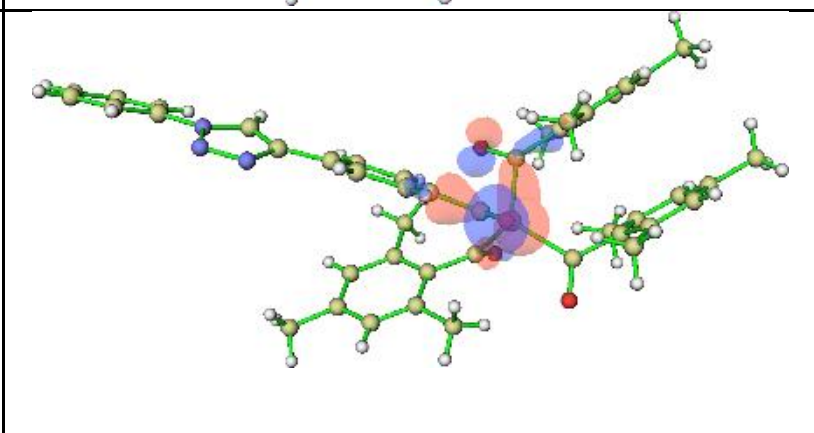	→	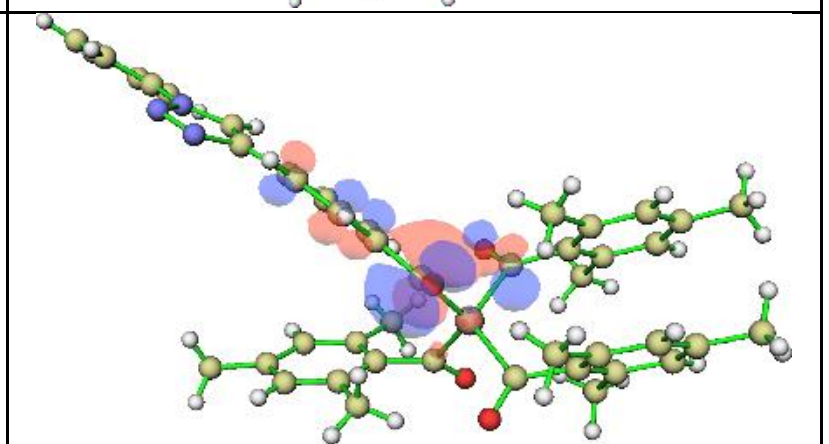
**S**$_2$	0.15	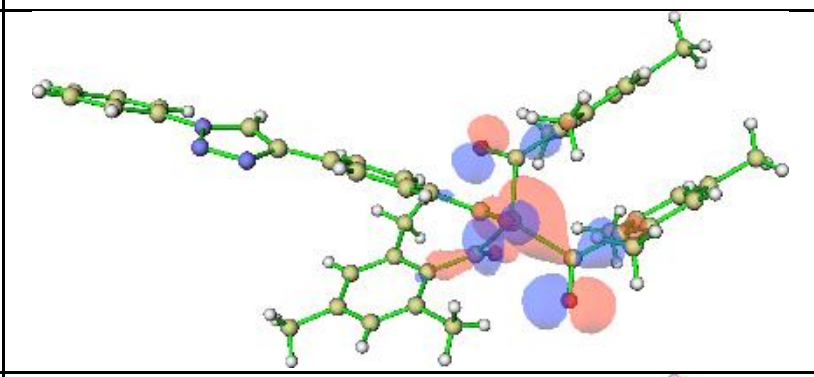	→	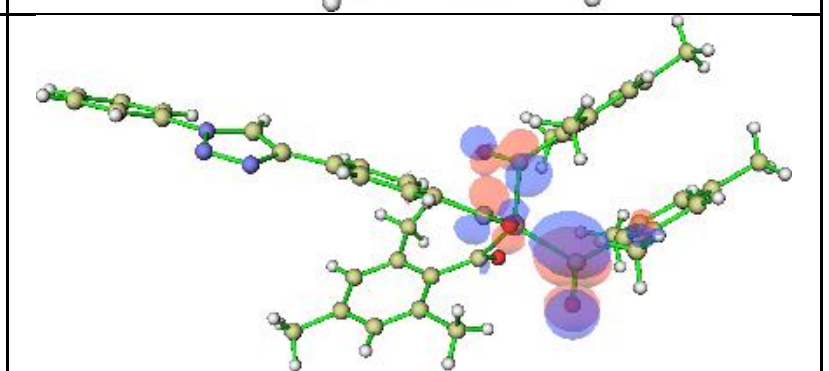
	0.26	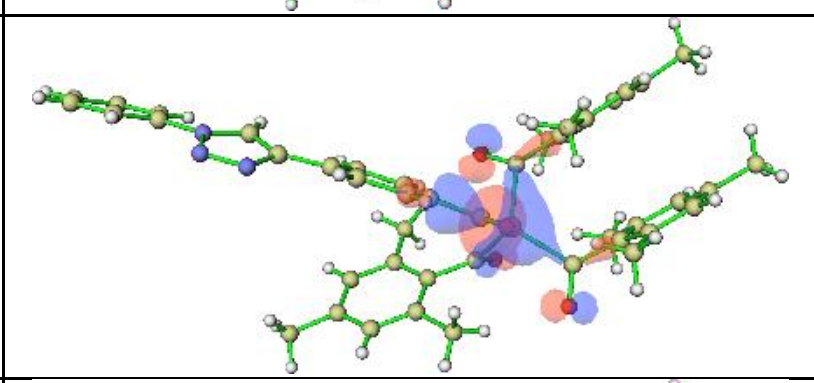	→	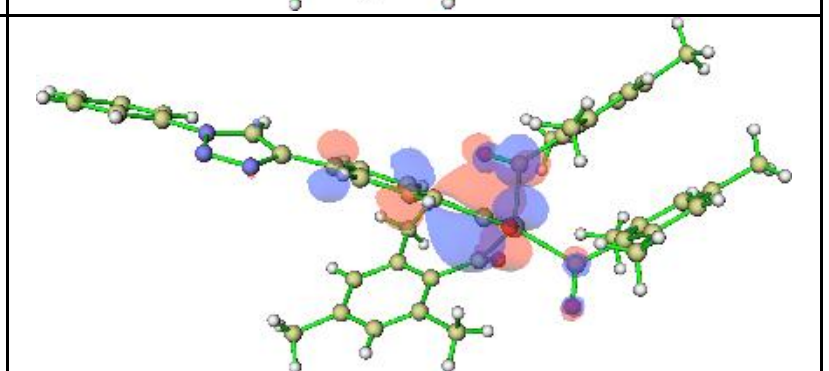
	0.58	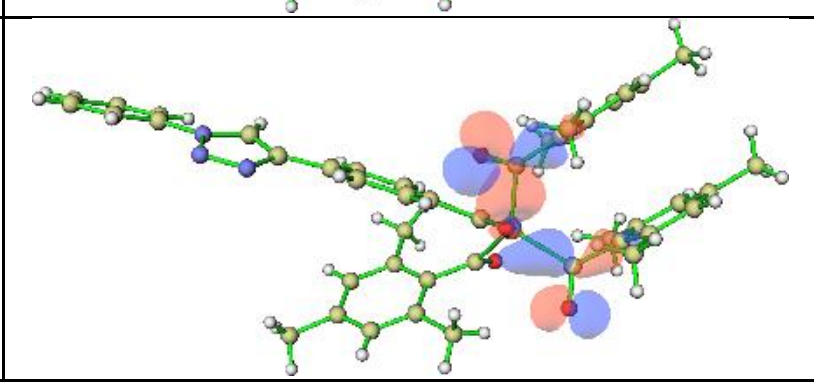	→	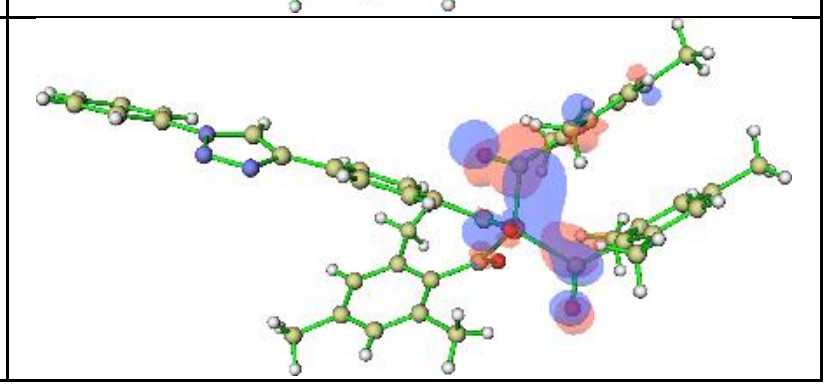

**S_3**	0.89	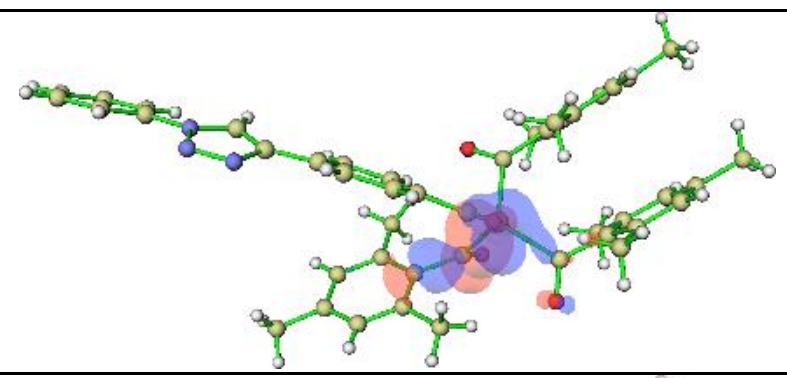	→	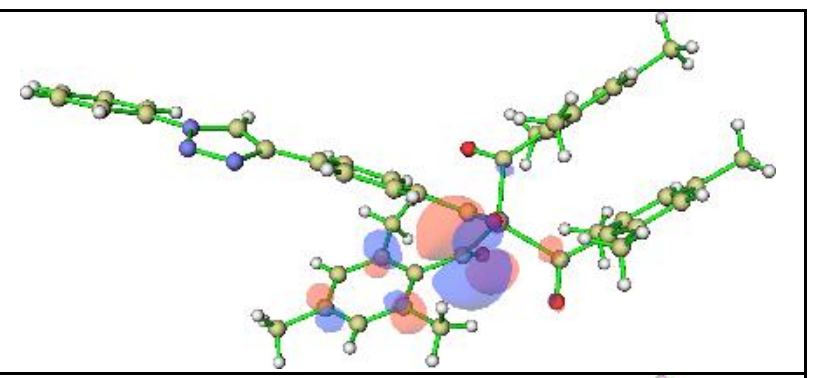
**S_4**	0.10	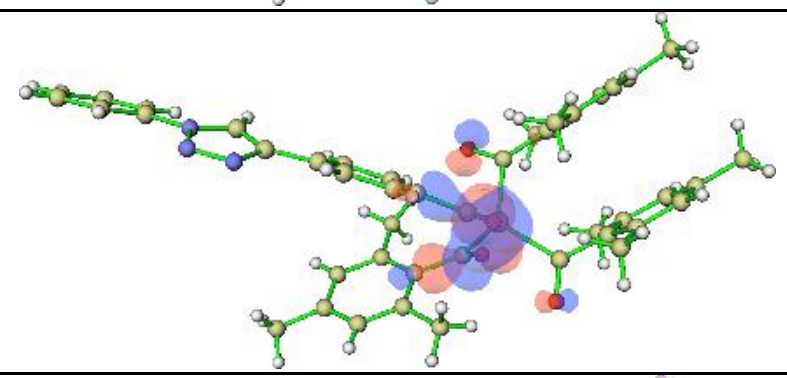	→	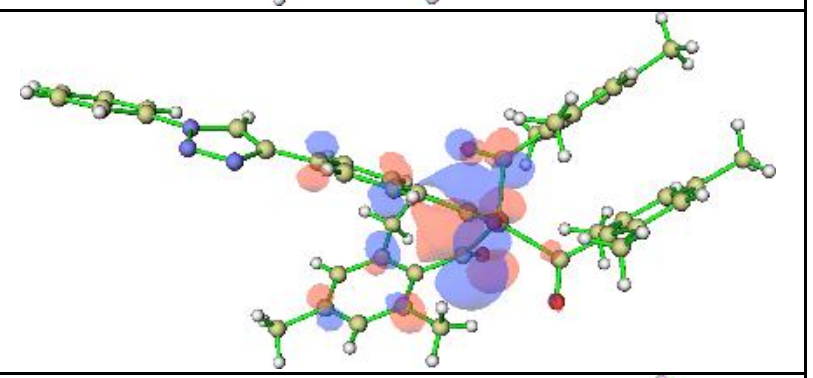
	0.20	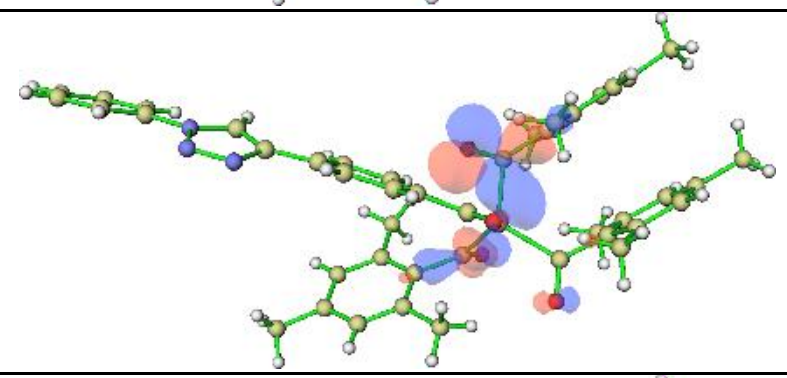	→	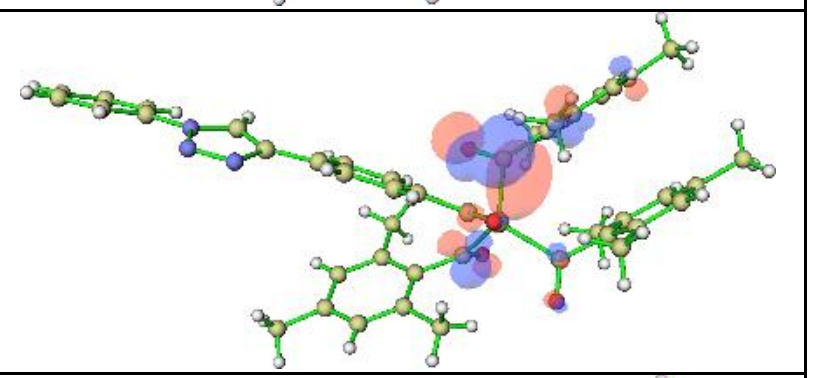
	0.66	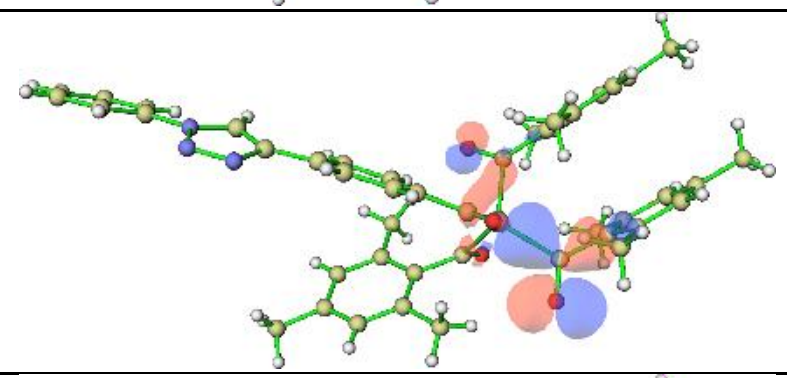	→	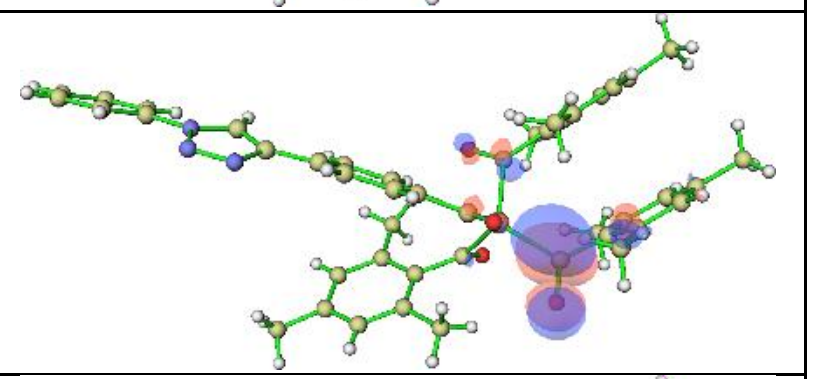
**S_5**	0.89	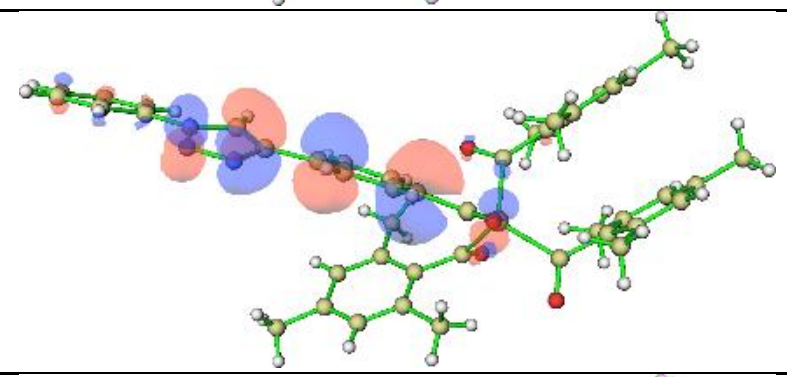	→	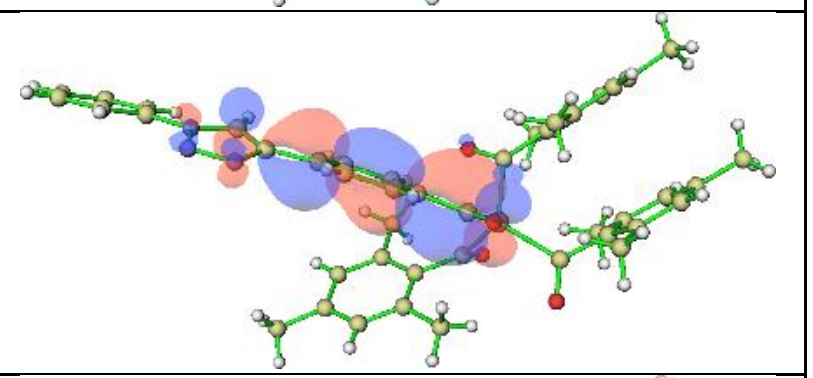
**S_6**	0.85	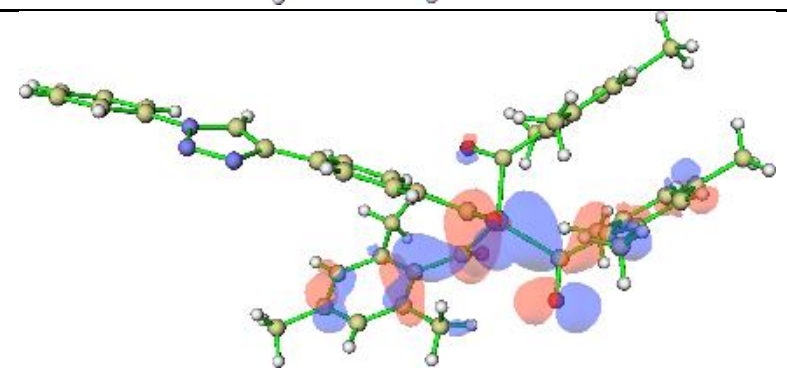	→	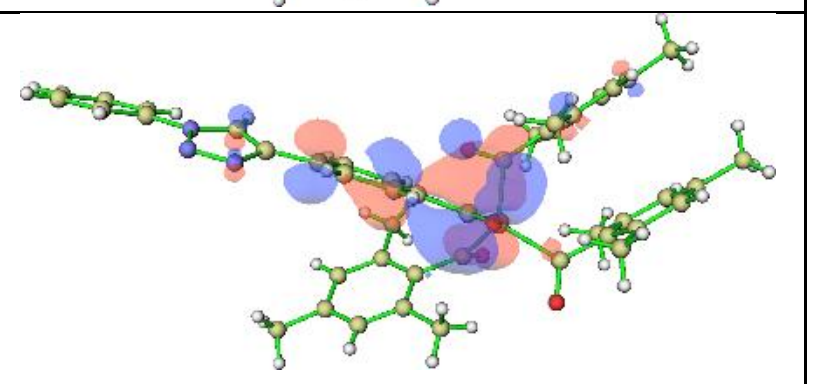
